# Phagosomal RNA sensing through TLR8 controls susceptibility to tuberculosis

**DOI:** 10.1016/j.celrep.2025.115657

**Published:** 2025-05-06

**Authors:** Charlotte Maserumule, Charlotte Passemar, Olivia S.H. Oh, Kriztina Hegyi, Karen Brown, Aaron Weimann, Adam Dinan, Sonia Davila, Catherine Klapholz, Josephine Bryant, Deepshikha Verma, Jacob Gadwa, Shivankari Krishnananthasivam, Kridakorn Vongtongsalee, Edward Kendall, Andres Trelles, Martin L. Hibberd, Joaquín Sanz, Jorge Bertol, Lucia Vázquez-Iniesta, Kaliappan Andi, S. Siva Kumar, Diane Ordway, Rafael Prados-Rosales, Paul A. MacAry, R. Andres Floto

**Affiliations:** 1 Molecular Immunity Unit, University of Cambridge Department of Medicine, MRC-Laboratory of Molecular Biology, Cambridge, UK; 2 Victor Philip Dahdaleh Heart & Lung Research Institute, University of Cambridge, Cambridge, UK; 3 Department of Microbiology, The Yong Loo Lin School of Medicine, National University of Singapore, Singapore, Singapore; 4 Cambridge Centre for Lung Infection, Royal Papworth Hospital, Cambridge, UK; 5 Cambridge Centre for AI in Medicine, University of Cambridge, Cambridge, UK; 6 Infectious Disease Group, Genome Institute of Singapore, Singapore, Singapore; 7 SingHealth Duke-NUS Institute of Precision Medicine, SingHealth Duke-NUS Genomic, Medicine Centre, Cardiovascular and Metabolic Disorder Program, Duke-NUS Medical, School, Singapore, Singapore; 8 Mycobacteria Research Laboratories, Department of Microbiology, Immunology and Pathology, Colorado State University, Fort Collins, CO, USA; 9 London School of Hygiene and Tropical Medicine, London, UK; 10 Institute for Bio-computation and Physics of Complex Systems BIFI, Department of Theoretical Physics, University of Zaragoza, Zaragoza, Spain; 11 ICMR- National Institute for Research in Tuberculosis, Chennai, India; 12 Department of Preventive Medicine, Public Health and Microbiology, School of Medicine, Universidad Autónoma de Madrid, Madrid, Spain

## Abstract

Genetic determinants of susceptibility to *Mycobacterium tuberculosis* (*Mtb*) remain poorly understood but could provide insights into critical pathways involved in infection, informing host-directed therapies and enabling risk stratification at individual and population levels. Through a genome-wide forward genetic screen, we identify Toll-like receptor 8 (TLR8) as a key regulator of intracellular killing of *Mtb*. Pharmacological TLR8 activation enhances the killing of phylogenetically diverse clinical isolates of drug-susceptible and multidrug-resistant *Mtb* by macrophages and during *in vivo* infection in mice. TLR8 is activated by phagosomal mycobacterial RNA released by extracellular membrane vesicles and enhances xenophagy-dependent *Mtb* killing. We find that the TLR8 variant M1V, common in Far Eastern populations, enhances intracellular killing of *Mtb* through preferential signal-dependent trafficking to phagosomes. TLR8 signaling may, therefore, both regulate susceptibility to tuberculosis and provide novel drug targets.

## Graphical abstract

**Graphical abstract:**
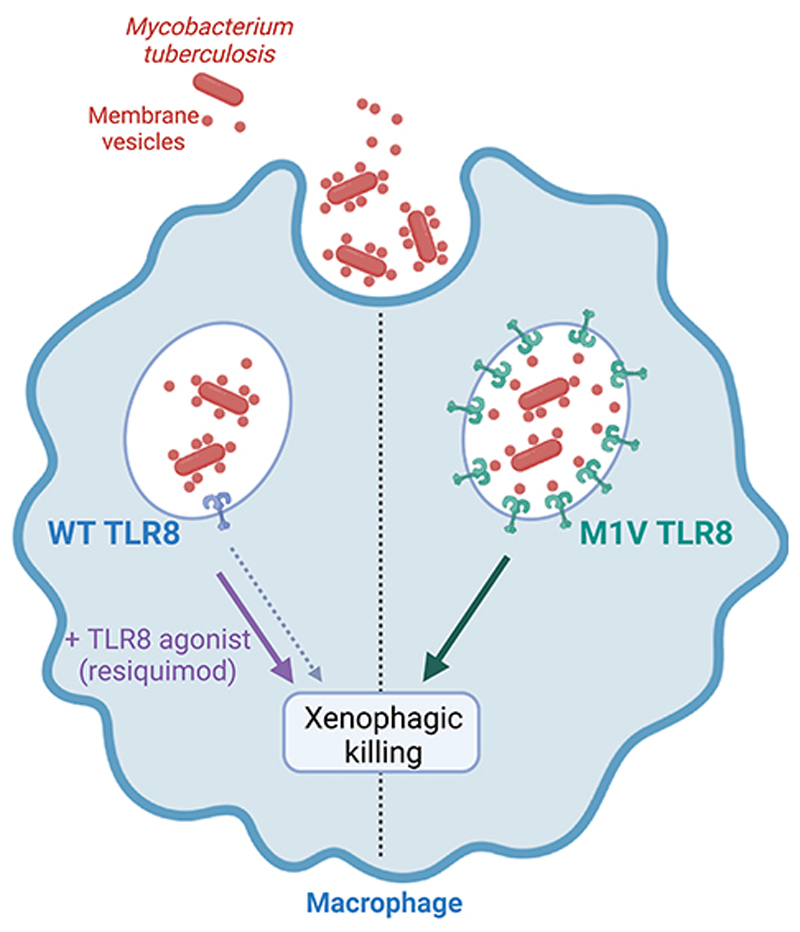

## Introduction

Tuberculosis (TB), a disease caused by mycobacteria of the *Mycobacterium tuberculosis* (*Mtb*) complex (MTBC), remains a major global threat to human health, with an estimated third of the world’s population at one time exposed,^1^ over 1.3 million deaths recorded per year,^2^ and growing rates seen for multidrug-resistant (MDR) and extensively drug-resistant (XDR)^3,4^ infections. Increasing antibiotic resistance and long treatment durations have motivated the search for druggable innate immune pathways that could be pharmacologically stimulated to deliver hostdirected therapy.^5,6^

The factors that control cell-autonomous immunity to *Mtb*, however, remain partially understood,^1,6^ despite forward genetic screens mostly using other mycobacterial species in macrophage or zebrafish infection models,^7–9^ genome-wide association studies in several ethnic populations,^10–13^ and genetic analyses of primary immunodeficiencies associated with mycobacterial susceptibility.^14^

Since the initial interactions of *Mtb* with macrophages appear critical in determining the outcome of human infection,^6^ we set out to discover new druggable pathways in macrophages that control the intracellular survival of *Mtb*.

## Results

### Forward genetic screening identifies TLR8 as a potential therapeutic target in *Mtb* infection

To identify novel host restriction factors, we exposed THP1 macrophages, transduced with a genome-wide CRISPR library,^15^ to a GFP-expressing auxotrophic *Mtb* strain with preserved virulence.^16^ We then selected cells with excess *Mtb*-associated fluorescence at 24 h post-infection (using fluorescence-activated cell sorting [FACS]), amplified and sequenced guide RNA (gRNA) templates from extracted DNA, and then detected targeted genes that were statistically over-represented in the sorted compared to the bulk populations (Figure S1).

**Figure 1:**
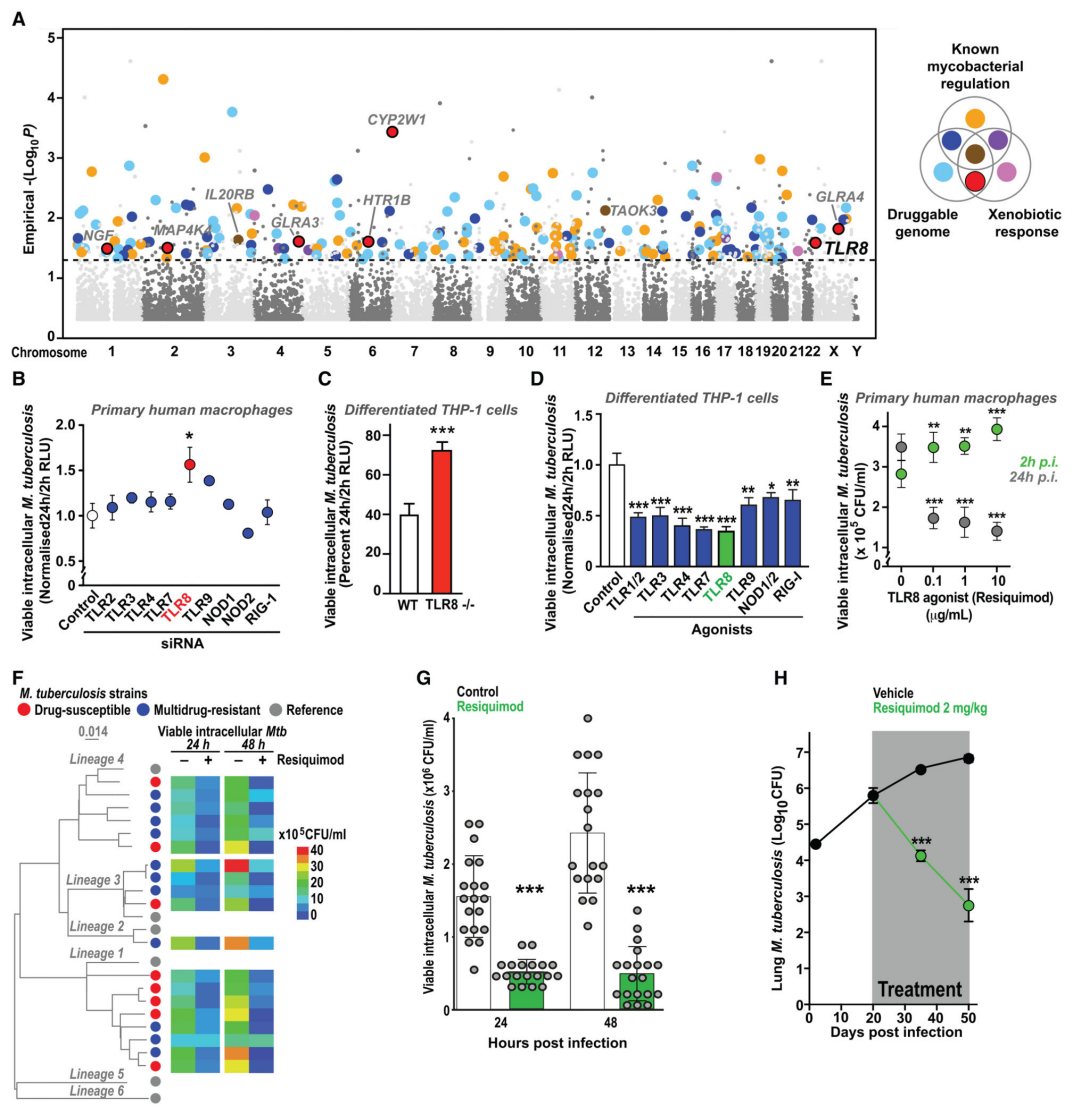
Forward genetic screening reveals TLR8 as a druggable pathway in *M. tuberculosis* infection (A) Genome-wide CRISPR screen in THP1 macrophages infected with GFP-expressing *M. tuberculosis* (*Mtb*) Δ*leu*D Δ*pan*CD (BleuPan) performed on three separate occasions. Cells with high *Mtb*-associated fluorescence at 24 h were fluorescence-activated cell-sorted and their gRNA representation compared to the bulk infected population at a gene level. A Manhattan plot of significant hits is shown, color annotated based on whether genes are known regulators of mycobacterial infection, involved in the xenobiotic response, part of the druggable genome, or combinations of these. Novel druggable genes involved in the xenobiotic response (red) include TLR8. (B) Effect on intracellular killing of luminescent *Mtb* (H37Rv) by primary human macrophages following knockdown (using pooled siRNA) of a panel of pattern recognition receptors (PRRs). (C) Intracellular killing of luminescent *Mtb* (Δ*leu*D Δ*pan*CD [BleuPan]) by wild-type and TLR8-knockout THP1 macrophages. (D) Effect of agonists targeting different PRRs (blue), including resiquimod targeting TLR8 (green), on intracellular killing of luminescent *Mtb* (H37Rv) by THP1 macrophages. (E) Effect of resiquimod (at a range of concentrations) on intracellular killing of *Mtb* (Δ*leu*D Δ*pan*CD [BleuPan]) by primary human macrophages from healthy volunteers. (B–E) Data (mean ± SEM) shown from representative experiments at least three independent repeats, performed in at least triplicate (using primary macrophages, B and E, from at least three different healthy volunteers) (*p < 0.05, **p < 0.01, and ***p < 0.001; Student’s t test). (F and G) Resiquimod improves intracellular killing of clinical isolates of *Mtb*. THP1 macrophages were infected with a phylogenetically diverse collection of drug-susceptible (red) or multidrug-resistant (blue) *Mtb* clinical isolates and co-treated with resiquimod (10 μg/mL) or vehicle alone for 24 or 48 h, and viable intracellular mycobacteria were enumerated through cell-associated colony-forming units (CFUs/mL). Experiments were performed in at least triplicate. (F) Maximum likelihood phylogenetic tree of all isolates tested constructed using RAxML, generated by mapping detected variable positions to *Mtb H37Rv* strain. Representatives from the main six *Mtb* lineages (gray) are included for genomic context. Scale bar indicates the number of substitutions per variable site. (G) Viable intracellular *Mtb* (mean ± SD) recovered from THP1 macrophages infected with each of the clinical isolates in (F) at 24 and 48 h post-infection in the presence of resiquimod (green) or vehicle control (white). ***p < 0.001 (paired Student’s t test). (H) Resiquimod treatment (via once-daily intraperitoneal injection) of C57BL/6 mice infected with multidrug-resistant *Mtb* (TB5904) resulted in a significant reduction in lung bacterial counts. Data represents mean ± SEM CFUs from 5 mice per time point in each group. ***p < 0.001 (Student’s t test).

Our screen identified many plausible hits across a range of cellular processes, including multiple genes known to influence *Mtb* growth within macrophages^17–25^ (Figures 1A and S1; Tables S1, S2, S3, and S4). We selected novel hits within the druggable genome^26^ involved in the xenobiotic response for further analysis. We focused on Toll-like receptor 8 (TLR8), a known endosomal sensor of single-stranded RNA (ssRNA) and established mediator of antiviral immunity^27^ that has previously been implicated in host defense against mycobacteria, including *Mtb*.^28–30^ We compared the relative contribution of a panel of pattern recognition receptors (PRRs) to *Mtb* infection of primary human macrophages using small interfering RNA (siRNA) knockdown (as done previously^31^) and found that TLR8 silencing led to the greatest increase in viable intracellular bacteria (Figure 1B), results supported by our findings of increased intracellular survival of *Mtb* in CRISPR-mediated TLR8-knockout THP1 macrophages (Figures 1C and S2).

We next examined the impact of pharmacological stimulation of TLR8 and other PRRs and found that the intracellular killing of *Mtb* by THP1 macrophages was most enhanced by treatment with the TLR8 agonist resiquimod^32^ (Figure 1D), which also increased mycobacterial uptake (Figure 1E) but had no direct effect on *Mtb* in liquid culture or cell viability (Figure S2). Together, our results indicate that TLR8 plays a critical role in the macrophage killing of *Mtb* and that this pathway is sub-maximally activated under baseline conditions, thus identifying TLR8 as a potential target for host-directed therapy.

Resiquimod is an imidazoquinoline with antiviral and antitumor activity in preclinical animal models^33,34^ and clinical activity as an adjuvant to vaccines^35^ and cancer immunotherapy^36^ and is currently licensed by the European Medicines Agency for the treatment of cutaneous T cell lymphomas.^37,38^ Resiquimod can activate both human intracellular receptors for ssRNA, TLR8 and TLR7.^39^ Since TLR7 is not expressed in human macrophages^40^ (Figure S3), resiquimod activity is abolished in *Mtb*-infected human *TLR8*^−/−^ macrophages (Figure S2) but preserved in *TLR7*^−/−^ cells (Figure S3). In contrast, TLR8 in mice is a pseudogene influencing TLR7 expression.^41^ Murine macrophages, therefore, only express TLR7, which is thus orthologous to human TLR8. As expected, the activity of resiquimod on *Mtb*-infected mouse bone marrow-derived macrophages is mediated exclusively by this receptor (Figure S2).

Resiquimod treatment of THP1 macrophages infected with a phylogenetically diverse collection of drug-susceptible and MDR *Mtb* (MDR-TB) clinical isolates resulted in profound reductions in viable intracellular bacteria at 24 and 48 h post-infection (Figures 1F, 1G, and S4). Resiquimod treatment of mice infected via aerosol with MDR-TB isolates led to almost a three log-fold reduction in lung colony-forming units and reduced inflammatory lung damage (Figures 1H and 1I)—a greater effect than that seen with imiquimod (when both were compared in separate experiments) (Figure S5)—suggesting a potential role for resiquimod and related compounds as host-directed therapy for TB.

### TLR8 detects phagosomal RNA released by *Mtb* through extracellular membrane vesicles

**Figure 2:**
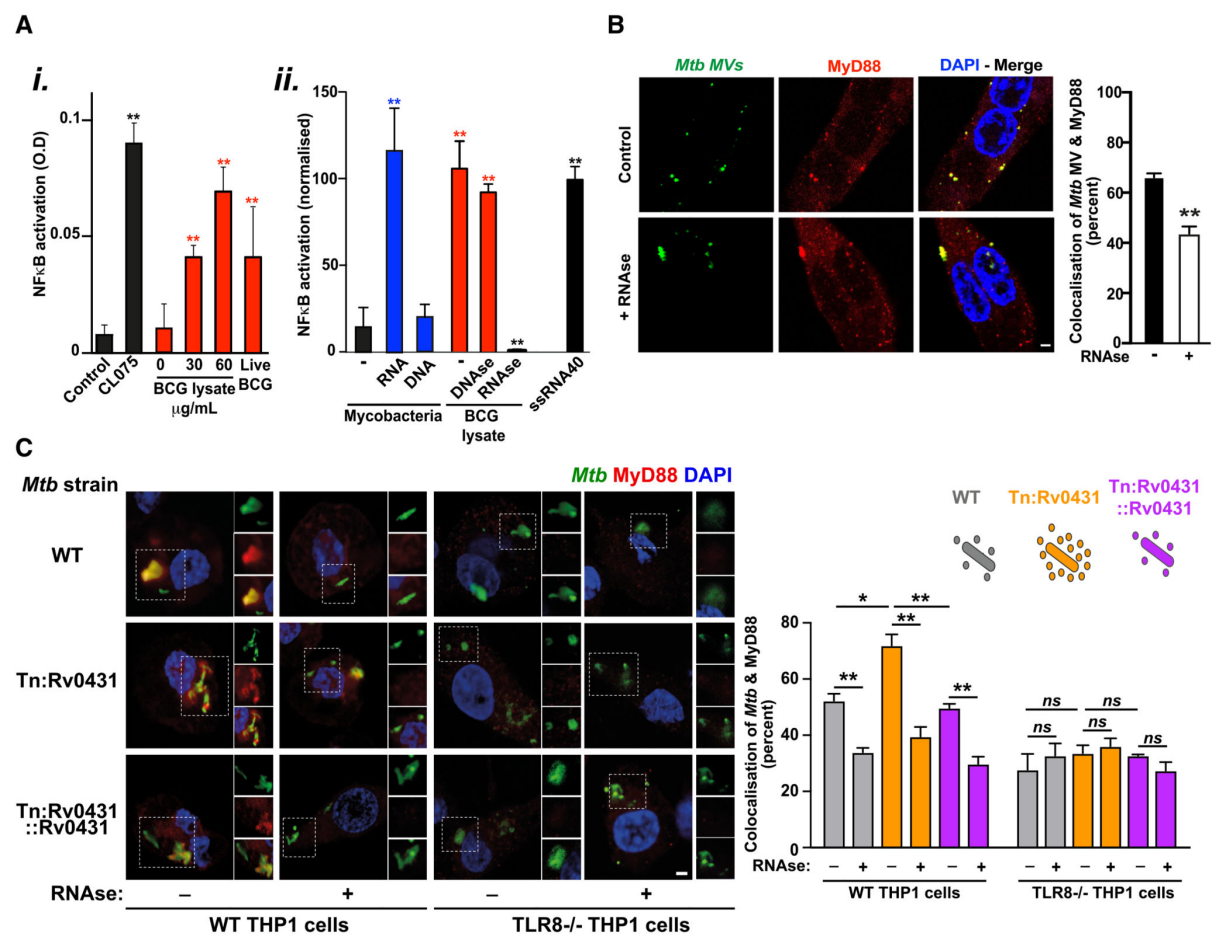
TLR8 senses phagosomal RNA released from *M. tuberculosis* in extracellular membrane vesicles (A) TLR8-2 chimeric receptors (made by fusing the TLR8 extracellular domain to the transmembrane and cytosolic domains of TLR2) were stably surface expressed in HEK293T cells containing a nuclear factor κB (NF-κB) luciferase reporter. TLR8-dependent NF-κB signaling was assessed following (i) addition of *M. bovis* BCG lysates or live bacteria (red) or the TLR8 ligand CL075 (black) or (ii) mycobacterial RNA or DNA (blue), *M. bovis* BCG lysates (untreated or pre-treated with DNase or RNase, red), or ssRNA40 (black). Data (mean ± SEM) are representative of at least three independent experiments performed in at least triplicate. *p < 0.05 and **p < 0.01 (Student’s t test). (B) Extracellular membrane vesicles isolated from *Mtb* H37Rv were CFSE labeled (green; *Mtb* MVs) and incubated with THP-1 macrophages for 24 h either with or without RNase A (100 μg/mL), immunostained for MyD88 (red), and imaged (and co-localization quantified) using confocal microscopy. (C) THP-1 macrophages were infected with CFSE-labeled *Mtb* H37Rv; the transposon mutant *Tn:rv0431* (ΔvirR), which releases increased numbers of membrane vesicles; or the complemented mutant *Tn:rv0431+rv0431* (Δ*virR*::*virR*) in the presence or absence of RNase A for 24 h, immunostained for MyD88 (red), and imaged (and co-localization quantified) using confocal microscopy. (B and C) Image scale bar: 2 μm. Data (mean ± SEM) are representative of at least three independent experiments preformed in at least triplicate (with a minimum of 50 *Mtb* phagosomes evaluated per replicate). *p < 0.05 and **p < 0.01 (Student’s t test).

We next explored the mechanism of action of TLR8 during *Mtb* infection. Using a surface-expressed TLR8-TLR2 chimeric receptor^42^ stably transfected in HEK293 cells, we showed that TLR8 can be activated by a wide range of slow- and rapid-growing mycobacterial species (Figure S6) and by purified mycobacterial RNA (but not DNA) and *M. bovis* BCG lysates and can be attenuated by RNase pre-treatment (Figure 2A). During THP1 macrophage infection, TLR8 activation within *Mtb*-containing phagosomes (monitored by recruitment of MyD88) was inhibited by co-incubation with RNase (Figure S6), indicating phagosomal sensing of mycobacteria-derived RNA.

Since *Mtb* is known to produce RNA-containing extracellular membrane vesicles (MVs),^43^ particularly in the context of limiting iron availability^44^ (as occurs in the phagosome^45^), we wondered whether MVs might trigger TLR8 signaling. We confirmed that *Mtb* (H37Rv and Δ*leu*D Δ*pan*CD [BleuPan]) and *M. bovis* BCG produced similar quantities and distributions of MVs (Figure S6) containing RNA-encoding proteins involved in a range of functions (Figure S6). We obtained several lines of evidence in support of MVs triggering TLR8 signaling. We found that exposure of THP1 macrophages to isolated *Mtb* MVs at input ratios (MVs:cells) of 10:1 (considerably less than observed by electron microscopy during *in vitro* or *in vivo Mtb* infection^43^) stimulated MyD88 recruitment to endosomal compartments, which was attenuated by co-treatment with RNase (Figure 2B). An *Mtb* mutant that over-produces MVs, *Tn:Rv0431* (ΔvirR)^46,47^ (Figure S6), was able to enhance TLR8 activation (in an RNase-inhibitable manner) during the infection of wild-type (WT), but not *TLR8*^−/−^, THP1 macrophages (Figure 2C). Thus, the previously observed reduced intracellular survival of *Tn:Rv0431* within macrophages^47^ is likely to be mediated through TLR8 activation by RNA-containing MVs.

### TLR8 promotes intracellular mycobacteria clearance via xenophagy

**Figure 3:**
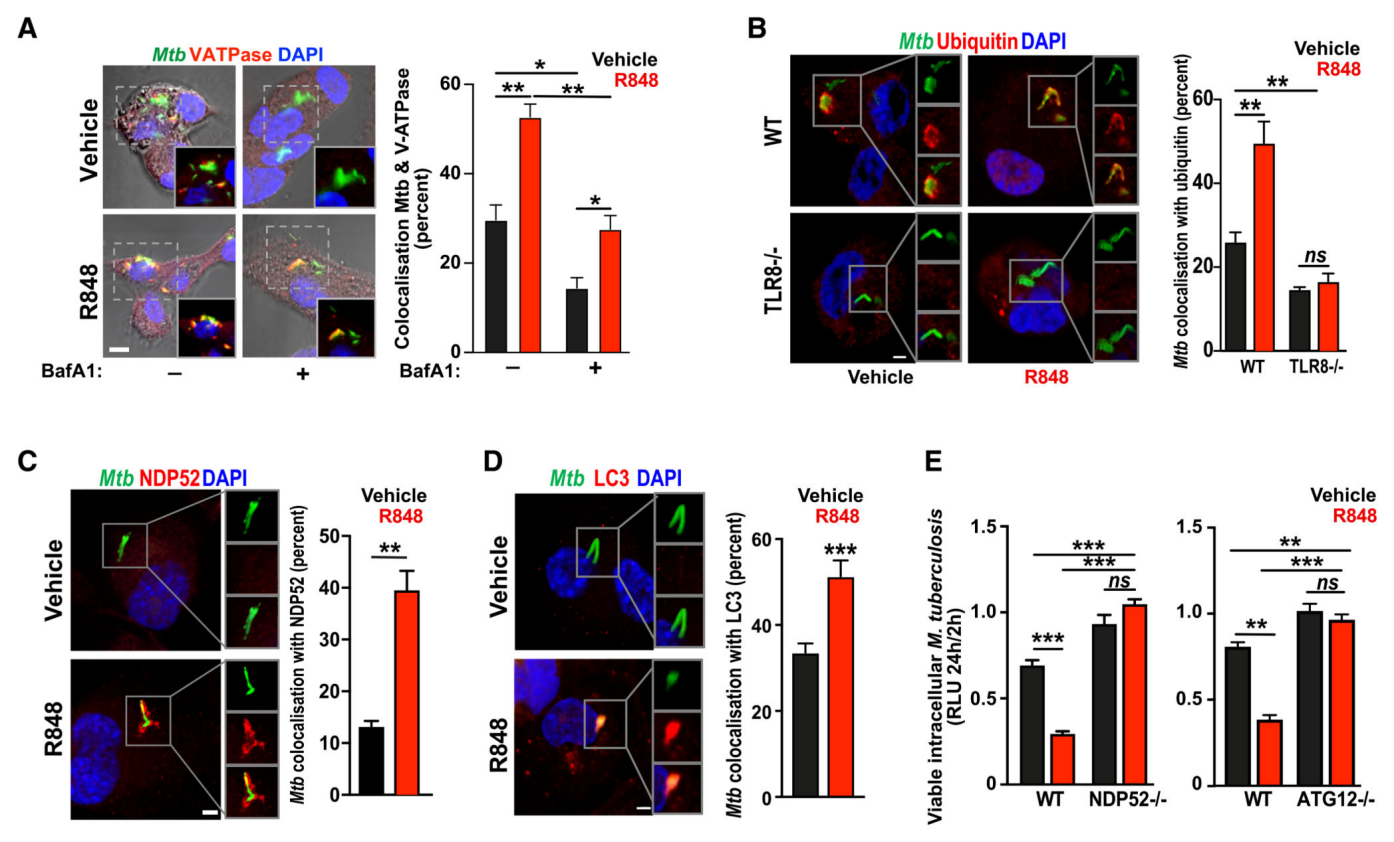
TLR8 enhances intracellular killing of mycobacteria through xenophagy (A) TLR8 activation using resiquimod increases fusion of *Mtb*-containing phagosomes with lysosomes. THP-1 macrophages infected with GFP-labeled *Mtb* H37Rv (green) in the presence of bafilomycin A1 (BafA1) and/or resiquimod (or vehicle controls) for 2 h were immunostained for V-ATPase (red) and imaged (and co-localization quantified) using confocal microscopy (scale bar: 5 μm). Data (mean ± SEM) are representative of at least three independent experiments preformed in at least triplicate (with a minimum of 50 *Mtb* phagosomes evaluated per replicate). *p < 0.05 and **p < 0.01 (Student’s t test). (B) Wild-type (WT) or TLR8-knockout (*TLR8*^−/−^) THP-1 macrophages were infected with GFP-labeled *Mtb* H37Rv (green) in the presence of resiquimod or vehicle control for 2 h, immunostained for ubiquitin (red), and imaged (and co-localization quantified) using confocal microscopy. Images and data (mean ± SEM) are representative of experiments performed in triplicate on at least three independent occasions with a minimum of 50 cells analyzed per replicate. **p < 0.01; ns, not significant (Student’s t test). Scale bar: 2 μm. (C and D) THP-1 macrophages were infected with GFP-labeled *Mtb* H37Rv (green) in the presence of resiquimod or vehicle control for 2 h, immunostained with (C) NDP52 or (D) LC3 (red), and imaged (and co-localization quantified) using confocal microscopy. Images and data (mean ± SEM) are representative of experiments performed in triplicate on at least two independent occasions with a minimum of 50 phagosomes analyzed per replicate. **p < 0.01 and ***p < 0.001 (Student’s t test). Scale bar: 2 μm. (E) WT, ATG12-knockout (*ATG12*^−/−^), or NDP52-knockout (*NDP52*^−/−^) THP-1 macrophages were infected with luminescent *Mtb* (Δ*leu*D Δ*pan*CD [BleuPan]) in the presence of resiquimod or vehicle control. Viable intracellular *Mtb* at 2 and 24 h was quantified using luminescence (relative light unit, RLU). Data (mean ± SEM) are representative of experiments performed in triplicate on at least three independent occasions. **p < 0.01 and ***p < 0.001 (Student’s t test).

We next examined how TLR8 signaling could enhance the intracellular killing of *Mtb*. Agonist stimulation of TLR8 led to enhanced phagosome-lysosome fusion (as monitored through *Mtb* co-localization with V-ATPase; Figure 3A) and increased numbers, acidification, and activity of lysosomes (Figure S7). Since lysosomal biogenesis and autophagy are known to be regulated by transcription factor EB (TFEB),^48,49^ we examined whether TLR8 stimulation might activate these processes. As expected, resiquimod led to the rapid nuclear localization of TFEB in primary human macrophages and in a reconstituted heterologous expression system (Figure S7).

We then explored the role of autophagy in TLR8 effector functions and observed agonist-triggered increases in the number and acidification of autophagosomes in bone marrow-derived macrophages from mRFP-GFP LC3 transgenic mice^50^ (Figure S7); agonist-induced ubiquitination of *Mtb*-containing phagosomes in WT, but not *TLR8*^−/−^ THP1 macrophages (Figures 3B and S7); agonist-stimulated recruitment of the autophagy adaptor NDP52 (Figure 3C) and of LC3 (Figure 3D) to *Mtb*-containing phagosomes; and inhibition of agonist-enhanced intracellular killing of *Mtb* in *NDP52*^−/−^ and *ATG12*^−/−^ THP1 macrophages (Figures 3E and S7). Our findings, therefore, indicate that TLR8 activation increases lysosomal activity and stimulates xenophagic clearance of intracellular *Mtb* (and not simply epiphenomenal activation of autophagy). Since we have implicated phagosomal ubiquitination and a dependence on NDP52, we conclude that TLR8 activates conventional autophagy rather than LC3-associated phagocytosis.

### The M1V variant of TLR8 modifies intracellular receptor localization and boosts *Mtb* clearance

**Figure 4:**
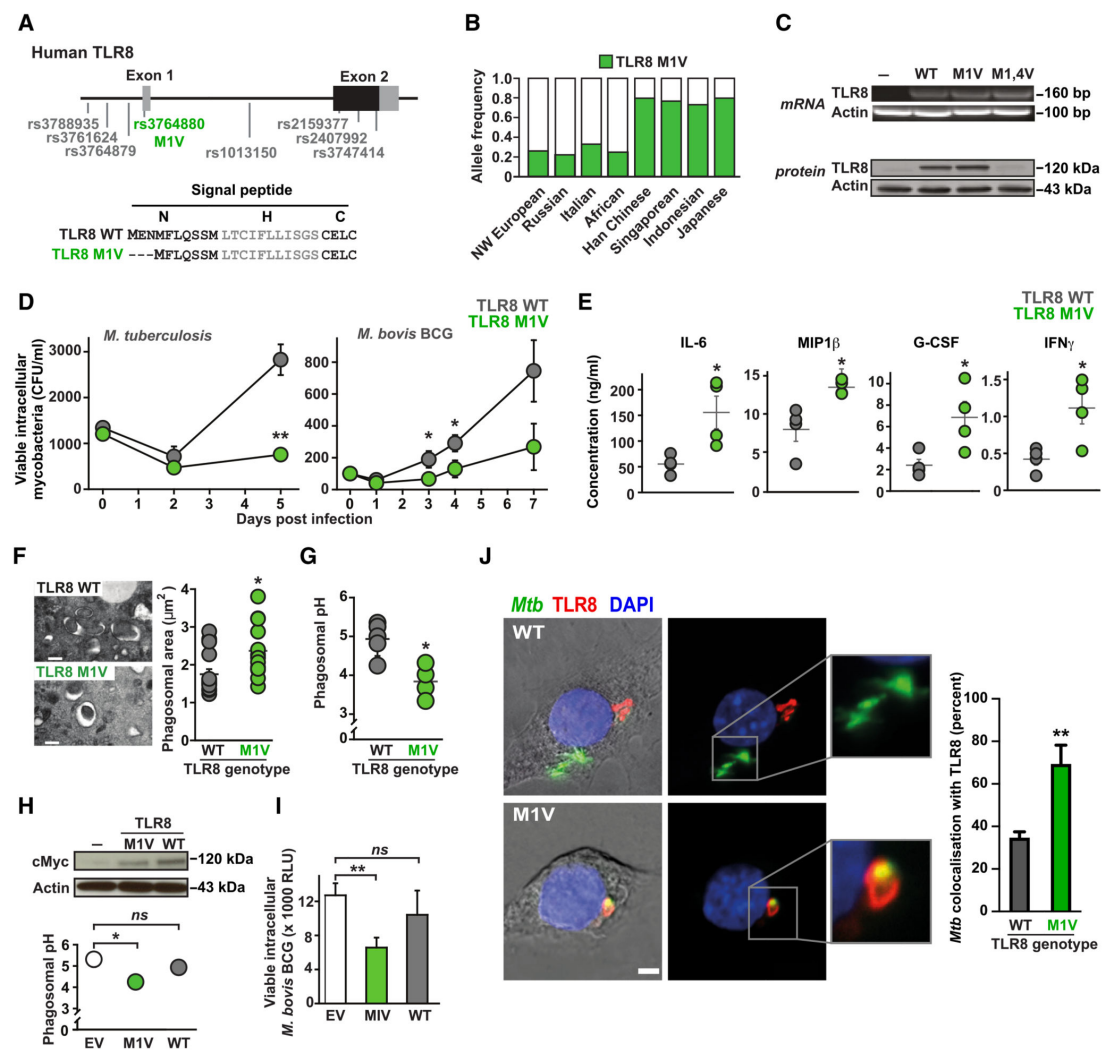
The M1V variant of TLR8 alters intracellular receptor localization and enhances intracellular killing of *M. tuberculosis* (A) Top: haplotype block of single-nucleotide polymorphisms (SNPs) in human TLR8 associated with protection from pulmonary tuberculosis^43^ with one non-synonymous coding polymorphism (rs3764880 M1V, green). Bottom: predicted signal peptide sequences of WT and M1V TLR8 suggest that the M1V polymorphism leads to alternative start codon usage (methionine at position 4). (B) Allele frequency of TLR8 (green) in different ethnic groups (data from NCBI SNP database and Davila et al.^41^). (C) RT-PCR (top) and western blot (bottom) analysis of ancestral TLR8 (WT), the M1V variant (M1V), and a mutant TLR8 with both methionines (at positions 1 and 4) changed to valines (M1,4V). (D–F) Primary human macrophages from healthy volunteers that are homozygous or hemizygous for the ancestral TLR8 (TLR8 WT, gray) or M1V variant (TLR8 M1V, green) (*n* = 5 for each genotype) were infected with either *Mtb* CDC1551 or *M. bovis* BCG. (D) Viable intracellular mycobacteria were enumerated by counting CFUs in cell lysates at indicated time points post-infection. (E) Secreted cytokines were measured in supernatants at 24 h post-infection. (F) Mycobacteria-containing phagosomes (by electron microscopy) within primary macrophages from M1V homo/hemizygotes were larger, indicating the probable formation of bactericidal phago-lysosomes. At least 150 phagosomes per donor were evaluated (with 5 donors per genotype) in 3 independent experiments. Data (mean ± SEM) are representative of experiments performed in at least triplicate. *p < 0.05, **p < 0.01, and ***p < 0.001 (Student’s t test). (G) Primary macrophages from M1V homo/hemizygotes were able to better acidify mycobacteria-containing phagosomes than macrophages from ancestral controls, measured by assessing fluorescent ratios of internalized heat-killed *Mtb* H37Rv labeled with both acid-quenchable (FITC) and pH-resistant (Alexa 633) fluorophores by flow cytometry. Data (mean ± SEM) are representative of experiments performed in at least triplicate using samples from *n* = 5 subjects for each genotype. *p < 0.05 (Student’s t test). (H and I) Primary human macrophages from healthy volunteers homo/hemizygous for ancestral TLR8 were transfected with either Myc-tagged TLR8 WT or M1V (or empty vector). Similar exogenous TLR8 expression was confirmed by western blot analysis using a c-Myc specific antibody. Macrophages transfected with the M1V variant demonstrated (H) greater acidification of mycobacteria-containing phagosomes and (I) improved killing of intracellular mycobacteria. Data (mean ± SEM) are representative of experiments performed in at least triplicate on three independent occasions (each using a separate donor). *p < 0.05 and **p < 0.01 (Student’s t test). (J) Mouse macrophage cells (RAW 264.7) transfected with either ancestral (WT, top) or M1V (M1V, bottom) human TLR8 tagged with c-Myc were infected with GFP-expressing *Mtb* (Δ*leu*D Δ*pan*CD [BleuPan]), immunostained, and imaged (and co-localization quantified) using confocal microscopy (TLR8: red, *Mtb*: green). Images and data (mean ± SEM) are representative of experiments performed in triplicate on at least three independent occasions with a minimum of 50 cells analyzed per replicate. **p < 0.01 (Student’s t test). Scale bar: 2 μm.

Given the profound effects of TLR8 activation on *in vitro* and *in vivo Mtb* infections, we wondered whether naturally occurring genetic polymorphisms in *TLR8* might influence host susceptibility to *Mtb* infection through altered receptor signaling. We focused on the M1V variant (rs3764880) that leads to an alternative start codon usage and, consequently, an altered signal peptide (Figures 4A–4C). The M1V variant has been implicated in significant protection from pulmonary TB in our previous population genetic studies^41^ and is found at variable allele frequencies in different ethnic groups, most abundantly in Far Eastern populations^51^ (Figure 4B). Primary macrophages from healthy volunteers homozygous or hemizygous for the M1V TLR8 variant demonstrated enhanced killing of *Mtb* and *M. bovis* BCG (Figure 4D), increased inflammatory cytokine release (Figures 4E and S8), and more mature mycobacteria-containing phagosomes (as evidenced by their increased size [Figure 4F] and greater acidification [Figure 4G]) compared to ancestral (WT) TLR8 controls. The transduction of M1V, but not WT, TLR8 into primary macrophages from WT hemizygous or homozygous individuals significantly increased phagosomal acidification (Figure 4H) and enhanced intracellular mycobacterial killing (Figure 4I), supporting a direct effect of the M1V variant on host restriction of *Mtb*.

Since the intracellular localization of the related receptor TLR7 is controlled by N-terminal determinants,^52^ we wondered whether the M1V polymorphism might favorably alter TLR8 trafficking within cells. Compared to the ancestral WT receptor, we found that the M1V variant showed improved co-localization with *Mtb*-containing phagosomes when expressed in RAW-264.7 mouse macrophages (Figure 4J) and has altered intracellular localization when heterologously expressed in HEK273 cells (Figure S8), which is dependent on its signal peptide, as WT and M1V TLR8-CD4 chimeric receptors are also differentially localized within cells (Figure S8). While the mechanism for this process remains unclear, changes to the signal peptide have been implicated in altered intracellular localization of other receptors (such as MC3R^53^).

## Discussion

In summary, we have identified TLR8 as an important mediator of cell-autonomous immunity against *Mtb* that acts by sensing mycobacterial RNA within macrophage phagosomes and stimulating xenophagic clearance. Our data suggest that TLR8 detects RNA-containing *Mtb* MVs released in response to iron starvation experienced within phagosomes,^44^ a mechanism that may explain the observed impact of TLR8 on macrophage responses to other bacteria.^54–56^ It is reasonable to imagine that TLR8 activation may sense viable bacteria (as previously suggested^57^) that are actively producing RNA-containing MVs since these are likely to be continuously and rapidly cleared or destroyed during *in vivo* infection.

We show that the TLR8 pathway is a potential therapeutic target during *Mtb* infection since it is sub-maximally activated physiologically and, when stimulated pharmacologically by resiquimod, enhances clearance of drug-susceptible and MDR-TB *in vitro* and *in vivo*. In addition to direct effects on macrophage clearance, TLR8 agonists may also improve adaptive immune responses during *Mtb* infection *in vivo* since they are recognized vaccine adjuvants.^35^ Importantly, resiquimod, as a licensed drug, could be rapidly repurposed and clinically evaluated as a host-directed therapy for *Mtb*.

Finally, we demonstrate that the M1V variant that alters the signal peptide of TLR8 is preferentially trafficked to *Mtb*-containing phagosomes and promotes greater intracellular mycobacterial killing, potentially explaining its genetic association with protection from pulmonary TB^41^ and its likely evolutionary selection and raising the possibility that polymorphisms in other genes may, singly or in combination, influence host susceptibility by regulating macrophage clearance of *Mtb*.

### Limitations of this study

This study has a number of limitations that should be considered. Genome-wide CRISPR screen libraries, while powerful, are not fully efficient, which may lead to false negative hits, potentially missing critical genes involved in *Mtb* host-pathogen interactions. *In vitro* analysis of macrophage infection with *Mtb* may be influenced by culture growth conditions, which may not fully replicate the complex environment within the human host. Additionally, reliance on mouse models for TB research presents limitations, as mice do not form granulomas in the same way humans do. Moreover, the involvement of (and impact of resiquimod on) adaptive immunity, which modulates and enhances macrophage responses, may additionally contribute to the observed *in vivo* phenotypes.

## Resource Availability

### Lead contact

Requests for further information and resources should be directed to and will be fulfilled by the lead contact, Prof. R. Andres Floto (arf27@cam.ac.uk).

### Materials availability

Plasmids generated in this study can be available upon request to the lead contact and the generation of a materials transfer agreement (MTA).

## Star✶Methods

### Key Resources Table

**Table:** 

REAGENT or RESOURCE	SOURCE	IDENTIFIER	
Antibodies			
Goat Polyclonal anti-Mouse Alexa Fluor 488	Thermo Fisher Scientific	A11029; RRID: AB_2534088	
Goat Polyclonal anti-Mouse Alexa Fluor 555	Thermo Fisher Scientific	A32727; RRID: AB_2333276	
Goat Polyclonal anti-Mouse Alexa Fluor 647	Thermo Fisher Scientific	A21236; RRID: AB_2535805	
Goat polyclonal anti-mouse HRP conjugated	Sigma-Aldrich	A2554; RRID: AB_258008	
Goat Polyclonal anti-Rabbit Alexa Fluor 488	Thermo Fisher Scientific	A11008; RRID: AB_143165	
Goat Polyclonal anti-Rabbit Alexa Fluor 555	Thermo Fisher Scientific	A21428; RRID: AB_2535849	
Goat Polyclonal anti-Rabbit Alexa Fluor 647	Thermo Fisher Scientific	A21245; RRID: AB_2535846	
Goat polyclonal anti-rabbit HRP conjugated	Sigma-Aldrich	A0545; RRID: AB_10689821	
IgG from human serum	Merck	I2511; RRID: AB_1163604	
Mouse monoclonal anti-FLAG M2	Sigma-Aldrich	F1804; RRID: AB_262044	
Mouse monoclonal anti-LC3B	Nanotools	0231-100/LC3-5F10; RRID: AB_2722733	
Mouse monoclonal anti-NDP52 [OTI4H5]	Abcam	Ab124372; RRID: AB_124372	
Mouse monoclonal anti-TFEB (M01), Clone S1	Abnova	H00007942; RRID: AB_548637	
Mouse monoclonal anti-Ubiquitin (FK2)	Sigma-Aldrich	ST1200; RRID: AB_10681625	
Rabbit monoclonal anti-c-Myc [Y69]	Abcam	Ab32072; RRID: AB_731658	
Rabbit polyclonal anti-ATP6V1A	Abcam	Ab137574; RRID: AB_2722516	
Rabbit polyclonal anti-MyD88	Abcam	Ab2064; RRID: AB_302807	
Rabbit polyclonal anti-TLR8	Sigma-Aldrich	HPA001608; RRID: AB_1080295	
Rabbit polyclonal V-ATPase A1 (H-140)	Santa Cruz	Sc-28801; RRID: AB_2258865	
Rabbit polyclonal V5-Tag	Novus Biologics	NB600-381; RRID: AB_527427	
Bacterial and virus strains			
Drug resistant *Mycobacterium tuberculosis* clinical isolates (NR006, NR007, NR009, NR004, NR010, NR008, NR021, NR024, NR029, NR033, NR041, NR045)	ICMR-NIRT, Chennai, India	N/A	
Drug susceptible *Mycobacterium tuberculosis *clinical isolates (NS001, NS007, NS006, NS008, NS009, NS0013, NS016, NS017, NS046, NS043, NS058, NS092, NC096, NS055, NS057)	ICMR-NIRT, Chennai, India	N/A	
*Escherichia coli* Stbl3 competent cells	Thermo Fisher Scientific	C737303	
*Mycobacterium bovis* BCG	ATCC	35733, TMC 1010 [BCG Danish]	
*Mycobacterium bovis* BCG-lux	Floto lab	(57)	
*Mycobacterium bovis* BCG-lux-GFP	Floto lab	(57)	
*Mycobacterium chelonae*	National University of Singapore	ATCC#TMC 1544 [Friedmann]	
*Mycobacterium fortuitum*	National University of Singapore	ATCC#[TMC 1529]	
*Mycobacterium marinum*	National University of Singapore	ATCC#[TMC 1218]	
*Mycobacterium scrofulaceum*	National University of Singapore	ATCC# L2238 [1356, NCTC 10803, TMC 1323]	
*Mycobacterium tuberculosis* H37Rv	ATCC	TMC 102 [H37Rv]	
*Mycobacterium tuberculosis* H37Rv-GFP	Dr S. Newton, London, UK	N/A	
*Mycobacterium tuberculosis* M10	Dr Chan, Seoul, Korea	N/A	
*Mycobacterium tuberculosis* TN5904	Dr B.N. Kreiswirth, Newark, NJ, USA	N/A	
*Mycobacterium tuberculosis* VirR- (Tn:rv0431)	Dr Rafael Prados-Rosales, Madrid, Spain	N/A	
*Mycobacterium tuberculosis* VirR-::WT (Tn:rv0431::rv0431)	Dr Rafael Prados-Rosales, Madrid, Spain	N/A	
*Mycobacterium tuberculosis* ΔleuD ΔpanCD (Bleupan)	Dr W. Jacobs III, NY, USA	N/A	
*Mycobacterium tuberculosis* ΔleuD ΔpanCD-GFP	Dr. Lalita Ramakrishnan, Cambridge, UK	N/A	
*Mycobacterium tuberculosis* ΔleuD ΔpanCD-lux-GFP	This paper	N/A	
*Mycobacterium tuberculosis* ΔleuD ΔpanCD-mCherry	Dr. Lalita Ramakrishnan, Cambridge, UK	N/A	
Biological samples			
Bone marrow derived macrophages	Dr Caetano Reis e Sousa, London, UK	N/A	
Bone marrow derived macrophages from mRFP-GFP-LC3 transgenic C57BL/6 mice	Prof. David Rubinstein, Cambridge, UK	N/A	
PBMCs − monocyte-derived macrophages	This paper	N/A	
Chemicals, peptides, and recombinant proteins			
Albumin-Dextrose-Catalase	Sigma-Aldrich	M0553-1VL	
Blasticidin	Thermo Fisher Scientific	R21001	
Calcium pantothenate	Sigma Aldrich	C8731-25G	
CD14^+^	Miltenyi Biotec	130-050-201	
CFSE	BioLegend	423801	
CL075	Invivogen	tlrl-c75	
cOmplete™, Mini, EDTA-free Protease Inhibitor Cocktail	Roche	11836170001	
DMEM	Sigma-Aldrich	D6429	
Dual-Luciferase Assay system	Promega	E1910	
ECL Advance western blotting detection	Sigma-Aldrich	RPN2106	
FCS	PanBiotech	P30-3702	
Ficoll-Hypaque	Amersham	17-5442-02	
Formaldehyde	Sigma-Aldrich	F8775-25ML	
G418/neomycin	Thermo Fisher Scientific	10131035	
Geneporter® 2 transfection reagent	AMSBio	AMS.T202015	
Glutamine	Sigma-Aldrich	G8540	
Glutaraldehyde	Sigma-Aldrich	340855	
Glycerol	Fisher	10795711	
Glycine	Sigma-Aldrich	G7126	
HEPES	Lonza	CC-5022	
HiPerFect transfection reagent	Qiagen	301705	
Human M-CSF	Peprotech	300-25-100UG	
Hygromycin B	Cambridge Bioscience	H011-20mL	
Imiquimod	Invivogen	tlrl-imq (5 mg)	
Kanamycin	Merck Millipore	420411-5GM	
L-Leucine	Sigma-Aldrich	L-8000	
Lipofectamine LTX	Thermo Fisher Scientific	A12621	
Lipofectamine® 3000 Transfection Reagent	Thermo Fisher Scientific	L3000008	
Live Cell Imaging solution	Thermo Fisher Scientific	A59688DJ	
LysoSensor Green DND-189	Thermo Fisher Scientific	L7535	
LysoTracker red DND-99	Thermo Fisher Scientific	L7528	
Middlebrook 7H11 agar	Sigma-Aldrich	M0428-500G	
Middlebrook 7H9 broth	Sigma-Aldrich	M0178-500	
Murine MCS-F	Peprotech	315-02 (100μg)	
OADC	Fisher	12674697	
Optiprep	Sigma-Aldrich	D1556	
Osmium tetroxide	Fisher Scientific	31253.01	
PBS	Sigma-Aldrich	P4474-1L	
Penicillin/Streptomycin	Sigma-Aldrich	P0781	
Phosphatase inhibitors	Sigma-Aldrich	4906845001	
PMA	Sigma-Aldrich	P1585	
Prolong Gold antifade mountant	Thermo Fisher Scientific	P36962	
Puromycin	Thermo Fisher Scientific	A1113803	
Resiquimod	Invivogen	tlrl-r848-5	
RIPA buffer	Thermo Fisher Scientific	R0278	
RNase A	New England BioLabs	T3018L	
Roche DNase I	Sigma-Aldrich	#10104159001	
RPMI	Sigma-Aldrich	R8758-500ML	
ssRNA40	Invivogen	tlrl-lrna40	
Triton X-100	Sigma-Aldrich	T9284	
Tween 20	Sigma-Aldrich	P9416-50ML	
Tween 80	Sigma-Aldrich	P4780-100ML	
Zeocin	Thermo Fisher Scientific	R25001	
Zymosan	Invivogen	tlrl-zyn	
β-Mercaptoethanol	Gibco	21985023	
Critical commercial assays			
AgencourtRNAClean XP beads	Beckman Coulter	a63987	
Agilent High Sensitivity DNA kit	Agilent Technologies	5067-4626	
Amaxa Cell Line Nucleofector Kit V	Lonza	VCA-1003	
Bio-Plex 17-plex (IL-1β, IL-2, IL-4, IL-5, IL-6, IL-7, IL-8, IL-10, IL-12 (p70), IL-13, IL-17, G-CSF, GM-CSF, IFN-γ, MCP-1 (MCAF), MIP-1β and TNF-α) cytokine assay kit	Merck Millipore	HCYTOMAG-60K	
DNeasy Blood and Tissue kit	Qiagen	69504	
Endotoxin Free Maxi kit	Qiagen	12362	
High Pure RNA isolation kit	Roche	11828665001	
Hiseq Rapid SBS kit V2 50 cycles	Illumina	FC-402-4022	
Magic Red Cathepsin B kit	BioRad	ICT938	
PCR cleanup kit	Qiagen	28104	
Pierce BCA Protein Assay Kits	Thermo Fisher Scientific	20-2,000 μg/mL	
QIAampFFPE purification kit	Qiagen	56404	
Qubit dsDNA HS DNA Kit	Thermo Fisher Scientific	Q32851	
Qubit RNA HS Assay Kit	Thermo Fisher Scientific	Q32852	
QuikChange® II XL	Stratagene	200522	
SuperScript-II Reverse Transcriptase	Thermo Fisher Scientific	18064014	
TruSeq small RNA library	Illumina	RS-200-0012	
Deposited data			
Raw and analyzed CRISPR data	This paper	EBI-ENA accession number: PRJEB62758	
RNA Seq Raw data	This paper	Gene Expression Omnibus number: GSE288494	
Experimental models: Cell lines			
FLAG-tagged TFEB reporter HeLa cells	Prof. David Rubinstein, Cambridge, UK	N/A	
HEK293T cells	ATCC	CRL-3216	
HeLa cells	ATCC	CCL-2	
Raw 264.7 cells	ATCC	TIB-71	
THP-1 BLUE NFkB cells	Invivogen	thp-nfkbv2	
THP-1 cells	ATCC	TIB-202	
THP1 AP-1-Luc2 cells	ATCC	(RRID:CVCL_A4CA)	
TLR8-expressing Raw 264.7 cells	This paper	N/A	
TLR8-TFEB expressing HeLa cells	This paper	N/A	
Experimental models: Organisms/strains			
C57BL/6 mice	The Jackson Laboratories	#000664	
Oligonucleotides			
See Table S5	N/A	N/A	
Recombinant DNA			
Human Toronto Knockout Library	Addgene	#90294	
LentiCRISPRv2	Addgene	#52961	
pcDNA3.1-*c*-myc-TLR8	This paper	N/A	
pcDNA3.1-V5-TLR8	This paper	N/A	
pcDNA3.1-TLR8-M1V	This paper	N/A	
pcDNA™3.1/V5-His-TLR8/TLR2	This paper	N/A	
pcDNA™3.1/V5-His	Thermo Fisher Scientific	V81020	
pCMV-VSV-G	Addgene	#8454	
pGL4 luciferase reporter vector	Promega	#TM259	
psPAX2	Addgene	#12260	
pSpCas9 (BB)-2A-Puro (PX459)	Addgene	#48139	
Software and algorithms			
CirGO	N/A	(67)	
CRISPR screen code	This paper	Zenodo: https://doi.org/10.5281/zenodo.14982932	
FASTQC software	N/A	version 0.11.9	
FlowJo	N/A	version 10.10.0	
GraphPad Prism	N/A	version 10.2.3 (347), April 21, 2024	
HGNC	N/A	https://www.genenames.org	
ImageJ software	N/A	Version 2.9.0/1.53t	
NIS Elements AR analysis software	N/A	Version 4.00.07	
Panther tool	N/A	http://www.pantherdb.org	
RAxML	N/A	version 8.2.8	
Trim Galore	N/A	version 0.6.4_dev	
Zen software	N/A	version 2010	
ZetaView Software	N/A	version 8.05.12 SP1	

## Experimental Model And Study Participant Details

### Mycobacteria

The following strains of mycobacteria were used: *Mycobacterium tuberculosis H37Rv, M. tuberculosis H37Rv-GFP; M. tuberculosis VirR*- (*Tn:rv0431*) and its complemented strain *M. tuberculosis VirR-::WT* (*Tn:rv0431::rv0431*),^47^ *M. bovis BCG*, BCG-lux (a luminescent reporter strain of *M. bovis BCG* encoding the Vibrio *lux AB* gen*e* or GFP^58^), clinical isolates of *M. scrofulaceum, M. marinum, M. chelonae, M. fortuitum*, multi-drug resistant (MDR) isolates of *M. tuberculosis* (TN5904 and M10); have also been used some clinical isolates of *M. tuberculosis* from the Bacteriology Division of the ICMR-National Institute for Research in Tuberculosis (Chennai, India) that were either drug-susceptible and multidrug-resistant. Isolates were grown as previously described^31,59^ in Middlebrook 7H9 broth containing 0.5% glycerol, 0.05% Tween 80 and 10% albumin–dextrose–catalase enrichment.

### *Auxotrophic* M. tuberculosis

*M. tuberculosis ΔleuD ΔpanCD* (Bleupan) double auxotroph strain^60^ (gift from Dr Bill Jacobs) was transduced with *pSMT12-mCherry* or *pSMT12-GFP* (gifts from Dr Lalita Ramakrishnan, Cambridge, UK) or *pSMT1-LuxAB-GFP*, and grown in Middlebrook 7H9 broth containing 0.5% glycerol, 0.05% Tween 80 and 10% oleic acid–albumin–dextrose–catalase enrichment (OADC), 0.05 mg/mL L-leucine, 0.024 mg/mL calcium pantothenate. When necessary, 50 μg/mL hygromycin B, 40 μg/mL kanamycin or 50 μg/mL zeocin were added to cultures. Bacteria were grown for 15 days at 37°C, then transferred in bigger culture volume (1/100 dilution) for 10 more days in media of the same composition.

### Mycobacterial homogenates

For generation of mycobacteria homogenates, mycobacterial cultures were harvested, washed, and resuspended in phosphate-buffered saline (PBS). Bacteria were disrupted by bead-beating in a bullet blender (Next Advance) for 5 min and homogenates were briefly centrifuged to remove the beads and intact cells. Experiments to identify the mycobacterial ligand for TLR8 were performed on *M. bovis* BCG homogenates either heat denatured at 95°C for 5min; or subjected to enzymatic digestion by RNase A or DNase I for 15 min at room temperature. 1 unit of RNase or DNase was used for every 1μg of *M. bovis* BCG homogenate. RNA and DNA from *M bovis* BCG were obtained from cultures grown to mid-log phase using the Roche High Pure RNA Isolation kit and the DNeasy Blood and Tissue Kit respectively, according to manufacturers’ instructions.

### Single cell bacterial suspensions

To prepare single cell suspensions of bacteria prior to infection, bacteria were centrifuged 24 h prior to experiment and resuspended in bacterial growth media without tween to allow one generation time and complete reformation of mycobacterial cell wall. On the day of infection, mycobacterial cultures were passed through a 27-gauge needle 10 to 12 times prior to injecting through a 5- μm filter to achieve close to single cell suspensions of bacteria.

### Colony forming units

To enumerate colony forming units (CFU) counts, bacterial suspensions were plated on Middlebrook 7H11 agar with 10% OADC enrichment supplement and CFU were counted after 21 days of incubation at 37°C.

### Subjects details

Healthy consented individuals were genotyped at the TLR8 locus and recalled, stratified by genotype to provide peripheral blood samples (described below). Samples from at least 5 individuals homozygous or hemizygous for ancestral (WT) TLR8 and at least 5 individuals homozygous or hemizygous for M1V TLR8 were compared in functional experiments. Informed by pilot experiments, samples size calculations, based on observed standard deviation of 10% in macrophage intracellular killing and cytokine production, indicated that recruitment of *n* = 4 subjects in each arm would provide an 80% power to detect a 20% difference in responses between genotypes (alpha 0.05).

### Mammalian cell cultures

#### Monocyte-derived macrophages

Peripheral blood mononuclear cells (PBMCs) were generated as previously described.^31^ Briefly, PBMCs were isolated from peripheral blood obtained from healthy consented subjects (approved by Regional NHS Research Ethics Committee), stratified by TLR8 WT or M1V genotype, by Ficoll-Hypaque density separation. CD14^+^ positive selection using magnetic beads was used to extract monocytes, which were subsequently differentiated into macrophages by stimulation with 200 ng/mL M-CSF in DMEM containing 2 mM L-glutamine, 10% FCS, 100 U/ml penicillin/streptomycin. Cells were differentiated for six days before assaying.

#### Bone-marrow-derived macrophages

Generation and culture of bone-marrow-derived macrophages (BMDM) was caried out as previous described.^61^ Femurs from 15-week-old female TLR7 knockout mice (generated as previously described^62^) or age and sex-matched C57BL/6 mouse controls were dissected to remove both ends and flushed with a 21-guage needle into serum-free DMEM. Cells were then centrifuged to remove cell culture medium and cultured in 10mL DMEM supplemented with 20% FCS, 100U/mL penicillin/streptomycin, 50μM tissueculture grade β-Mercaptoethanol and 200 nM murine MCS-F for 3 days, after which fresh medium was added. Cells were then incubated for another 3 days before the cells were scraped and seeded onto 24-well plates for experiments.

#### THP-1 macrophages

THP-1 cells, THP-1 BLUE NF-κB and AP-1 reporter monocytes were maintained in RPMI 1640, 10% FCS, 100 U/ml penicillin/streptomycin, 2 mM L-glutamine, and 200 μg/mL Zeocin when needed. Cells were supplemented with 40 ng/mL 12-phorbol 13-myristate acetate PMA for 48h to stimulate differentiation into macrophages.

#### Other cell lines

HeLa cells, FLAG-tagged TFEB reporter HeLa cells (gift from Dr David Rubinzstein, Cambridge, UK^49^), HEK 293T cells and RAW 264.7 cells were maintained in DMEM, 10% FCS, 100 U/ml penicillin/streptomycin, 2 mM L-glutamine. G418/neomycin (500 μg/mL was added to transfected cells (TLR8/TFEB-expressing HeLa, TLR8-expressing RAW 264.7).

#### *In vivo* animal study

Specific-pathogen-free female C57BL/6 mice, from 6 to 8 weeks old, were purchased from the Jackson Laboratories, Bar Harbor, Maine. Mice were maintained in the Biosafety Level III animal laboratory at Colorado State University, and were given sterile water, mouse chow, bedding, and enrichment for the duration of the experiments. The specific pathogen-free nature of the mouse colonies was demonstrated by testing sentinel animals. All experimental protocols were approved by the Animal Care and Usage Committee of Colorado State University. The CSU animal assurance welfare number is A3572-01.

## Method Details

### Extracellular membrane vesicles

#### Purification of extracellular membrane vesicles (MVs)^44^

*M. tuberculosis* H37Rv bacterial cultures were grown in 7H9 medium for 7 days, then subsequently inoculated into minimal medium and incubated at 37°C for 14 days.^43^ Bacterial cultures were sequentially filtered through 0.45-μm and 0.22-μm-pore size filters, and concentrated using an Amicon Ultrafiltration system with a 100-kDa-exclusion filter. The recovered concentrate was centrifuged to recover the vesicle pellet. The membrane vesicles were purified by density gradient ultracentrifugation using OptiPrep solution prepared in Dulbecco’s phosphate buffered saline.

#### MV RNA isolation, sequencing, and analysis

RNA was isolated from *Mtb* H37Rv MVs following and acid phenol-chlorophorm isoamyl alcohol method. The quantity and quality of the RNAs were evaluated using Qubit RNA HS Assay Kit and Agilent RNA 6000 Nano Chips (Agilent Technologies), respectively. Sequencing libraries were prepared using a TruSeq small RNA library and selecting RNA sizes from 20 to 300 nt. Briefly, starting from 300 ng of total RNA, rRNA was removed and the remaining RNA was cleaned up using AgencourtRNAClean XP beads. Purified RNA was fragmented and primed for cDNA synthesis. cDNA first strand was synthesized with SuperScript-II Reverse Transcriptase for 10 min at 25°C, 15 min at 42°C, 15 min at 70°C and pause at 4°C. cDNA second strand was synthesized with Illumina reagents at 16°C for 1 h. Then, A-tailing and adaptor ligation were performed. Libraries enrichment was achieved by PCR (30 s at 98°C; 15 cycles of 10 s at 98°C, 30 s at 60°C, 30 s at 72°C; 5 min at 72°C and pause at 4°C). Afterward, libraries were visualized on an Agilent 2100 Bioanalyzer using Agilent High Sensitivity DNA kit and quantified using Qubit dsDNA HS DNA Kit. Library sequencing was carried out on an Illumina HiSeq2500 sequencer with 50 nucleotides single end reads. Quality Control of sequenced samples was performed using FASTQC software (version 0.11.9).^63^ Adapter trimming and low-quality reads removal were performed using Trim Galore version 0.6.4_dev.^64^ Reads from each strain were mapped against the *M. tuberculosis* H37RV strain reference genome from Myco-browser (Release 4, 2021-03-23) using kallisto^65^ with single-end run mode. Functional categories of *M. tuberculosis* H37RV protein coding genes were extracted from Mycobrowser.^66^

#### Confocal microscopy

Isolated *M. tuberculosis* MVs were subsequently fluorescently labeled with carboxyfluorescein succinimidyl ester (CFSE) as previously described.^44^ prior to incubation with differentiated THP-1 cells. To assess MyD88 signaling, CFSE-labelled MVs suspension in DMEM with 10% FCS were added to differentiated THP-1 cells in the presence or absence of 100 μg/mL RNAse A and incubated at 37°C for 2 h. The cells were then washed, fixed and immunostained for MyD88.

#### Quantification of MVs by nanoparticle tracking analysis (NTA)

Nanoparticle tracking analysis (NTA) was conducted using ZetaView (*Particle Metrix*). Instrument calibration was performed prior to EV analysis using 102 nm polystyrene beads (Thermo Fisher Scientific, USA), according to manufacturer instructions. Measurements were performed using a 405 nm 68 mW laser and CMOS camera by scanning 11 cell positions and capturing 60 frames per position at 25°C with camera sensitivity 85, shutter speed 100, autofocus and automatic scattering intensity. Samples were diluted in pre-filtered PBS to approximately 10^6^-10^7^ particles⋅ml^−1^ in Millipore DI water. Analysis was performed using ZetaView Software version 8.05.12 SP1 with a minimum brightness 30, maximum brightness 255, minimum area 5, maximum area 1000, and minimum trace length 15. Triplicate videos of each sample were taken in light scatter mode. Particle size and concentration were analyzed using a built-in protocol and plotted using graph pad Prism 8.0 software.

#### Scanning electron microscopy (SEM)

Cells were fixed with 2.5% glutaraldehyde, 0.1 M sodium cacodylate, 0.2 M sucrose, 5 mM MgCl_2_(pH 7.4) and were dehydrated through a graded series of ethanol solutions before critical-point drying using liquid carbon dioxide in a Toumisis Samdri 795 device and sputter-coating with gold-palladium in a Denton Vacuum Desk-2 device. Samples were examined in a Zeiss Supra Field Emission Scanning Electron Microscope (Carl Zeiss Microscopy, LLC North America), using an accelerating voltage of 5 kV.

### THP1 CRISPR knockout library

#### Genome-wide CRISPR library

The CRISPR knockout pooled library plasmids was prepared following the protocol previously described.^15^ Briefly, the Human Toronto Knockout library (TKO V1) was amplified by transformation in Stbl3 competent cells. Colonies were scraped off plates, pooled and purified using Qiagen endotoxin free Maxi kit. Lentiviral particles were generated by transfecting HEK293T cells with the pooled CRISPR library plasmids, and used to transduce Cas-9-expressing THP-1. Cells were maintained in RPMI 1640, 10% FCS, 100 U/ml penicillin/streptomycin, 2 mM L-glutamine, 1 μg/mL Puromycin and 10 μg/mL Blasticidin. Differentiation into macrophages was achieved by treating cells with 20 ng/mL 12-phorbol 13-myristate acetate PMA for 48h prior to experiment.

After 24h of infection with fluorescent *M. tuberculosis*, THP-1 cells were detached using accutase incubation for 20 min at 37°C 5% CO_2_, spun down 300g 5min and fixed in formaldehyde 4% for 1 h. Cells were then FACS sorted to obtain the top brightest 21% of the population (together with the total population) using a Sony Biotechnology Synergy High Speed Cell Sorter. Genomic DNA was purified using Qiagen FFPE purification kit for fixed cells and amplified by PCR. PCR products were purified using Qiagen PCR clean up and reamplified using the following primers containing P5/7 adaptors as well as appropriate indexes necessary for Illumina sequencing. PCR reactions were cleaned up and remaining low molecular weight contaminants removed by AMPure XP beads purification using a ratio of 1.6:1. Purification and quantitation were validated using Agilent DNA 1000 chips and confirmed by qPCR. Sequencing was performed on Illumina Hiseq NGS using a Hiseq Rapid SBS kit V2 50 cycles (Illumina).

#### CRISPR screen analysis

Read counts were quantified using the cluster-based approach CB2 by aligning against the Toronto Knock out Library.^67^ Guide counts were normalized to counts per 1M sequencing reads for every sample. The values for the unsorted population from all three experiments were combined, choosing the highest count across experiments to represent each guide and compared to the sorted samples from each of the three independent experiments. An aggregate fold change was calculated conservatively as the minimum fold change between the unsorted population and each of the three experiments. To test for overrepresentation of guides in the sorted vs. unsorted population, a permutation test was performed. Three rounds of label permuting were conducted to accurately simulate the quantification process generating a randomized unsorted guide count distribution for each of the three experiments. Guides that had less than 0.1 counts per 1M reads in the unsorted population were removed from the analysis. As before, the aggregate log fold change was calculated as the minimum fold change across the three (permuted) experiments. For every guide, the *p*-value was then defined as the fraction of the number of guides that had a higher minimum log fold change value in the real than in the permuted dataset, and the total number of guides analyzed.

#### GO terms analysis

To visualise genes in the CRISPR screen, hits with a *p* value < 0.05 were transformed into approved symbols using HUGO Gene Nomenclature Committee (HGNC, https://www.genenames.org). The approved symbols were then entered into Panther tools software to assign Gene Ontology (GO) terms to all the hits (http://www.pantherdb.org). REVIGO enrichment analysis^68^ was used to reduce and visualise GO terms. GO terms are therefore summarised and redundancy is removed. Finally, a Python script for circular visualisation of GO terms (CirGO) was used for graphic representation.^69^

#### Network analysis

To investigate the functional connectivity of genes identified in the CRISPR screen, we constructed a network of interactions from the Pathway Commons database^70^ and performed network propagation using a random walk with restart (RWR) algorithm.^71^ RWR is designed to retain local connectivity between genes by restarting the signal diffusion process after a limited number of steps, with a fixed probability determined by a restart parameter (r).We used the implementation of RWR provided in the dnet package of the R statistical computing environment,^72^ with r = 0.2 and Laplacian normalisation of the adjacency matrix. The −log10(*p* value) for each of the 19,102 genes in the CRISPR screen were used as starting weights for propagation. To account for the fact that highly connected genes (nodes) tend to receive higher steady state scores via RWR, we performed a permutation test in which the starting weights for genes (−log10(Pvalue)) were randomised and RWR was performed a total of 30,000 times. An empirical *p* value foreach gene was then calculated as the proportion of permuted steady state scores at least as large as that observed from the CRISPR screen data. Interactions between genes with *p* values <0.05 were (*n* = 928) were used to construct a sub-network from the Pathway Commons database. From the resulting sub-network, interactions between genes in the top six largest significantly enriched GO terms identified through the REVIGO enrichment analysis were visualised.

### Individual CRISPR knockout cell lines

For individual single guide RNA (sgRNA) cloning, pairs of oligonucleotides were designed and ordered from Sigma with restriction enzyme-compatible overhangs, separately annealed and cloned into the transient CRISPR plasmid pSpCas9 (BB)-2A-Puro (PX459) as previously described.^73^ For cloning into lentiviral vector, LentiCRISPRv2 was digested with BsmBI (*Fermentas*), and the linearized vector was gel purified before ligation of annealed guide oligo pairs.^74^ The constructs were amplified in Stbl3 cells cells and plasmids were purified using endotoxin-free maxi kits. Lentiviral particles were produced by co-transfection of LentiCRISPRv2 constructs, psPAX2, and pCMV-VSV-G at a 1:2:1 ratio into HEK 293ET cells using TransIT-293 Transfection Reagent (*Mirus Bio LLC*) reagent according to manufacturer’s instructions. TLR8, TLR7, and ATG12 CRISPR knockout in THP-1 cells were generated by cloning relevant targeting guide sequences into lentiGuide-Puro vector, producing viral particles by transfection into HEK 293T cells as previously described^74^ and subsequently transducing THP-1 cells expressing lentiCas9-Blast. Cells were maintained in RPMI 1640, 10% FCS, 100 U/ml penicillin/streptomycin, 2 mM L-glutamine, 1 μg/mL Puromycin and 10 μg/mL Blasticidin. Single cell clones were expanded, sequenced to confirm gene knockout, and then pooled. To stimulate differentiation into macrophages, cells were treated with 20 ng/mL 12-phorbol 13-myristate acetate PMA for 48h prior to experiment.

### Plasmid constructs

TLR8 was cloned into a pcDNA3.1 vector (adding c-myc or V5 tags where indicated) and the TLR8 M1V variant generated by sitedirected mutagenesis using QuikChange II XL. TLR8/2 chimera constructs were generated by PCR cloning to fuse the transmembrane (TM) domain of TLR2 with the extracellular TLR8 domain using the Platinum Taq DNA Polymerase High Fidelity Master Mix (*Invitrogen*) with TLR8/2 primers. The primers used were specific for TLR8 amino acid residues 1–843. For TLR2, primers for the transmembrane domain amino acid residues 588–610 were used. The fragments were then purified, combined and used as templates for a second PCR with the fragment overlapping sequences and the respective forward and reverse primers. The resultant full-length PCR products were subsequently cloned into pcDNA3.1/V5-His.

### Transfections

HEK293T cells, HeLa cells, FLAG-tagged TFEB reporter HeLa cells^49^ were transfected using Lipofectamine 3000 Transfection Reagent and THP1 cells using Lipofectamine LTX according to manufacturer’s instructions, and assayed 48h post transfection. Primary human macrophages and RAW 264.7 macrophages were nucleofected using the Amaxa Cell Line Nucleofector Kit V and NucleofectorII Device with programs Y-010 and D-032, respectively. Prior to transfection, complete cell culture medium was removed, and cells were incubated at 37°C 5% CO_2_ in DMEM containing 10% FCS. Cells were evaluated at least 48 h post transfection, either by western blot or immunofluorescence.

### SiRNA experiments

For silencing experiments, Accell SMARTpool siRNA for Human TLR8 was obtained from Dharmacon with target sequences against CAAUUAAUAUAGAUCGUUU, CUGGGAUG UUUGGUUAUA, CUAUCAACUUGGGUAUUAA and GUCUUGACUGAAAAUGAUU. PMA-differentiated THP-1 cells were transfected with 1μM of siTLR8 according to manufacturer’s protocol, and assayed 72h post-transfection. Primary human macrophages were differentiated for three days and transfected with 1μM of either siTLR8 or other PRRs siRNApools using HiPerFect transfection reagent (*Qiagen*) for 5min, and complexes were added drop by drop onto the cells and incubated for 6hours. DMEM was added afterward and cells were kept at 37°C for 3 more days.

### Mycobacterial infections of macrophages

Infection of primary human macrophages was adapted from Schiebler et al.^31^ Primary human macrophages WT or knocked down with siTLR8 were infected with *M. tuberculosis H37Rv, M. tuberculosis* Bleupan, *M. bovis* BCG or *M. bovis* BCG-lux at a multiplicity of infection (MOI) of 5:1 for 2 h, washed in PBS and incubated at 37°C for 24 h. At indicated time points cells were washed repeatedly, lysed in ddH_2_O, serially diluted and plated onto Middlebrook 7H11 agar plates for CFU enumeration or cell-associated luminescence measurement. Infection of THP-1 macrophages with *M. tuberculosis* Bleupan, *M. bovis* BCG, or clinical isolates of drug-sensitive and drug-resistant *M. tuberculosis*, was performed at a MOI of 5:1. Infected macrophages were harvested at defined time points, lysed in ddH_2_O, serially diluted, and plated on 7H11 agar medium supplemented as described above.

For infection of THP1 cells with WT, VirR-, VirR-:WT strains, bacteria cultures were grown as previously described, harvested at mid-log phase, resuspended in PBS and fluorescently labeled with Carboxyfluorescein succinimidyl ester (CFSE) kit for 30 min at 37°C. Bacterial suspensions were then washed twice in PBS with centrifugation steps (3000 g for 10 min) to remove supernatants, and bacterial pellets were finally resuspended in DMEM, 10% FCS prior to macrophage infection. Infection was carried out as described above and cells were incubated either with or without 200 μg/mL RNAse A for 2 h prior to fixing and immunostaining for MyD88.

### Cell death analysis

#### LDH release assay

To assess cell viability, 200uL of cell supernants were harvested after 24h of infection of different cell types (Human Primary macrophages and THP-1 cells) with *M. tuberculosis* Bleupan at MOI 10:1. A CyQUANT LDH Cytotoxicity assay was then immediately performed following manufacturer’s instruction. In details, supernatants were incubated with CyQUANT substrate mix for 30 min at room temperature and protected from light. Reaction was then stopped by addition of the stop solution volume to volume with substrate mix. Absorbance was measure at both 490nm (sample signal) and 680nm (background signal from the instrument). Maximum release was measured by lysis cells using 10x lysis buffer. Data are presented as the percentage of maximum release on 3 independent experiments each ran in triplicate.

#### Live or dye staining

An orthogonal way to assess cell viability was to measure cell positively stained with Live or Dye Fixable Staining Viability Kit using flow cytometry. Basically, 24 h following infection with a range of *M. tuberculosis* Bleupan MOIs, WT and TLR8 KO THP-1 cells were washed once with PBS without Ca^2+^ nor Mg^2+^, and detached using Accutase incubation for 15min. Accutase was inactivated by addition of FCS-containing media, cells were then harvested, washed again once with PBS and incubated at room temperature for 30min protected from light with Live or Dye at a concentration of 1uL of dye per million cells per mL. Cells were washed again once with PBS and kept at 4C in BD CellFIX solution until analysis by FACS using a BD Fortessa (analysis was run within the next 48 h). These experiments have been done in triplicate, and for each experiment every condition was assess in triplicate.

#### Confocal imaging

Immunofluorescence experiments were undertaken as previously described.^31^ Cells were seeded on glass coverslips in 24-well tissue culture plates prior to infection with either *M. tuberculosis* H37Rv or *M. tuberculosis ΔleuD ΔpanCD* (Bleupan) double auxotroph expressing either GFP- or mCherry. Following incubation at various time points, cells were washed with PBS, fixed wih 4% parafor-maldehyde (PFA) in PBS for at least 30 min and permeabilized for 5 min with 0.1% Triton X-100 prior to immunostaining. Primary antibodies (against MyD88, V-ATPase, Ubiquitin, NDP52, LC3, V5 or Myc) were diluted to recommended concentrations in staining medium (DMEM, 10% FCS, 10 mM Glycine, 10 mM HEPES pH 7.4) to which IgG (1:00) was added and the cells were incubated at room temperature for 2 h. Cells were subsequently washed twice in staining medium. Cells were incubated with secondary antibodies (Alexa Fluor 555 and 647 (*Invitrogen*)) for 30 min, protected from light. The cells were subsequently washed, and the coverslips were dipped in water prior to mounting on slides using ProLong Gold Antifade Mountant with DAPI. Slides were left to dry overnight, protected from light. Images were acquired either on a Zeiss LSM780 or LSM880 confocal microscope (Plan-Apochromat 63x/1.40 Oil immersion lens) and analyzed with Zen 2010 software, Zeiss LSM Image Browser (*Carl Zeiss*), or NIS Elements AR analysis (*Nikon*) software and ImageJ.

For analysis of lysosomal number and acidification in THP1 macrophages, uninfected THP1 macrophages were either treated with the TLR8 agonist R848 at 10 μg/mL or left untreated for 24 h at 37°C, subsequently washed and incubated with 40 nM LysoTracker Red DND-99 and 1 μM LysoSensor Green DND-189 for 15 min. The cells were then washed twice with PBS after which Live Cell Imaging solution was added. Live confocal imaging was carried out on the Zeiss LSM 780 UV. Quantitation of lysosomes was performed using the ImageJ plugin on Fiji app. HEK 293T co-transfected with TLR8 WT-Myc tagged and TLR8 M1V-V5 tagged were fixed in methanol-acetone for immunofluorescent staining with anti-Myc and anti-V5 antibodies, and counterstained with Alexa 488 and Alexa 647 conjugated secondary antibodies. All cells were visualised using a Leica True Confocal Scanner SP5.

Bone marrow-derived macrophages were generated (as previously described^61^) from femurs of transgenic mice stably expressing mRFP-GFP-LC3^75^ (kind gift from Dr David Rubinsztein, Cambridge, UK), and were either left untreated or treated with 10ug/mL of TLR8 agonist R848 for 24hours at 37°C 5% CO_2_. Cells were then washed with PBS, and incubated in live cell imaging solution (*In-vitrogen*) prior to live confocal imaging to visualize lysosomes. Imaging was carried out using on a Zeiss LSM 780UV microscope and quantitation of lysosomes was performed using ImageJ.

### Cytokine analysis

Primary macrophages were infected with either *M. tuberculosis* ΔleuD ΔpanCD (Bleupan) double auxotroph or *M. bovis* BCG, and either left untreated or treated with R848 10 μg/ml. Cell culture supernatant was collected 24 h post infection and analyzed using Bio-Plex 17-plex (IL-1β, IL-2, IL-4, IL-5, IL-6, IL-7, IL-8, IL-10, IL-12 (p70), IL-13, IL-17, G-CSF, GM-CSF, IFN-γ, MCP-1 (MCAF), MIP-1β and TNF-α) cytokine assay kit (*Biorad*) according to manufacturer’s instructions.

### Activation assays to determine TLR ligand

#### Dual luciferase assay

HEK293T cells were co-transfected with TLR8/2 and the pGL4 luciferase reporter vectors (*Promega*) using Geneporter 2 (*Genlantis*) transfection reagent according to manufacturers’ protocol. HEK293T cells expressing the TLR8/2 and the pGL4 luciferase reporter vectors were treated with various ligands 24h post-transfection. Cells were lysed in passive lysis buffer and lysates were analyzed for luciferase activity using the Dual-Luciferase Assay system (*Promega*). The resultant TLR8/2 chimera generated to promote stable surface expression of TLR8 in HEK293T cells were treated with either whole or lysed mycobacteria, TLR8 ligands CL075 or ssRNA40 (*InvivoGen*) or TLR2 ligand Zymosan (*InvivoGen*) to monitor NF-κB signaling by luminescence.

#### Quantification of NF-κB activation

differentiated TLR8 knockdown (or control) THP-1 BLUE cells were infected with *M. tuberculosis* H37Rv, *M. bovis* BCG, *M. marinum, M. scrofulaceum, M. fortuitum*, or *M. chelonae*, or treated with the TLR8 ligand ssRNA40. Supernatants were collected after 24hours and the levels of NF-κB-induced SEAP were quantified by colorimetric analysis, according to manufacturer’s instructions.

#### Phylogenetic analysis

Maximum likelihood phylogenetic tree of all isolates tested were constructed using RAxML (version 8.2.8), generated by mapping detected SNP positions to *M. tuberculosis H37Rv* strain. Representative strains from the main six *M. tuberculosis* lineages described by Comas et al., (2013)^76^ were included in the analysis for genomic context.

### Quantitation of lysosomal degradative capacity

Uninfected control or R848-tretated THP-1 macrophages were incubated at 37°C for 24hours, washed and incubated with Magic Red Cathepsin B Kit for 1 h according to manufacturer’s instructions, then washed twice in PBS. Cells were then resuspended in colorless live imaging solution and transferred onto 96 well plate for detection of cell-associated fluorescence on the CLARIOstar Plus Multi-mode Microplate Reader (*BMG Labtech*).

### Analysis of phagosomal pH and size

The pH of phagosomes containing MTB was assessed as previously described.^77^ Briefly, primary human macrophages from individuals that were either homo/hemizygous for the WT and M1V alleles were incubated with PFA-killed *M. tuberculosis H37Rv* double-labelled with acid quenchable (FITC) and pH-resistant (Alexa 633) for a 1h pulse and 23 h chase. The cells were analyzed by flow cytometry and intracellular calibration was performed as previously described.^77^ At least 3 independent experiments were performed, each of them on 5 donors for each genotype, assessing at least 3000 cells per donor using flow cytometry.

For electron microscopy visualization, primary macrophages were infected with *M. bovis* BCG (MOI 10:1). After 24 h of infection, macrophages were washed and fixed in 0.4% glutaraldehyde for 2 h at room temperature. Samples were then post-fixed in 1% osmium tetroxide followed by dehydration in an ascending graded series of ethanol and embedding in LR white resin. Ultrathin sections (50-70nm) were stained with 2% uranyl acetate and lead citrate and examined in a JM1010 electron microscope (*JEOL*). Phagosome area was measured using ImageJ. At least 150 phagosomes per donor were evaluated, (with 5 donors per genotype) in 3 independent experiments.

### Western blotting

At the indicated time points, cells were washed twice with PBS, and lysed using RIPA buffer containing a proteinase inhibitor cocktail and phosphatase inhibitors. Total protein content was quantified by BCA (*Thermo Scientific*) prior to loading at 20μg and resolving on 17% SDS-PAGE gels, and electro-blotting on to PVDF membranes (*Millipore*) in a wet transfer Cell (*Bio-Rad*). PVDF membranes were blocked by incubation in PBS supplemented with 5% (w/v) fat-free milk powder and 0.005% (v/v) Tween 20 for 1 h at room temperature. Membranes were washed repeatedly and incubated with primary antibodies following manufacturer recommended concentrations overnight at 4°C. Membranes were then washed and incubated for 1 h with 1:50 000 dilution of the horseradish peroxidase-(HRP) conjugated secondary antibodies: HRP (*Santa Cruz Biotechnology*). Membranes were revealed using ECL Advance Western Blotting Detection kit according to the manufacturer’s instructions.

### Mouse infection experiment

C57BL/6 mice were challenged by low-dose aerosol exposure with M. tuberculosis using a Glas-Col aerosol generator calibrated to deliver 50–100 CFU of bacteria into the lungs. Information regarding preparation of bacterial stocks and growth characteristics of the various bacterial strains (*n* = 5) used were as previously described. Strain MDR-TB M10 (resistance profile: Low-level fluoroquinolone resistance, Isoniazid, Rifampicin, Ethambutol, Streptomycin and Pyrazinamide) was originally provided by Dr. Chan, (*Seoul, Korea*). Strain MDR-TB TN5904 (resistance profile: INH (R, 1.6), EMB (S), RIF (R > 8), STR (R, 10), KAN (S) was originally provided by B. N. Kreiswirth, (Public Health Research Institute TB Center, Newark, NJ).

On Day 1 after infection, enumeration of bacteria was performed on two mice. Treatment was started from Day 20 to Day 50 after infection and consisted of the following groups: Control (saline; 0.1 mL intraperitoneal injection once daily) and R848 (2 mg/kg by 0.1 mL intraperitoneal injection once daily). On days 20, 35 and 50 following infection, bacterial loads in the lungs and spleen, lung and spleen histology, and flow cytometry were determined in 5 mice from each group. Bacterial counts were determined by plating serial dilutions of homogenates of lungs on nutrient 7H11 agar and counting colony-forming units after incubation at 37°C. All experimental protocols were approved by the Animal Care and Usage Committee of Colorado State University, and experiments were performed in accordance with NIH guidelines. To minimize bias, two groups of independent researchers performed the experiment. One group dosed the animals, whereas the second group determined bacterial burden in the different organs. A total of five animals were infected for each time point. Statistical analysis was performed by first converting CFU to logarithmic values and evaluated by a one-way ANOVA followed by a multiple comparison analysis of variance by a one-way Tukey test (SigmaStat software program). Differences were considered significant at the 95% level of confidence.

## Quantification and Stastitical Analysis

Having confirmed the normality of data, P-values for assays were determined using two-tailed Student’s t-test or ANOVA (as indicated) using GraphPad. Unless otherwise indicated, experiments were performed on at least three separate occasions with at least triplicate samples for each condition and represented as mean and standard error (SEM).

## Supplementary Material

Supplemental information can be found online at https://doi.org/10.1016/j.celrep.2025.115657.

Supplementary Material

## Data Availability

Single-cell RNA sequencing (RNA-seq) data have been deposited at GEO at GEO: GSE288494 and are publicly available as of the date of publication. CRISPR screen data have been deposited at EBI-ENA under accession number EBI-ENA: PRJEB62758. CRISPR screen code has been deposited at Zenodo : https://doi.org/10.5281/zenodo.14982932. Any additional information required to reanalyze the data reported in this paper is available from the lead contact upon request.

## Highlights

- CRISPR screening implicates TLR8 as a controller of intracellular *Mtb* replication
- TLR8 detects RNA-rich *Mtb* membrane vesicles in phagosomes, triggering xenophagy
- The TLR8 M1V variant boosts *Mtb* killing via preferential trafficking to phagosome
- An FDA-approved TLR8 agonist improves Mtb clearance, suggesting clinical utility

## In brief

Maserumule et al. show that TLR8 senses mycobacterial RNA in macrophage phagosomes, triggering xenophagy to clear *Mycobacterium tuberculosis* (*Mtb*). TLR8 polymorphisms affect tuberculosis susceptibility, and resiquimod, a TLR8 agonist, enhances clearance of drug-susceptible and multidrug-resistant *Mtb in vitro* and *in vivo*, offering the potential for therapeutic repurposing.
