# Supplementary material for: Phagosomal RNA sensing through TLR8 controls susceptibility to tuberculosis
Source: Cell Rep. Author manuscript; Available in PMC 2025 Nov 18. (PMC7618372; doi:10.1016/j.celrep.2025.115657)
Supplement: Supplementary Material [file EMS209620-supplement-Supplementary_Material.zip › 1-s2.0-S2211124725004280-mmc1.pdf]

Cell Reports, Volume *44*

Supplemental information

Phagosomal RNA sensing through TLR8 controls susceptibility to tuberculosis

Charlotte Maserumule, Charlotte Passemar, Olivia S.H. Oh, Kriztina Hegyi, Karen Brown, Aaron Weimann, Adam Dinan, Sonia Davila, Catherine Klapholz, Josephine Bryant, Deepshikha Verma, Jacob Gadwa, Shivankari Krishnananthasivam, Kridakorn Vongtongsalee, Edward Kendall, Andres Trelles, Martin L. Hibberd, Joaquín Sanz, Jorge Bertol, Lucia Vázquez-Iniesta, Kaliappan Andi, S. Siva Kumar, Diane Ordway, Rafael Prados-Rosales, Paul A. MacAry, and R. Andres Floto

Supplementary Figure 1

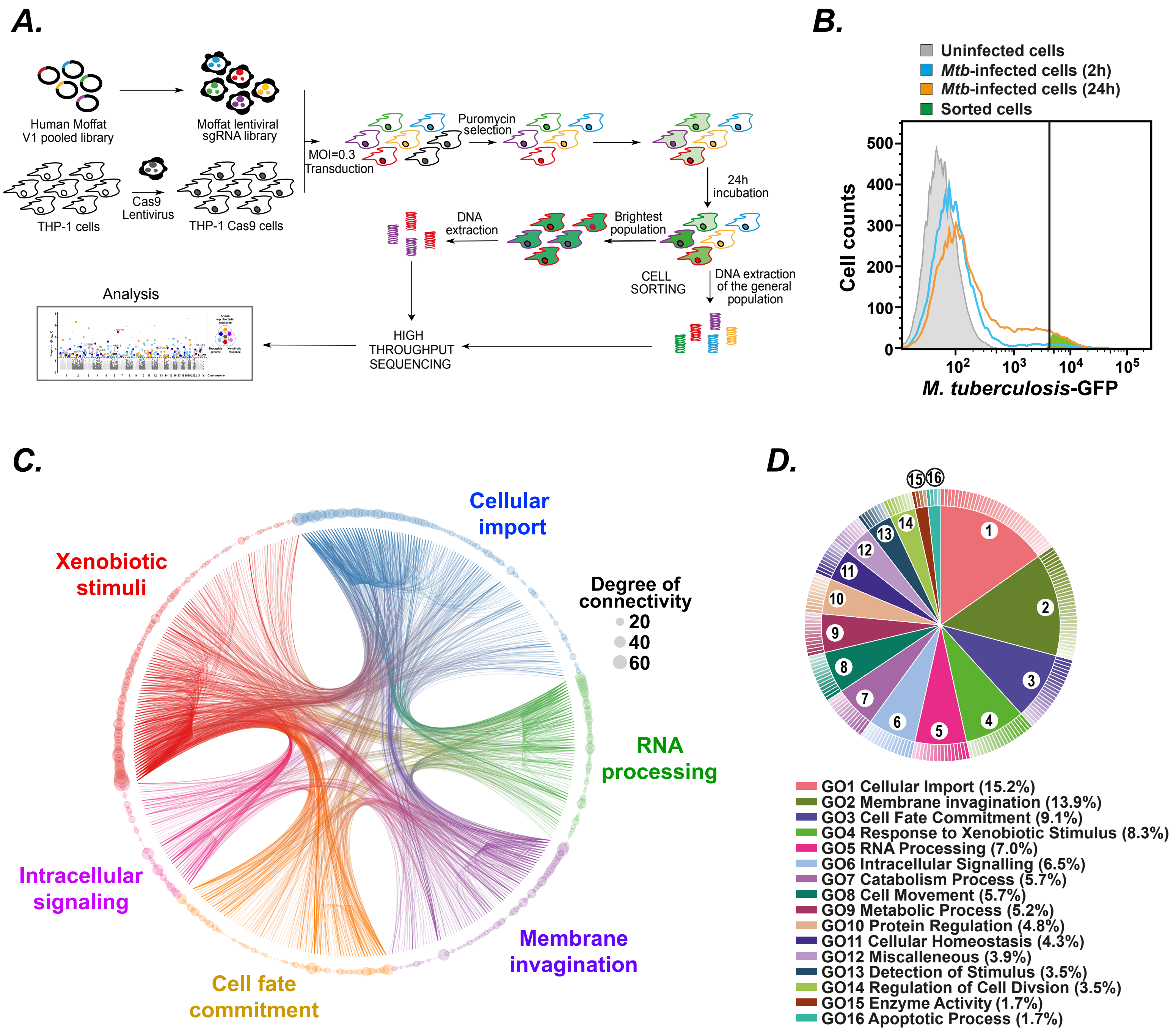

Supplementary Figure 2

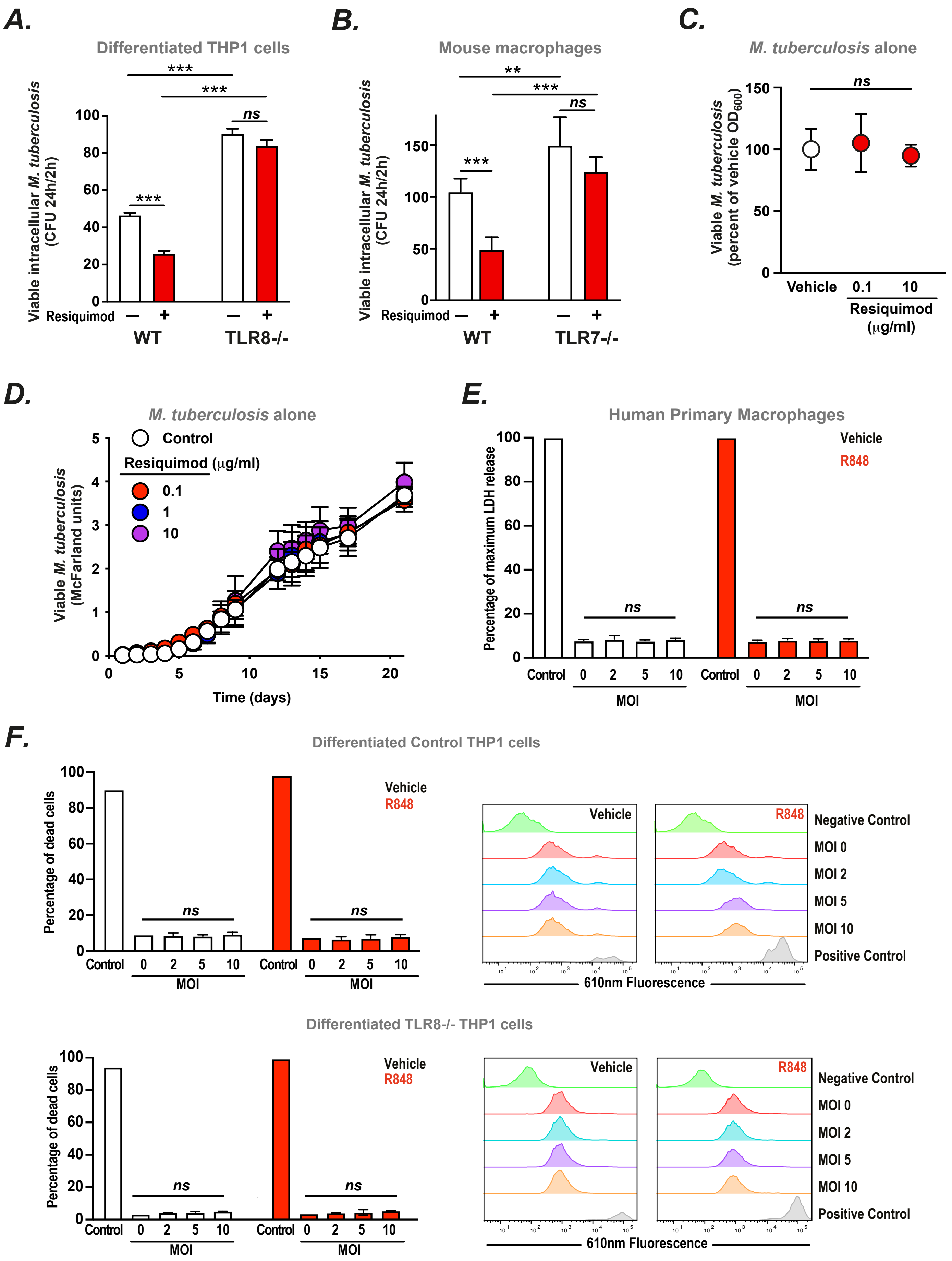

Supplementary Figure 3

A.

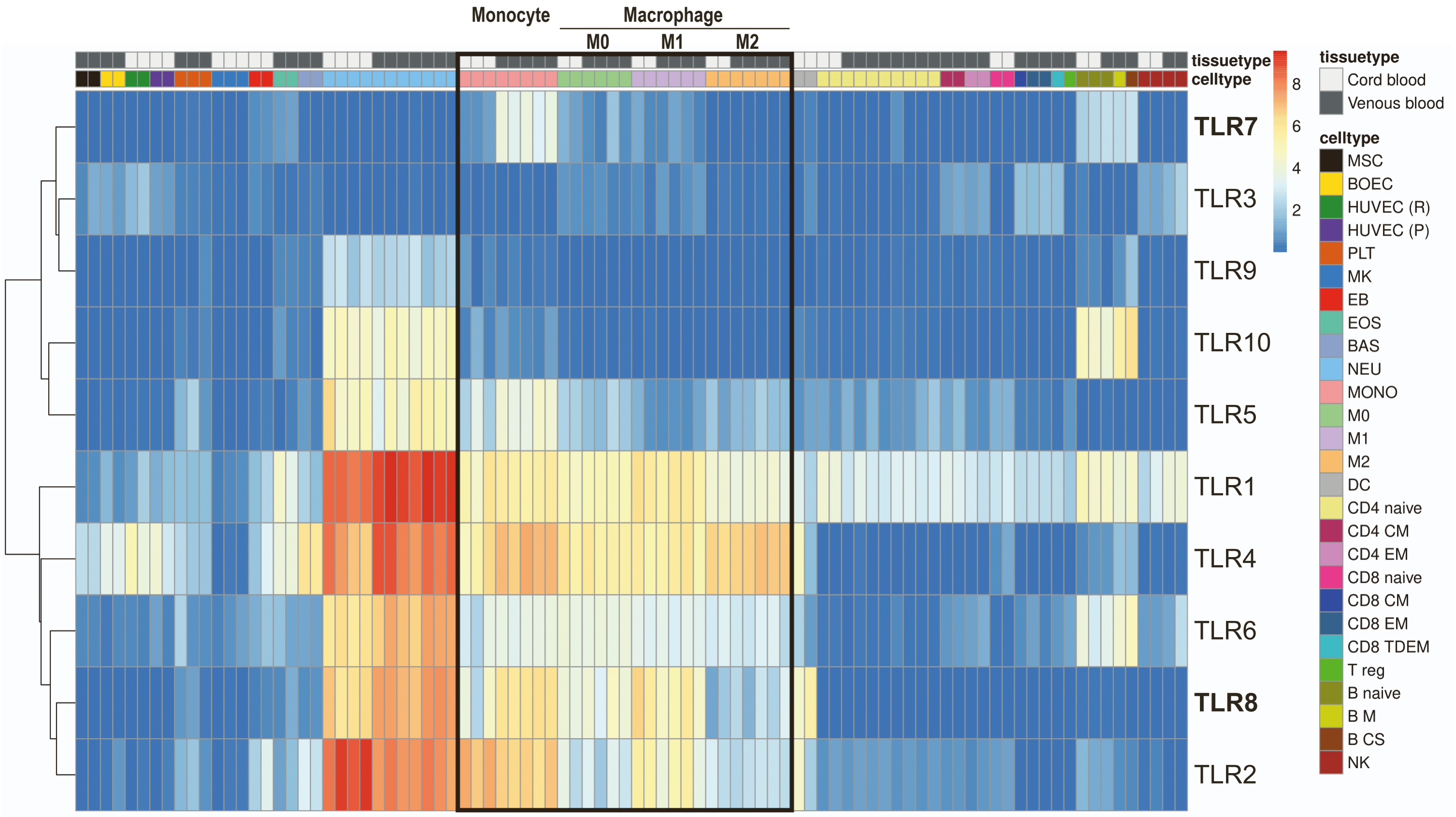

B.

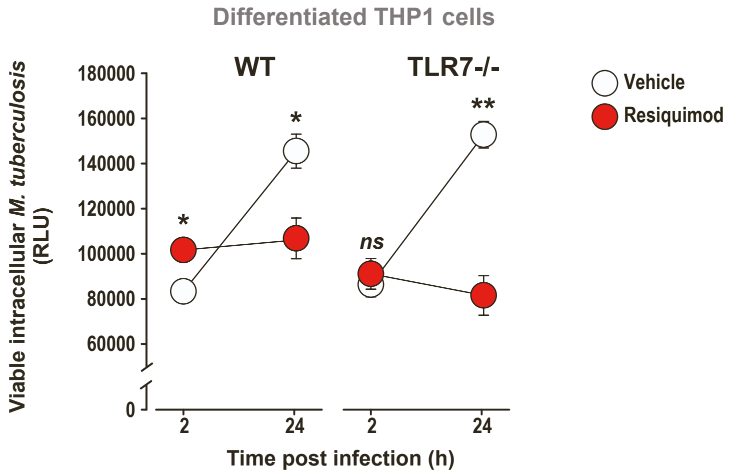

Supplementary Figure 4

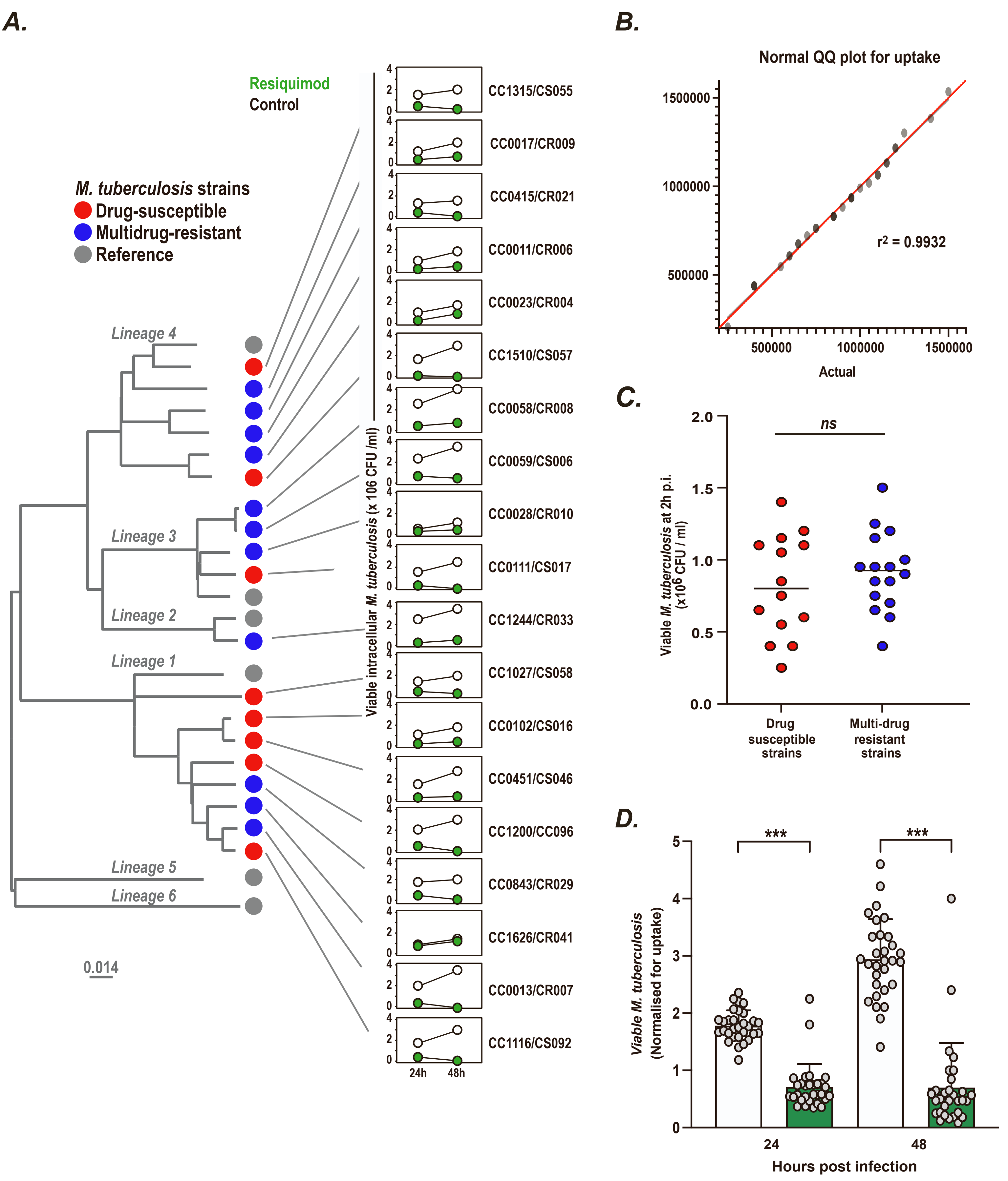

Supplementary Figure 5

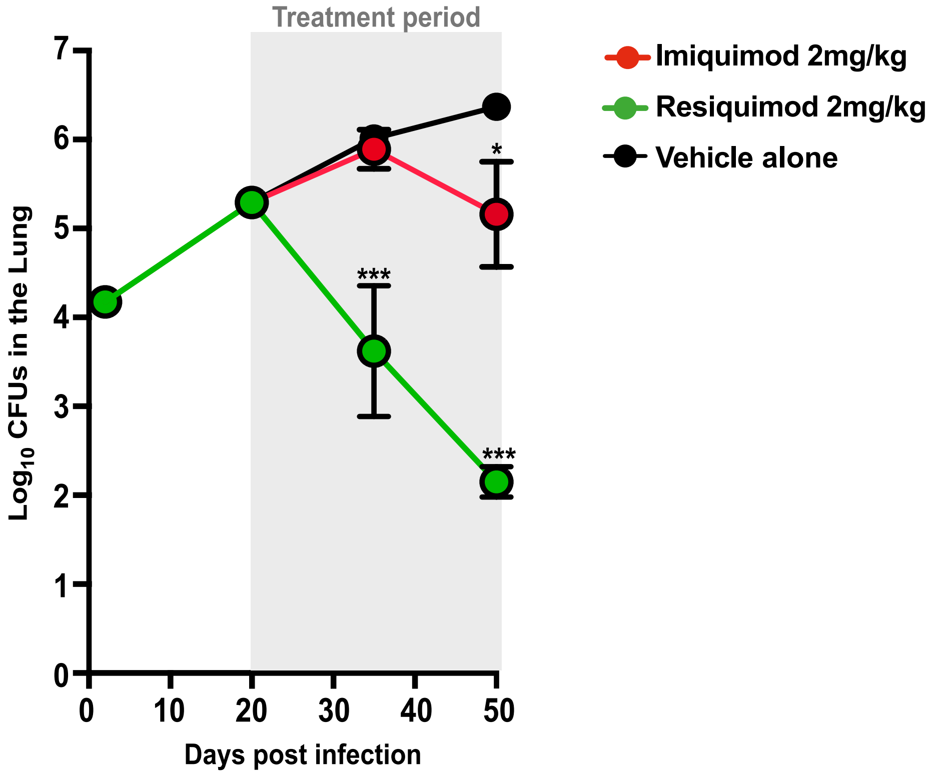

Supplementary Figure 6

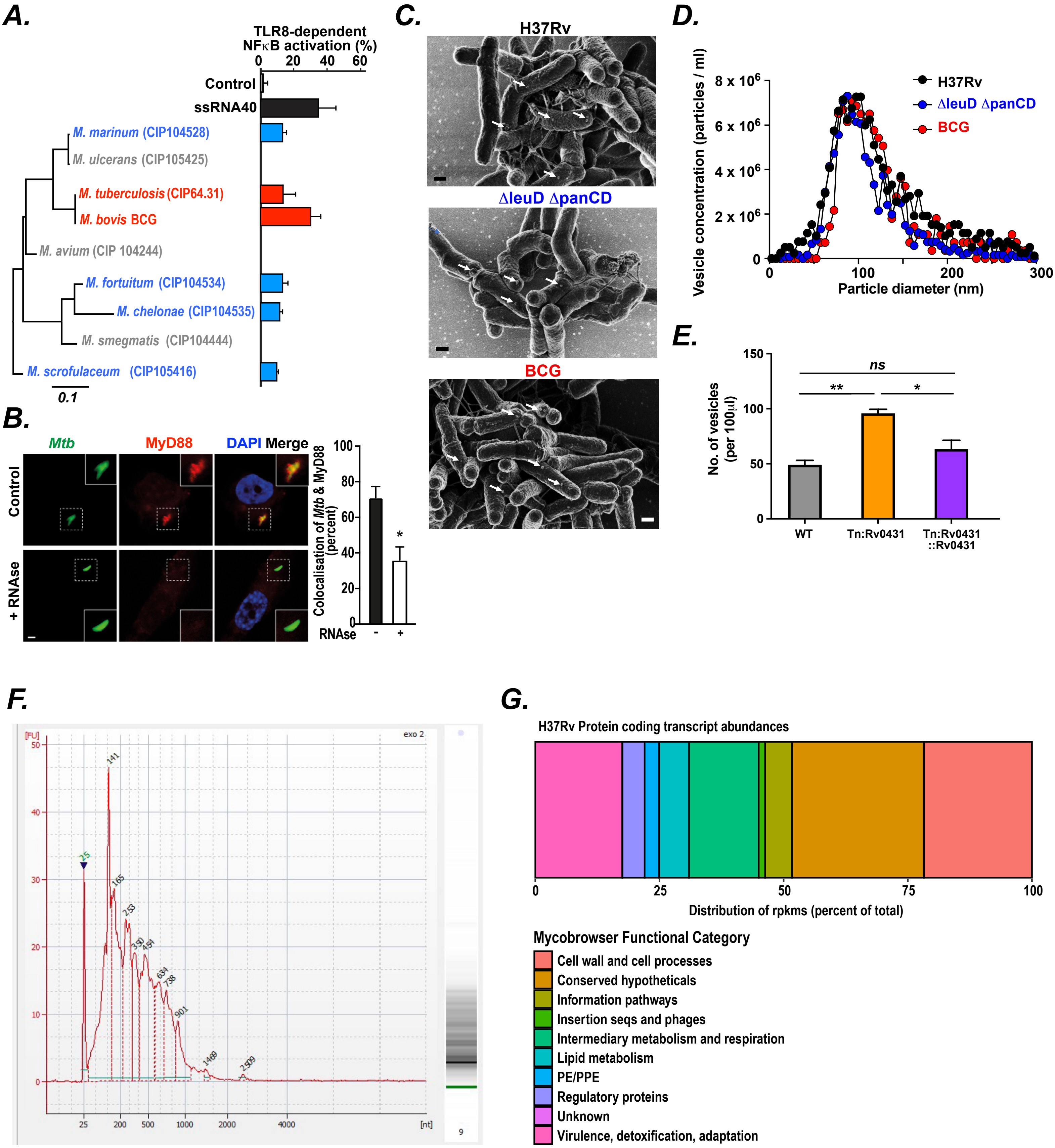

Supplementary Figure 7

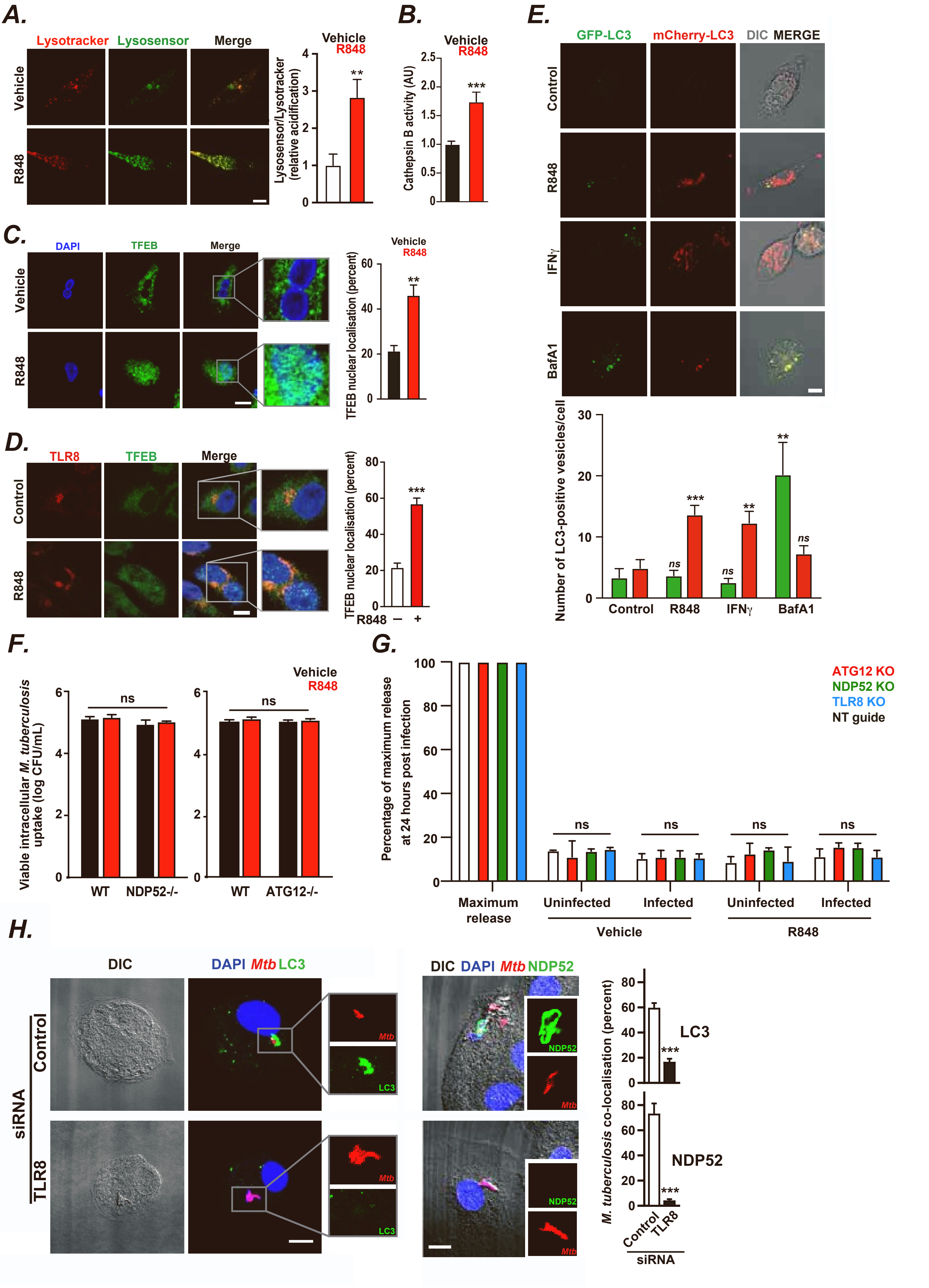

Supplementary Figure 8

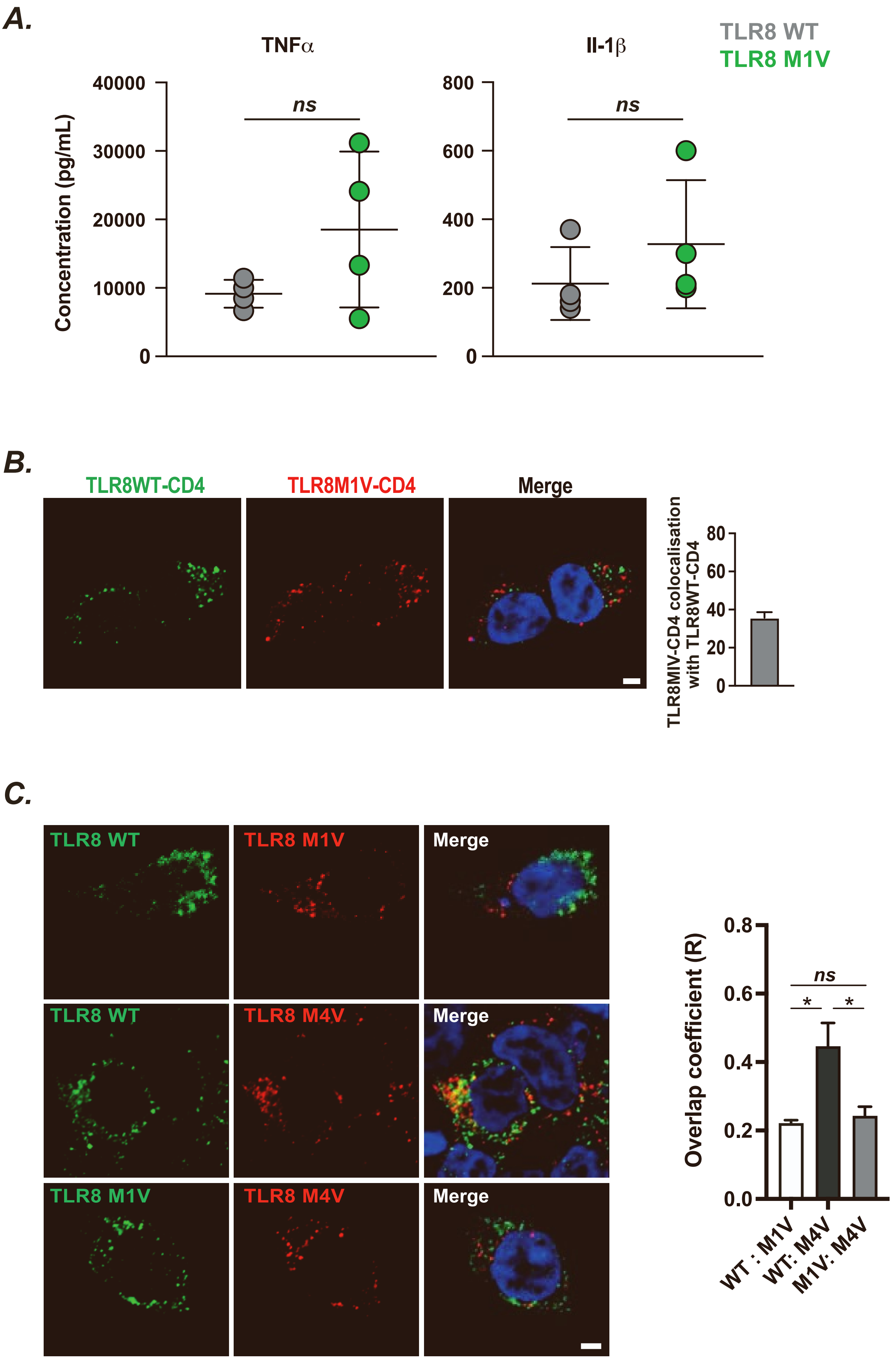

Figure S1. Genome-wide CRISPR screen of M. tuberculosis-infected THP-1 cells, Related to Figure 1. (A) Schematic representation of CRISPR screen workflow. **(B)** Representative flow cytometry histograms of THP-1 macrophages expressing the CRISPR knockout library showing uninfected cells (grey), cell infected with GFP-expressing *M. tuberculosis* ΔleuD ΔpanCD (Bleupan) after 2 h (blue) and 24 h (orange), and the FACS-sorted population (green). **(C).** Network propagation identifies interactions between gene hits in enriched GO terms (identified through REVIGO). The −log10(P value) of each gene from the CRISPR screen was used as a starting weight for propagation of the network. Node size reflects the degree of connectivity per node. **(D)** Visualisation of Gene Ontology (GO) terms (using CirGO (Circular Gene Ontology) python script), assigned to hits using Panther Tools and summarized and cleared for redundancy using REVIGO (Reduce & Visualize Gene Ontology terms) software.

Figure S2. Specificity of Resiquimod for human TLR8 and mouse TLR7 in macrophages, Related to Figure 1. **(A, B)** Viable intracellular *Mtb* (*Mtb* ΔleuD ΔpanCD Bleupan), expressed as the ratio of cell-associated CFUs at 24h/2h, in **(A)** wild type (WT) or TLR8 knockout (TLR8-/-) THP-1 macrophages or **(B)** bone marrow-derived macrophages from wild type (WT) or TLR7 knockout (TLR7-/-) mice, treated with Resiquimod (red) or vehicle alone (white). **(C, D)** The direct effect of Resiquimod (at a range of concentrations) on *M. tuberculosis* growth (in the absence of mammalian cells) assessed **(C)** after 24 h and **(D)** as growth curves (measured by absorbance).**(E, F)** Assessment of macrophages viability upon *Mtb* infection by LDH assay **(E)** and flow cytometry analysis of Live or Dye staining **(F)**. **(E)** Quantification of LDH released by Human Primary Macrophages after 24hours of infection with a range of *M. tuberculosis* MOIs, treated with Resiquimod (red) or vehicle alone (white). Data shown as percentage of maximum LDH release by lysed macrophages. **(F)** Quantification (left) of non-targeting guide (control, top) and TLR8 KO (bottom) THP1 cells positive to Live or Dye staining 24hours after infection with a range of *M. tuberculosis* MOIs, treated with Resiquimod (red) or vehicle alone (white). Representative flow cytometry histograms (right) of Live or Dye positive cells staining in non-targeting guide (control, top) and TLR8 KO (bottom) THP1 cells.

Figure S3. Expression of human Toll-like receptors in mature haemopoietic cell types, Related to Figure 1. (**A**) Heatmap shows the expression of Toll-like receptor (TLR) genes across mature hematopoietic cell types from BLUEPRINT consortium[76] data sets (n = 90). Values were normalised to fragments per kilobase per million (FPKM) and are displayed as log2(FPKM + 1). Key: mesenchymal stem cells (MSC), blood outgrowth endothelial cell progenitors (BOEC), umbilical vein endothelial cells (resting and proliferating; HUVEC R and P), platelets (PLT), megakaryocytes (MK), erythroblast (EB), eosinophils (EOS), basophils (BAS), neutrophils (NEU), monocytes (MONO), macrophages (M0), lipopolysaccharide-activated macrophages (M1), alternatively activated macrophages (M2), dendritic cells (DC), naïve CD4/CD8 cells (CD4/CD8 naive), central memory CD4/CD8 lymphocytes (CD4/CD8 CM), effector memory CD4/CD8 lymphocytes (CD4/CD8 EM), terminally differentiated effector memory CD4/CD8 lymphocytes (CD4/CD8 TDEM), regulatory CD4 lymphocytes (T reg), naive B lymphocytes (B naive), memory B lymphocytes (B M), class switch B lymphocytes (B CS), natural killer cells (NK). These data can be publically accessed at https://blueprint.haem.cam.ac.uk/bloodatlas/.

(**B**) Lack of contribution of TLR7 to Resiquimod signaling in THP1 macrophages. Wild type (WT) and TLR7-/- knockout differentiated THP1 cells were infected with a luminescent reporter strain of *Mtb* (*Mtb* ΔleuD ΔpanCD Bleupan) at an input MOI of 10:1. Viable intracellular *Mtb* (assessed by cell-associated luminescence, reported as relative light units; RLU) were determined at 2h and 24h post infection in the presence of Resiquimod (10 μg/ml; *red*) or vehicle alone (*white*). Data (mean +/- SEM) are representative of experiments performed in at least triplicate on at least three independent occasions. * $p < 0.05$; ** $p < 0.005$ (Student's t-test).

Figure S4. Enhanced intracellular killing of *M. tuberculosis* clinical isolates by Resiquimod, related to Figure 1. (A).THP1 macrophages were infected with a phylogenetically diverse collection of drug-susceptible (red) or multidrugresistant (blue) *Mtb* clinical isolates and co-treated with Resiquimod or vehicle alone for 24 or 48 hours and viable intracellular mycobacteria were enumerated through cell-associated colony forming units (CFU/ml shown for each clinical isolate at each time point. **(B)** Quantile-quantile plot comparing empirically observed uptake CFU values to those expected from a normal distribution with matching mean and variance. **(C, D)** Viable intracellular *Mtb* (mean ± SD) recovered from THP1 macrophages infected with the clinical isolates shown in (A) at 2 h **(C)** and 24 h and 48 h post-infection **(D)**, with Resiquimod (green) or vehicle control (white). Panel D is normalized to uptake shown in (C).

Figure S5. Effect of Resiquimod on *M. tuberculosis* infection *in vivo,* Related to Figure 1. (A) C57BL/6 mice were infected via aerosol with *Mycobacterium tuberculosis* MDR strain M10 and were then treated with Resiquimod (2 mg/kg, i.p. once daily; green), Imiquimod (2mg/kg, ip once daily; red) or vehicle control (black) for 30 days. Viable mycobacteria were enumerated at 1, 20, 35 and 50 days post infection by plating serial dilutions of lung homogenates on nutrient 7H11 agar and quantifying CFU after 3-4 weeks incubation at 37ºC, and are expressed as log10 CFU (mean +/- SEM, n = 5 mice per condition). *p < 0.05, ***p< 0.001 (determined by ANOVA and the Tukey post-test).

Figure S6. Extracellular membrane vesicles produced by H37Rv, ΔleuD ΔpanCD (Bleupan) *M. tuberculosis*, and *M. bovis* BCG, Related to Figure 2. (A) TLR8-dependent NFkB activation in response to homogenates derived from M. tuberculosis and M. bovis BCG (red) as well from rapid-growing (*M. fortuitum, M. chelonae*) and slow-growing (*M. marinum, M. scrofulaceum*) nontuberculous mycobacteria (blue) at varying evolutionary distance from the MTB complex was assessed using NFkB reporter THP-1 macrophages (THP-1-Blue) following transfection with TLR8 siRNA or scrambled controls. The response to the TLR8 ligand ssRNA40 (black) is shown as a positive control. Maximum likelihood phylogenetic tree (generated using RAxML, version 2.8.2) of representative isolates shown (scale bar indicates the number of substitutions per variable site). **(B)** TLR8 detects *Mtb*-derived RNA within macrophage phagosomes. THP-1 macrophages were infected with GFP-labelled *Mtb* (ΔleuD ΔpanCD (BleuPan)) in the presence of RNAse A (100 µg/ml) or vehicle (control). Quantitative confocal microscopy was used to detect colocalization of Mtb (green) with MyD88 (red) in both conditions. Nuclei stained with DAPI (blue). **(C)** Scanning electron micrographs of *Mtb* H37Rv (*top*), *Mtb* ΔleuD ΔpanCD (Bleupan) (*middle*), and *M. bovis* BCG (*bottom*) illustrating MV production. Scale bar 200 nm. **(D)** Quantification of extracellular membrane vesicles (MV) in culture supernatants of *Mtb* H37Rv (black), *Mtb* ΔleuD ΔpanCD (Bleupan) (blue), and *M. bovis* BCG (*red*) using nanoparticle tracking analysis. Data shown indicates mean values of three independent biological replicates, show no significant difference in MV characteristics between *Mtb* strains. **(E)** Quantification of membrane vesicles obtained from wild type (WT, *grey*), ΔvirR mutants ($virR^-$, *yellow*) and complemented ΔvirR mutants ($virR^-$: WT, *purple*). $^*p < 0.05$, $^{***}p < 0.001$ (determined by ANOVA). **(F)** RNA was extracted from *Mtb* H37Rv and analysed using an Agilent Bioanalyzer. Tapestation gel shows RNA profile. Graph shows RNA concentration in fluorescent units (fu) against nucleotide length (nt). Representative of 3 independent experiments. **(G)** Relative abundance of protein coding RNA reads stratified by Mycobrowser functional categories.

Figure S7. Activation of TLR8 increases TFEB translocation and stimulates xenophagy, Related to Figure 3. (A,B). Activation of TLR8 using Resiquimod increases numbers and degradative capacity of lysosomes. Uninfected THP-1 macrophages were incubated with Resiquimod and **(A)** subsequently incubated with LysoTrackerRed DND-99 and LysoSensorGreen DND-189 for 15 minutes prior to live confocal imaging to visualise total numbers of lysosomes (red) and acidified lysosomes (green), or **(B)** incubated with Magic Red™ MR-(RR)2 reagent for 1 hour prior to fluorescence measurements (to evaluate cathepsin B activity). Data (mean +/- SEM) are representative of at least three independent experiments performed in triplicate. *** $p < 0.001$ (ANOVA). **(C)** Resiquimod treatment of uninfected primary human macrophages for 24h leads to nuclear translocation (and presumed activation) of TFEB monitored and quantified by confocal microscopy (TFEB green, nuclei blue). Images and data (mean +/- SEM) are representative of experiments performed in triplicate on at least three independent occasions with a minimum of 50 cells analysed per replicate. ** $p < 0.01$, Student's t-test. Scale bar, 4 µm. **(D)** HeLa cells stably expressing Flag-tagged TFEB (green) were transfected with myc-tagged TLR8 (red) and treated with Resiquimod or vehicle control for 24 h. Nuclear translocation of TFEB was quantified by confocal microscopy of DAPI-stained cells (blue). Experiments were performed in triplicate, with at least 50 cells analysed per sample. Data (mean +/- SEM) are representative of at least two independent experiments. ***$p < 0.001$ (Student's t-test). Scale bar 5 µm. **(E)** Bone marrow-derived macrophages from transgenic mice expressing mRFP-GFP-tagged LC3 (56) were treated with vehicle control, Resiquimod (10 µg/ml), and interferon gamma (IFN-g; 200 ng/ml) as a positive control for autophagic flux enhancement, or Bafilomycin A1 (BafA1; 200 nM) as an inhibitor of autophagic flux, for 24 h and imaged using live cell confocal microscopy to quantify acidified (mCherry+ GFP–; red) and non-acidified (mCherry+ GFP+, green) vesicles. Experiments were performed in duplicate, with >50 cells analysed per sample. Data (mean +/- SEM) are representative of at least three independent repeats. **$p < 0.01$; ns, non-significant (Student's t-test). Scale bar 5µm. **(F)** Viable intracellular *Mtb* quantitation at 2 hours post infection in Control, NDP52 KO (left) or ATG12 KO (right) THP1 cells, treated with either Resiquimod (R848, red) or vehicle (black). **(G)** Quantification of LDH released by Non-Targetting guide control (white) TLR8 KO (blue) ATG12 (red) or NDP52 KO (green) THP1 cells after 24hours of infection with *M. tuberculosis* at MOI 10:1, treated either with Resiquimod (R848) or vehicle alone. Data shown as percentage of maximum LDH release by lysed macrophages. **(H)** Representative images of LC3 (left) or NDP52 (right) recruitment to *Mtb*-containing phagosomes in Human primary macrophages from M1V hemizygous healthy male volunteers either transfected with control (top) or TLR8 siRNA(bottom). Histogram of LC3 (top) or NDP52 (bottom) recruitment to Mtb-containing phagosomes in control (white) or TLR8

knocked down (black) Human primary macrophages. Data (mean +/- SEM) are representative of at least three independent experiments. ***p < 0.001 (Student's t-test). Scale bar 5 μm.

Figure S8. Cytokine profile expression of TLR8 M1V Primary Human Macrophages during *M. tuberculosis* infection, Related to Figure 4. (A) Primary human macrophages from healthy volunteers that are homozygous or hemizygous for the ancestral TLR8 (TLR8 WT, grey) or M1V variant (TLR8 M1V, green) (n = 5 for each genotype) were infected with either *Mtb* CDC1551 or *M. bovis* BCG. Secreted cytokines were measure in supernatants at 24 hours post infection. **(B)** Confocal microscopy of HEK 293T cells co-transfected with two of the following TLR8 receptors: (top) wild type (green) and M1V (red); (middle) wild type (green) and M4V (where the second methionine has been mutated to a valine to enforce transcription from the first methionine, red); and (bottom) M1V (green) and M4V (red). Quantification of receptor colocalization indicates only limited colocalization of the M1V variant with the other receptors, suggesting a descrete effect of the truncated signal peptide on intracellular localization. Scale bar 2 μm. **(C)** HEK293T cells were co-transfected with chimaeric receptors made from the signal peptide and intracellular domain of TLR8 joined to the extracellular and transmembrane domains of CD4. Cells were simultaneously transfected with tagged chimaeric receptors containing the wild type signal peptide (WT TLR8-CD4, green) and M1V signal peptide (M1V TLR8-CD4, red), and imaged using confocal microscopy. Quantification showing limited colocalization of WT and M1V fusion receptors shown. Scale bar 2μm. All experiments were performed in triplicate, with at least 50 cells analysed per coverslip. Data (mean +/- SEM) are representative of at least three independent experiments. Vesicles per cell and overlap coefficients were quantified using Image J. *$p < 0.05$, (Student's t-test).

TABLE 2

DRUGGABLE GENES

Gene	Gene Name	HGNC ID	Location	FC	*p* value	-Log10(*p* value)
ACVR2B	activin A receptor type 2B	HGNC:174	3p22.2	2422709039	0011205167	1950581663
ADM	adrenomedullin	HGNC:259	11p15.4	1010620394	0022557127	164671622
ADORA2B	adenosine A2b receptor	HGNC:264	17p12	403870386	0042496453	1371647322
ADRA1A	adrenoceptor alpha 1A	HGNC:277	8p21.2	1418884162	0017517248	1756534118
ALDH1A1	aldehyde dehydrogenase 1 family member A1	HGNC:402	9q21.13	4293361497	0006067427	221699546
APEX1	apurinic/apyrimidinic endodeoxyribonuclease 1	HGNC:587	14q11.2	5633394859	0034324999	146438947
APOF	apolipoprotein F	HGNC:615	12q13.3	7872610167	0027156628	1566124162
BIRC3	baculoviral IAP repeat containing 3	HGNC:591	11q22.2	1207276882	0019816999	1702962122
BMP5	bone morphogenetic protein 5	HGNC:1072	6p12.1	344462978	0048441552	131478195
BMP6	bone morphogenetic protein 6	HGNC:1073	6p24.3	1493907417	001702794	1768837901
BOC	BOC cell adhesion associated, oncogene regulated	HGNC:17173	3q13.2	1805727403	171E-04	3766349101
BPIFA2	BPI fold containing family A member 2	HGNC:16203	20q11.21	5881979322	0033444243	1475678626
BRD3	bromodomain containing 3	HGNC:1104	9q34.2	4536857795	0039829721	139979274
BTD	biotinidase	HGNC:1122	3p25.1	5033092367	0037407643	1427039655
C1QTNF8	C1q and TNF related 8	HGNC:31374	16p13.3	1019304522	0001345599	2871084451
C4BPA	complement component 4 binding protein alpha	HGNC:1325	1q32.2	8464404551	0026006752	1584913876
CA1	carbonic anhydrase 1	HGNC:1368	8q21.2	5385763258	0004501639	2346629318
CA6	carbonic anhydrase 6	HGNC:1380	1p36.23	3259887579	0008391643	2076153021
CASP6	caspase 6	HGNC:1507	4q25	616184837	0032220972	1491861366
CCR8	C-C motif chemokine receptor 8	HGNC:1609	3p22.1	6754418004	003041053	1516976012
CD5L	CD5 molecule like	HGNC:1690	1q23.1	2349722226	0011327494	194586615
CEACAM3	CEA cell adhesion molecule 3	HGNC:1815	19q13.2	1039273176	0022312472	1651452302
CFHR3	complement factor H related 3	HGNC:16980	1q31.3	5755701423	0033884621	1469997367
CHRNA1	cholinergic receptor nicotinic alpha 1 subunit	HGNC:1955	2q31.1	4422125293	000599403	2222281056
CNDP1	carnosine dipeptidase 1	HGNC:20675	18q22.3	1215468887	0019719137	1705112099
COL22A1	collagen type XXII alpha 1 chain	HGNC:22989	8q24.23-q24.3	4084785101	0042447522	1372147661
CPT1B	carnitine palmitoyltransferase 1B	HGNC:2329	22q13.33	2470553564	0010691393	1970965704
CST5	cystatin D	HGNC:2477	20p11.21	3380572874	0049395704	1306310822
CTSH	cathepsin H	HGNC:2535	15q25.1	2957035302	0009345794	2029383778
CXCL16	C-X-C motif chemokine ligand 16	HGNC:16642	17p13.2	3146782157	0008758624	2057564114
CXCR5	C-X-C motif chemokine receptor 5	HGNC:1060	11q23.3	650202224	003126682	1504916287
CYP24A1	cytochrome P450 family 24 subfamily A member 1	HGNC:2602	20q13.2	1369859033	0017761902	175051052
CYP2W1	cytochrome P450 family 2 subfamily W member 1	HGNC:20243	7p22.3	1454641344	367E-04	3435355882
DAND5	DAN domain BMP antagonist family member 5	HGNC:26780	19p13.13	2864992174	0009345794	2029383778
DCN	decorin	HGNC:2705	12q21.33	1354177663	0018128884	1741628933
DKK1	dickkopf WNT signaling pathway inhibitor 1	HGNC:2891	10q21.1	4450674334	0040294564	1394753541
DKK4	dickkopf WNT signaling pathway inhibitor 4	HGNC:2894	8p11.21	754963855	0027988452	1553021116
DMD	dystrophin	HGNC:2928	Xp21.2-p21.1	1785252211	0014874982	1827543561
ECE1	endothelin converting enzyme 1	HGNC:3146	1p36.12	3528266083	0007510887	2124308765
EFEMP1	EGF containing fibulin extracellular matrix protein 1	HGNC:3218	2p16.1	5082307833	0005015413	229969328
ELANE	elastase, neutrophil expressed	HGNC:3309	19p13.3	5971114156	0033101727	1480149344
ENPP1	ectonucleotide pyrophosphatase/phosphodiesterase 1	HGNC:3356	6q23.2	8858806179	0025199393	1598609916
EPHB3	EPH receptor B3	HGNC:3394	3q27.1	3220551933	0008587366	2066140024
ESAM	endothelial cell adhesion molecule	HGNC:17474	11q24.2	1106400126	0021284924	1671927888
F2RL1	F2R like trypsin receptor 1	HGNC:3538	5q13.3	805179697	0026520526	1576417858
FBLN5	fibulin 5	HGNC:3602	14q32.12	3438009916	0007682145	2114517493
FGF22	fibroblast growth factor 22	HGNC:3679	19p13.3	4300228722	004122425	1384847235
FKBP14	FKBP prolyl isomerase 14	HGNC:18625	7p14.3	7769526968	0027425747	1561841528
GALK1	galactokinase 1	HGNC:4118	17q25.1	4850411796	0038288398	1416932799
GGT6	gamma-glutamyltransferase 6	HGNC:26891	17p13.2	7377477169	0002397612	2620221065
GLP2R	glucagon like peptide 2 receptor	HGNC:4325	17p13.1	2429996363	0011156236	1952482298
GLRA3	glycine receptor alpha 3	HGNC:4328	4q34.1	9310451583	0024563292	1609713428
GLRA4	glycine receptor alpha 4 (pseudogene)	HGNC:31715	Xq22.2	1751911967	0015144101	1819756492
GPC4	glypican 4	HGNC:4452	Xq26.2	3843716209	0006727993	2172114447
GPR3	G protein-coupled receptor 3	HGNC:4484	1p36.11	6190308222	003212311	1493182415
GPR37L1	G protein-coupled receptor 37 like 1	HGNC:14923	1q32.1	9707398108	0001345599	2871084451
GRIA3	glutamate ionotropic receptor AMPA type subunit 3	HGNC:4573	Xq25	2547439459	0010642462	1972957884
GUSB	glucuronidase beta	HGNC:4696	7q11.21	4510090635	0039927582	1398726986
HDGF	heparin binding growth factor	HGNC:4856	1q23.1	3574473746	0046998092	1327919776
HPSE2	heparanase 2 (inactive)	HGNC:18374	10q24.2	6256444536	0031976317	1495171553
HRC	histidine rich calcium binding protein	HGNC:5178	19q13.33	3695698605	0045799286	1339141296
HTR1B	5-hydroxytryptamine receptor 1B	HGNC:5287	6q14.1	9179475443	0024685619	1607555974
ICMT	isoprenylcysteine carboxyl methyltransferase	HGNC:5350	1p36.31	6968725345	0029529774	152973987
IL11RA	interleukin 11 receptor subunit alpha	HGNC:5967	9p13.3	6512600288	0031242355	1505256243
IL20RB	interleukin 20 receptor subunit beta	HGNC:6004	3q22.3	1010307037	002302197	1637857517
IL3	interleukin 3	HGNC:6011	5q31.1	4100967465	0042325195	1373401037
IL32	interleukin 32	HGNC:16830	16p13.3	4423800642	0040490287	1392649142
ITGAX	integrin subunit alpha X	HGNC:6152	16p11.2	1515357121	0016881147	177259805
KCNK15	potassium two pore domain channel subfamily K member 15	HGNC:13814	20q13.12	4668315893	0039169154	1407055809
KLHL1	kelch like family member 1	HGNC:6352	13q21.33	1607401518	0016024857	1795205841
KLHL18	kelch like family member 18	HGNC:29120	3p21.31	1816697646	0014654793	1834020318
KLK10	kallikrein related peptidase 10	HGNC:6358	19q13.41	1451623322	0017419386	1758967147
KNG1	kininogen 1	HGNC:6383	3q27.3	4990022861	0037603366	1424773273
LPL	lipoprotein lipase	HGNC:6677	8p21.3	1259423359	0019009639	1721026122
MAP3K1	mitogen-activated protein kinase kinase kinase 1	HGNC:6848	5q11.2	4349268863	00409062	1388210867
MAP3K19	mitogen-activated protein kinase kinase kinase 19	HGNC:26249	2q21.3	3816534721	0006776924	2168967372
MAP3K3	mitogen-activated protein kinase kinase kinase 3	HGNC:6855	17q23.3	5953280236	0033150658	1479507845
MAP3K5	mitogen-activated protein kinase kinase kinase 5	HGNC:6857	6q23.3	4172728774	0041909282	1377689778
MAP4K2	mitogen-activated protein kinase kinase kinase kinase 2	HGNC:6864	11q13.1	3539674012	0047389539	132431752
MAP4K4	mitogen-activated protein kinase kinase kinase kinase 4	HGNC:6866	2q11.2	6485720461	0031315751	1504237171
MASP1	mannan binding lectin serine peptidase 1	HGNC:6901	3q27.3	7502034706	0028012918	1552641654
MDM2	MDM2 proto-oncogene	HGNC:6973	12q15	8665116798	0001761511	2754114644
MET	MET proto-oncogene, receptor tyrosine kinase	HGNC:7029	7q31	38852002	0044184567	1354729395
MMP14	matrix metallopeptidase 14	HGNC:7160	14q11.2	580741427	0033639967	1473144442
MMP25	matrix metallopeptidase 25	HGNC:14246	16p13.3	5715449891	0004036796	2393963196
MSTN	myostatin	HGNC:4223	2q32.2	4214727213	0006214219	2206613424
MYH7	myosin heavy chain 7	HGNC:7577	14q11.2	447386694	004009884	1396868187
NCSTN	nicastrin	HGNC:17091	1q23.2	6020569691	0032832608	1483694625
NEGR1	neuronal growth regulator 1	HGNC:17302	1p31.1	2130160481	0012844351	1891287837
NGF	nerve growth factor	HGNC:7808	1p13.2	6136258972	0032269903	1491202345
OC90	otoconin 90	HGNC:8100	8q24.22	142292473	0017492783	1757141099
OVGP1	oviductal glycoprotein 1	HGNC:8524	1p13.2	5118716246	0036918334	1432757901

KNOWN MYCOBCATERIAL REGULATION

Gene	Gene Name	HGNC ID	Location	FC	*p* value	-Log10(*p* value)
ABCD4	ATP binding cassette subfamily D member 4	HGNC:68	14q24.3	513230236	0004990948	2301816973
ACAD8	acyl-CoA dehydrogenase family member 8	HGNC:87	11q25	167689087	0015731272	1803236168
ACSL1	acyl-CoA synthetase long chain family member 1	HGNC:3569	4q35.1	405707005	0006434408	2191491392
ADRA1A	adrenoceptor alpha 1A	HGNC:277	8p21.2	141888416	0017517248	1756534118
ALDH18A1	aldehyde dehydrogenase 18 family member A1	HGNC:9722	10q24.1	633628792	0003278368	2484342342
ARHGEF12	Rho guanine nucleotide exchange factor 12	HGNC:14193	11q23.3	414655794	0042031609	1376423981
ARL8B	ADP ribosylation factor like GTPase 8B	HGNC:25564	3p26.1	119760651	979E-04	3009387149
ATF3	activating transcription factor 3	HGNC:785	1q32.3	454270837	0039756324	1400593775
ATG7	autophagy related 7	HGNC:16935	3p25.3	559212761	0034545188	1461612444
BANK1	B cell scaffold protein with ankyrin repeats 1	HGNC:18233	4q24	631696299	0031756129	1498172448
BATF2	basic leucine zipper ATF-like transcription factor 2	HGNC:25163	11q13.1	353448052	0047414004	1324093368
BIRC3	baculoviral IAP repeat containing 3	HGNC:591	11q22.2	120727688	0019816999	1702962122
C1QBP	complement C1q binding protein	HGNC:1243	17p13.2	960369054	002387826	1621997323
CASP6	caspase 6	HGNC:1507	4q25	616184837	0032220972	1491861366
CCR8	C-C motif chemokine receptor 8	HGNC:1609	3p22.1	6754418	003041053	1516976012
CD5L	CD5 molecule like	HGNC:1690	1q23.1	234972223	0011327494	194586615
CDH10	cadherin 10	HGNC:1749	5p14.2-p14.1	570622552	0034031414	1468120011
CDH24	cadherin 24	HGNC:14265	14q11.2	518846354	0036526888	1437387333
CFHR3	complement factor H related 3	HGNC:16980	1q31.3	575570142	0033884621	1469997367
CHRNA1	cholinergic receptor nicotinic alpha 1 subunit	HGNC:1955	2q31.1	442212529	000599403	2222281056
CLDN1	claudin 1	HGNC:2032	3q28	134572607	0018128884	1741628933
CLEC4A	C-type lectin domain family 4 member A	HGNC:13257	12p13.31	225016477	0012183784	1914217798
CNDP1	carnosine dipeptidase 1	HGNC:20675	18q22.3	121546889	0019719137	1705112099
CORO2A	coronin 2A	HGNC:2255	9q22.33	544074663	0034863238	1457632276
CPLX2	complexin 2	HGNC:2310	5q35.2	477743245	0038704311	1412240661
CPT1B	carnitine palmitoyltransferase 1B	HGNC:2329	22q13.33	247055356	0010691393	1970965704
CREM	cAMP responsive element modulator	HGNC:2352	10p11.21	625052694	0032049714	1494175845
CTSH	cathepsin H	HGNC:2535	15q25.1	29570353	0009345794	2029383778
CXCL16	C-X-C motif chemokine ligand 16	HGNC:16642	17p13.2	314678216	0008758624	2057564114
DHCR24	24-dehydrocholesterol reductase	HGNC:2859	1p32.3	87405589	0001688115	277259805
ELANE	elastase, neutrophil expressed	HGNC:3309	19p13.3	597111416	0033101727	1480149344
ELMO2	engulfment and cell motility 2	HGNC:17233	20q13.12	886709776	0001639184	2785372338
ENPP1	ectonucleotide pyrophosphatase/phosphodiesterase 1	HGNC:3356	6q23.2	885880618	0025199393	1598609916
EPSTI1	epithelial stromal interaction 1	HGNC:16465	13q14.11	537336424	0035352547	1451579294
FAM180B	family with sequence similarity 180 member B	HGNC:34451	11p11.2	863146508	0001785976	274812428
FBLN5	fibulin 5	HGNC:3602	14q32.12	343800992	0007682145	2114517493
FTH1	ferritin heavy chain 1	HGNC:3976	11q12.3	187156278	0014116553	1850271327
FZD1	frizzled class receptor 1	HGNC:4038	7q21.13	519360156	0036257768	1440598937
GAL3ST3	galactose-3-O-sulfotransferase 3	HGNC:24144	11q13.1	12177049	0019719137	1705112099
GALK1	galactokinase 1	HGNC:4118	17q25.1	48504118	0038288398	1416932799
GATA4	GATA binding protein 4	HGNC:4173	8p23.1	576521701	0033762294	1471568054
GRIA3	glutamate ionotropic receptor AMPA type subunit 3	HGNC:4573	Xq25	254743946	0010642462	1972957884
GUSB	glucuronidase beta	HGNC:4696	7q11.21	451009064	0039927582	1398726986
HAUS4	HAUS augmin like complex subunit 4	HGNC:20163	14q11.2	165096491	0015804668	1801214623
IL11RA	interleukin 11 receptor subunit alpha	HGNC:5967	9p13.3	651260029	0031242355	1505256243
IL20RB	interleukin 20 receptor subunit beta	HGNC:6004	3q22.3	101030704	002302197	1637857517
IL3	interleukin 3	HGNC:6011	5q31.1	410096746	0042325195	1373401037
IL32	interleukin 32	HGNC:16830	16p13.3	442380064	0040490287	1392649142
INPP4A	inositol polyphosphate-4-phosphatase type I A	HGNC:6074	2q11.2	373912886	0045432304	1342635237
IRF8	interferon regulatory factor 8	HGNC:5358	16q24.1	929152144	0024612223	160884916
ITGAX	integrin subunit alpha X	HGNC:6152	16p11.2	151535712	0016881147	177259805
KTN1	kinectin 1	HGNC:6467	14q22.1	460116467	0039633997	1401932126
MAP3K3	mitogen-activated protein kinase kinase kinase 3	HGNC:6855	17q23.3	595328024	0033150658	1479507845
MASP1	mannan binding lectin serine peptidase 1	HGNC:6901	3q27.3	750203471	0028012918	1552641654
MRPL3	mitochondrial ribosomal protein L3	HGNC:10379	3q22.1	380631482	0006874786	2162740821
MS4A14	membrane spanning 4-domains A14	HGNC:30706	11q12.2	701037865	0029382982	1531904133
MSTN	myostatin	HGNC:4223	2q32.2	421472721	0006214219	2206613424
MYO5B	myosin VB	HGNC:7603	18q	387019348	0044257963	1354008574
NEBL	nebulette	HGNC:16932	10p12.31	393495755	0043866517	1357866851
NINJ1	ninjurin 1	HGNC:7824	9q22.31	905582783	0024881343	1604126188
NXPE4	neurexophilin and PC-esterase domain family member 4	HGNC:23117	11q23.2	627233688	0031780594	149783799
OSGIN1	oxidative stress induced growth inhibitor 1	HGNC:30093	16q23.3	409250022	0042447522	1372147661
P2RX2	purinergic receptor P2X 2	HGNC:15459	12q24.33	848798708	0025933356	1586141275
PCDH12	protocadherin 12	HGNC:8657	5q31.3	971391622	0023682537	1625571783
PDGFRA	platelet derived growth factor receptor alpha	HGNC:8803	4q12	589746344	0003327299	2477908232
PLG	plasminogen	HGNC:9071	6q26	348166661	0007608749	2118686752
PLIN2	perilipin 2	HGNC:248	9p22.1	263456872	0010177619	199235381
POLR2G	RNA polymerase II subunit G	HGNC:9194	11q12.3	122307889	0019719137	1705112099
PPP2R5A	protein phosphatase 2 regulatory subunit B'alpha	HGNC:9309	1q32.3	28497339	0009492587	2022615415
PTGES2	prostaglandin E synthase 2	HGNC:17822	9q34.11	815855363	0002030631	2692369048
QPRT	quinolinate phosphoribosyltransferase	HGNC:9755	16p11.2	194873753	0013749572	1861710825
RAB33A	RAB33A, member RAS oncogene family	HGNC:9773	Xq26.1	257420507	0010324412	198613469
RAB3C	RAB3C, member RAS oncogene family	HGNC:30269	5q11.2	617717487	003212311	1493182415
RGS3	regulator of G protein signaling 3	HGNC:9999	9q32	60251923	0032783677	1484342342
RNF175	ring finger protein 175	HGNC:27735	4q31.3	441426745	000599403	2222281056
SAMD11	sterile alpha motif domain containing 11	HGNC:28706	1p36.33	755983174	0027988452	1553021116
SARM1	sterile alpha and TIR motif containing 1	HGNC:17074	17q11.2	117618437	0020086118	1697103983
SCCPDH	saccharopine dehydrogenase (putative)	HGNC:24275	1q44	78602998	0027205559	1565342353
SCNN1D	sodium channel epithelial 1 subunit delta	HGNC:10601	1p36.33	108817848	0021676371	1664013419
SCPEP1	serine carboxypeptidase 1	HGNC:29507	17q22	573559675	0033933552	146937068
SDC1	syndecan 1	HGNC:10658	2p24.1	87286175	0025395117	1595249787
SDC4	syndecan 4	HGNC:10661	20q13.12	365346797	0045799286	1339141296
SERPINA1	serpin family A member 1	HGNC:8941	14q32.13	598602238	00329794	1481757248
SERPINB2	serpin family B member 2	HGNC:8584	18q21.33-q22.1	397081951	0006679062	2175284494
SFXN4	sideroflexin 4	HGNC:16088	10q26.11	183297619	0014483535	1839125434
SHISAL1	shisa like 1	HGNC:29335	22q13.31	554672056	0034667515	146007729
SIN3B	SIN3 transcription regulator family member B	HGNC:19354	19p13.11	115675107	0001052014	2977978685
SLC25A34	solute carrier family 25 member 34	HGNC:27653	1p36.21	512096578	0036893869	1433045799
SLC39A7	solute carrier family 39 member 7	HGNC:4927	6p21.32	725522093	0028624554	1543261279
SLC6A6	solute carrier family 6 member 6	HGNC:11052	3p25.1	111434826	0020673289	1684590432
SLFN11	schlafen family member 11	HGNC:26633	17q12	11038535	0021431717	1668943034
SNTB1	syntrophin beta 1	HGNC:11168	8q24.12	876425474	0025199393	1598609916

XENOBIOTIC RESPONSE GO TERMS GROUP

Gene	Gene Name	HGNC ID	Location	FC	*p* value	-Log10(*p* value)
BANK1	B cell scaffold protein with ankyrin repeats 1	HGNC:18233	4q24	6316962986	00317561	1498172448
BCL6B	BCL6B transcription repressor	HGNC:1002	17p13.1	7720144365	00020796	2682028215
CCS	copper chaperone for superoxide dismutase	HGNC:1613	11q13.2	439374414	00406615	1390816121
CDH10	cadherin 10	HGNC:1749	5p14.2-p14.1	570622552	00340314	1468120011
CYP2W1	cytochrome P450 family 2 subfamily W member 1	HGNC:20243	7p22.3	1454641344	367E-04	3435355882
GLRA3	glycine receptor alpha 3	HGNC:4328	4q34.1	9310451583	00245633	1609713428
GLRA4	glycine receptor alpha 4 (pseudogene)	HGNC:31715	Xq22.2	1751911967	00151441	1819756492
HTR1B	5-hydroxytryptamine receptor 1B	HGNC:5287	6q14.1	9179475443	00246856	1607555974
IL20RB	interleukin 20 receptor subunit beta	HGNC:6004	3q22.3	1010307037	0023022	1637857517
MAP4K4	mitogen-activated protein kinase kinase kinase kinase 4	HGNC:6866	2q11.2	6485720461	00313158	1504237171
NGF	nerve growth factor	HGNC:7808	1p13.2	6136258972	00322699	1491202345
SLFN11	schlafen family member 11	HGNC:26633	17q12	11038535	00214317	1668943034
TAOK3	TAO kinase 3	HGNC:18133	12q24.23	3563437089	0007462	2127147301
TLR8	toll like receptor 8	HGNC:15632	Xp22.2	8504140759	00258355	1587783222
TNIP2	TNFAIP3 interacting protein 2	HGNC:19118	4p16.3	3003762738	00090522	2043245417
UBASH3A	ubiquitin associated and SH3 domain containing A	HGNC:12462	21q22.3	5359684177	00354259	1450678579

P2RX2	purinergic receptor P2X 2	HGNC:15459	12q24.33	8487987082	0025933356	1586141275	SOAT1	sterol O-acyltransferase 1	HGNC:11177	1q25.2	425841984	0041542301	1381509455
PADI1	peptidyl arginine deiminase 1	HGNC:18367	1p36.13	3436403389	0007755541	2110387878	SOX18	SRY-box transcription factor 18	HGNC:11194	20q13.33	562596953	0004110192	2386137859
PDE5A	phosphodiesterase 5A	HGNC:8784	4q26	3527026681	0047438469	1323869332	SPIB	Spi-B transcription factor	HGNC:11242	19q13.33	386362765	0044257963	1354008574
PDGFRA	platelet derived growth factor receptor alpha	HGNC:8803	4q12	5897463445	0003327299	2477908232	SPOCK1	SPARC (osteonectin), cwcv and kazal like domains proteoglycan 1	HGNC:11251	5q31.2	744074953	0002275285	2642964192
PENK	proenkephalin	HGNC:8831	8q12.1	3419115151	0007755541	2110387878	TAOK3	TAO kinase 3	HGNC:18133	12q24.23	356343709	0007461956	2127147301
PKD2	polycystin 2, transient receptor potential cation channel	HGNC:9009	4q22.1	1357483085	0018104418	1742215421	TENT4A	terminal nucleotidyltransferase 4A	HGNC:16705	5p15.31	440637558	000599403	2222281056
PKD2L2	polycystin 2 like 2, transient receptor potential cation channel	HGNC:9012	5q31.2	4475857141	0005578118	2253512294	TFAM	transcription factor A, mitochondrial	HGNC:11741	10q21.1	50781829	0037065127	1431034508
PLG	plasminogen	HGNC:9071	6q26	3481666613	0007608749	2118686752	TRAF3	TNF receptor associated factor 3	HGNC:12033	14q32.32	390680596	0043866517	1357866851
PLIN2	perilipin 2	HGNC:248	9p22.1	2634568721	0010177619	199235381	TXNIP	thioredoxin interacting protein	HGNC:16952	1q21.1	931905837	0024343103	161362406
PLTP	phospholipid transfer protein	HGNC:9093	20q13.12	2709516949	0009517052	2021497539	VAMP5	vesicle associated membrane protein 5	HGNC:12646	2p11.2	25088208	489E-05	4310417145
PNLIPRP2	pancreatic lipase related protein 2 (gene/pseudogene)	HGNC:9157	10q25.3	5493089391	000428145	2368409092	XDH	xanthine dehydrogenase	HGNC:12805	2p23.1	907933333	0024856877	1604553433
PSMB10	proteasome 20S subunit beta 10	HGNC:9538	16q22.1	7892964359	0027034301	1568084863							
PSPN	persephin	HGNC:9579	19p13.3	176616751	0014948378	182540593							
PTPRG	protein tyrosine phosphatase receptor type G	HGNC:9671	3p14.2	1094074747	0021480648	1667952625							
PXDN	peroxidasin	HGNC:14966	2p25.3	3744663055	0045358908	1343337411							
SCNN1D	sodium channel epithelial 1 subunit delta	HGNC:10601	1p36.33	1088178479	0021676371	1664013419							
SCPEP1	serine carboxypeptidase 1	HGNC:29507	17q22	5735596754	0033933552	146937068							
SDC1	syndecan 1	HGNC:10658	2p24.1	8728617496	0025395117	1595249787							
SDC4	syndecan 4	HGNC:10661	20q13.12	3653467966	0045799286	1339141296							
SENP6	SUMO specific peptidase 6	HGNC:20944	6q14.1	4565836889	0039658463	1401664126							
SERPINA1	serpin family A member 1	HGNC:8941	14q32.13	5986022379	00329794	1481757248							
SERPINA9	serpin family A member 9	HGNC:15995	14q32.13	7081126986	0029113862	1535900179							
SERPINB2	serpin family B member 2	HGNC:8584	18q21.33-q22.1	3970819507	0006679062	2175284494							
SIGLEC12	sialic acid binding Ig like lectin 12	HGNC:15482	19q13.41	1226338869	0019303225	1714370137							
SLC12A2	solute carrier family 12 member 2	HGNC:10911	5q23.3	7096660237	0002422078	2615811946							
SLC6A6	solute carrier family 6 member 6	HGNC:11052	3p25.1	1114348255	0020673289	1684590432							
SLCO5A1	solute carrier organic anion transporter family member 5A1	HGNC:19046	8q13.3	3457703716	0047952243	1319191069							
SLIT1	slit guidance ligand 1	HGNC:11085	10q24.1	6341224149	0003204971	2494175845							
SOAT1	sterol O-acyltransferase 1	HGNC:11177	1q25.2	4258419844	0041542301	1381509455							
SPINK1	serine peptidase inhibitor Kazal type 1	HGNC:11244	5q32	307120083	0009003278	2045599322							
SPOCK1	SPARC (osteonectin), cwcv and kazal like domains proteoglycan 1	HGNC:11251	5q31.2	7440749533	0002275285	2642964192							
SPOCK2	SPARC (osteonectin), cwcv and kazal like domains proteoglycan 2	HGNC:13564	10q22.1	3348241475	0049664824	1303951103							
SULT1A2	sulfotransferase family 1A member 2	HGNC:11454	16p11.2	5781016932	0033664432	1472828707							
TAOK3	TAO kinase 3	HGNC:18133	12q24.23	3563437089	0007461956	2127147301							
TG	thyroglobulin	HGNC:11764	8q24.22	1807807633	0014654793	1834020318							
TLR8	toll like receptor 8	HGNC:15632	Xp22.2	8504140759	0025835494	1587783222							
UBA2	ubiquitin like modifier activating enzyme 2	HGNC:30661	19q13.11	118101393	0020037187	1698163239							
VWF	von Willebrand factor	HGNC:12726	12p13.31	5575955782	0004183589	237845103							
WNK4	WNK lysine deficient protein kinase 4	HGNC:14544	17q21.2	1702964192	0015584479	1807307708							
WNT16	Wnt family member 16	HGNC:16267	7q31.31	6845841489	0030043548	1522248774							
XDH	xanthine dehydrogenase	HGNC:12805	2p23.1	907933333	0024856877	1604553433							

TABLE 4

GO TERMS GROUPS (REVIGO)	GO TERMS	FONCTION	HGNC	GENES
Cellular Import	GO:0051222	positive regulation of protein transport	HGNC=22201	TCAF1
Cellular Import	GO:0006836	neurotransmitter transport	HGNC=20151	SLC17A8
Cellular Import	GO:0006829	zinc II ion transport	HGNC=4927	SLC39A7
Cellular Import	GO:1990126	retrograde transport, endosome to plasma membrane	HGNC=20073 HGNC=18708	SNX27 , GRIP1
Cellular Import	GO:0006855	drug transmembrane transport	HGNC=11061	SLC7A3
Cellular Import	GO:0006869	lipid transport	HGNC=36	ABCA6
Cellular Import	GO:0006890	retrograde vesicle-mediated transport, Golgi to ER	HGNC=18622 HGNC=29205	COG7 , ERGIC1
Cellular Import	GO:0071705	nitrogen compound transport	HGNC=11052	SLC6A6
Cellular Import	GO:1990778	protein localization to cell periphery	HGNC=4452	GPC4
Cellular Import	GO:0016197	endosomal transport	HGNC=25104	AP5B1
Cellular Import	GO:0017157	regulation of exocytosis	HGNC=30269	RAB3C
Cellular Import	GO:0099504	synaptic vesicle cycle	HGNC=20151	SLC17A8
Cellular Import	GO:0099500	vesicle fusion to plasma membrane	HGNC=30269 HGNC=2310	RAB3C , CPLX2
Cellular Import	GO:0098657	import into cell	HGNC=11061 HGNC=10911 HGNC=14544	SLC7A3 , SLC12A2 , WNK4
Cellular Import	GO:0008298	intracellular mRNA localization	HGNC=17351	PRPF18
Cellular Import	GO:0072657	protein localization to membrane	HGNC=17359 HGNC=4452	NUP54 , GPC4
Cellular Import	GO:0006606	protein import into nucleus	HGNC=15913 HGNC=9851	NXT1 , RANBP6
Cellular Import	GO:0042886	amide transport	HGNC=10919 HGNC=9093	SLC14A2 , PLTP
Cellular Import	GO:0097120	receptor localization to synapse	HGNC=4452	GPC4
Cellular Import	GO:0015802	basic amino acid transport	HGNC=23090	SLC15A4
Cellular Import	GO:0015809	arginine transport	HGNC=11061	SLC7A3
Cellular Import	GO:0015849	organic acid transport	HGNC=11052	SLC6A6
Cellular Import	GO:0051051	negative regulation of transport	HGNC=14544	WNK4
Cellular Import	GO:0006623	protein targeting to vacuole	HGNC=1908	VPS13A
Cellular Import	GO:0042908	xenobiotic transport	HGNC=11052	SLC6A6
Cellular Import	GO:0034067	protein localization to Golgi apparatus	HGNC=1908	VPS13A
Cellular Import	GO:0009306	protein secretion	HGNC=30269	RAB3C
Cellular Import	GO:1990542	mitochondrial transmembrane transport	HGNC=29786 HGNC=16088	SLC25A37 , SFXN4
Cellular Import	GO:0061512	protein localization to cilium	HGNC=21057 HGNC=19009 HGNC=25705	RSPH9 , CLUAP1 , KCTD17
Cellular Import	GO:1902476	chloride transmembrane transport	HGNC=10911 HGNC=4328 HGNC=31715	SLC12A2 , GLRA3 , GLRA4
Cellular Import	GO:0015698	inorganic anion transport	HGNC=22201 HGNC=3356	TCAF1 , ENPP1
Cellular Import	GO:0048193	Golgi vesicle transport	HGNC=19347	CUX2
Cellular Import	GO:0006909	phagocytosis	HGNC=7029 HGNC=9802 HGNC=3309	MET , RAC2 , ELANE
Cellular Import	GO:0043266	regulation of potassium ion transport	HGNC=14544	WNK4
Cellular Import	GO:0051640	organelle localization	HGNC=30269	RAB3C
Membrane Invagination	GO:0032273	positive regulation of protein polymerization	HGNC=24164	TPPP
Membrane Invagination	GO:0051260	protein homooligomerization	HGNC=12968	RNF112
Membrane Invagination	GO:0051276	chromosome organization	HGNC=20944	SENP6
Membrane Invagination	GO:0010639	negative regulation of organelle organization	HGNC=18165	STYXL1
Membrane Invagination	GO:0010638	positive regulation of organelle organization	HGNC=25705	KCTD17
Membrane Invagination	GO:0043062	extracellular structure organization	HGNC=9093	PLTP
Membrane Invagination	GO:0097435	supramolecular fiber organization	HGNC=3602	FBLN5
Membrane Invagination	GO:0030198	extracellular matrix organization	HGNC=14246 HGNC=3602 HGNC=7160 HGNC=11345	MMP25 , FBLN5 , MMP14 , RECK
Membrane Invagination	GO:0000422	mitophagy	HGNC=25072	WDR45B
Membrane Invagination	GO:0099560	synaptic membrane adhesion	HGNC=4452	GPC4
Membrane Invagination	GO:0044089	positive regulation of cellular component biogenesis	HGNC=3420 HGNC=25705 HGNC=21295	EPS8 , KCTD17 , EPS8L1
Membrane Invagination	GO:0044087	regulation of cellular component biogenesis	HGNC=4452	GPC4
Membrane Invagination	GO:0065004	protein-DNA complex assembly	HGNC=20944	SENP6
Membrane Invagination	GO:0030865	cortical cytoskeleton organization	HGNC=9802	RAC2
Membrane Invagination	GO:0016233	telomere capping	HGNC=7652	NBN
Membrane Invagination	GO:1905349	ciliary transition zone assembly	HGNC=21057 HGNC=19009 HGNC=25705	RSPH9 , CLUAP1 , KCTD17
Membrane Invagination	GO:0010324	membrane invagination	HGNC=9782 HGNC=9802	RAB4B , RAC2
Membrane Invagination	GO:0032508	DNA duplex unwinding	HGNC=7652	NBN
Membrane Invagination	GO:0000045	autophagosome assembly	HGNC=25072	WDR45B
Membrane Invagination	GO:0016575	histone deacetylation	HGNC=23784 HGNC=19354	MTA3 , SIN3B
Membrane Invagination	GO:0001558	regulation of cell growth	HGNC=30093	OSGIN1
Membrane Invagination	GO:0007029	endoplasmic reticulum organization	HGNC=12968	RNF112
Membrane Invagination	GO:0007030	Golgi organization	HGNC=18622	COG7
Membrane Invagination	GO:0035735	intraciliary transport involved in cilium assembly	HGNC=21057 HGNC=19009 HGNC=25705	RSPH9 , CLUAP1 , KCTD17
Membrane Invagination	GO:0006998	nuclear envelope organization	HGNC=11214	SPAG4
Membrane Invagination	GO:0006997	nucleus organization	HGNC=17359	NUP54
Membrane Invagination	GO:0035082	axoneme assembly	HGNC=21057 HGNC=19009 HGNC=25705	RSPH9 , CLUAP1 , KCTD17
Membrane Invagination	GO:0097712	vesicle targeting, trans-Golgi to periciliary membrane compartment	HGNC=21057 HGNC=19009 HGNC=25705	RSPH9 , CLUAP1 , KCTD17
Membrane Invagination	GO:0030031	cell projection assembly	HGNC=9802	RAC2
Membrane Invagination	GO:0045216	cell-cell junction organization	HGNC=1749 HGNC=14265	CDH10 , CDH24
Membrane Invagination	GO:0090148	membrane fission	HGNC=25589	SEPTIN11
Membrane Invagination	GO:0008360	regulation of cell shape	HGNC=9802	RAC2
Cell Fate Commitment	GO:0007610	behavior	HGNC=7808	NGF
Cell Fate Commitment	GO:0051301	cell division	HGNC=26624	KDF1
Cell Fate Commitment	GO:0042692	muscle cell differentiation	HGNC=19098	RBPMS2
Cell Fate Commitment	GO:0001708	cell fate specification	HGNC=6132 HGNC=11598 HGNC=11597	ISL1 , TBX20 , TBX2
Cell Fate Commitment	GO:0048747	muscle fiber development	HGNC=16932	NEBL
Cell Fate Commitment	GO:0006939	smooth muscle contraction	HGNC=277	ADRA1A
Cell Fate Commitment	GO:0003009	skeletal muscle contraction	HGNC=11353	STAC
Cell Fate Commitment	GO:0001501	skeletal system development	HGNC=7160	MMP14
Cell Fate Commitment	GO:0007283	spermatogenesis	HGNC=25929	MAEL
Cell Fate Commitment	GO:0045165	cell fate commitment	HGNC=4173 HGNC=16267	GATA4 , WNT16
Cell Fate Commitment	GO:0043588	skin development	HGNC=3052	DSP
Cell Fate Commitment	GO:0030282	bone mineralization	HGNC=3356	ENPP1
Cell Fate Commitment	GO:0014033	neural crest cell differentiation	HGNC=2254	CORO1C
Cell Fate Commitment	GO:0045446	endothelial cell differentiation	HGNC=243 HGNC=22989	ADD1 , COL22A1
Cell Fate Commitment	GO:0007606	sensory perception of chemical stimulus	HGNC=15249	OR5A2
Cell Fate Commitment	GO:0007140	male meiosis	HGNC=25929	MAEL
Cell Fate Commitment	GO:0045664	regulation of neuron differentiation	HGNC=7808	NGF
Cell Fate Commitment	GO:0051960	regulation of nervous system development	HGNC=4452	GPC4
Cell Fate Commitment	GO:0031016	pancreas development	HGNC=7029	MET
Cell Fate Commitment	GO:0007422	peripheral nervous system development	HGNC=7808	NGF
Cell Fate Commitment	GO:0048732	gland development	HGNC=7029	MET
Response to Xenobiotic Stimulus	GO:0001816	cytokine production	HGNC:15632	**TLR8**
Response to Xenobiotic Stimulus	GO:0050727	regulation of inflammatory response	HGNC:1002	BCL6B
Response to Xenobiotic Stimulus	GO:0002224	toll-like receptor signaling pathway	HGNC:19118 HGNC:15632	TNIP2 , **TLR8**
Response to Xenobiotic Stimulus	GO:0051607	defense response to virus	HGNC:15632 HGNC:26633	**TLR8** , SLFN11
Response to Xenobiotic Stimulus	GO:0006954	inflammatory response	HGNC=1002	BCL6B
Response to Xenobiotic Stimulus	GO:0031098	stress-activated protein kinase signaling cascade	HGNC=18133 HGNC=6866	TAOK3 , MAP4K4
Response to Xenobiotic Stimulus	GO:0042113	B cell activation	HGNC=18233	BANK1
Response to Xenobiotic Stimulus	GO:0050852	T cell receptor signaling pathway	HGNC=12462	UBASH3A
Response to Xenobiotic Stimulus	GO:0002683	negative regulation of immune system process	HGNC=12462	UBASH3A
Response to Xenobiotic Stimulus	GO:0009410	response to xenobiotic stimulus	HGNC=20243	CYP2W1
Response to Xenobiotic Stimulus	GO:0043200	response to amino acid	HGNC=4328 HGNC=31715	GLRA3 , GLRA4
Response to Xenobiotic Stimulus	GO:0000302	response to reactive oxygen species	HGNC=1613	CCS
Response to Xenobiotic Stimulus	GO:0042493	response to drug	HGNC=5287 HGNC=20243	HTR1B , CYP2W1
Response to Xenobiotic Stimulus	GO:0019221	cytokine-mediated signaling pathway	HGNC=6004	IL20RB
Response to Xenobiotic Stimulus	GO:0009636	response to toxic substance	HGNC=1613	CCS
Response to Xenobiotic Stimulus	GO:0071363	cellular response to growth factor stimulus	HGNC=7808 HGNC=174	NGF , CDH10
Response to Xenobiotic Stimulus	GO:0071407	cellular response to organic cyclic compound	HGNC=5287	HTR1B
Response to Xenobiotic Stimulus	GO:0010035	response to inorganic substance	HGNC=1613	CCS
Response to Xenobiotic Stimulus	GO:0071417	cellular response to organonitrogen compound	HGNC=5287	HTR1B
RNA Processing	GO:0090503	RNA phosphodiester bond hydrolysis, exonucleolytic	HGNC=17035	EXOSC8
RNA Processing	GO:0006367	transcription initiation from RNA polymerase II promoter	HGNC=9194	POLR2G
RNA Processing	GO:0016071	mRNA metabolic process	HGNC=16060	TRUB1
RNA Processing	GO:0000398	mRNA splicing, via spliceosome	HGNC=9900 HGNC=17351	RBM3 , PRPF18
RNA Processing	GO:0034472	snRNA 3'-end processing	HGNC=17035	EXOSC8
RNA Processing	GO:0000466	maturation of 5.8S rRNA from tricistronic rRNA transcript (SSU-rRNA, 5.8S rRNA, LSU-rRNA)	HGNC=17035	EXOSC8
RNA Processing	GO:0002181	cytoplasmic translation	HGNC=21746	CPEB3
RNA Processing	GO:0032543	mitochondrial translation	HGNC=1243	C1QBP
RNA Processing	GO:0006575	cellular modified amino acid metabolic process	HGNC=2329	CPT1B
RNA Processing	GO:0006413	translational initiation	HGNC=9194	POLR2G
RNA Processing	GO:0006414	translational elongation	HGNC=14027 HGNC=1243 HGNC=7234 HGNC=9900 HGNC=21746 HGNC=9194	MRPL39 , C1QBP , MRRF , RBM3 , CPEB3 , POLR2G
RNA Processing	GO:0006399	tRNA metabolic process	HGNC=14027 HGNC=16219 HGNC=17035	MRPL39 , DTD1 , EXOSC8
RNA Processing	GO:0072521	purine-containing compound metabolic process	HGNC=12805	XDH
RNA Processing	GO:0031123	RNA 3'-end processing	HGNC=16705	TENT4A
RNA Processing	GO:0000737	DNA catabolic process, endonucleolytic	HGNC=2957	DNASE1L1
RNA Processing	GO:0034660	ncRNA metabolic process	HGNC=25929	MAEL
Intracellular Signalling	GO:0035249	synaptic transmission, glutamatergic	HGNC=20151	SLC17A8
Intracellular Signalling	GO:0048016	inositol phosphate-mediated signaling	HGNC=3538 HGNC=277	F2RL1 , ADRA1
Intracellular Signalling	GO:0016055	Wnt signaling pathway	HGNC=16267	WNT16
Intracellular Signalling	GO:0007265	Ras protein signal transduction	HGNC=21165 HGNC=9768	RAP2C , RAB28
Intracellular Signalling	GO:0050804	modulation of synaptic transmission	HGNC=7808	NGF
Intracellular Signalling	GO:0043123	positive regulation of I-kappaB kinase/NF-kappaB signaling	HGNC=19118	TNIP2

Intracellular Signalling	GO:0007249	I-kappaB kinase/NF-kappaB signaling	HGNC=19118 HGNC=15632 HGNC=12033	TNIP2 , **TLR8** , TRAF3
Intracellular Signalling	GO:0007200	phospholipase C-activating G-protein coupled receptor signaling pathway	HGNC=3538 HGNC=277	F2RL1 , ADRA1
Intracellular Signalling	GO:0007193	adenylate cyclase-inhibiting G-protein coupled receptor signaling pathway	HGNC=14923 HGNC=5287	GPR37L1 , HTR1B
Intracellular Signalling	GO:0007218	neuropeptide signaling pathway	HGNC=8831 HGNC=4328 HGNC=31715	PENK , GLRA3 , GLRA4
Intracellular Signalling	GO:0051896	regulation of protein kinase B signaling	HGNC=18233	BANK1
Intracellular Signalling	GO:0007169	transmembrane receptor protein tyrosine kinase signaling pathway	HGNC=7808	NGF
Intracellular Signalling	GO:0007187	G-protein coupled receptor signaling pathway, coupled to cyclic nucleotide second messenger	HGNC=15296	OR5T2
Intracellular Signalling	GO:0043491	protein kinase B signaling	HGNC=18233	BANK1
Intracellular Signalling	GO:1902532	negative regulation of intracellular signal transduction	HGNC=18233	BANK1
Catabolism process	GO:0031331	positive regulation of cellular catabolic process	HGNC=9194	POLR2G
Catabolism process	GO:0046395	carboxylic acid catabolic process	HGNC=9755 HGNC=4696	QPRT , GUSB
Catabolism process	GO:0044270	cellular nitrogen compound catabolic process	HGNC=9755 HGNC=12805 HGNC=25941	QPRT , XDH , TET2
Catabolism process	GO:0030433	ER-associated ubiquitin-dependent protein catabolic process	HGNC=27735 HGNC=14236	RNF175 , DERL3
Catabolism process	GO:0043632	modification-dependent macromolecule catabolic process	HGNC=17035 HGNC=17351	EXOSC8 , PRPF18
Catabolism process	GO:0046348	amino sugar catabolic process	HGNC=8524	OVGP1
Catabolism process	GO:1901565	organonitrogen compound catabolic process	HGNC=9755 HGNC=12805 HGNC=8524 HGNC=25941	QPRT1 , XDH , OVGP1 , TET2
Catabolism process	GO:0019439	aromatic compound catabolic process	HGNC=12805 HGNC=25941	XDH , TET2
Catabolism process	GO:0006022	aminoglycan metabolic process	HGNC=8524	OVGP1
Catabolism process	GO:0030166	proteoglycan biosynthetic process	HGNC=19721	CANT1
Catabolism process	GO:0044282	small molecule catabolic process	HGNC=14522 HGNC=4118	MIOX , GALK1
Catabolism process	GO:1901361	organic cyclic compound catabolic process	HGNC=9755 HGNC=12805 HGNC=25941	QPRT , XDH , TET2
Catabolism process	GO:0031146	SCF-dependent proteasomal ubiquitin-dependent protein catabolic process	HGNC=14150	FBXL16
Cell Movement	GO:0007018	microtubule-based movement	HGNC=14566 HGNC=21202	KIF13A , KIF6
Cell Movement	GO:0030336	negative regulation of cell migration	HGNC=21165	RAP2C
Cell Movement	GO:0008045	motor neuron axon guidance	HGNC=9802	RAC2
Cell Movement	GO:0072676	lymphocyte migration	HGNC=16642	CXCL16
Cell Movement	GO:0007098	centrosome cycle	HGNC=20163 HGNC=30532	HAUS4 , HAUS8
Cell Movement	GO:0001578	microtubule bundle formation	HGNC=24164	TPPP
Cell Movement	GO:0007052	mitotic spindle organization	HGNC=24502	WDR62
Cell Movement	GO:0007015	actin filament organization	HGNC=9802 HGNC=2255 HGNC=16932 HGNC=2254	RAC2 , CORO2A , NEBL , CORO1C
Cell Movement	GO:0060294	cilium movement involved in cell motility	HGNC=21057	RSPH9
Cell Movement	GO:0000132	establishment of mitotic spindle orientation	HGNC=978	BBCIP
Cell Movement	GO:0060326	cell chemotaxis	HGNC=1060 HGNC=1609	CXCR5 , CCR8
Cell Movement	GO:0031122	cytoplasmic microtubule organization	HGNC=18600	TUBGCP5
Cell Movement	GO:0030042	actin filament depolymerization	HGNC=243	ADD1
Metabolic Process	GO:0070989	oxidative demethylation	HGNC=25941	TET2
Metabolic Process	GO:1901568	fatty acid derivative metabolic process	HGNC=26222	FAR1
Metabolic Process	GO:0009112	nucleobase metabolic process	HGNC=12805	XDH
Metabolic Process	GO:0008610	lipid biosynthetic process	HGNC=20715	MID1IP1
Metabolic Process	GO:0009143	nucleoside triphosphate catabolic process	HGNC=3356	ENPP1
Metabolic Process	GO:0046890	regulation of lipid biosynthetic process	HGNC=20715	MID1IP1
Metabolic Process	GO:0006208	pyrimidine nucleobase catabolic process	HGNC=3013	DPYS
Metabolic Process	GO:0046034	ATP metabolic process	HGNC=3356	ENPP1
Metabolic Process	GO:0006066	alcohol metabolic process	HGNC=14522	MIOX
Metabolic Process	GO:0009247	glycolipid biosynthetic process	HGNC=24275	SCCPDH
Metabolic Process	GO:0006631	fatty acid metabolic process	HGNC=2329	CPT1B
Metabolic Process	GO:0045761	regulation of adenylate cyclase activity	HGNC=13841 HGNC=240 HGNC=277 HGNC=237	ADGRG6 , ADCY9 , ADRA1A , ADCY6
Protein Regulation	GO:0018205	peptidyl-lysine modification	HGNC=20944	SENP6
Protein Regulation	GO:0045859	regulation of protein kinase activity	HGNC=9309	PPP2R5A
Protein Regulation	GO:0007254	JNK cascade	HGNC=12033	TRAF3
Protein Regulation	GO:0001522	pseudouridine synthesis	HGNC=16060	TRUB1
Protein Regulation	GO:0070475	rRNA base methylation	HGNC=16385	NSUM5
Protein Regulation	GO:0016567	protein ubiquitination	HGNC=30688 HGNC=591	CAND1 , BIRC3
Protein Regulation	GO:0006470	protein dephosphorylation	HGNC=9671 HGNC=9309	PTPRG , PPP2R5A
Protein Regulation	GO:0000186	activation of MAPKK activity	HGNC=6857	MAP3K5
Protein Regulation	GO:0070534	protein K63-linked ubiquitination	HGNC=12033	TRAF3
Protein Regulation	GO:0016485	protein processing	HGNC=591 HGNC=17091	BIRC3
Protein Regulation	GO:0006497	protein lipidation	HGNC=25072	WDR45B
Cellular Homeostasis	GO:0050803	regulation of synapse structure or activity	HGNC=20151	SLC17A8
Cellular Homeostasis	GO:0055067	monovalent inorganic cation homeostasis	HGNC=10911	SLC12A2
Cellular Homeostasis	GO:0051208	sequestering of calcium ion	HGNC=9009	PKD2
Cellular Homeostasis	GO:0051235	maintenance of location	HGNC=3976 HGNC=11214 HGNC=1908	FTH1 , SPAG4 , VPS13A
Cellular Homeostasis	GO:0019725	cellular homeostasis	HGNC=10911	SLC12A2
Cellular Homeostasis	GO:0006882	cellular zinc ion homeostasis	HGNC=4927	SLC39A7
Cellular Homeostasis	GO:0050801	ion homeostasis	HGNC=14544	WNK4
Cellular Homeostasis	GO:0051453	regulation of intracellular pH	HGNC=16438	SLC4A11
Cellular Homeostasis	GO:0042391	regulation of membrane potential	HGNC=13814 HGNC=1955 HGNC=4328 HGNC=31715	KCNK15 , CHRNA1 , GLRA3 , GLRA4
Cellular Homeostasis	GO:0008361	regulation of cell size	HGNC=10911	SLC12A2
Other	GO:0044262	cellular carbohydrate metabolic process	HGNC=4696 HGNC=14522	GUSB , MIOX
Other	GO:0009108	coenzyme biosynthetic process	HGNC=9755	QPRT
Other	GO:0017144	drug metabolic process	HGNC=9755 HGNC=8524 HGNC=20243	QPRT , OVGP1 , CYP2W1
Other	GO:0007163	establishment or maintenance of cell polarity	HGNC=9802	RAC2
Other	GO:0006801	superoxide metabolic process	HGNC=3062 HGNC=7890 HGNC=1613	DUOX1 , NOX3 , CCS
Other	GO:0001525	angiogenesis	HGNC=22989	COL22A1
Other	GO:0045777	positive regulation of blood pressure	HGNC=277	ADRA1A
Other	GO:0008284	positive regulation of cell proliferation	HGNC=5967	Il11RA
Other	GO:0006457	protein folding	HGNC=28115	TBCEL
Detection of Stimulus	GO:0006298	mismatch repair	HGNC=7527	MUTYH
Detection of Stimulus	GO:0006284	base-excision repair	HGNC=7527 HGNC=587	MUTYH , APEX1
Detection of Stimulus	GO:0042060	wound healing	HGNC=3052	DSP
Detection of Stimulus	GO:0009612	response to mechanical stimulus	HGNC=9009 HGNC=18053 HGNC=9012 HGNC=9015	PKD2 , PKD1L1 , PKD2L2 , PKDREJ
Detection of Stimulus	GO:0009314	response to radiation	HGNC=6990	MECP2
Detection of Stimulus	GO:0009411	response to UV	HGNC=2718	DDB2
Detection of Stimulus	GO:0032675	regulation of interleukin-6 production	HGNC=15632	**TLR8**
Regulation of Cell Division	GO:0000086	G2/M transition of mitotic cell cycle	HGNC=7652	NBN
Regulation of Cell Division	GO:0006281	DNA repair	HGNC=2718	DDB2
Regulation of Cell Division	GO:0044773	mitotic DNA damage checkpoint	HGNC=7652	NBN
Regulation of Cell Division	GO:0010564	regulation of cell cycle process	HGNC=20944	SENP6
Regulation of Cell Division	GO:0000724	double-strand break repair via homologous recombination	HGNC=7652	NBN
Regulation of Cell Division	GO:0007134	meiotic telophase I	HGNC=29814 HGNC=27729	MUS81 , CTXN3
Regulation of Cell Division	GO:0051302	regulation of cell division	HGNC=26624	KDF1
Regulation of Cell Division	GO:0061640	cytoskeleton-dependent cytokinesis	HGNC=25589	SEPTIN11
Enzyme Activity	GO:0007202	activation of phospholipase C activity	HGNC=3538 HGNC=277	F2RL1 , ADRA1A
Enzyme Activity	GO:0043547	positive regulation of GTPase activity	HGNC=25418 HGNC=17791 HGNC=20310 HGNC=24715	RUNDC1 , TBC1D8 , GRTP1 , TBC1D8B
Enzyme Activity	GO:0010951	negative regulation of endopeptidase activity	HGNC=15995 HGNC=6383 HGNC=8584 HGNC=8941 HGNC=11345	SERPINA9 , KNG1 , SERPINB2 , RECK
Enzyme Activity	GO:0043433	negative regulation of sequence-specific DNA binding transcription factor activity	HGNC=12033	TRAF3
Apoptotic Process	GO:0051402	neuron apoptotic process	HGNC=7808	NGF
Apoptotic Process	GO:0043066	negative regulation of apoptotic process	HGNC=7808	NGF
Apoptotic Process	GO:0097193	intrinsic apoptotic signaling pathway	HGNC=18165	STYXL1
Apoptotic Process	GO:0042771	intrinsic apoptotic signaling pathway in response to DNA damage by p53 class mediator	HGNC=25722	AEN

TABLE 5

TLR8 Forward 5’- CAGAAACATGGAAAACATG TTCCTTCA GTCGTCAATGC-3’	Sigma-Aldrich	This paper
TLR8-2 Reverse 5’-CACATGCCAGACACCAG TGCTGTCATAACC ATGGTGGTGATAAAGAACG-3’	Sigma-Aldrich	This paper
TLR8-2 Forward 5’CGTTCTTT ATCACCACCATGGTTATGACAGCACTGGTGTCTGGCATGTG- 3’	Sigma-Aldrich	This paper
TLR2-TM Reverse 5’-CCTAGGACTTTATCGCAGCTC TCAGATTTACCCAAAATCC-3’	Sigma-Aldrich	This paper
PCR1 CRISPR screen: Fwd 5’-AGGGCCTATTTCCCATGATTCCTT-3’	Sigma-Aldrich	This paper
Rev 5’-TCAAAAAAGCACCGACTCG-3’	Sigma-Aldrich	This paper
Illumina amplification PCR Fwd 5’-AATGATACGGCGACCACCGAGATCTACACTCTCTTGTGGAAAGGACGAGGTACCG-3’	Sigma-Aldrich	This paper
Illumina amplification PCR Rev 5’-CAAGCAGAAGACGGCATACGAGAT [TCACTGT]GTGACTGGAGTTCAGACG TGTGCTCTTCCGATCTATTTTAACTTGCTATTTC TAGCTCTAAAAC-3’	Sigma-Aldrich	This paper
CAAUUAAUAUAGAUCGUUU<br>CUGGGAUG UUUGGUUAUA<br>CUAUCAACUUGGGUAUUAA<br>GUCUUGACUGAAAAUGAUU	Accell SMARTpool siRNA for Human TLR8	E-004715-00-0010
GCAAUAACUACGUUUUCUA<br>UUGUGACCGCAAUGGUAU<br>UUCUCAUCUCACAAAAUUG<br>UCUUUAUGUCACUAGUUAU	Accell SMARTpool siRNA for Human TLR2	E-005120-00-0010
GCAGCAUAUAUAAUUCAUG<br>UCACUAUGCUCGAUCUUUC<br>UUGGAUGUAGGAUUUAAC<br>CUGUUAGCCAUGAAGUUGC	Accell SMARTpool siRNA for Human TLR3	E-007745-00-0010
CUAGCUUUCUUAAAUCUUA<br>CUCUCUACCUUAAUAUUGA<br>UUCUGGACUAUCAAGUUUA<br>CCUAUAAGCUAAUAUCAUA	Accell SMARTpool siRNA for Human TLR4	E-008088-00-0010
CUAUCGUGCAUCUAUGAAU<br>CUGUGAUGCUGUGUGGUUU<br>CUAUGAUGCUUUUAUUGUG<br>GUAUCAGCGUCUAAUAUCA	Accell SMARTpool siRNA for Human TLR7	E-004714-00-0010
GCCACAACUUCAGCUUCGU<br>CUUGGAUCUGUCACGGAAC<br>CCUUCGUGGUCUUCGACAA<br>CCUGCAAAUACUAGAUGUA	Accell SMARTpool siRNA for Human TLR9	E-004066-00-0010
CCUUCGUCCUGCAUCACUU<br>CGUUCAGGUCGAAAGCUUC<br>GGCUUAUCCAGAAUCAGAU<br>CCAGCAGUCCUAUGAGUUU	Accell SMARTpool siRNA for Human NOD1	E-004398-00-0010
GCCACAUGCAAGAAGUAUA<br>CGAGCAAUUGCAGAAGUUA<br>GCUUUAGGAUGUACAGUUA<br>CUGUUAACCUUUGAUGGCU	Accell SMARTpool siRNA for Human NOD2	E-003464-00-0010
GUAUAAUCAUGGAUCGCUU<br>GGAUUAGCGACAAAUUUAA<br>CUGACAUACAGAUUUUCUA<br>UCUUGAUGCGUCAGUGAUA	Accell SMARTpool siRNA for Human RIG-1, DDX58	E-012511-00-0010
UGGUUUACAUGUCGACUAA<br>UGGUUUACAUGUUUUCUGA<br>UGGUUUACAUGUUUUCCUA<br>UGGUUUACAUGUUGUGUGA.	Accell SMARTpool siRNA for Human non-targeting control	D-001910-10-20
