# Supplementary material for: Phagosomal RNA sensing through TLR8 controls susceptibility to tuberculosis
Source: Cell Rep. Author manuscript; Available in PMC 2025 Nov 18. (PMC7618372; doi:10.1016/j.celrep.2025.115657)
Supplement: Supplementary Material [file EMS209620-supplement-Supplementary_Material.zip › 1-s2.0-S2211124725004280-mmc4.pdf]

Cell Reports

Article

Phagosomal RNA sensing through TLR8 controls susceptibility to tuberculosis

Graphical abstract

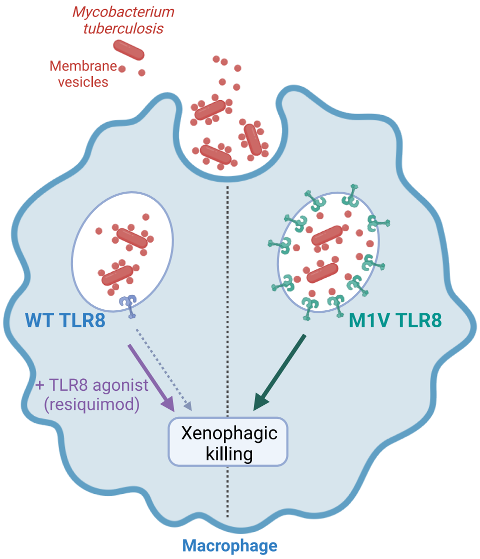

Authors

Charlotte Maserumule, Charlotte Passemar, Olivia S.H. Oh, ..., Rafael Prados-Rosales, Paul A. MacAry, R. Andres Floto

Correspondence

micpam@nus.edu.sg (P.A.M.), arf27@cam.ac.uk (R.A.F.)

In brief

Maserumule et al. show that TLR8 senses mycobacterial RNA in macrophage phagosomes, triggering xenophagy to clear *Mycobacterium tuberculosis* (*Mtb*). TLR8 polymorphisms affect tuberculosis susceptibility, and resiquimod, a TLR8 agonist, enhances clearance of drug-susceptible and multidrug-resistant *Mtb* *in vitro* and *in vivo*, offering the potential for therapeutic repurposing.

Highlights

- CRISPR screening implicates TLR8 as a controller of intracellular *Mtb* replication
- TLR8 detects RNA-rich *Mtb* membrane vesicles in phagosomes, triggering xenophagy
- The TLR8 M1V variant boosts *Mtb* killing via preferential trafficking to phagosome
- An FDA-approved TLR8 agonist improves Mtb clearance, suggesting clinical utility

Maserumule et al., 2025, Cell Reports *44*, 115657
May 27, 2025 © 2025 The Authors. Published by Elsevier Inc.
https://doi.org/10.1016/j.celrep.2025.115657

Cell Reports

CellPress
OPEN ACCESS

Article

Phagosomal RNA sensing through TLR8 controls susceptibility to tuberculosis

Charlotte Maserumule,[1,13] Charlotte Passemar,[1,2,13] Olivia S.H. Oh,[3] Kriztina Hegyi,[1,14] Karen Brown,[1,2,4] Aaron Weimann,[1,2,5] Adam Dinan,[1,2,5] Sonia Davila,[6,7] Catherine Klapholz,[1] Josephine Bryant,[1,5] Deepshikha Verma,[8] Jacob Gadwa,[8] Shivankari Krishnananthasivam,[3] Kridakorn Vongtongsalee,[8] Edward Kendall,[8] Andres Trelles,[8] Martin L. Hibberd,[6,9] Joaquín Sanz,[10] Jorge Bertol,[10] Lucia Vázquez-Iniesta,[12] Kaliappan Andi,[11] S. Siva Kumar,[11] Diane Ordway,[8] Rafael Prados-Rosales,[12] Paul A. MacAry,[3,*] and R. Andres Floto[1,2,4,5,15,*]

[1]Molecular Immunity Unit, University of Cambridge Department of Medicine, MRC-Laboratory of Molecular Biology, Cambridge, UK
[2]Victor Philip Dahdaleh Heart & Lung Research Institute, University of Cambridge, Cambridge, UK
[3]Department of Microbiology, The Yong Loo Lin School of Medicine, National University of Singapore, Singapore, Singapore
[4]Cambridge Centre for Lung Infection, Royal Papworth Hospital, Cambridge, UK
[5]Cambridge Centre for AI in Medicine, University of Cambridge, Cambridge, UK
[6]Infectious Disease Group, Genome Institute of Singapore, Singapore, Singapore
[7]SingHealth Duke-NUS Institute of Precision Medicine, SingHealth Duke-NUS Genomic, Medicine Centre, Cardiovascular and Metabolic Disorder Program, Duke-NUS Medical, School, Singapore, Singapore
[8]Mycobacteria Research Laboratories, Department of Microbiology, Immunology and Pathology, Colorado State University, Fort Collins, CO, USA
[9]London School of Hygiene and Tropical Medicine, London, UK
[10]Institute for Bio-computation and Physics of Complex Systems BIFI, Department of Theoretical Physics, University of Zaragoza, Zaragoza, Spain
[11]ICMR-National Institute for Research in Tuberculosis, Chennai, India
[12]Department of Preventive Medicine, Public Health and Microbiology, School of Medicine, Universidad Autónoma de Madrid, Madrid, Spain
[13]These authors contributed equally
[14]Deceased
[15]Lead contact
*Correspondence: micpam@nus.edu.sg (P.A.M.), arf27@cam.ac.uk (R.A.F.)
https://doi.org/10.1016/j.celrep.2025.115657

SUMMARY

Genetic determinants of susceptibility to *Mycobacterium tuberculosis* (*Mtb*) remain poorly understood but could provide insights into critical pathways involved in infection, informing host-directed therapies and enabling risk stratification at individual and population levels. Through a genome-wide forward genetic screen, we identify Toll-like receptor 8 (TLR8) as a key regulator of intracellular killing of *Mtb*. Pharmacological TLR8 activation enhances the killing of phylogenetically diverse clinical isolates of drug-susceptible and multidrug-resistant *Mtb* by macrophages and during *in vivo* infection in mice. TLR8 is activated by phagosomal mycobacterial RNA released by extracellular membrane vesicles and enhances xenophagy-dependent *Mtb* killing. We find that the TLR8 variant M1V, common in Far Eastern populations, enhances intracellular killing of *Mtb* through preferential signal-dependent trafficking to phagosomes. TLR8 signaling may, therefore, both regulate susceptibility to tuberculosis and provide novel drug targets.

INTRODUCTION

Tuberculosis (TB), a disease caused by mycobacteria of the *Mycobacterium tuberculosis* (*Mtb*) complex (MTBC), remains a major global threat to human health, with an estimated third of the world's population at one time exposed,[1] over 1.3 million deaths recorded per year,[2] and growing rates seen for multidrug-resistant (MDR) and extensively drug-resistant (XDR)[3,4] infections. Increasing antibiotic resistance and long treatment durations have motivated the search for druggable innate immune pathways that could be pharmacologically stimulated to deliver host-directed therapy.[5,6]

The factors that control cell-autonomous immunity to *Mtb*, however, remain partially understood,[1,6] despite forward genetic screens mostly using other mycobacterial species in macrophage or zebrafish infection models,[7–9] genome-wide association studies in several ethnic populations,[10–13] and genetic analyses of primary immunodeficiencies associated with mycobacterial susceptibility.[14]

Since the initial interactions of *Mtb* with macrophages appear critical in determining the outcome of human infection,[6] we set

© 2025 The Authors. Published by Elsevier Inc.
This is an open access article under the CC BY license (http://creativecommons.org/licenses/by/4.0/).

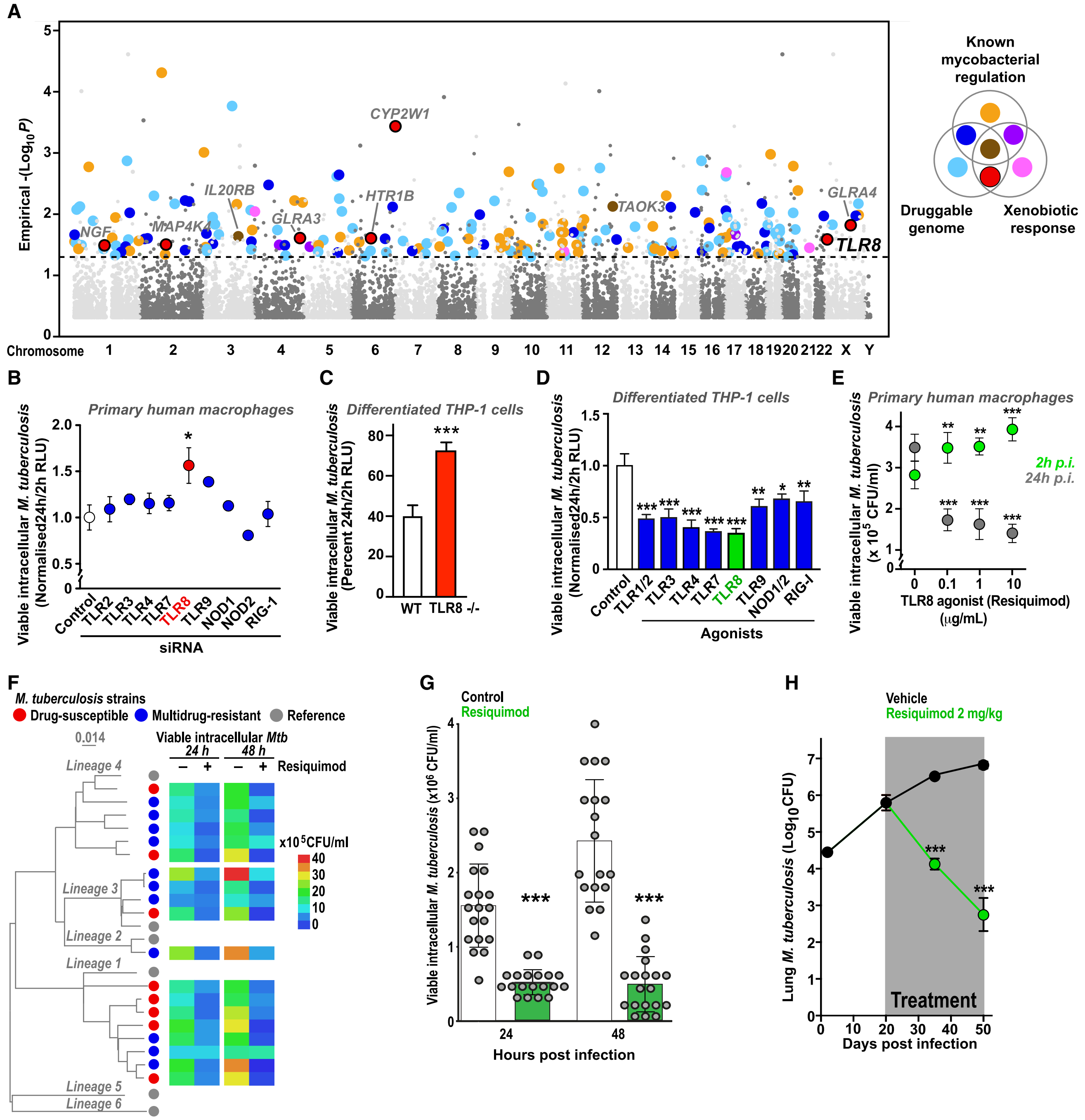

Figure 1. Forward genetic screening reveals TLR8 as a druggable pathway in *M. tuberculosis* infection

(A) Genome-wide CRISPR screen in THP1 macrophages infected with GFP-expressing *M. tuberculosis* (*Mtb*) Δ*leu*D Δ*pan*CD (BleuPan) performed on three separate occasions. Cells with high *Mtb*-associated fluorescence at 24 h were fluorescence-activated cell-sorted and their gRNA representation compared to the bulk infected population at a gene level. A Manhattan plot of significant hits is shown, color annotated based on whether genes are known regulators of mycobacterial infection, involved in the xenobiotic response, part of the druggable genome, or combinations of these. Novel druggable genes involved in the xenobiotic response (red) include TLR8.

(B) Effect on intracellular killing of luminescent *Mtb* (H37Rv) by primary human macrophages following knockdown (using pooled siRNA) of a panel of pattern recognition receptors (PRRs).

(C) Intracellular killing of luminescent *Mtb* (Δ*leu*D Δ*pan*CD [BleuPan]) by wild-type and TLR8-knockout THP1 macrophages.

(D) Effect of agonists targeting different PRRs (blue), including resiquimod targeting TLR8 (green), on intracellular killing of luminescent *Mtb* (H37Rv) by THP1 macrophages.

(legend continued on next page)

out to discover new druggable pathways in macrophages that control the intracellular survival of *Mtb*.

RESULTS

Forward genetic screening identifies TLR8 as a potential therapeutic target in *Mtb* infection

To identify novel host restriction factors, we exposed THP1 macrophages, transduced with a genome-wide CRISPR library,[15] to a GFP-expressing auxotrophic *Mtb* strain with preserved virulence.[16] We then selected cells with excess *Mtb*-associated fluorescence at 24 h post-infection (using fluorescence-activated cell sorting [FACS]), amplified and sequenced guide RNA (gRNA) templates from extracted DNA, and then detected targeted genes that were statistically over-represented in the sorted compared to the bulk populations (Figure S1).

Our screen identified many plausible hits across a range of cellular processes, including multiple genes known to influence *Mtb* growth within macrophages[17–25] (Figures 1A and S1; Tables S1, S2, S3, and S4). We selected novel hits within the druggable genome[26] involved in the xenobiotic response for further analysis. We focused on Toll-like receptor 8 (TLR8), a known endosomal sensor of single-stranded RNA (ssRNA) and established mediator of antiviral immunity[27] that has previously been implicated in host defense against mycobacteria, including *Mtb*.[28–30] We compared the relative contribution of a panel of pattern recognition receptors (PRRs) to *Mtb* infection of primary human macrophages using small interfering RNA (siRNA) knockdown (as done previously[31]) and found that TLR8 silencing led to the greatest increase in viable intracellular bacteria (Figure 1B), results supported by our findings of increased intracellular survival of *Mtb* in CRISPR-mediated TLR8-knockout THP1 macrophages (Figures 1C and S2).

We next examined the impact of pharmacological stimulation of TLR8 and other PRRs and found that the intracellular killing of *Mtb* by THP1 macrophages was most enhanced by treatment with the TLR8 agonist resiquimod[32] (Figure 1D), which also increased mycobacterial uptake (Figure 1E) but had no direct effect on *Mtb* in liquid culture or cell viability (Figure S2). Together, our results indicate that TLR8 plays a critical role in the macrophage killing of *Mtb* and that this pathway is sub-maximally activated under baseline conditions, thus identifying TLR8 as a potential target for host-directed therapy.

Resiquimod is an imidazoquinoline with antiviral and antitumor activity in preclinical animal models[33,34] and clinical activity as an adjuvant to vaccines[35] and cancer immunotherapy[36] and is currently licensed by the European Medicines Agency for the treatment of cutaneous T cell lymphomas.[37,38] Resiquimod can activate both human intracellular receptors for ssRNA, TLR8 and TLR7.[39] Since TLR7 is not expressed in human macrophages[40] (Figure S3), resiquimod activity is abolished in *Mtb*-infected human $TLR8^{-/-}$ macrophages (Figure S2) but preserved in $TLR7^{-/-}$ cells (Figure S3). In contrast, TLR8 in mice is a pseudogene influencing TLR7 expression.[41] Murine macrophages, therefore, only express TLR7, which is thus orthologous to human TLR8. As expected, the activity of resiquimod on *Mtb*-infected mouse bone marrow-derived macrophages is mediated exclusively by this receptor (Figure S2).

Resiquimod treatment of THP1 macrophages infected with a phylogenetically diverse collection of drug-susceptible and MDR *Mtb* (MDR-TB) clinical isolates resulted in profound reductions in viable intracellular bacteria at 24 and 48 h post-infection (Figures 1F, 1G, and S4). Resiquimod treatment of mice infected via aerosol with MDR-TB isolates led to almost a three log-fold reduction in lung colony-forming units and reduced inflammatory lung damage (Figures 1H and 1I)—a greater effect than that seen with imiquimod (when both were compared in separate experiments) (Figure S5)—suggesting a potential role for resiquimod and related compounds as host-directed therapy for TB.

TLR8 detects phagosomal RNA released by *Mtb* through extracellular membrane vesicles

We next explored the mechanism of action of TLR8 during *Mtb* infection. Using a surface-expressed TLR8-TLR2 chimeric receptor[42] stably transfected in HEK293 cells, we showed that TLR8 can be activated by a wide range of slow- and rapid-growing mycobacterial species (Figure S6) and by purified mycobacterial RNA (but not DNA) and *M. bovis* BCG lysates and can be attenuated by RNase pre-treatment (Figure 2A). During THP1 macrophage infection, TLR8 activation within *Mtb*-containing phagosomes (monitored by recruitment of MyD88) was inhibited by co-incubation with RNase (Figure S6), indicating phagosomal sensing of mycobacteria-derived RNA.

Since *Mtb* is known to produce RNA-containing extracellular membrane vesicles (MVs),[43] particularly in the context of limiting iron availability[44] (as occurs in the phagosome[45]), we wondered whether MVs might trigger TLR8 signaling. We confirmed that *Mtb* (H37Rv and Δ*leu*D Δ*pan*CD [BleuPan]) and *M. bovis* BCG produced similar quantities and distributions of MVs (Figure S6) containing RNA-encoding proteins involved in a

(E) Effect of resiquimod (at a range of concentrations) on intracellular killing of *Mtb* (Δ*leu*D Δ*pan*CD [BleuPan]) by primary human macrophages from healthy volunteers.

(B–E) Data (mean ± SEM) shown from representative experiments at least three independent repeats, performed in at least triplicate (using primary macrophages, B and E, from at least three different healthy volunteers) (*$p < 0.05$, **$p < 0.01$, and ***$p < 0.001$; Student's t test).

(F and G) Resiquimod improves intracellular killing of clinical isolates of *Mtb*. THP1 macrophages were infected with a phylogenetically diverse collection of drug-susceptible (red) or multidrug-resistant (blue) *Mtb* clinical isolates and co-treated with resiquimod (10 μg/mL) or vehicle alone for 24 or 48 h, and viable intracellular mycobacteria were enumerated through cell-associated colony-forming units (CFUs/mL). Experiments were performed in at least triplicate.

(F) Maximum likelihood phylogenetic tree of all isolates tested constructed using RAxML, generated by mapping detected variable positions to *Mtb H37Rv* strain. Representatives from the main six *Mtb* lineages (gray) are included for genomic context. Scale bar indicates the number of substitutions per variable site.

(G) Viable intracellular *Mtb* (mean ± SD) recovered from THP1 macrophages infected with each of the clinical isolates in (F) at 24 and 48 h post-infection in the presence of resiquimod (green) or vehicle control (white). ***$p < 0.001$ (paired Student's t test).

(H) Resiquimod treatment (via once-daily intraperitoneal injection) of C57BL/6 mice infected with multidrug-resistant *Mtb* (TB5904) resulted in a significant reduction in lung bacterial counts. Data represents mean ± SEM CFUs from 5 mice per time point in each group. ***$p < 0.001$ (Student's t test).

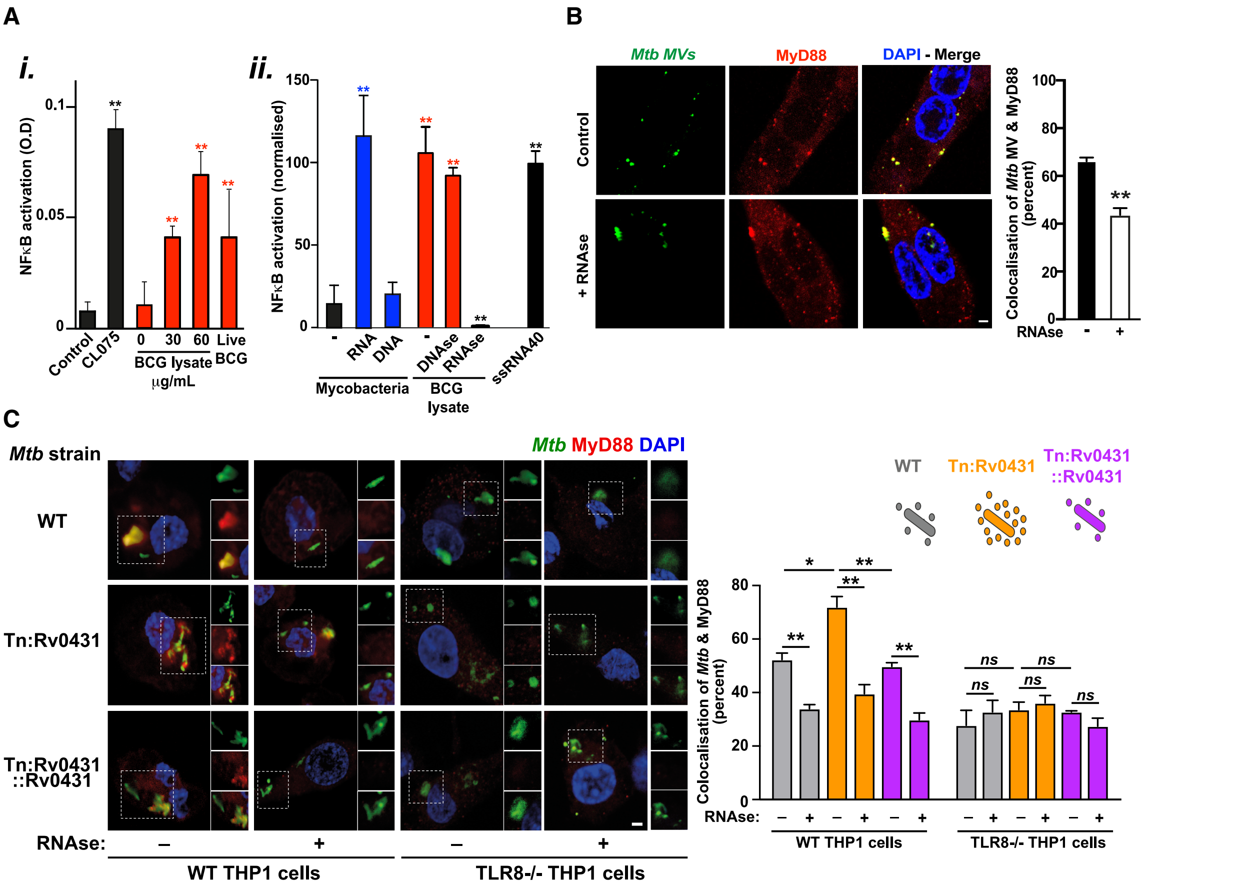

Figure 2. TLR8 senses phagosomal RNA released from *M. tuberculosis* in extracellular membrane vesicles

(A) TLR8-2 chimeric receptors (made by fusing the TLR8 extracellular domain to the transmembrane and cytosolic domains of TLR2) were stably surface expressed in HEK293T cells containing a nuclear factor κB (NF-κB) luciferase reporter. TLR8-dependent NF-κB signaling was assessed following (i) addition of *M. bovis* BCG lysates or live bacteria (red) or the TLR8 ligand CL075 (black) or (ii) mycobacterial RNA or DNA (blue), *M. bovis* BCG lysates (untreated or pre-treated with DNase or RNase, red), or ssRNA40 (black). Data (mean ± SEM) are representative of at least three independent experiments performed in at least triplicate. **p* < 0.05 and ***p* < 0.01 (Student's t test).

(B) Extracellular membrane vesicles isolated from *Mtb* H37Rv were CFSE labeled (green; *Mtb* MVs) and incubated with THP-1 macrophages for 24 h either with or without RNase A (100 μg/mL), immunostained for MyD88 (red), and imaged (and co-localization quantified) using confocal microscopy.

(C) THP-1 macrophages were infected with CFSE-labeled *Mtb* H37Rv; the transposon mutant *Tn:rv0431* (ΔvirR), which releases increased numbers of membrane vesicles; or the complemented mutant *Tn:rv0431+rv0431* (Δ*virR*::*virR*) in the presence or absence of RNase A for 24 h, immunostained for MyD88 (red), and imaged (and co-localization quantified) using confocal microscopy.

(B and C) Image scale bar: 2 μm. Data (mean ± SEM) are representative of at least three independent experiments preformed in at least triplicate (with a minimum of 50 *Mtb* phagosomes evaluated per replicate). **p* < 0.05 and ***p* < 0.01 (Student's t test).

range of functions (Figure S6). We obtained several lines of evidence in support of MVs triggering TLR8 signaling. We found that exposure of THP1 macrophages to isolated *Mtb* MVs at input ratios (MVs:cells) of 10:1 (considerably less than observed by electron microscopy during *in vitro* or *in vivo Mtb* infection[43]) stimulated MyD88 recruitment to endosomal compartments, which was attenuated by co-treatment with RNase (Figure 2B). An *Mtb* mutant that over-produces MVs, *Tn:Rv0431* (ΔvirR)[46,47] (Figure S6), was able to enhance TLR8 activation (in an RNase-inhibitable manner) during the infection of wild-type (WT), but not $TLR8^{-/-}$, THP1 macrophages (Figure 2C). Thus, the previously observed reduced intracellular survival of *Tn:Rv0431* within macrophages[47] is likely to be mediated through TLR8 activation by RNA-containing MVs.

TLR8 promotes intracellular mycobacteria clearance via xenophagy

We next examined how TLR8 signaling could enhance the intracellular killing of *Mtb.* Agonist stimulation of TLR8 led to enhanced phagosome-lysosome fusion (as monitored through *Mtb* co-localization with V-ATPase; Figure 3A) and increased numbers, acidification, and activity of lysosomes (Figure S7). Since lysosomal biogenesis and autophagy are known to be regulated by transcription factor EB (TFEB),[48,49] we examined whether TLR8 stimulation might activate these processes. As expected, resiquimod led to the rapid nuclear localization of TFEB in primary human macrophages and in a reconstituted heterologous expression system (Figure S7).

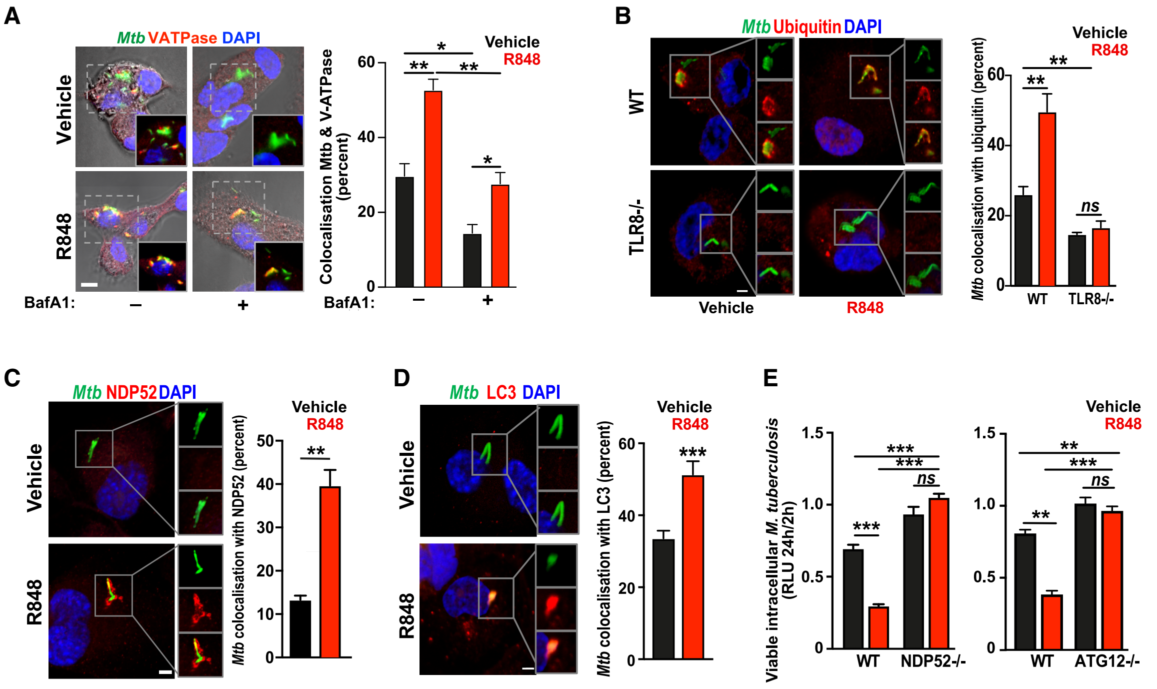

Figure 3. TLR8 enhances intracellular killing of mycobacteria through xenophagy

(A) TLR8 activation using resiquimod increases fusion of *Mtb*-containing phagosomes with lysosomes. THP-1 macrophages infected with GFP-labeled *Mtb* H37Rv (green) in the presence of bafilomycin A1 (BafA1) and/or resiquimod (or vehicle controls) for 2 h were immunostained for V-ATPase (red) and imaged (and co-localization quantified) using confocal microscopy (scale bar: 5 μm). Data (mean ± SEM) are representative of at least three independent experiments preformed in at least triplicate (with a minimum of 50 *Mtb* phagosomes evaluated per replicate). *$p < 0.05$ and **$p < 0.01$ (Student's t test).

(B) Wild-type (WT) or TLR8-knockout ($TLR8^{-/-}$) THP-1 macrophages were infected with GFP-labeled *Mtb* H37Rv (green) in the presence of resiquimod or vehicle control for 2 h, immunostained for ubiquitin (red), and imaged (and co-localization quantified) using confocal microscopy. Images and data (mean ± SEM) are representative of experiments performed in triplicate on at least three independent occasions with a minimum of 50 cells analyzed per replicate. **$p < 0.01$; ns, not significant (Student's t test). Scale bar: 2 μm.

(C and D) THP-1 macrophages were infected with GFP-labeled *Mtb* H37Rv (green) in the presence of resiquimod or vehicle control for 2 h, immunostained with (C) NDP52 or (D) LC3 (red), and imaged (and co-localization quantified) using confocal microscopy. Images and data (mean ± SEM) are representative of experiments performed in triplicate on at least two independent occasions with a minimum of 50 phagosomes analyzed per replicate. **$p < 0.01$ and ***$p < 0.001$ (Student's t test). Scale bar: 2 μm.

(E) WT, ATG12-knockout ($ATG12^{-/-}$), or NDP52-knockout ($NDP52^{-/-}$) THP-1 macrophages were infected with luminescent *Mtb* (Δ*leu*D Δ*pan*CD [BleuPan]) in the presence of resiquimod or vehicle control. Viable intracellular *Mtb* at 2 and 24 h was quantified using luminescence (relative light unit, RLU). Data (mean ± SEM) are representative of experiments performed in triplicate on at least three independent occasions. **$p < 0.01$ and ***$p < 0.001$ (Student's t test).

We then explored the role of autophagy in TLR8 effector functions and observed agonist-triggered increases in the number and acidification of autophagosomes in bone marrow-derived macrophages from mRFP-GFP LC3 transgenic mice[50] (Figure S7); agonist-induced ubiquitination of *Mtb*-containing phagosomes in WT, but not $TLR8^{-/-}$ THP1 macrophages (Figures 3B and S7); agonist-stimulated recruitment of the autophagy adaptor NDP52 (Figure 3C) and of LC3 (Figure 3D) to *Mtb*-containing phagosomes; and inhibition of agonist-enhanced intracellular killing of *Mtb* in $NDP52^{-/-}$ and $ATG12^{-/-}$ THP1 macrophages (Figures 3E and S7). Our findings, therefore, indicate that TLR8 activation increases lysosomal activity and stimulates xenophagic clearance of intracellular *Mtb* (and not simply epiphenomenal activation of autophagy). Since we have implicated phagosomal ubiquitination and a dependence on NDP52, we conclude that TLR8 activates conventional autophagy rather than LC3-associated phagocytosis.

The M1V variant of TLR8 modifies intracellular receptor localization and boosts *Mtb* clearance

Given the profound effects of TLR8 activation on *in vitro* and *in vivo Mtb* infections, we wondered whether naturally occurring genetic polymorphisms in *TLR8* might influence host susceptibility to *Mtb* infection through altered receptor signaling. We focused on the M1V variant (rs3764880) that leads to an alternative start codon usage and, consequently, an altered signal peptide (Figures 4A–4C). The M1V variant has been implicated in significant protection from pulmonary TB in our previous population genetic studies[41] and is found at variable allele frequencies in different ethnic groups, most abundantly in Far Eastern populations[51] (Figure 4B). Primary macrophages from healthy volunteers homozygous or hemizygous for the M1V TLR8 variant demonstrated enhanced killing of *Mtb* and *M. bovis* BCG (Figure 4D), increased inflammatory cytokine release (Figures 4E and S8), and more mature mycobacteria-containing

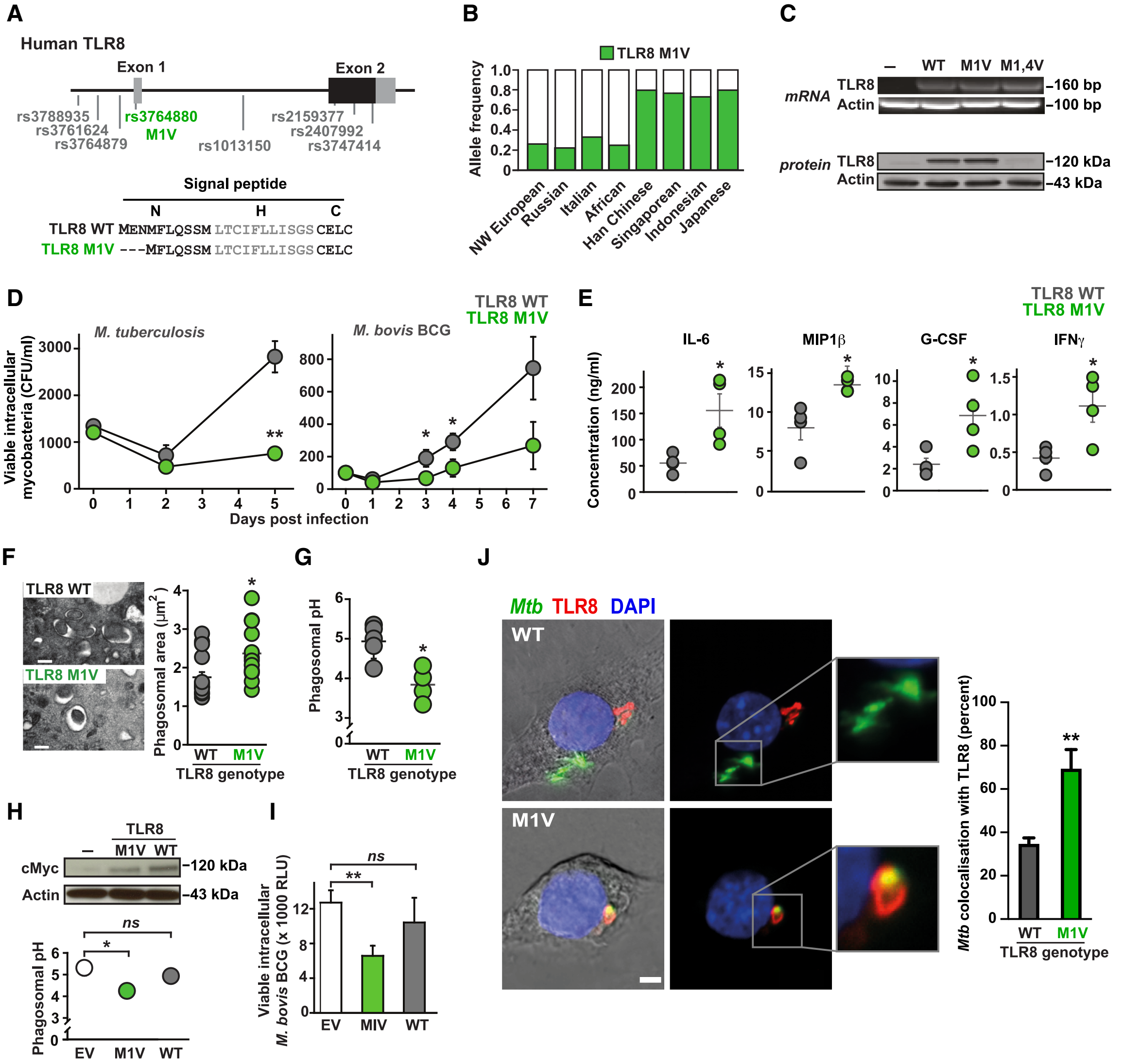

Figure 4. The M1V variant of TLR8 alters intracellular receptor localization and enhances intracellular killing of *M. tuberculosis*

(A) Top: haplotype block of single-nucleotide polymorphisms (SNPs) in human TLR8 associated with protection from pulmonary tuberculosis[43] with one non-synonymous coding polymorphism (rs3764880 M1V, green). Bottom: predicted signal peptide sequences of WT and M1V TLR8 suggest that the M1V polymorphism leads to alternative start codon usage (methionine at position 4).

(B) Allele frequency of TLR8 (green) in different ethnic groups (data from NCBI SNP database and Davila et al.[41]).

(C) RT-PCR (top) and western blot (bottom) analysis of ancestral TLR8 (WT), the M1V variant (M1V), and a mutant TLR8 with both methionines (at positions 1 and 4) changed to valines (M1,4V).

(D–F) Primary human macrophages from healthy volunteers that are homozygous or hemizygous for the ancestral TLR8 (TLR8 WT, gray) or M1V variant (TLR8 M1V, green) (n = 5 for each genotype) were infected with either *Mtb* CDC1551 or *M. bovis* BCG. (D) Viable intracellular mycobacteria were enumerated by counting CFUs in cell lysates at indicated time points post-infection. (E) Secreted cytokines were measured in supernatants at 24 h post-infection. (F) Mycobacteria-containing phagosomes (by electron microscopy) within primary macrophages from M1V homo/hemizygotes were larger, indicating the probable formation of bactericidal phago-lysosomes. At least 150 phagosomes per donor were evaluated (with 5 donors per genotype) in 3 independent experiments. Data (mean ± SEM) are representative of experiments performed in at least triplicate. *$p < 0.05$, **$p < 0.01$, and ***$p < 0.001$ (Student's t test).

(G) Primary macrophages from M1V homo/hemizygotes were able to better acidify mycobacteria-containing phagosomes than macrophages from ancestral controls, measured by assessing fluorescent ratios of internalized heat-killed *Mtb* H37Rv labeled with both acid-quenchable (FITC) and pH-resistant (Alexa 633) fluorophores by flow cytometry. Data (mean ± SEM) are representative of experiments performed in at least triplicate using samples from n = 5 subjects for each genotype. *$p < 0.05$ (Student's t test).

(H and I) Primary human macrophages from healthy volunteers homo/hemizygous for ancestral TLR8 were transfected with either Myc-tagged TLR8 WT or M1V (or empty vector). Similar exogenous TLR8 expression was confirmed by western blot analysis using a c-Myc specific antibody. Macrophages transfected with the M1V variant demonstrated (H) greater acidification of mycobacteria-containing phagosomes and (I) improved killing of intracellular mycobacteria. Data (mean ± SEM) are representative of experiments performed in at least triplicate on three independent occasions (each using a separate donor). *$p < 0.05$ and **$p < 0.01$ (Student's t test).

(legend continued on next page)

phagosomes (as evidenced by their increased size [Figure 4F] and greater acidification [Figure 4G]) compared to ancestral (WT) TLR8 controls. The transduction of M1V, but not WT, TLR8 into primary macrophages from WT hemizygous or homozygous individuals significantly increased phagosomal acidification (Figure 4H) and enhanced intracellular mycobacterial killing (Figure 4I), supporting a direct effect of the M1V variant on host restriction of *Mtb*.

Since the intracellular localization of the related receptor TLR7 is controlled by N-terminal determinants,[52] we wondered whether the M1V polymorphism might favorably alter TLR8 trafficking within cells. Compared to the ancestral WT receptor, we found that the M1V variant showed improved co-localization with *Mtb*-containing phagosomes when expressed in RAW-264.7 mouse macrophages (Figure 4J) and has altered intracellular localization when heterologously expressed in HEK273 cells (Figure S8), which is dependent on its signal peptide, as WT and M1V TLR8-CD4 chimeric receptors are also differentially localized within cells (Figure S8). While the mechanism for this process remains unclear, changes to the signal peptide have been implicated in altered intracellular localization of other receptors (such as MC3R[53]).

DISCUSSION

In summary, we have identified TLR8 as an important mediator of cell-autonomous immunity against *Mtb* that acts by sensing mycobacterial RNA within macrophage phagosomes and stimulating xenophagic clearance. Our data suggest that TLR8 detects RNA-containing *Mtb* MVs released in response to iron starvation experienced within phagosomes,[44] a mechanism that may explain the observed impact of TLR8 on macrophage responses to other bacteria.[54–56] It is reasonable to imagine that TLR8 activation may sense viable bacteria (as previously suggested[57]) that are actively producing RNA-containing MVs since these are likely to be continuously and rapidly cleared or destroyed during *in vivo* infection.

We show that the TLR8 pathway is a potential therapeutic target during *Mtb* infection since it is sub-maximally activated physiologically and, when stimulated pharmacologically by resiquimod, enhances clearance of drug-susceptible and MDR-TB *in vitro* and *in vivo*. In addition to direct effects on macrophage clearance, TLR8 agonists may also improve adaptive immune responses during *Mtb* infection *in vivo* since they are recognized vaccine adjuvants.[35] Importantly, resiquimod, as a licensed drug, could be rapidly repurposed and clinically evaluated as a host-directed therapy for *Mtb.*

Finally, we demonstrate that the M1V variant that alters the signal peptide of TLR8 is preferentially trafficked to *Mtb*-containing phagosomes and promotes greater intracellular mycobacterial killing, potentially explaining its genetic association with protection from pulmonary TB[41] and its likely evolutionary selection and raising the possibility that polymorphisms in other genes may, singly or in combination, influence host susceptibility by regulating macrophage clearance of *Mtb.*

Limitations of this study

This study has a number of limitations that should be considered. Genome-wide CRISPR screen libraries, while powerful, are not fully efficient, which may lead to false negative hits, potentially missing critical genes involved in *Mtb* host-pathogen interactions. *In vitro* analysis of macrophage infection with *Mtb* may be influenced by culture growth conditions, which may not fully replicate the complex environment within the human host. Additionally, reliance on mouse models for TB research presents limitations, as mice do not form granulomas in the same way humans do. Moreover, the involvement of (and impact of resiquimod on) adaptive immunity, which modulates and enhances macrophage responses, may additionally contribute to the observed *in vivo* phenotypes.

RESOURCE AVAILABILITY

Lead contact

Requests for further information and resources should be directed to and will be fulfilled by the lead contact, Prof. R. Andres Floto (arf27@cam.ac.uk).

Materials availability

Plasmids generated in this study can be available upon request to the lead contact and the generation of a materials transfer agreement (MTA).

Data and code availability

- Single-cell RNA sequencing (RNA-seq) data have been deposited at GEO at GEO: GSE288494 and are publicly available as of the date of publication. CRISPR screen data have been deposited at EBI-ENA under accession number EBI-ENA: PRJEB62758.
- CRISPR screen code has been deposited at Zenodo : https://doi.org/10.5281/zenodo.14982932.
- Any additional information required to reanalyze the data reported in this paper is available from the lead contact upon request.

ACKNOWLEDGMENTS

We thank Dr. Ben Porebski (MRC LMB, UK) for help with CRISPR screen sequencing, Dr. Caetano Reis e Sousa (Crick Institute, London, UK) for TLR7 mouse bone marrow, Dr. David Rubinzstein for TFEB cell lines and bone marrow from mCherry-GFP LC3 transgenic mice, and Dr. Brian Robertson for help with *Mtb* transfections. Primary cell *in vitro* experiments were authorized by regional ethics approval REC 12/WA/0148. This work was supported by Wellcome Trust grants 107032AIA (R.A.F., C.P., K.H., K.B., A.W., and A.D.), 226602/Z/22/Z (R.A.F., C.P., A.W., and A.D.), and 10224/Z/15/Z (J. Bryant); the UK Cystic Fibrosis Trust (Innovation Hub grant 001 [R.A.F., C.P., K.H., K.B., A.W., and A.D.] and Strategic Research Centre grants 002 and 010 [R.A.F., D.O., and D.V.]); the NIHR Cambridge Biomedical Research Centre (R.A.F. and K.B.); the Cambridge Commonwealth Trust (C.M.); Botnar Foundation grant 6063 (R.A.F., C.P., K.H., K.B., A.W., and A.D.); NIH RO1AI162821 and Spanish MICINN contracts PID2019-110240RB-I00 and PID2022-136611OB-I00 (R.P.-R.); and the Bill and Melinda Gates Foundation (P.A.M., O.S.H.O., and S.K.).

(J) Mouse macrophage cells (RAW 264.7) transfected with either ancestral (WT, top) or M1V (M1V, bottom) human TLR8 tagged with c-Myc were infected with GFP-expressing *Mtb* (Δ*leu*D Δ*pan*CD [BleuPan]), immunostained, and imaged (and co-localization quantified) using confocal microscopy (TLR8: red, *Mtb*: green). Images and data (mean ± SEM) are representative of experiments performed in triplicate on at least three independent occasions with a minimum of 50 cells analyzed per replicate. $^{**}p < 0.01$ (Student's t test). Scale bar: 2 μm.

AUTHOR CONTRIBUTIONS

P.A.M. and R.A.F. conceived the project, designed the experiments, and wrote the manuscript; C.M., C.P., O.S.H.O., K.H., K.B., C.K., K.A., and S.S.K. performed the *in vitro* experiments; R.P.-R. assisted with the MV experiments; D.V., J.G., K.V., E.K., A.T., and D.O. performed the mouse infection experiments; S.D. and M.L.H. performed the population genetics analysis; A.W. and A.D. performed the CRISPR screening analyses; J. Bryant performed the *Mtb* phylogenetic analysis; and P.A.M. and R.A.F. provided supervisory support.

DECLARATION OF INTERESTS

The authors declare no competing interests.

STAR★METHODS

Detailed methods are provided in the online version of this paper and include the following:

- KEY RESOURCES TABLE
- EXPERIMENTAL MODEL AND STUDY PARTICIPANT DETAILS
  - Mycobacteria
  - Subjects details
  - Mammalian cell cultures
  - *In vivo* animal study
- METHOD DETAILS
  - Extracellular membrane vesicles
  - THP1 CRISPR knockout library
  - Individual CRISPR knockout cell lines
  - Plasmid constructs
  - Transfections
  - SiRNA experiments
  - Mycobacterial infections of macrophages
  - Cell death analysis
  - Confocal imaging
  - Cytokine analysis
  - Activation assays to determine TLR ligand
  - Phylogenetic analysis
  - Quantitation of lysosomal degradative capacity
  - Analysis of phagosomal pH and size
  - Western blotting
  - Mouse infection experiment
- QUANTIFICATION AND STASTITICAL ANALYSIS

SUPPLEMENTAL INFORMATION

Supplemental information can be found online at https://doi.org/10.1016/j.celrep.2025.115657.

Received: July 11, 2024
Revised: January 13, 2025
Accepted: April 15, 2025
Published: May 6, 2025

REFERENCES

1. Abel, L., Fellay, J., Haas, D.W., Schurr, E., Srikrishna, G., Urbanowski, M., Chaturvedi, N., Srinivasan, S., Johnson, D.H., and Bishai, W.R. (2018). Genetics of human susceptibility to active and latent tuberculosis: present knowledge and future perspectives. Lancet Infect. Dis. *18*, e64–e75. https://doi.org/10.1016/S1473-3099(17)30623-0.
2. World Health Organization (2021). Global Tuberculosis Report 2021. https://www.who.int/publications/digital/global-tuberculosis-report-2021.
3. Dheda, K., Gumbo, T., Maartens, G., Dooley, K.E., McNerney, R., Murray, M., Furin, J., Nardell, E.A., London, L., Lessem, E., et al. (2017). The epidemiology, pathogenesis, transmission, diagnosis, and management of multidrug-resistant, extensively drug-resistant, and incurable tuberculosis. Lancet Respir. Med. *5*, 291–360. https://doi.org/10.1016/S2213-2600(17)30079-6.
4. Castro, R.A.D., Borrell, S., and Gagneux, S. (2021). The within-host evolution of antimicrobial resistance in Mycobacterium tuberculosis. FEMS Microbiol. Rev. *45*, fuaa071. https://doi.org/10.1093/femsre/fuaa071.
5. Mehta, K., Spaink, H.P., Ottenhoff, T.H.M., van der Graaf, P.H., and van Hasselt, J.G.C. (2022). Host-directed therapies for tuberculosis: quantitative systems pharmacology approaches. Trends Pharmacol. Sci. *43*, 293–304. https://doi.org/10.1016/j.tips.2021.11.016.
6. Simmons, J.D., Stein, C.M., Seshadri, C., Campo, M., Alter, G., Fortune, S., Schurr, E., Wallis, R.S., Churchyard, G., Mayanja-Kizza, H., et al. (2018). Immunological mechanisms of human resistance to persistent Mycobacterium tuberculosis infection. Nat. Rev. Immunol. *18*, 575–589. https://doi.org/10.1038/s41577-018-0025-3.
7. Kumar, D., Nath, L., Kamal, M.A., Varshney, A., Jain, A., Singh, S., and Rao, K.V.S. (2010). Genome-wide analysis of the host intracellular network that regulates survival of Mycobacterium tuberculosis. Cell *140*, 731–743. https://doi.org/10.1016/j.cell.2010.02.012.
8. Tobin, D.M., Vary, J.C., Ray, J.P., Walsh, G.S., Dunstan, S.J., Bang, N.D., Hagge, D.A., Khadge, S., King, M.-C., Hawn, T.R., et al. (2010). The lta4h locus modulates susceptibility to mycobacterial infection in zebrafish and humans. Cell *140*, 717–730. https://doi.org/10.1016/j.cell.2010.02.013.
9. Lai, Y., Babunovic, G.H., Cui, L., Dedon, P.C., Doench, J.G., Fortune, S. M., and Lu, T.K. (2020). Illuminating Host-Mycobacterial Interactions with Genome-wide CRISPR Knockout and CRISPRi Screens. Cell Syst. *11*, 239–251.e7. https://doi.org/10.1016/j.cels.2020.08.010.
10. EBI-GWAS catalog https://www.ebi.ac.uk/gwas/efotraits/Orphanet_3389.
11. Ishigaki, K., Akiyama, M., Kanai, M., Takahashi, A., Kawakami, E., Sugishita, H., Sakaue, S., Matoba, N., Low, S.-K., Okada, Y., et al. (2020). Large-scale genome-wide association study in a Japanese population identifies novel susceptibility loci across different diseases. Nat. Genet. *52*, 669–679. https://doi.org/10.1038/s41588-020-0640-3.
12. Zheng, R., Li, Z., He, F., Liu, H., Chen, J., Chen, J., Xie, X., Zhou, J., Chen, H., Wu, X., et al. (2018). Genome-wide association study identifies two risk loci for tuberculosis in Han Chinese. Nat. Commun. *9*, 4072. https://doi.org/10.1038/s41467-018-06539-w.
13. Mahasirimongkol, S., Yanai, H., Mushiroda, T., Promphittayarat, W., Wattanapokayakit, S., Phromjai, J., Yuliwulandari, R., Wichukchinda, N., Yowang, A., Yamada, N., et al. (2012). Genome-wide association studies of tuberculosis in Asians identify distinct at-risk locus for young tuberculosis. J. Hum. Genet. *57*, 363–367. https://doi.org/10.1038/jhg.2012.35.
14. Boisson-Dupuis, S., Bustamante, J., El-Baghdadi, J., Camcioglu, Y., Parvaneh, N., El Azbaoui, S., Agader, A., Hassani, A., El Hafidi, N., Mrani, N.A., et al. (2015). Inherited and acquired immunodeficiencies underlying tuberculosis in childhood. Immunol. Rev. *264*, 103–120. https://doi.org/10.1111/imr.12272.
15. Hart, T., Chandrashekhar, M., Aregger, M., Steinhart, Z., Brown, K.R., MacLeod, G., Mis, M., Zimmermann, M., Fradet-Turcotte, A., Sun, S., et al. (2015). High-Resolution CRISPR Screens Reveal Fitness Genes and Genotype-Specific Cancer Liabilities. Cell *163*, 1515–1526. https://doi.org/10.1016/j.cell.2015.11.015.
16. Mouton, J.M., Heunis, T., Dippenaar, A., Gallant, J.L., Kleynhans, L., and Sampson, S.L. (2019). Comprehensive Characterization of the Attenuated Double Auxotroph Mycobacterium tuberculosisΔleuDΔpanCD as an Alternative to H37Rv. Front. Microbiol. *10*, 1922. https://doi.org/10.3389/fmicb.2019.01922.
17. Michelet, X., Tuli, A., Gan, H., Geadas, C., Sharma, M., Remold, H.G., and Brenner, M.B. (2018). Lysosome-Mediated Plasma Membrane Repair Is Dependent on the Small GTPase Arl8b and Determines Cell Death Type in Mycobacterium tuberculosis Infection. J. Immunol. *200*, 3160–3169. https://doi.org/10.4049/jimmunol.1700829.

CellPress
OPEN ACCESS

18. Holla, S., Prakhar, P., Singh, V., Karnam, A., Mukherjee, T., Mahadik, K., Parikh, P., Singh, A., Rajmani, R.S., Ramachandra, S.G., and Balaji, K.N. (2016). MUSASHI-Mediated Expression of JMJD3, a H3K27me3 Demethylase, Is Involved in Foamy Macrophage Generation during Mycobacterial Infection. PLoS Pathog. *12*, e1005814. https://doi.org/10.1371/journal.ppat.1005814.

19. Gan, H., Lee, J., Ren, F., Chen, M., Kornfeld, H., and Remold, H.G. (2008). Mycobacterium tuberculosis blocks crosslinking of annexin-1 and apoptotic envelope formation on infected macrophages to maintain virulence. Nat. Immunol. *9*, 1189–1197. https://doi.org/10.1038/ni.1654.

20. Dey, R.J., Dey, B., Zheng, Y., Cheung, L.S., Zhou, J., Sayre, D., Kumar, P., Guo, H., Lamichhane, G., Sintim, H.O., and Bishai, W.R. (2017). Inhibition of innate immune cytosolic surveillance by an M. tuberculosis phosphodiesterase. Nat. Chem. Biol. *13*, 210–217. https://doi.org/10.1038/nchembio.2254.

21. Kumar, M., Majumder, D., Mal, S., Chakraborty, S., Gupta, P., Jana, K., Gupta, U.D., Ghosh, Z., Kundu, M., and Basu, J. (2020). Activating transcription factor 3 modulates the macrophage immune response to Mycobacterium tuberculosis infection via reciprocal regulation of inflammatory genes and lipid body formation. Cell. Microbiol. *22*, e13142. https://doi.org/10.1111/cmi.13142.

22. Bai, X., Kim, S.-H., Azam, T., McGibney, M.T., Huang, H., Dinarello, C.A., and Chan, E.D. (2010). IL-32 is a host protective cytokine against Mycobacterium tuberculosis in differentiated THP-1 human macrophages. J. Immunol. *184*, 3830–3840. https://doi.org/10.4049/jimmunol.0901913.

23. Ordway, D., Henao-Tamayo, M., Orme, I.M., and Gonzalez-Juarrero, M. (2005). Foamy macrophages within lung granulomas of mice infected with Mycobacterium tuberculosis express molecules characteristic of dendritic cells and antiapoptotic markers of the TNF receptor-associated factor family. J. Immunol. *175*, 3873–3881. https://doi.org/10.4049/jimmunol.175.6.3873.

24. Roy, S., Guler, R., Parihar, S.P., Schmeier, S., Kaczkowski, B., Nishimura, H., Shin, J.W., Negishi, Y., Ozturk, M., Hurdayal, R., et al. (2015). Batf2/Irf1 induces inflammatory responses in classically activated macrophages, lipopolysaccharides, and mycobacterial infection. J. Immunol. *194*, 6035–6044. https://doi.org/10.4049/jimmunol.1402521.

25. Dutta, N.K., Mehra, S., Martinez, A.N., Alvarez, X., Renner, N.A., Morici, L. A., Pahar, B., Maclean, A.G., Lackner, A.A., and Kaushal, D. (2012). The stress-response factor SigH modulates the interaction between Mycobacterium tuberculosis and host phagocytes. PLoS One *7*, e28958. https://doi.org/10.1371/journal.pone.0028958.

26. Finan, C., Gaulton, A., Kruger, F.A., Lumbers, R.T., Shah, T., Engmann, J., Galver, L., Kelley, R., Karlsson, A., Santos, R., et al. (2017). The druggable genome and support for target identification and validation in drug development. Sci. Transl. Med. *9*, eaag1166. https://doi.org/10.1126/scitranslmed.aag1166.

27. Martínez-Espinoza, I., and Guerrero-Plata, A. (2022). The Relevance of TLR8 in Viral Infections. Pathogens *11*, 134. https://doi.org/10.3390/pathogens11020134.

28. Tang, J., Zhan, L., and Qin, C. (2016). Inhibition of TLR8 mediated signaling promotes BCG induced apoptosis in THP-1 cells. Microb. Pathog. *93*, 78–82. https://doi.org/10.1016/j.micpath.2015.11.028.

29. Tang, J., Sun, M., Shi, G., Xu, Y., Han, Y., Li, X., Dong, W., Zhan, L., and Qin, C. (2017). Toll-Like Receptor 8 Agonist Strengthens the Protective Efficacy of ESAT-6 Immunization to Mycobacterium tuberculosis Infection. Front. Immunol. *8*, 1972. https://doi.org/10.3389/fimmu.2017.01972.

30. Thada, S., Horvath, G.L., Müller, M.M., Dittrich, N., Conrad, M.L., Sur, S., Hussain, A., Pelka, K., Gaddam, S.L., Latz, E., et al. (2021). Interaction of TLR4 and TLR8 in the Innate Immune Response against Mycobacterium Tuberculosis. Int. J. Mol. Sci. *22*, 1560. https://doi.org/10.3390/ijms22041560.

31. Schiebler, M., Brown, K., Hegyi, K., Newton, S.M., Renna, M., Hepburn, L., Klapholz, C., Coulter, S., Obregón-Henao, A., Henao Tamayo, M., et al. (2015). Functional drug screening reveals anticonvulsants as enhancers of mTOR-independent autophagic killing of Mycobacterium tuberculosis through inositol depletion. EMBO Mol. Med. *7*, 127–139. https://doi.org/10.15252/emmm.201404137.

32. Engel, A.L., Holt, G.E., and Lu, H. (2011). The pharmacokinetics of Toll-like receptor agonists and the impact on the immune system. Expert Rev. Clin. Pharmacol. *4*, 275–289. https://doi.org/10.1586/ecp.11.5.

33. Bernstein, D.I., Harrison, C.J., Tomai, M.A., and Miller, R.L. (2001). Daily or weekly therapy with resiquimod (R-848) reduces genital recurrences in herpes simplex virus-infected guinea pigs during and after treatment. J. Infect. Dis. *183*, 844–849. https://doi.org/10.1086/319262.

34. Bernstein, D.I., Harrison, C.J., Tepe, E.R., Shahwan, A., and Miller, R.L. (1995). Effect of imiquimod as an adjuvant for immunotherapy of genital HSV in guinea-pigs. Vaccine *13*, 72–76. https://doi.org/10.1016/0264-410x(95)80014-5.

35. Vasilakos, J.P., and Tomai, M.A. (2013). The use of Toll-like receptor 7/8 agonists as vaccine adjuvants. Expert Rev. Vaccines *12*, 809–819. https://doi.org/10.1586/14760584.2013.811208.

36. Schön, M.P., and Schön, M. (2008). TLR7 and TLR8 as targets in cancer therapy. Oncogene *27*, 190–199. https://doi.org/10.1038/sj.onc.1210913.

37. Rook, A.H., Gelfand, J.M., Wysocka, M., Troxel, A.B., Benoit, B., Surber, C., Elenitsas, R., Buchanan, M.A., Leahy, D.S., Watanabe, R., et al. (2015). Topical resiquimod can induce disease regression and enhance T-cell effector functions in cutaneous T-cell lymphoma. Blood *126*, 1452–1461. https://doi.org/10.1182/blood-2015-02-630335.

38. Killock, D. (2015). Haematological cancer: Resiquimod—a topical CTCL therapy. Nat. Rev. Clin. Oncol. *12*, 563. https://doi.org/10.1038/nrclinonc.2015.142.

39. Jurk, M., Heil, F., Vollmer, J., Schetter, C., Krieg, A.M., Wagner, H., Lipford, G., and Bauer, S. (2002). Human TLR7 or TLR8 independently confer responsiveness to the antiviral compound R-848. Nat. Immunol. *3*, 499. https://doi.org/10.1038/ni0602-499.

40. Hornung, V., Rothenfusser, S., Britsch, S., Krug, A., Jahrsdörfer, B., Giese, T., Endres, S., and Hartmann, G. (2002). Quantitative expression of toll-like receptor 1-10 mRNA in cellular subsets of human peripheral blood mononuclear cells and sensitivity to CpG oligodeoxynucleotides. J. Immunol. *168*, 4531–4537. https://doi.org/10.4049/jimmunol.168.9.4531.

41. Davila, S., Hibberd, M.L., Hari Dass, R., Wong, H.E.E., Sahiratmadja, E., Bonnard, C., Alisjahbana, B., Szeszko, J.S., Balabanova, Y., Drobniewski, F., et al. (2008). Genetic association and expression studies indicate a role of toll-like receptor 8 in pulmonary tuberculosis. PLoS Genet. *4*, e1000218. https://doi.org/10.1371/journal.pgen.1000218.

42. Leifer, C.A., Brooks, J.C., Hoelzer, K., Lopez, J., Kennedy, M.N., Mazzoni, A., and Segal, D.M. (2006). Cytoplasmic targeting motifs control localization of toll-like receptor 9. J. Biol. Chem. *281*, 35585–35592. https://doi.org/10.1074/jbc.M607511200.

43. Prados-Rosales, R., Baena, A., Martinez, L.R., Luque-Garcia, J., Kalscheuer, R., Veeraraghavan, U., Camara, C., Nosanchuk, J.D., Besra, G.S., Chen, B., et al. (2011). Mycobacteria release active membrane vesicles that modulate immune responses in a TLR2-dependent manner in mice. J. Clin. Investig. *121*, 1471–1483. https://doi.org/10.1172/JCI44261.

44. Prados-Rosales, R., Weinrick, B.C., Piqué, D.G., Jacobs, W.R., Casadevall, A., and Rodriguez, G.M. (2014). Role for Mycobacterium tuberculosis membrane vesicles in iron acquisition. J. Bacteriol. *196*, 1250–1256. https://doi.org/10.1128/JB.01090-13.

45. Jha, V., Pal, R., Kumar, D., and Mukhopadhyay, S. (2020). ESAT-6 Protein of Mycobacterium tuberculosis Increases Holotransferrin-Mediated Iron Uptake in Macrophages by Downregulating Surface Hemochromatosis Protein HFE. J. Immunol. *205*, 3095–3106. https://doi.org/10.4049/jimmunol.1801357.

46. White, D.W., Elliott, S.R., Odean, E., Bemis, L.T., and Tischler, A.D. (2018). Mycobacterium tuberculosis Pst/SenX3-RegX3 Regulates Membrane Vesicle Production Independently of ESX-5 Activity. mBio *9*, e00778-18. https://doi.org/10.1128/mBio.00778-18.

47. Rath, P., Huang, C., Wang, T., Wang, T., Li, H., Prados-Rosales, R., Elemento, O., Casadevall, A., and Nathan, C.F. (2013). Genetic regulation of vesiculogenesis and immunomodulation in Mycobacterium tuberculosis. Proc. Natl. Acad. Sci. USA *110*, E4790–E4797. https://doi.org/10.1073/pnas.1320118110.
48. Sardiello, M., Palmieri, M., di Ronza, A., Medina, D.L., Valenza, M., Gennarino, V.A., Di Malta, C., Donaudy, F., Embrione, V., Polishchuk, R.S., et al. (2009). A gene network regulating lysosomal biogenesis and function. Science *325*, 473–477. https://doi.org/10.1126/science.1174447.
49. Settembre, C., Di Malta, C., Polito, V.A., Garcia Arencibia, M., Vetrini, F., Erdin, S., Erdin, S.U., Huynh, T., Medina, D., Colella, P., et al. (2011). TFEB links autophagy to lysosomal biogenesis. Science *332*, 1429–1433. https://doi.org/10.1126/science.1204592.
50. Li, L., Wang, Z.V., Hill, J.A., and Lin, F. (2014). New autophagy reporter mice reveal dynamics of proximal tubular autophagy. J. Am. Soc. Nephrol. *25*, 305–315. https://doi.org/10.1681/ASN.2013040374.
51. Sun, Q., Zhang, Q., Xiao, H.-P., and Bai, C. (2015). Toll-like receptor polymorphisms and tuberculosis susceptibility: A comprehensive meta-analysis. J. Huazhong Univ. Sci. Technol. Med. Sci. *35*, 157–168. https://doi.org/10.1007/s11596-015-1405-6.
52. Hipp, M.M., Shepherd, D., Booth, S., Waithe, D., Reis e Sousa, C., and Cerundolo, V. (2015). The Processed Amino-Terminal Fragment of Human TLR7 Acts as a Chaperone To Direct Human TLR7 into Endosomes. J. Immunol. *194*, 5417–5425. https://doi.org/10.4049/jimmunol.1402703.
53. Park, J., Sharma, N., and Cutting, G.R. (2014). Melanocortin 3 Receptor Has a 5′ Exon That Directs Translation of Apically Localized Protein From the Second In-Frame ATG. Mol. Endocrinol. *28*, 1547–1557. https://doi.org/10.1210/me.2014-1105.
54. Cervantes, J.L., Dunham-Ems, S.M., La Vake, C.J., Petzke, M.M., Sahay, B., Sellati, T.J., Radolf, J.D., and Salazar, J.C. (2011). Phagosomal signaling by Borrelia burgdorferi in human monocytes involves Toll-like receptor (TLR) 2 and TLR8 cooperativity and TLR8-mediated induction of IFN-beta. Proc. Natl. Acad. Sci. USA *108*, 3683–3688. https://doi.org/10.1073/pnas.1013776108.
55. Gantier, M.P., Irving, A.T., Kaparakis-Liaskos, M., Xu, D., Evans, V.A., Cameron, P.U., Bourne, J.A., Ferrero, R.L., John, M., Behlke, M.A., and Williams, B.R.G. (2010). Genetic modulation of TLR8 response following bacterial phagocytosis. Hum. Mutat. *31*, 1069–1079. https://doi.org/10.1002/humu.21321.
56. Cervantes, J.L., Weinerman, B., Basole, C., and Salazar, J.C. (2012). TLR8: the forgotten relative revindicated. Cell. Mol. Immunol. *9*, 434–438. https://doi.org/10.1038/cmi.2012.38.
57. Ugolini, M., Gerhard, J., Burkert, S., Jensen, K.J., Georg, P., Ebner, F., Volkers, S.M., Thada, S., Dietert, K., Bauer, L., et al. (2018). Recognition of microbial viability via TLR8 drives TFH cell differentiation and vaccine responses. Nat. Immunol. *19*, 386–396. https://doi.org/10.1038/s41590-018-0068-4.
58. Kampmann, B., Gaora, P.O., Snewin, V.A., Gares, M.P., Young, D.B., and Levin, M. (2000). Evaluation of human antimycobacterial immunity using recombinant reporter mycobacteria. J. Infect. Dis. *182*, 895–901. https://doi.org/10.1086/315766.
59. Cader, M.Z., Boroviak, K., Zhang, Q., Assadi, G., Kempster, S.L., Sewell, G.W., Saveljeva, S., Ashcroft, J.W., Clare, S., Mukhopadhyay, S., et al. (2016). C13orf31 (FAMIN) is a central regulator of immunometabolic function. Nat. Immunol. *17*, 1046–1056. https://doi.org/10.1038/ni.3532.
60. Sampson, S.L., Dascher, C.C., Sambandamurthy, V.K., Russell, R.G., Jacobs, W.R., Bloom, B.R., and Hondalus, M.K. (2004). Protection elicited by a double leucine and pantothenate auxotroph of Mycobacterium tuberculosis in guinea pigs. Infect. Immun. *72*, 3031–3037. https://doi.org/10.1128/IAI.72.5.3031-3037.2004.
61. Hepburn, L., Prajsnar, T.K., Klapholz, C., Moreno, P., Loynes, C.A., Ogryzko, N.V., Brown, K., Schiebler, M., Hegyi, K., Antrobus, R., et al. (2014). Innate immunity. A Spaetzle-like role for nerve growth factor β in vertebrate immunity to Staphylococcus aureus. Science *346*, 641–646. https://doi.org/10.1126/science.1258705.
62. Hemmi, H., Kaisho, T., Takeuchi, O., Sato, S., Sanjo, H., Hoshino, K., Horiuchi, T., Tomizawa, H., Takeda, K., and Akira, S. (2002). Small anti-viral compounds activate immune cells via the TLR7 MyD88-dependent signaling pathway. Nat. Immunol. *3*, 196–200. https://doi.org/10.1038/ni758.
63. http://www.bioinformatics.babraham.ac.uk/projects/fastqc/.
64. https://www.bioinformatics.babraham.ac.uk/projects/trim_galore/.
65. Bray, N.L., Pimentel, H., Melsted, P., and Pachter, L. (2016). Near-optimal probabilistic RNA-seq quantification. Nat. Biotechnol. *34*, 525–527. https://doi.org/10.1038/nbt.3519.
66. https://mycobrowser.epfl.ch/.
67. Jeong, H.-H., Kim, S.Y., Rousseaux, M.W.C., Zoghbi, H.Y., and Liu, Z. (2019). Beta-binomial modeling of CRISPR pooled screen data identifies target genes with greater sensitivity and fewer false negatives. Genome Res. *29*, 999–1008. https://doi.org/10.1101/gr.245571.118.
68. Supek, F., Bošnjak, M., Škunca, N., and Šmuc, T. (2011). REVIGO summarizes and visualizes long lists of gene ontology terms. PLoS One *6*, e21800. https://doi.org/10.1371/journal.pone.0021800.
69. Kuznetsova, I., Lugmayr, A., Siira, S.J., Rackham, O., and Filipovska, A. (2019). CirGO: an alternative circular way of visualising gene ontology terms. BMC Bioinf. *20*, 84. https://doi.org/10.1186/s12859-019-2671-2.
70. Rodchenkov, I., Babur, O., Luna, A., Aksoy, B.A., Wong, J.V., Fong, D., Franz, M., Siper, M.C., Cheung, M., Wrana, M., et al. (2020). Pathway Commons 2019 Update: integration, analysis and exploration of pathway data. Nucleic Acids Res. *48*, D489–D497. https://doi.org/10.1093/nar/gkz946.
71. Cowen, L., Ideker, T., Raphael, B.J., and Sharan, R. (2017). Network propagation: a universal amplifier of genetic associations. Nat. Rev. Genet. *18*, 551–562. https://doi.org/10.1038/nrg.2017.38.
72. Fang, H., and Gough, J. (2014). The "dnet" approach promotes emerging research on cancer patient survival. Genome Med. *6*, 64. https://doi.org/10.1186/s13073-014-0064-8.
73. Ran, F.A., Hsu, P.D., Wright, J., Agarwala, V., Scott, D.A., and Zhang, F. (2013). Genome engineering using the CRISPR-Cas9 system. Nat. Protoc. *8*, 2281–2308. https://doi.org/10.1038/nprot.2013.143.
74. Sanjana, N.E., Shalem, O., and Zhang, F. (2014). Improved vectors and genome-wide libraries for CRISPR screening. Nat. Methods *11*, 783–784. https://doi.org/10.1038/nmeth.3047.
75. Iwai-Kanai, E., Yuan, H., Huang, C., Sayen, M.R., Perry-Garza, C.N., Kim, L., and Gottlieb, R.A. (2008). A method to measure cardiac autophagic flux in vivo. Autophagy *4*, 322–329. https://doi.org/10.4161/auto.5603.
76. Comas, I., Coscolla, M., Luo, T., Borrell, S., Holt, K.E., Kato-Maeda, M., Parkhill, J., Malla, B., Berg, S., Thwaites, G., et al. (2013). Out-of-Africa migration and Neolithic coexpansion of Mycobacterium tuberculosis with modern humans. Nat. Genet. *45*, 1176–1182. https://doi.org/10.1038/ng.2744.
77. Renna, M., Schaffner, C., Brown, K., Shang, S., Tamayo, M.H., Hegyi, K., Grimsey, N.J., Cusens, D., Coulter, S., Cooper, J., et al. (2011). Azithromycin blocks autophagy and may predispose cystic fibrosis patients to mycobacterial infection. J. Clin. Investig. *121*, 3554–3563. https://doi.org/10.1172/JCI46095.

STAR★METHODS

KEY RESOURCES TABLE

REAGENT or RESOURCE	SOURCE	IDENTIFIER
Antibodies		
Goat Polyclonal anti-Mouse Alexa Fluor 488	Thermo Fisher Scientific	A11029; RRID: AB_2534088
Goat Polyclonal anti-Mouse Alexa Fluor 555	Thermo Fisher Scientific	A32727; RRID: AB_2333276
Goat Polyclonal anti-Mouse Alexa Fluor 647	Thermo Fisher Scientific	A21236; RRID: AB_2535805
Goat polyclonal anti-mouse HRP conjugated	Sigma-Aldrich	A2554; RRID: AB_258008
Goat Polyclonal anti-Rabbit Alexa Fluor 488	Thermo Fisher Scientific	A11008; RRID: AB_143165
Goat Polyclonal anti-Rabbit Alexa Fluor 555	Thermo Fisher Scientific	A21428; RRID: AB_2535849
Goat Polyclonal anti-Rabbit Alexa Fluor 647	Thermo Fisher Scientific	A21245; RRID: AB_2535846
Goat polyclonal anti-rabbit HRP conjugated	Sigma-Aldrich	A0545; RRID: AB_10689821
IgG from human serum	Merck	I2511; RRID: AB_1163604
Mouse monoclonal anti-FLAG M2	Sigma-Aldrich	F1804; RRID: AB_262044
Mouse monoclonal anti-LC3B	Nanotools	0231-100/LC3-5F10; RRID: AB_2722733
Mouse monoclonal anti-NDP52 [OTI4H5]	Abcam	Ab124372; RRID: AB_124372
Mouse monoclonal anti-TFEB (M01), Clone S1	Abnova	H00007942; RRID: AB_548637
Mouse monoclonal anti-Ubiquitin (FK2)	Sigma-Aldrich	ST1200; RRID: AB_10681625
Rabbit monoclonal anti-c-Myc [Y69]	Abcam	Ab32072; RRID: AB_731658
Rabbit polyclonal anti-ATP6V1A	Abcam	Ab137574; RRID: AB_2722516
Rabbit polyclonal anti-MyD88	Abcam	Ab2064; RRID: AB_302807
Rabbit polyclonal anti-TLR8	Sigma-Aldrich	HPA001608; RRID: AB_1080295
Rabbit polyclonal V-ATPase A1 (H-140)	Santa Cruz	Sc-28801; RRID: AB_2258865
Rabbit polyclonal V5-Tag	Novus Biologics	NB600-381; RRID: AB_527427
Bacterial and virus strains		
Drug resistant *Mycobacterium tuberculosis* clinical isolates (NR006, NR007, NR009, NR004, NR010, NR008, NR021, NR024, NR029, NR033, NR041, NR045)	ICMR-NIRT, Chennai, India	N/A
Drug susceptible *Mycobacterium tuberculosis* clinical isolates (NS001, NS007, NS006, NS008, NS009, NS0013, NS016, NS017, NS046, NS043, NS058, NS092, NC096, NS055, NS057)	ICMR-NIRT, Chennai, India	N/A
Escherichia coli Stbl3 competent cells	Thermo Fisher Scientific	C737303
Mycobacterium bovis BCG	ATCC	35733, TMC 1010 [BCG Danish]
Mycobacterium bovis BCG-lux	Floto lab	(57)
Mycobacterium bovis BCG-lux-GFP	Floto lab	(57)
Mycobacterium chelonae	National University of Singapore	ATCC#TMC 1544 [Friedmann]
Mycobacterium fortuitum	National University of Singapore	ATCC#[TMC 1529]
Mycobacterium marinum	National University of Singapore	ATCC#[TMC 1218]
Mycobacterium scrofulaceum	National University of Singapore	ATCC# L2238 [1356, NCTC 10803, TMC 1323]
Mycobacterium tuberculosis H37Rv	ATCC	TMC 102 [H37Rv]
Mycobacterium tuberculosis H37Rv-GFP	Dr S. Newton, London, UK	N/A
Mycobacterium tuberculosis M10	Dr Chan, Seoul, Korea	N/A
Mycobacterium tuberculosis TN5904	Dr B.N. Kreiswirth, Newark, NJ, USA	N/A
Mycobacterium tuberculosis VirR- (Tn:rv0431)	Dr Rafael Prados-Rosales, Madrid, Spain	N/A

(*Continued on next page*)

Continued

REAGENT or RESOURCE	SOURCE	IDENTIFIER
Mycobacterium tuberculosis VirR-::WT (Tn:rv0431::rv0431)	Dr Rafael Prados-Rosales, Madrid, Spain	N/A
Mycobacterium tuberculosis ΔleuD ΔpanCD (Bleupan)	Dr W. Jacobs III, NY, USA	N/A
Mycobacterium tuberculosis ΔleuD ΔpanCD-GFP	Dr. Lalita Ramakrishnan, Cambridge, UK	N/A
Mycobacterium tuberculosis ΔleuD ΔpanCD-lux-GFP	This paper	N/A
Mycobacterium tuberculosis ΔleuD ΔpanCD-mCherry	Dr. Lalita Ramakrishnan, Cambridge, UK	N/A
Biological samples		
Bone marrow derived macrophages	Dr Caetano Reis e Sousa, London, UK	N/A
Bone marrow derived macrophages from mRFP-GFP-LC3 transgenic C57BL/6 mice	Prof. David Rubinstein, Cambridge, UK	N/A
PBMCs – monocyte-derived macrophages	This paper	N/A
Chemicals, peptides, and recombinant proteins		
Albumin-Dextrose-Catalase	Sigma-Aldrich	M0553-1VL
Blasticidin	Thermo Fisher Scientific	R21001
Calcium pantothenate	Sigma Aldrich	C8731-25G
CD14$^+$	Miltenyi Biotec	130-050-201
CFSE	BioLegend	423801
CL075	Invivogen	tlrl-c75
cOmplete™, Mini, EDTA-free Protease Inhibitor Cocktail	Roche	11836170001
DMEM	Sigma-Aldrich	D6429
Dual-Luciferase Assay system	Promega	E1910
ECL Advance western blotting detection	Sigma-Aldrich	RPN2106
FCS	PanBiotech	P30-3702
Ficoll-Hypaque	Amersham	17-5442-02
Formaldehyde	Sigma-Aldrich	F8775-25ML
G418/neomycin	Thermo Fisher Scientific	10131035
Geneporter® 2 transfection reagent	AMSBio	AMS.T202015
Glutamine	Sigma-Aldrich	G8540
Glutaraldehyde	Sigma-Aldrich	340855
Glycerol	Fisher	10795711
Glycine	Sigma-Aldrich	G7126
HEPES	Lonza	CC-5022
HiPerFect transfection reagent	Qiagen	301705
Human M-CSF	Peprotech	300-25-100UG
Hygromycin B	Cambridge Bioscience	H011-20mL
Imiquimod	Invivogen	tlrl-imq (5 mg)
Kanamycin	Merck Millipore	420411-5GM
L-Leucine	Sigma-Aldrich	L-8000
Lipofectamine LTX	Thermo Fisher Scientific	A12621
Lipofectamine® 3000 Transfection Reagent	Thermo Fisher Scientific	L3000008
Live Cell Imaging solution	Thermo Fisher Scientific	A59688DJ
LysoSensor Green DND-189	Thermo Fisher Scientific	L7535
LysoTracker red DND-99	Thermo Fisher Scientific	L7528

(*Continued on next page*)

Continued		
REAGENT or RESOURCE	SOURCE	IDENTIFIER
Middlebrook 7H11 agar	Sigma-Aldrich	M0428-500G
Middlebrook 7H9 broth	Sigma-Aldrich	M0178-500
Murine MCS-F	Peprotech	315-02 (100μg)
OADC	Fisher	12674697
Optiprep	Sigma-Aldrich	D1556
Osmium tetroxide	Fisher Scientific	31253.01
PBS	Sigma-Aldrich	P4474-1L
Penicillin/Streptomycin	Sigma-Aldrich	P0781
Phosphatase inhibitors	Sigma-Aldrich	4906845001
PMA	Sigma-Aldrich	P1585
Prolong Gold antifade mountant	Thermo Fisher Scientific	P36962
Puromycin	Thermo Fisher Scientific	A1113803
Resiquimod	Invivogen	tlrl-r848-5
RIPA buffer	Thermo Fisher Scientific	R0278
RNase A	New England BioLabs	T3018L
Roche DNase I	Sigma-Aldrich	#10104159001
RPMI	Sigma-Aldrich	R8758-500ML
ssRNA40	Invivogen	tlrl-lrna40
Triton X-100	Sigma-Aldrich	T9284
Tween 20	Sigma-Aldrich	P9416-50ML
Tween 80	Sigma-Aldrich	P4780-100ML
Zeocin	Thermo Fisher Scientific	R25001
Zymosan	Invivogen	tlrl-zyn
β-Mercaptoethanol	Gibco	21985023
Critical commercial assays		
AgencourtRNAClean XP beads	Beckman Coulter	a63987
Agilent High Sensitivity DNA kit	Agilent Technologies	5067–4626
Amaxa Cell Line Nucleofector Kit V	Lonza	VCA-1003
Bio-Plex 17-plex (IL-1β, IL-2, IL-4, IL-5, IL-6, IL-7, IL-8, IL-10, IL-12 (p70), IL-13, IL-17, G-CSF, GM-CSF, IFN-γ, MCP-1 (MCAF), MIP-1β and TNF-α) cytokine assay kit	Merck Millipore	HCYTOMAG-60K
DNeasy Blood and Tissue kit	Qiagen	69504
Endotoxin Free Maxi kit	Qiagen	12362
High Pure RNA isolation kit	Roche	11828665001
Hiseq Rapid SBS kit V2 50 cycles	Illumina	FC-402-4022
Magic Red Cathepsin B kit	BioRad	ICT938
PCR cleanup kit	Qiagen	28104
Pierce BCA Protein Assay Kits	Thermo Fisher Scientific	20–2,000 μg/mL
QIAampFFPE purification kit	Qiagen	56404
Qubit dsDNA HS DNA Kit	Thermo Fisher Scientific	Q32851
Qubit RNA HS Assay Kit	Thermo Fisher Scientific	Q32852
QuikChange® II XL	Stratagene	200522
SuperScript-II Reverse Transcriptase	Thermo Fisher Scientific	18064014
TruSeq small RNA library	Illumina	RS-200-0012
Deposited data		
Raw and analyzed CRISPR data	This paper	EBI-ENA accession number: PRJEB62758

(*Continued on next page*)

Continued		
REAGENT or RESOURCE	SOURCE	IDENTIFIER
RNA Seq Raw data	This paper	Gene Expression Omnibus number: GSE288494
Experimental models: Cell lines		
FLAG-tagged TFEB reporter HeLa cells	Prof. David Rubinstein, Cambridge, UK	N/A
HEK293T cells	ATCC	CRL-3216
HeLa cells	ATCC	CCL-2
Raw 264.7 cells	ATCC	TIB-71
THP-1 BLUE NFkB cells	Invivogen	thp-nfkbv2
THP-1 cells	ATCC	TIB-202
THP1 AP-1-Luc2 cells	ATCC	(RRID:CVCL_A4CA)
TLR8-expressing Raw 264.7 cells	This paper	N/A
TLR8-TFEB expressing HeLa cells	This paper	N/A
Experimental models: Organisms/strains		
C57BL/6 mice	The Jackson Laboratories	#000664
Oligonucleotides		
See Table S5	N/A	N/A
Recombinant DNA		
Human Toronto Knockout Library	Addgene	#90294
LentiCRISPRv2	Addgene	#52961
pcDNA3.1-c-myc-TLR8	This paper	N/A
pcDNA3.1-V5-TLR8	This paper	N/A
pcDNA3.1-TLR8-M1V	This paper	N/A
pcDNA^{TM}3.1/V5-His-TLR8/TLR2	This paper	N/A
pcDNA^{TM}3.1/V5-His	Thermo Fisher Scientific	V81020
pCMV-VSV-G	Addgene	#8454
pGL4 luciferase reporter vector	Promega	#TM259
psPAX2	Addgene	#12260
pSpCas9 (BB)-2A-Puro (PX459)	Addgene	#48139
Software and algorithms		
CirGO	N/A	(67)
CRISPR screen code	This paper	Zenodo: https://doi.org/10.5281/zenodo.14982932
FASTQC software	N/A	version 0.11.9
FlowJo	N/A	version 10.10.0
GraphPad Prism	N/A	version 10.2.3 (347), April 21, 2024
HGNC	N/A	https://www.genenames.org
ImageJ software	N/A	Version 2.9.0/1.53t
NIS Elements AR analysis software	N/A	Version 4.00.07
Panther tool	N/A	http://www.pantherdb.org
RAxML	N/A	version 8.2.8
Trim Galore	N/A	version 0.6.4_dev
Zen software	N/A	version 2010
ZetaView Software	N/A	version 8.05.12 SP1

EXPERIMENTAL MODEL AND STUDY PARTICIPANT DETAILS

Mycobacteria

The following strains of mycobacteria were used: *Mycobacterium tuberculosis H37Rv, M. tuberculosis H37Rv-GFP; M. tuberculosis VirR-* (*Tn:rv0431*) and its complemented strain *M. tuberculosis VirR-::WT* (*Tn:rv0431::rv0431*),[47] *M. bovis BCG*, BCG-lux (a luminescent reporter strain of *M. bovis BCG* encoding the Vibrio *lux AB* gene or GFP[58]), clinical isolates of *M. scrofulaceum, M. marinum,*

M. chelonae, M. fortuitum, multi-drug resistant (MDR) isolates of *M. tuberculosis* (TN5904 and M10); have also been used some clinical isolates of *M. tuberculosis* from the Bacteriology Division of the ICMR-National Institute for Research in Tuberculosis (Chennai, India) that were either drug-susceptible and multidrug-resistant. Isolates were grown as previously described[31,59] in Middlebrook 7H9 broth containing 0.5% glycerol, 0.05% Tween 80 and 10% albumin–dextrose–catalase enrichment.

Auxotrophic M. tuberculosis

M. tuberculosis ΔleuD ΔpanCD (Bleupan) double auxotroph strain[60] (gift from Dr Bill Jacobs) was transduced with *pSMT12-mCherry* or *pSMT12-GFP* (gifts from Dr Lalita Ramakrishnan, Cambridge, UK) or *pSMT1-LuxAB-GFP,* and grown in Middlebrook 7H9 broth containing 0.5% glycerol, 0.05% Tween 80 and 10% oleic acid–albumin–dextrose–catalase enrichment (OADC), 0.05 mg/mL L-leucine, 0.024 mg/mL calcium pantothenate. When necessary, 50 μg/mL hygromycin B, 40 μg/mL kanamycin or 50 μg/mL zeocin were added to cultures. Bacteria were grown for 15 days at 37°C, then transferred in bigger culture volume (1/100 dilution) for 10 more days in media of the same composition.

Mycobacterial homogenates

For generation of mycobacteria homogenates, mycobacterial cultures were harvested, washed, and resuspended in phosphate-buffered saline (PBS). Bacteria were disrupted by bead-beating in a bullet blender (Next Advance) for 5 min and homogenates were briefly centrifuged to remove the beads and intact cells. Experiments to identify the mycobacterial ligand for TLR8 were performed on *M. bovis* BCG homogenates either heat denatured at 95°C for 5min; or subjected to enzymatic digestion by RNase A or DNase I for 15 min at room temperature. 1 unit of RNase or DNase was used for every 1μg of *M. bovis* BCG homogenate. RNA and DNA from *M bovis* BCG were obtained from cultures grown to mid-log phase using the Roche High Pure RNA Isolation kit and the DNeasy Blood and Tissue Kit respectively, according to manufacturers' instructions.

Single cell bacterial suspensions

To prepare single cell suspensions of bacteria prior to infection, bacteria were centrifuged 24 h prior to experiment and resuspended in bacterial growth media without tween to allow one generation time and complete reformation of mycobacterial cell wall. On the day of infection, mycobacterial cultures were passed through a 27-gauge needle 10 to 12 times prior to injecting through a 5- μm filter to achieve close to single cell suspensions of bacteria.

Colony forming units

To enumerate colony forming units (CFU) counts, bacterial suspensions were plated on Middlebrook 7H11 agar with 10% OADC enrichment supplement and CFU were counted after 21 days of incubation at 37°C.

Subjects details

Healthy consented individuals were genotyped at the TLR8 locus and recalled, stratified by genotype to provide peripheral blood samples (described below). Samples from at least 5 individuals homozygous or hemizygous for ancestral (WT) TLR8 and at least 5 individuals homozygous or hemizygous for M1V TLR8 were compared in functional experiments. Informed by pilot experiments, samples size calculations, based on observed standard deviation of 10% in macrophage intracellular killing and cytokine production, indicated that recruitment of $n = 4$ subjects in each arm would provide an 80% power to detect a 20% difference in responses between genotypes (alpha 0.05).

Mammalian cell cultures

Monocyte-derived macrophages

Peripheral blood mononuclear cells (PBMCs) were generated as previously described.[31] Briefly, PBMCs were isolated from peripheral blood obtained from healthy consented subjects (approved by Regional NHS Research Ethics Committee), stratified by TLR8 WT or M1V genotype, by Ficoll-Hypaque density separation. $CD14^+$ positive selection using magnetic beads was used to extract monocytes, which were subsequently differentiated into macrophages by stimulation with 200 ng/mL M-CSF in DMEM containing 2 mM L-glutamine, 10% FCS, 100 U/ml penicillin/streptomycin. Cells were differentiated for six days before assaying.

Bone-marrow-derived macrophages

Generation and culture of bone-marrow-derived macrophages (BMDM) was caried out as previous described.[61] Femurs from 15-week-old female TLR7 knockout mice (generated as previously described[62]) or age and sex-matched C57BL/6 mouse controls were dissected to remove both ends and flushed with a 21-guage needle into serum-free DMEM. Cells were then centrifuged to remove cell culture medium and cultured in 10mL DMEM supplemented with 20% FCS, 100U/mL penicillin/streptomycin, 50μM tissue-culture grade β-Mercaptoethanol and 200 nM murine MCS-F for 3 days, after which fresh medium was added. Cells were then incubated for another 3 days before the cells were scraped and seeded onto 24-well plates for experiments.

THP-1 macrophages

THP-1 cells, THP-1 BLUE NF-κB and AP-1 reporter monocytes were maintained in RPMI 1640, 10% FCS, 100 U/ml penicillin/streptomycin, 2 mM L-glutamine, and 200 μg/mL Zeocin when needed. Cells were supplemented with 40 ng/mL 12-phorbol 13-myristate acetate PMA for 48h to stimulate differentiation into macrophages.

Other cell lines

HeLa cells, FLAG-tagged TFEB reporter HeLa cells (gift from Dr David Rubinzstein, Cambridge, UK[49]), HEK 293T cells and RAW 264.7 cells were maintained in DMEM, 10% FCS, 100 U/ml penicillin/streptomycin, 2 mM L-glutamine. G418/neomycin (500 μg/mL was added to transfected cells (TLR8/TFEB-expressing HeLa, TLR8-expressing RAW 264.7).

In vivo animal study

Specific-pathogen-free female C57BL/6 mice, from 6 to 8 weeks old, were purchased from the Jackson Laboratories, Bar Harbor, Maine. Mice were maintained in the Biosafety Level III animal laboratory at Colorado State University, and were given sterile water, mouse chow, bedding, and enrichment for the duration of the experiments. The specific pathogen-free nature of the mouse colonies was demonstrated by testing sentinel animals. All experimental protocols were approved by the Animal Care and Usage Committee of Colorado State University. The CSU animal assurance welfare number is A3572-01.

METHOD DETAILS

Extracellular membrane vesicles

Purification of extracellular membrane vesicles (MVs)[44]

M. tuberculosis H37Rv bacterial cultures were grown in 7H9 medium for 7 days, then subsequently inoculated into minimal medium and incubated at 37°C for 14 days.[43] Bacterial cultures were sequentially filtered through 0.45-µm and 0.22-µm-pore size filters, and concentrated using an Amicon Ultrafiltration system with a 100-kDa-exclusion filter. The recovered concentrate was centrifuged to recover the vesicle pellet. The membrane vesicles were purified by density gradient ultracentrifugation using OptiPrep solution prepared in Dulbecco's phosphate buffered saline.

MV RNA isolation, sequencing, and analysis

RNA was isolated from *Mtb* H37Rv MVs following and acid phenol-chlorophorm isoamyl alcohol method. The quantity and quality of the RNAs were evaluated using Qubit RNA HS Assay Kit and Agilent RNA 6000 Nano Chips (Agilent Technologies), respectively. Sequencing libraries were prepared using a TruSeq small RNA library and selecting RNA sizes from 20 to 300 nt. Briefly, starting from 300 ng of total RNA, rRNA was removed and the remaining RNA was cleaned up using AgencourtRNAClean XP beads. Purified RNA was fragmented and primed for cDNA synthesis. cDNA first strand was synthesized with SuperScript-II Reverse Transcriptase for 10 min at 25°C, 15 min at 42°C, 15 min at 70°C and pause at 4°C. cDNA second strand was synthesized with Illumina reagents at 16°C for 1 h. Then, A-tailing and adaptor ligation were performed. Libraries enrichment was achieved by PCR (30 s at 98°C; 15 cycles of 10 s at 98°C, 30 s at 60°C, 30 s at 72°C; 5 min at 72°C and pause at 4°C). Afterward, libraries were visualized on an Agilent 2100 Bioanalyzer using Agilent High Sensitivity DNA kit and quantified using Qubit dsDNA HS DNA Kit. Library sequencing was carried out on an Illumina HiSeq2500 sequencer with 50 nucleotides single end reads. Quality Control of sequenced samples was performed using FASTQC software (version 0.11.9).[63] Adapter trimming and low-quality reads removal were performed using Trim Galore version 0.6.4_dev.[64] Reads from each strain were mapped against the *M. tuberculosis* H37RV strain reference genome from Mycobrowser (Release 4, 2021-03-23) using kallisto[65] with single-end run mode. Functional categories of *M. tuberculosis* H37RV protein coding genes were extracted from Mycobrowser.[66]

Confocal microscopy

Isolated *M. tuberculosis* MVs were subsequently fluorescently labeled with carboxyfluorescein succinimidyl ester (CFSE) as previously described.[44] prior to incubation with differentiated THP-1 cells. To assess MyD88 signaling, CFSE-labelled MVs suspension in DMEM with 10% FCS were added to differentiated THP-1 cells in the presence or absence of 100 µg/mL RNAse A and incubated at 37°C for 2 h. The cells were then washed, fixed and immunostained for MyD88.

Quantification of MVs by nanoparticle tracking analysis (NTA)

Nanoparticle tracking analysis (NTA) was conducted using ZetaView (*Particle Metrix*). Instrument calibration was performed prior to EV analysis using 102 nm polystyrene beads (Thermo Fisher Scientific, USA), according to manufacturer instructions. Measurements were performed using a 405 nm 68 mW laser and CMOS camera by scanning 11 cell positions and capturing 60 frames per position at 25°C with camera sensitivity 85, shutter speed 100, autofocus and automatic scattering intensity. Samples were diluted in pre-filtered PBS to approximately 10^6-10^7 particles·ml^{-1} in Millipore DI water. Analysis was performed using ZetaView Software version 8.05.12 SP1 with a minimum brightness 30, maximum brightness 255, minimum area 5, maximum area 1000, and minimum trace length 15. Triplicate videos of each sample were taken in light scatter mode. Particle size and concentration were analyzed using a built-in protocol and plotted using graph pad Prism 8.0 software.

Scanning electron microscopy (SEM)

Cells were fixed with 2.5% glutaraldehyde, 0.1 M sodium cacodylate, 0.2 M sucrose, 5 mM $MgCl_2$(pH 7.4) and were dehydrated through a graded series of ethanol solutions before critical-point drying using liquid carbon dioxide in a Toumisis Samdri 795 device and sputter-coating with gold-palladium in a Denton Vacuum Desk-2 device. Samples were examined in a Zeiss Supra Field Emission Scanning Electron Microscope (Carl Zeiss Microscopy, LLC North America), using an accelerating voltage of 5 kV.

THP1 CRISPR knockout library

Genome-wide CRISPR library

The CRISPR knockout pooled library plasmids was prepared following the protocol previously described.[15] Briefly, the Human Toronto Knockout library (TKO V1) was amplified by transformation in Stbl3 competent cells. Colonies were scraped off plates, pooled and purified using Qiagen endotoxin free Maxi kit. Lentiviral particles were generated by transfecting HEK293T cells with the pooled CRISPR library plasmids, and used to transduce Cas-9-expressing THP-1. Cells were maintained in RPMI 1640, 10% FCS, 100 U/ml

penicillin/streptomycin, 2 mM L-glutamine, 1 μg/mL Puromycin and 10 μg/mL Blasticidin. Differentiation into macrophages was achieved by treating cells with 20 ng/mL 12-phorbol 13-myristate acetate PMA for 48h prior to experiment.

After 24h of infection with fluorescent *M. tuberculosis*, THP-1 cells were detached using accutase incubation for 20 min at 37°C 5% CO_2, spun down 300g 5min and fixed in formaldehyde 4% for 1 h. Cells were then FACS sorted to obtain the top brightest 21% of the population (together with the total population) using a Sony Biotechnology Synergy High Speed Cell Sorter. Genomic DNA was purified using Qiagen FFPE purification kit for fixed cells and amplified by PCR. PCR products were purified using Qiagen PCR clean up and reamplified using the following primers containing P5/7 adaptors as well as appropriate indexes necessary for Illumina sequencing. PCR reactions were cleaned up and remaining low molecular weight contaminants removed by AMPure XP beads purification using a ratio of 1.6:1. Purification and quantitation were validated using Agilent DNA 1000 chips and confirmed by qPCR. Sequencing was performed on Illumina Hiseq NGS using a Hiseq Rapid SBS kit V2 50 cycles (Illumina).

CRISPR screen analysis

Read counts were quantified using the cluster-based approach CB2 by aligning against the Toronto Knock out Library.[67] Guide counts were normalized to counts per 1M sequencing reads for every sample. The values for the unsorted population from all three experiments were combined, choosing the highest count across experiments to represent each guide and compared to the sorted samples from each of the three independent experiments. An aggregate fold change was calculated conservatively as the minimum fold change between the unsorted population and each of the three experiments. To test for overrepresentation of guides in the sorted vs. unsorted population, a permutation test was performed. Three rounds of label permuting were conducted to accurately simulate the quantification process generating a randomized unsorted guide count distribution for each of the three experiments. Guides that had less than 0.1 counts per 1M reads in the unsorted population were removed from the analysis. As before, the aggregate log fold change was calculated as the minimum fold change across the three (permuted) experiments. For every guide, the *p*-value was then defined as the fraction of the number of guides that had a higher minimum log fold change value in the real than in the permuted dataset, and the total number of guides analyzed.

GO terms analysis

To visualise genes in the CRISPR screen, hits with a p value < 0.05 were transformed into approved symbols using HUGO Gene Nomenclature Committee (HGNC, https://www.genenames.org). The approved symbols were then entered into Panther tools software to assign Gene Ontology (GO) terms to all the hits (http://www.pantherdb.org). REVIGO enrichment analysis[68] was used to reduce and visualise GO terms. GO terms are therefore summarised and redundancy is removed. Finally, a Python script for circular visualisation of GO terms (CirGO) was used for graphic representation.[69]

Network analysis

To investigate the functional connectivity of genes identified in the CRISPR screen, we constructed a network of interactions from the Pathway Commons database[70] and performed network propagation using a random walk with restart (RWR) algorithm.[71] RWR is designed to retain local connectivity between genes by restarting the signal diffusion process after a limited number of steps, with a fixed probability determined by a restart parameter (r).We used the implementation of RWR provided in the dnet package of the R statistical computing environment,[72] with r = 0.2 and Laplacian normalisation of the adjacency matrix. The −log10(p value) for each of the 19,102 genes in the CRISPR screen were used as starting weights for propagation. To account for the fact that highly connected genes (nodes) tend to receive higher steady state scores via RWR, we performed a permutation test in which the starting weights for genes (−log10(Pvalue)) were randomised and RWR was performed a total of 30,000 times. An empirical p value foreach gene was then calculated as the proportion of permuted steady state scores at least as large as that observed from the CRISPR screen data. Interactions between genes with p values <0.05 were (n = 928) were used to construct a sub-network from the Pathway Commons database. From the resulting sub-network, interactions between genes in the top six largest significantly enriched GO terms identified through the REVIGO enrichment analysis were visualised.

Individual CRISPR knockout cell lines

For individual single guide RNA (sgRNA) cloning, pairs of oligonucleotides were designed and ordered from Sigma with restriction enzyme-compatible overhangs, separately annealed and cloned into the transient CRISPR plasmid pSpCas9 (BB)-2A-Puro (PX459) as previously described.[73] For cloning into lentiviral vector, LentiCRISPRv2 was digested with BsmBI (*Fermentas*), and the linearized vector was gel purified before ligation of annealed guide oligo pairs.[74] The constructs were amplified in Stbl3 cells cells and plasmids were purified using endotoxin-free maxi kits. Lentiviral particles were produced by co-transfection of LentiCRISPRv2 constructs, psPAX2, and pCMV-VSV-G at a 1:2:1 ratio into HEK 293ET cells using TransIT-293 Transfection Reagent (*Mirus Bio LLC*) reagent according to manufacturer's instructions. TLR8, TLR7, and ATG12 CRISPR knockout in THP-1 cells were generated by cloning relevant targeting guide sequences into lentiGuide-Puro vector, producing viral particles by transfection into HEK 293T cells as previously described[74] and subsequently transducing THP-1 cells expressing lentiCas9-Blast. Cells were maintained in RPMI 1640, 10% FCS, 100 U/ml penicillin/streptomycin, 2 mM L-glutamine, 1 μg/mL Puromycin and 10 μg/mL Blasticidin. Single cell clones were expanded, sequenced to confirm gene knockout, and then pooled. To stimulate differentiation into macrophages, cells were treated with 20 ng/mL 12-phorbol 13-myristate acetate PMA for 48h prior to experiment.

Plasmid constructs

TLR8 was cloned into a pcDNA3.1 vector (adding c-myc or V5 tags where indicated) and the TLR8 M1V variant generated by site-directed mutagenesis using QuikChange II XL. TLR8/2 chimera constructs were generated by PCR cloning to fuse the transmembrane (TM) domain of TLR2 with the extracellular TLR8 domain using the Platinum Taq DNA Polymerase High Fidelity Master Mix (*Invitrogen*) with TLR8/2 primers. The primers used were specific for TLR8 amino acid residues 1–843. For TLR2, primers for the transmembrane domain amino acid residues 588–610 were used. The fragments were then purified, combined and used as templates for a second PCR with the fragment overlapping sequences and the respective forward and reverse primers. The resultant full-length PCR products were subsequently cloned into pcDNA3.1/V5-His.

Transfections

HEK293T cells, HeLa cells, FLAG-tagged TFEB reporter HeLa cells[49] were transfected using Lipofectamine 3000 Transfection Reagent and THP1 cells using Lipofectamine LTX according to manufacturer's instructions, and assayed 48h post transfection. Primary human macrophages and RAW 264.7 macrophages were nucleofected using the Amaxa Cell Line Nucleofector Kit V and NucleofectorII Device with programs Y-010 and D-032, respectively. Prior to transfection, complete cell culture medium was removed, and cells were incubated at 37°C 5% CO_2 in DMEM containing 10% FCS. Cells were evaluated at least 48 h post transfection, either by western blot or immunofluorescence.

SiRNA experiments

For silencing experiments, Accell SMARTpool siRNA for Human TLR8 was obtained from Dharmacon with target sequences against CAAUUAAUAUAGAUCGUUU, CUGGGAUG UUUGGUUAUA, CUAUCAACUUGGGUAUUAA and GUCUUGACUGAAAAUGAUU. PMA-differentiated THP-1 cells were transfected with 1μM of siTLR8 according to manufacturer's protocol, and assayed 72h post-transfection. Primary human macrophages were differentiated for three days and transfected with 1μM of either siTLR8 or other PRRs siRNApools using HiPerFect transfection reagent (*Qiagen*) for 5min, and complexes were added drop by drop onto the cells and incubated for 6hours. DMEM was added afterward and cells were kept at 37°C for 3 more days.

Mycobacterial infections of macrophages

Infection of primary human macrophages was adapted from Schiebler et al.[31] Primary human macrophages WT or knocked down with siTLR8 were infected with *M. tuberculosis H37Rv, M. tuberculosis* Bleupan, *M. bovis* BCG or *M. bovis* BCG-lux at a multiplicity of infection (MOI) of 5:1 for 2 h, washed in PBS and incubated at 37°C for 24 h. At indicated time points cells were washed repeatedly, lysed in ddH_2O, serially diluted and plated onto Middlebrook 7H11 agar plates for CFU enumeration or cell-associated luminescence measurement. Infection of THP-1 macrophages with *M. tuberculosis* Bleupan, *M. bovis* BCG, or clinical isolates of drug-sensitive and drug-resistant *M. tuberculosis*, was performed at a MOI of 5:1. Infected macrophages were harvested at defined time points, lysed in ddH_2O, serially diluted, and plated on 7H11 agar medium supplemented as described above.

For infection of THP1 cells with WT, VirR-, VirR-:WT strains, bacteria cultures were grown as previously described, harvested at mid-log phase, resuspended in PBS and fluorescently labeled with Carboxyfluorescein succinimidyl ester (CFSE) kit for 30 min at 37°C. Bacterial suspensions were then washed twice in PBS with centrifugation steps (3000 g for 10 min) to remove supernatants, and bacterial pellets were finally resuspended in DMEM, 10% FCS prior to macrophage infection. Infection was carried out as described above and cells were incubated either with or without 200 μg/mL RNAse A for 2 h prior to fixing and immunostaining for MyD88.

Cell death analysis

LDH release assay

To assess cell viability, 200uL of cell supernants were harvested after 24h of infection of different cell types (Human Primary macrophages and THP-1 cells) with *M. tuberculosis* Bleupan at MOI 10:1. A CyQUANT LDH Cytotoxicity assay was then immediately performed following manufacturer's instruction. In details, supernatants were incubated with CyQUANT substrate mix for 30 min at room temperature and protected from light. Reaction was then stopped by addition of the stop solution volume to volume with substrate mix. Absorbance was measure at both 490nm (sample signal) and 680nm (background signal from the instrument). Maximum release was measured by lysis cells using 10x lysis buffer. Data are presented as the percentage of maximum release on 3 independent experiments each ran in triplicate.

Live or dye staining

An orthogonal way to assess cell viability was to measure cell positively stained with Live or Dye Fixable Staining Viability Kit using flow cytometry. Basically, 24 h following infection with a range of *M. tuberculosis* Bleupan MOIs, WT and TLR8 KO THP-1 cells were washed once with PBS without Ca^{2+} nor Mg^{2+}, and detached using Accutase incubation for 15min. Accutase was inactivated by addition of FCS-containing media, cells were then harvested, washed again once with PBS and incubated at room temperature for 30min protected from light with Live or Dye at a concentration of 1uL of dye per million cells per mL. Cells were washed again once with PBS and kept at 4C in BD CellFIX solution until analysis by FACS using a BD Fortessa (analysis was run within the next 48 h). These experiments have been done in triplicate, and for each experiment every condition was assess in triplicate.

Confocal imaging

Immunofluorescence experiments were undertaken as previously described.[31] Cells were seeded on glass coverslips in 24-well tissue culture plates prior to infection with either *M. tuberculosis* H37Rv or *M. tuberculosis ΔleuD ΔpanCD* (Bleupan) double auxotroph expressing either GFP- or mCherry. Following incubation at various time points, cells were washed with PBS, fixed wih 4% paraformaldehyde (PFA) in PBS for at least 30 min and permeabilized for 5 min with 0.1% Triton X-100 prior to immunostaining. Primary antibodies (against MyD88, V-ATPase, Ubiquitin, NDP52, LC3, V5 or Myc) were diluted to recommended concentrations in staining medium (DMEM, 10% FCS, 10 mM Glycine, 10 mM HEPES pH 7.4) to which IgG (1:00) was added and the cells were incubated at room temperature for 2 h. Cells were subsequently washed twice in staining medium. Cells were incubated with secondary antibodies (Alexa Fluor 555 and 647 (*Invitrogen*)) for 30 min, protected from light. The cells were subsequently washed, and the coverslips were dipped in water prior to mounting on slides using ProLong Gold Antifade Mountant with DAPI. Slides were left to dry overnight, protected from light. Images were acquired either on a Zeiss LSM780 or LSM880 confocal microscope (Plan-Apochromat 63x/1.40 Oil immersion lens) and analyzed with Zen 2010 software, Zeiss LSM Image Browser (*Carl Zeiss*), or NIS Elements AR analysis (*Nikon*) software and ImageJ.

For analysis of lysosomal number and acidification in THP1 macrophages, uninfected THP1 macrophages were either treated with the TLR8 agonist R848 at 10 μg/mL or left untreated for 24 h at 37°C, subsequently washed and incubated with 40 nM LysoTracker Red DND-99 and 1 μM LysoSensor Green DND-189 for 15 min. The cells were then washed twice with PBS after which Live Cell Imaging solution was added. Live confocal imaging was carried out on the Zeiss LSM 780 UV. Quantitation of lysosomes was performed using the ImageJ plugin on Fiji app. HEK 293T co-transfected with TLR8 WT-Myc tagged and TLR8 M1V-V5 tagged were fixed in methanol-acetone for immunofluorescent staining with anti-Myc and anti-V5 antibodies, and counterstained with Alexa 488 and Alexa 647 conjugated secondary antibodies. All cells were visualised using a Leica True Confocal Scanner SP5.

Bone marrow-derived macrophages were generated (as previously described[61]) from femurs of transgenic mice stably expressing mRFP-GFP-LC3[75] (kind gift from Dr David Rubinsztein, Cambridge, UK), and were either left untreated or treated with 10ug/mL of TLR8 agonist R848 for 24hours at 37°C 5% CO_2. Cells were then washed with PBS, and incubated in live cell imaging solution (*Invitrogen*) prior to live confocal imaging to visualize lysosomes. Imaging was carried out using on a Zeiss LSM 780UV microscope and quantitation of lysosomes was performed using ImageJ.

Cytokine analysis

Primary macrophages were infected with either *M. tuberculosis* ΔleuD ΔpanCD (Bleupan) double auxotroph or *M. bovis* BCG, and either left untreated or treated with R848 10 μg/ml. Cell culture supernatant was collected 24 h post infection and analyzed using Bio-Plex 17-plex (IL-1β, IL-2, IL-4, IL-5, IL-6, IL-7, IL-8, IL-10, IL-12 (p70), IL-13, IL-17, G-CSF, GM-CSF, IFN-γ, MCP-1 (MCAF), MIP-1β and TNF-α) cytokine assay kit (*Biorad*) according to manufacturer's instructions.

Activation assays to determine TLR ligand

Dual luciferase assay

HEK293T cells were co-transfected with TLR8/2 and the pGL4 luciferase reporter vectors (*Promega*) using Geneporter 2 (*Genlantis*) transfection reagent according to manufacturers' protocol. HEK293T cells expressing the TLR8/2 and the pGL4 luciferase reporter vectors were treated with various ligands 24h post-transfection. Cells were lysed in passive lysis buffer and lysates were analyzed for luciferase activity using the Dual-Luciferase Assay system (*Promega*). The resultant TLR8/2 chimera generated to promote stable surface expression of TLR8 in HEK293T cells were treated with either whole or lysed mycobacteria, TLR8 ligands CL075 or ssRNA40 (*InvivoGen*) or TLR2 ligand Zymosan (*InvivoGen*) to monitor NF-κB signaling by luminescence.

Quantification of NF-κB activation

differentiated TLR8 knockdown (or control) THP-1 BLUE cells were infected with *M. tuberculosis* H37Rv, *M. bovis* BCG, *M. marinum*, *M. scrofulaceum, M. fortuitum*, or *M. chelonae,* or treated with the TLR8 ligand ssRNA40. Supernatants were collected after 24hours and the levels of NF-κB-induced SEAP were quantified by colorimetric analysis, according to manufacturer's instructions.

Phylogenetic analysis

Maximum likelihood phylogenetic tree of all isolates tested were constructed using RAxML (version 8.2.8), generated by mapping detected SNP positions to *M. tuberculosis H37Rv* strain. Representative strains from the main six *M. tuberculosis* lineages described by Comas et al., (2013)[76] were included in the analysis for genomic context.

Quantitation of lysosomal degradative capacity

Uninfected control or R848-tretated THP-1 macrophages were incubated at 37°C for 24hours, washed and incubated with Magic Red Cathepsin B Kit for 1 h according to manufacturer's instructions, then washed twice in PBS. Cells were then resuspended in colorless live imaging solution and transferred onto 96 well plate for detection of cell-associated fluorescence on the CLARIOstar Plus Multi-mode Microplate Reader (*BMG Labtech*).

Analysis of phagosomal pH and size

The pH of phagosomes containing MTB was assessed as previously described.[77] Briefly, primary human macrophages from individuals that were either homo/hemizygous for the WT and M1V alleles were incubated with PFA-killed *M. tuberculosis H37Rv* double-labelled with acid quenchable (FITC) and pH-resistant (Alexa 633) for a 1h pulse and 23 h chase. The cells were analyzed by flow cytometry and intracellular calibration was performed as previously described.[77] At least 3 independent experiments were performed, each of them on 5 donors for each genotype, assessing at least 3000 cells per donor using flow cytometry.

For electron microscopy visualization, primary macrophages were infected with *M. bovis* BCG (MOI 10:1). After 24 h of infection, macrophages were washed and fixed in 0.4% glutaraldehyde for 2 h at room temperature. Samples were then post-fixed in 1% osmium tetroxide followed by dehydration in an ascending graded series of ethanol and embedding in LR white resin. Ultrathin sections (50-70nm) were stained with 2% uranyl acetate and lead citrate and examined in a JM1010 electron microscope (*JEOL*). Phagosome area was measured using ImageJ. At least 150 phagosomes per donor were evaluated, (with 5 donors per genotype) in 3 independent experiments.

Western blotting

At the indicated time points, cells were washed twice with PBS, and lysed using RIPA buffer containing a proteinase inhibitor cocktail and phosphatase inhibitors. Total protein content was quantified by BCA (*Thermo Scientific*) prior to loading at 20μg and resolving on 17% SDS-PAGE gels, and electro-blotting on to PVDF membranes (*Millipore*) in a wet transfer Cell (*Bio-Rad*). PVDF membranes were blocked by incubation in PBS supplemented with 5% (w/v) fat-free milk powder and 0.005% (v/v) Tween 20 for 1 h at room temperature. Membranes were washed repeatedly and incubated with primary antibodies following manufacturer recommended concentrations overnight at 4°C. Membranes were then washed and incubated for 1 h with 1:50 000 dilution of the horseradish peroxidase-(HRP) conjugated secondary antibodies: HRP (*Santa Cruz Biotechnology*). Membranes were revealed using ECL Advance Western Blotting Detection kit according to the manufacturer's instructions.

Mouse infection experiment

C57BL/6 mice were challenged by low-dose aerosol exposure with M. tuberculosis using a Glas-Col aerosol generator calibrated to deliver 50–100 CFU of bacteria into the lungs. Information regarding preparation of bacterial stocks and growth characteristics of the various bacterial strains ($n = 5$) used were as previously described. Strain MDR-TB M10 (resistance profile: Low-level fluoroquinolone resistance, Isoniazid, Rifampicin, Ethambutol, Streptomycin and Pyrazinamide) was originally provided by Dr. Chan, (*Seoul*, *Korea*). Strain MDR-TB TN5904 (resistance profile: INH (R, 1.6), EMB (S), RIF (R > 8), STR (R, 10), KAN (S) was originally provided by B. N. Kreiswirth, (Public Health Research Institute TB Center, Newark, NJ).

On Day 1 after infection, enumeration of bacteria was performed on two mice. Treatment was started from Day 20 to Day 50 after infection and consisted of the following groups: Control (saline; 0.1 mL intraperitoneal injection once daily) and R848 (2 mg/kg by 0.1 mL intraperitoneal injection once daily). On days 20, 35 and 50 following infection, bacterial loads in the lungs and spleen, lung and spleen histology, and flow cytometry were determined in 5 mice from each group. Bacterial counts were determined by plating serial dilutions of homogenates of lungs on nutrient 7H11 agar and counting colony-forming units after incubation at 37°C. All experimental protocols were approved by the Animal Care and Usage Committee of Colorado State University, and experiments were performed in accordance with NIH guidelines. To minimize bias, two groups of independent researchers performed the experiment. One group dosed the animals, whereas the second group determined bacterial burden in the different organs. A total of five animals were infected for each time point. Statistical analysis was performed by first converting CFU to logarithmic values and evaluated by a one-way ANOVA followed by a multiple comparison analysis of variance by a one-way Tukey test (SigmaStat software program). Differences were considered significant at the 95% level of confidence.

QUANTIFICATION AND STASTITICAL ANALYSIS

Having confirmed the normality of data, P-values for assays were determined using two-tailed Student's t-test or ANOVA (as indicated) using GraphPad. Unless otherwise indicated, experiments were performed on at least three separate occasions with at least triplicate samples for each condition and represented as mean and standard error (SEM).

Cell Reports, Volume *44*

Supplemental information

Phagosomal RNA sensing through TLR8 controls susceptibility to tuberculosis

Charlotte Maserumule, Charlotte Passemar, Olivia S.H. Oh, Kriztina Hegyi, Karen Brown, Aaron Weimann, Adam Dinan, Sonia Davila, Catherine Klapholz, Josephine Bryant, Deepshikha Verma, Jacob Gadwa, Shivankari Krishnananthasivam, Kridakorn Vongtongsalee, Edward Kendall, Andres Trelles, Martin L. Hibberd, Joaquín Sanz, Jorge Bertol, Lucia Vázquez-Iniesta, Kaliappan Andi, S. Siva Kumar, Diane Ordway, Rafael Prados-Rosales, Paul A. MacAry, and R. Andres Floto

Supplementary Figure 1

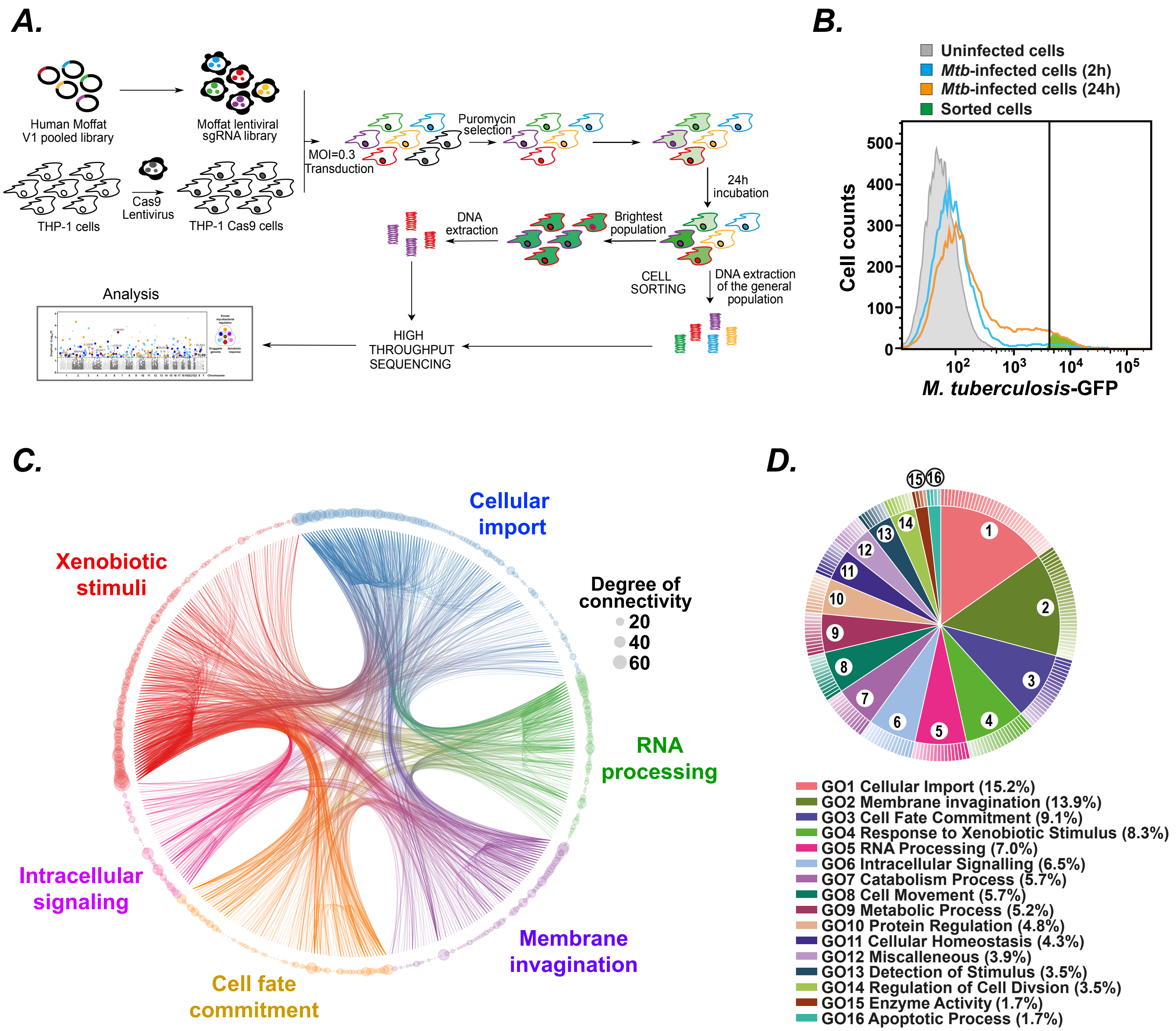

Supplementary Figure 2

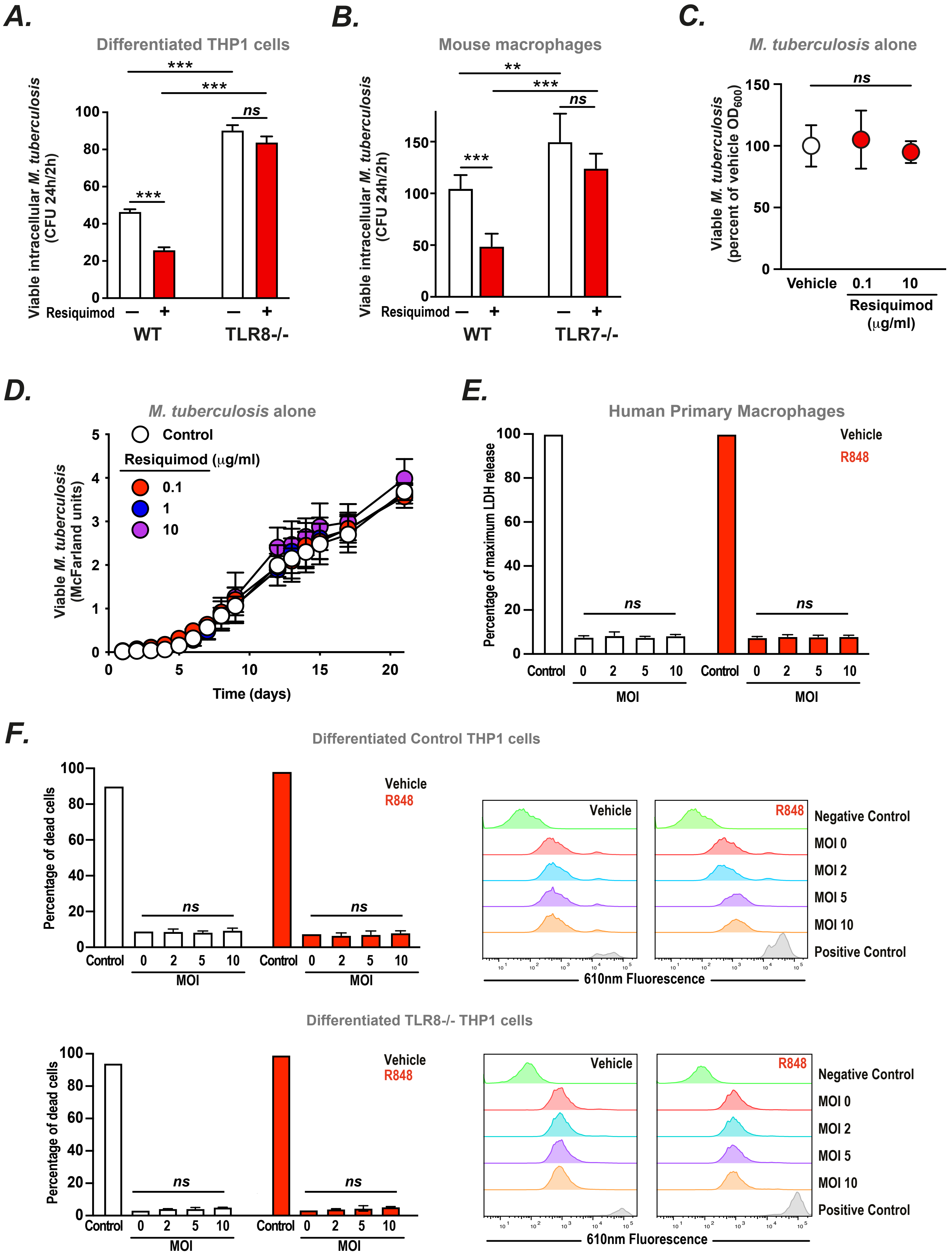

Supplementary Figure 3

A.

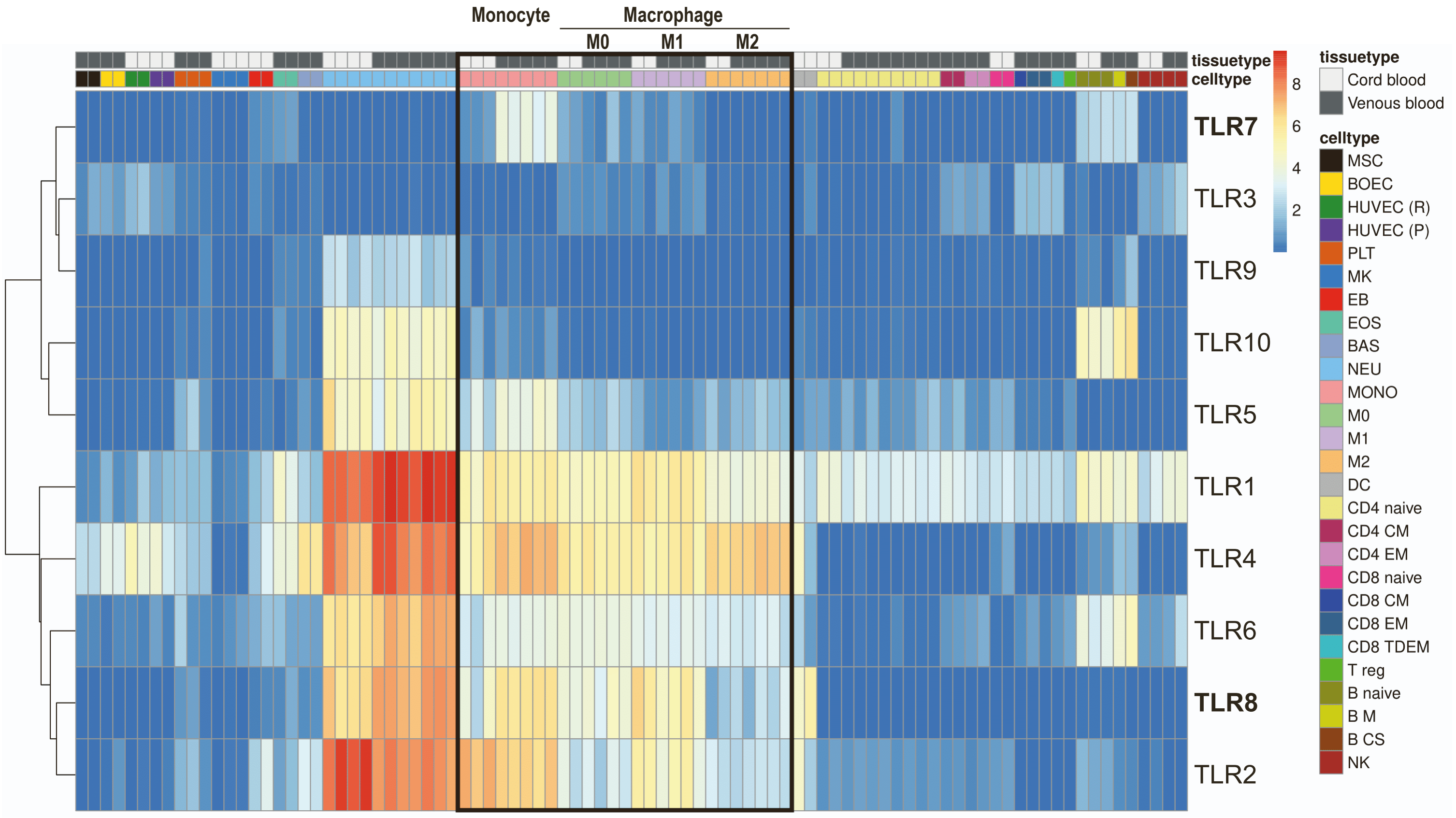

B.

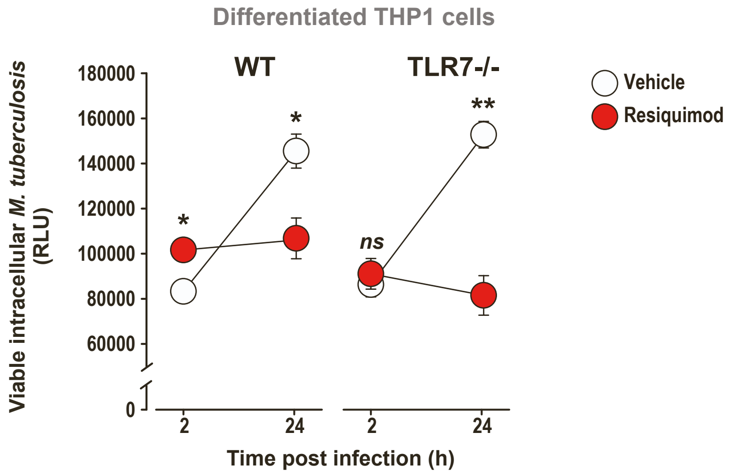

Supplementary Figure 4

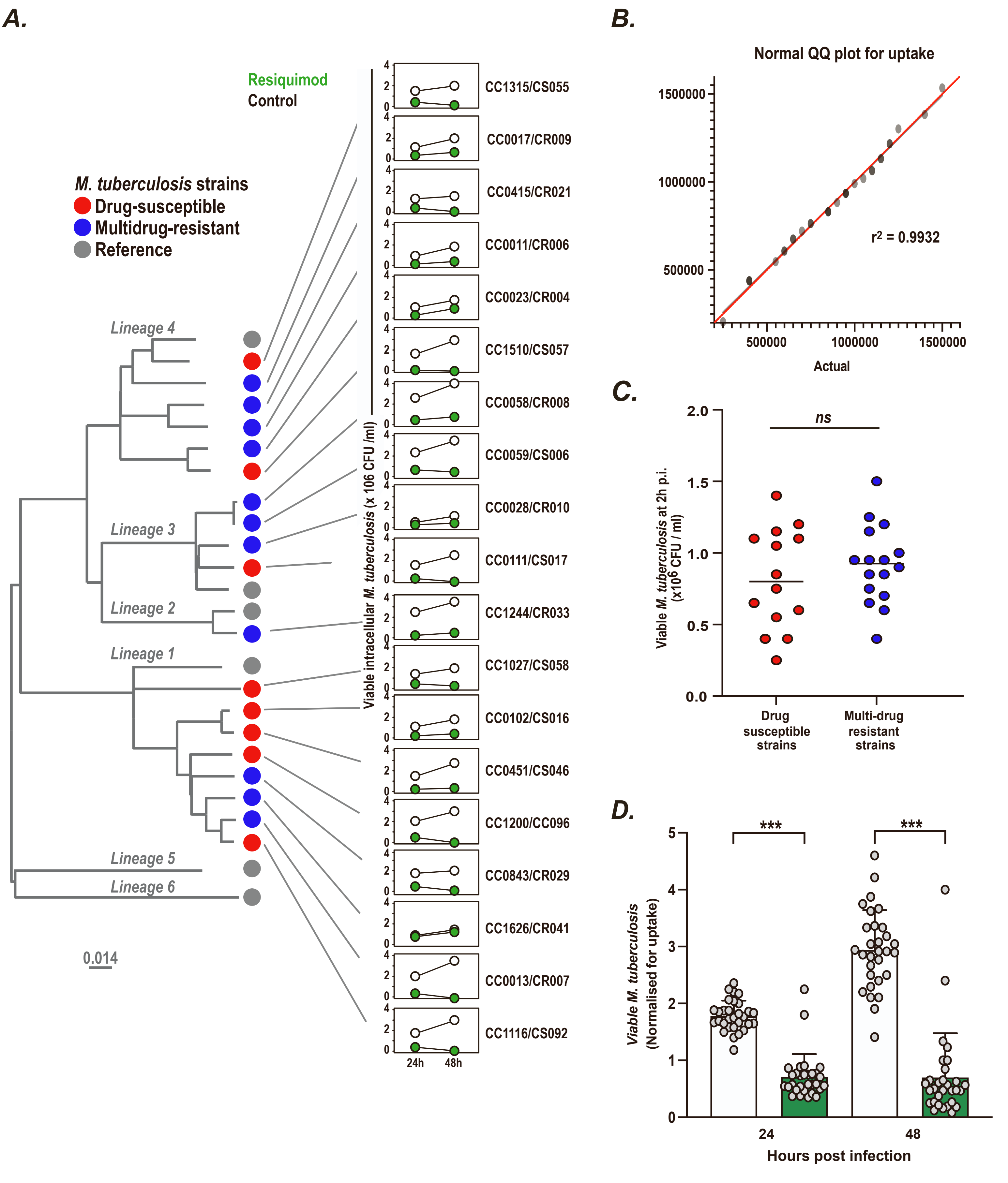

Supplementary Figure 5

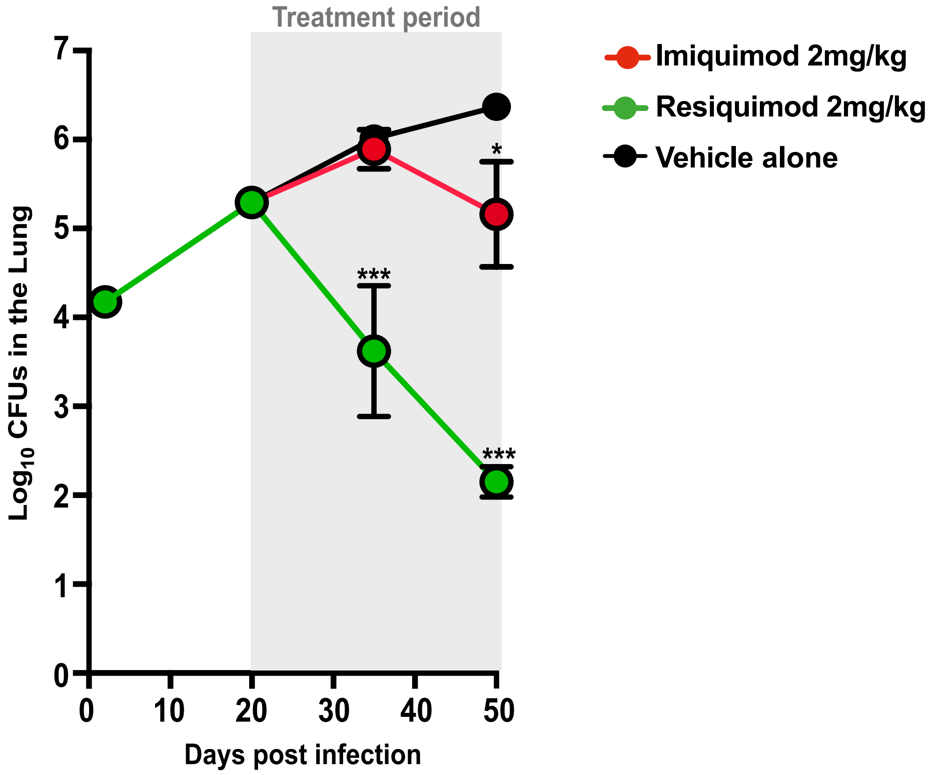

Supplementary Figure 6

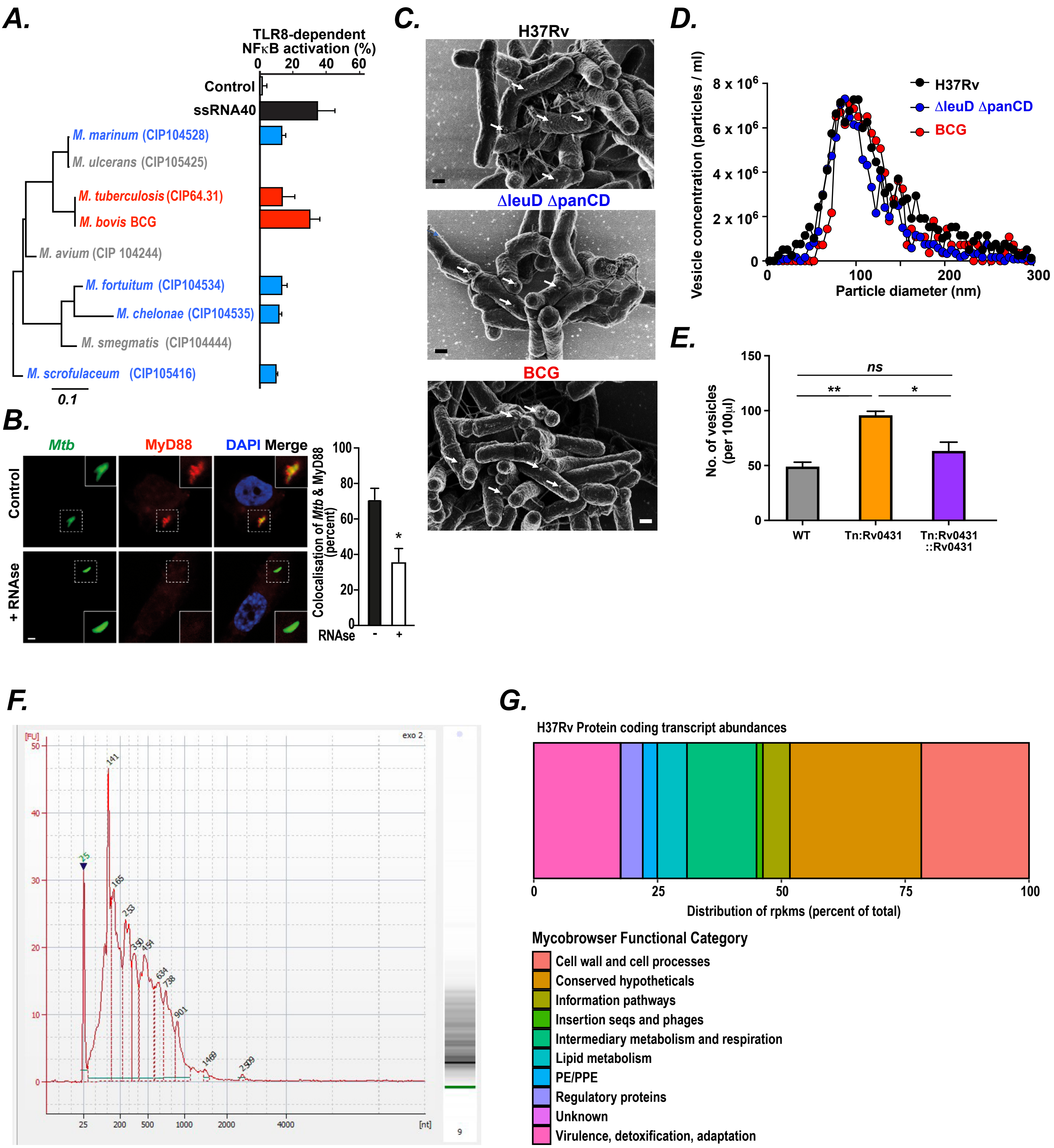

Supplementary Figure 7

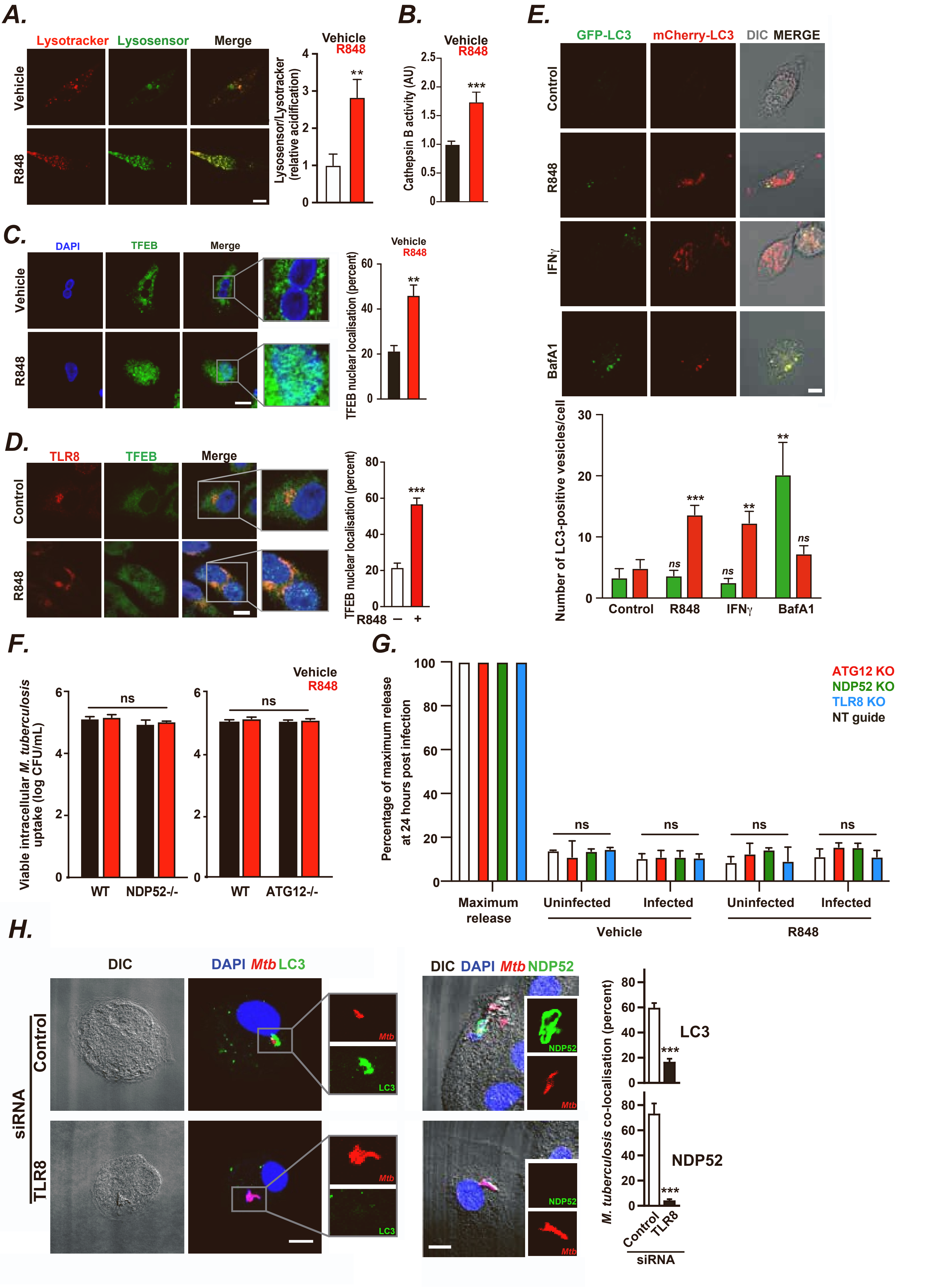

Supplementary Figure 8

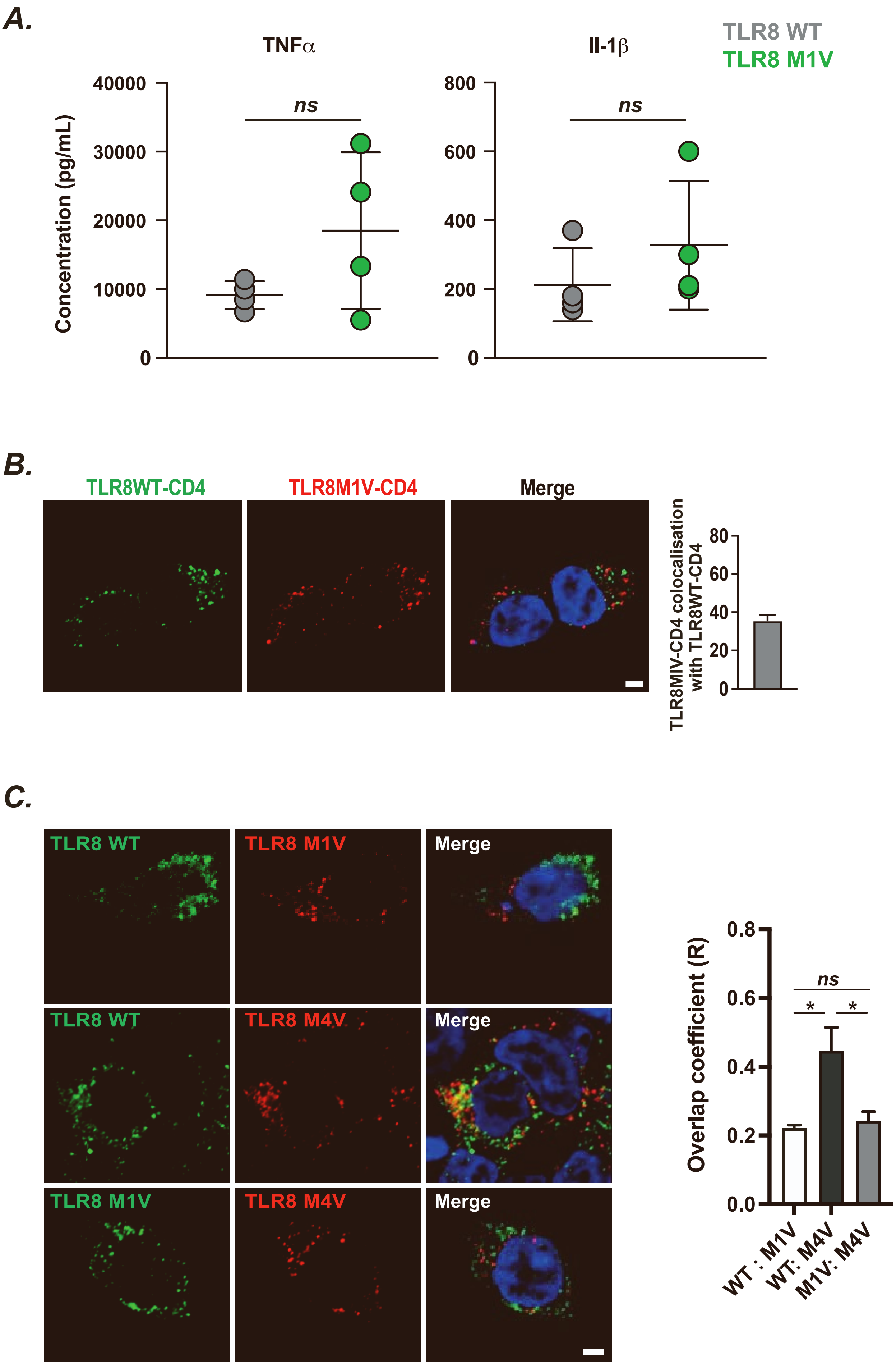

Figure S1. Genome-wide CRISPR screen of M. tuberculosis-infected THP-1 cells, Related to Figure 1. (A) Schematic representation of CRISPR screen workflow. **(B)** Representative flow cytometry histograms of THP-1 macrophages expressing the CRISPR knockout library showing uninfected cells (grey), cell infected with GFP-expressing *M. tuberculosis* ΔleuD ΔpanCD (Bleupan) after 2 h (blue) and 24 h (orange), and the FACS-sorted population (green). **(C).** Network propagation identifies interactions between gene hits in enriched GO terms (identified through REVIGO). The −log10(P value) of each gene from the CRISPR screen was used as a starting weight for propagation of the network. Node size reflects the degree of connectivity per node. **(D)** Visualisation of Gene Ontology (GO) terms (using CirGO (Circular Gene Ontology) python script), assigned to hits using Panther Tools and summarized and cleared for redundancy using REVIGO (Reduce & Visualize Gene Ontology terms) software.

Figure S2. Specificity of Resiquimod for human TLR8 and mouse TLR7 in macrophages, Related to Figure 1. **(A, B)** Viable intracellular *Mtb* (*Mtb* ΔleuD ΔpanCD Bleupan), expressed as the ratio of cell-associated CFUs at 24h/2h, in **(A)** wild type (WT) or TLR8 knockout (TLR8-/-) THP-1 macrophages or **(B)** bone marrow-derived macrophages from wild type (WT) or TLR7 knockout (TLR7-/-) mice, treated with Resiquimod (red) or vehicle alone (white). **(C, D)** The direct effect of Resiquimod (at a range of concentrations) on *M. tuberculosis* growth (in the absence of mammalian cells) assessed **(C)** after 24 h and **(D)** as growth curves (measured by absorbance).**(E, F)** Assessment of macrophages viability upon *Mtb* infection by LDH assay **(E)** and flow cytometry analysis of Live or Dye staining **(F)**. **(E)** Quantification of LDH released by Human Primary Macrophages after 24hours of infection with a range of *M. tuberculosis* MOIs, treated with Resiquimod (red) or vehicle alone (white). Data shown as percentage of maximum LDH release by lysed macrophages. **(F)** Quantification (left) of non-targeting guide (control, top) and TLR8 KO (bottom) THP1 cells positive to Live or Dye staining 24hours after infection with a range of *M. tuberculosis* MOIs, treated with Resiquimod (red) or vehicle alone (white). Representative flow cytometry histograms (right) of Live or Dye positive cells staining in non-targeting guide (control, top) and TLR8 KO (bottom) THP1 cells.

Figure S3. Expression of human Toll-like receptors in mature haemopoietic cell types, Related to Figure 1. (**A**) Heatmap shows the expression of Toll-like receptor (TLR) genes across mature hematopoietic cell types from BLUEPRINT consortium[76] data sets (n = 90). Values were normalised to fragments per kilobase per million (FPKM) and are displayed as log2(FPKM + 1). Key: mesenchymal stem cells (MSC), blood outgrowth endothelial cell progenitors (BOEC), umbilical vein endothelial cells (resting and proliferating; HUVEC R and P), platelets (PLT), megakaryocytes (MK), erythroblast (EB), eosinophils (EOS), basophils (BAS), neutrophils (NEU), monocytes (MONO), macrophages (M0), lipopolysaccharide-activated macrophages (M1), alternatively activated macrophages (M2), dendritic cells (DC), naïve CD4/CD8 cells (CD4/CD8 naive), central memory CD4/CD8 lymphocytes (CD4/CD8 CM), effector memory CD4/CD8 lymphocytes (CD4/CD8 EM), terminally differentiated effector memory CD4/CD8 lymphocytes (CD4/CD8 TDEM), regulatory CD4 lymphocytes (T reg), naive B lymphocytes (B naive), memory B lymphocytes (B M), class switch B lymphocytes (B CS), natural killer cells (NK). These data can be publically accessed at https://blueprint.haem.cam.ac.uk/bloodatlas/.

(**B**) Lack of contribution of TLR7 to Resiquimod signaling in THP1 macrophages. Wild type (WT) and TLR7-/- knockout differentiated THP1 cells were infected with a luminescent reporter strain of *Mtb* (*Mtb* ΔleuD ΔpanCD Bleupan) at an input MOI of 10:1. Viable intracellular *Mtb* (assessed by cell-associated luminescence, reported as relative light units; RLU) were determined at 2h and 24h post infection in the presence of Resiquimod (10 μg/ml; *red*) or vehicle alone (*white*). Data (mean +/- SEM) are representative of experiments performed in at least triplicate on at least three independent occasions. * $p < 0.05$; ** $p < 0.005$ (Student's t-test).

Figure S4. Enhanced intracellular killing of *M. tuberculosis* clinical isolates by Resiquimod, related to Figure 1. (A).THP1 macrophages were infected with a phylogenetically diverse collection of drug-susceptible (red) or multidrugresistant (blue) *Mtb* clinical isolates and co-treated with Resiquimod or vehicle alone for 24 or 48 hours and viable intracellular mycobacteria were enumerated through cell-associated colony forming units (CFU/ml shown for each clinical isolate at each time point. **(B)** Quantile-quantile plot comparing empirically observed uptake CFU values to those expected from a normal distribution with matching mean and variance. **(C, D)** Viable intracellular *Mtb* (mean ± SD) recovered from THP1 macrophages infected with the clinical isolates shown in (A) at 2 h **(C)** and 24 h and 48 h post-infection **(D)**, with Resiquimod (green) or vehicle control (white). Panel D is normalized to uptake shown in (C).

Figure S5. Effect of Resiquimod on *M. tuberculosis* infection *in vivo,* Related to Figure 1. (A) C57BL/6 mice were infected via aerosol with *Mycobacterium tuberculosis* MDR strain M10 and were then treated with Resiquimod (2 mg/kg, i.p. once daily; green), Imiquimod (2mg/kg, ip once daily; red) or vehicle control (black) for 30 days. Viable mycobacteria were enumerated at 1, 20, 35 and 50 days post infection by plating serial dilutions of lung homogenates on nutrient 7H11 agar and quantifying CFU after 3-4 weeks incubation at 37ºC, and are expressed as log10 CFU (mean +/- SEM, n = 5 mice per condition). $*p < 0.05$, $***p < 0.001$ (determined by ANOVA and the Tukey post-test).

Figure S6. Extracellular membrane vesicles produced by H37Rv, ΔleuD ΔpanCD (Bleupan) ***M. tuberculosis*****, and** ***M. bovis*** **BCG, Related to Figure 2. (A)** TLR8-dependent NFkB activation in response to homogenates derived from M. tuberculosis and M. bovis BCG (red) as well from rapid-growing (*M. fortuitum, M. chelonae*) and slow-growing (*M. marinum, M. scrofulaceum*) nontuberculous mycobacteria (blue) at varying evolutionary distance from the MTB complex was assessed using NFkB reporter THP-1 macrophages (THP-1-Blue) following transfection with TLR8 siRNA or scrambled controls. The response to the TLR8 ligand ssRNA40 (black) is shown as a positive control. Maximum likelihood phylogenetic tree (generated using RAxML, version 2.8.2) of representative isolates shown (scale bar indicates the number of substitutions per variable site). **(B)** TLR8 detects *Mtb*-derived RNA within macrophage phagosomes. THP-1 macrophages were infected with GFP-labelled *Mtb* (ΔleuD ΔpanCD (BleuPan)) in the presence of RNAse A (100 µg/ml) or vehicle (control). Quantitative confocal microscopy was used to detect colocalization of Mtb (green) with MyD88 (red) in both conditions. Nuclei stained with DAPI (blue). **(C)** Scanning electron micrographs of *Mtb* H37Rv (*top*), *Mtb* ΔleuD ΔpanCD (Bleupan) (*middle*), and *M. bovis* BCG (*bottom*) illustrating MV production. Scale bar 200 nm. **(D)** Quantification of extracellular membrane vesicles (MV) in culture supernatants of *Mtb* H37Rv (black), *Mtb* ΔleuD ΔpanCD (Bleupan) (blue), and *M. bovis* BCG (*red*) using nanoparticle tracking analysis. Data shown indicates mean values of three independent biological replicates, show no significant difference in MV characteristics between *Mtb* strains. **(E)** Quantification of membrane vesicles obtained from wild type (WT, *grey*), ∆virR mutants (virR^{-}, *yellow*) and complemented ∆virR mutants (virR^{-}: WT, *purple*). $^{*}p < 0.05$, $^{***}p < 0.001$ (determined by ANOVA). **(F)** RNA was extracted from *Mtb* H37Rv and analysed using an Agilent Bioanalyzer. Tapestation gel shows RNA profile. Graph shows RNA concentration in fluorescent units (fu) against nucleotide length (nt). Representative of 3 independent experiments. **(G)** Relative abundance of protein coding RNA reads stratified by Mycobrowser functional categories.

Figure S7. Activation of TLR8 increases TFEB translocation and stimulates xenophagy, Related to Figure 3. (A,B). Activation of TLR8 using Resiquimod increases numbers and degradative capacity of lysosomes. Uninfected THP-1 macrophages were incubated with Resiquimod and **(A)** subsequently incubated with LysoTrackerRed DND-99 and LysoSensorGreen DND-189 for 15 minutes prior to live confocal imaging to visualise total numbers of lysosomes (red) and acidified lysosomes (green), or **(B)** incubated with Magic Red™ MR-(RR)2 reagent for 1 hour prior to fluorescence measurements (to evaluate cathepsin B activity). Data (mean +/- SEM) are representative of at least three independent experiments performed in triplicate. *** $p < 0.001$ (ANOVA). **(C)** Resiquimod treatment of uninfected primary human macrophages for 24h leads to nuclear translocation (and presumed activation) of TFEB monitored and quantified by confocal microscopy (TFEB green, nuclei blue). Images and data (mean +/- SEM) are representative of experiments performed in triplicate on at least three independent occasions with a minimum of 50 cells analysed per replicate. ** $p < 0.01$, Student's t-test. Scale bar, 4 µm. **(D)** HeLa cells stably expressing Flag-tagged TFEB (green) were transfected with myc-tagged TLR8 (red) and treated with Resiquimod or vehicle control for 24 h. Nuclear translocation of TFEB was quantified by confocal microscopy of DAPI-stained cells (blue). Experiments were performed in triplicate, with at least 50 cells analysed per sample. Data (mean +/- SEM) are representative of at least two independent experiments. ***$p < 0.001$ (Student's t-test). Scale bar 5 µm. **(E)** Bone marrow-derived macrophages from transgenic mice expressing mRFP-GFP-tagged LC3 (56) were treated with vehicle control, Resiquimod (10 µg/ml), and interferon gamma (IFN-g; 200 ng/ml) as a positive control for autophagic flux enhancement, or Bafilomycin A1 (BafA1; 200 nM) as an inhibitor of autophagic flux, for 24 h and imaged using live cell confocal microscopy to quantify acidified (mCherry+ GFP–; red) and non-acidified (mCherry+ GFP+, green) vesicles. Experiments were performed in duplicate, with >50 cells analysed per sample. Data (mean +/- SEM) are representative of at least three independent repeats. **$p < 0.01$; ns, non-significant (Student's t-test). Scale bar 5µm. **(F)** Viable intracellular *Mtb* quantitation at 2 hours post infection in Control, NDP52 KO (left) or ATG12 KO (right) THP1 cells, treated with either Resiquimod (R848, red) or vehicle (black). **(G)** Quantification of LDH released by Non-Targetting guide control (white) TLR8 KO (blue) ATG12 (red) or NDP52 KO (green) THP1 cells after 24hours of infection with *M. tuberculosis* at MOI 10:1, treated either with Resiquimod (R848) or vehicle alone. Data shown as percentage of maximum LDH release by lysed macrophages. **(H)** Representative images of LC3 (left) or NDP52 (right) recruitment to *Mtb*-containing phagosomes in Human primary macrophages from M1V hemizygous healthy male volunteers either transfected with control (top) or TLR8 siRNA(bottom). Histogram of LC3 (top) or NDP52 (bottom) recruitment to Mtb-containing phagosomes in control (white) or TLR8

knocked down (black) Human primary macrophages. Data (mean +/- SEM) are representative of at least three independent experiments. ***p < 0.001 (Student's t-test). Scale bar 5 μm.

Figure S8. Cytokine profile expression of TLR8 M1V Primary Human Macrophages during *M. tuberculosis* infection, Related to Figure 4. (A) Primary human macrophages from healthy volunteers that are homozygous or hemizygous for the ancestral TLR8 (TLR8 WT, grey) or M1V variant (TLR8 M1V, green) (n = 5 for each genotype) were infected with either *Mtb* CDC1551 or *M. bovis* BCG. Secreted cytokines were measure in supernatants at 24 hours post infection. **(B)** Confocal microscopy of HEK 293T cells co-transfected with two of the following TLR8 receptors: (top) wild type (green) and M1V (red); (middle) wild type (green) and M4V (where the second methionine has been mutated to a valine to enforce transcription from the first methionine, red); and (bottom) M1V (green) and M4V (red). Quantification of receptor colocalization indicates only limited colocalization of the M1V variant with the other receptors, suggesting a descrete effect of the truncated signal peptide on intracellular localization. Scale bar 2 μm. **(C)** HEK293T cells were co-transfected with chimaeric receptors made from the signal peptide and intracellular domain of TLR8 joined to the extracellular and transmembrane domains of CD4. Cells were simultaneously transfected with tagged chimaeric receptors containing the wild type signal peptide (WT TLR8-CD4, green) and M1V signal peptide (M1V TLR8-CD4, red), and imaged using confocal microscopy. Quantification showing limited colocalization of WT and M1V fusion receptors shown. Scale bar 2μm. All experiments were performed in triplicate, with at least 50 cells analysed per coverslip. Data (mean +/- SEM) are representative of at least three independent experiments. Vesicles per cell and overlap coefficients were quantified using Image J. *$p < 0.05$, (Student's t-test).

TABLE 2

DRUGGABLE GENES

Gene	Gene Name	HGNC ID	Location	FC	*p* value	-Log10(*p* value)
ACVR2B	activin A receptor type 2B	HGNC:174	3p22.2	2422709039	0011205167	1950581663
ADM	adrenomedullin	HGNC:259	11p15.4	1010620394	0022557127	164671622
ADORA2B	adenosine A2b receptor	HGNC:264	17p12	403870386	0042496453	1371647322
ADRA1A	adrenoceptor alpha 1A	HGNC:277	8p21.2	1418884162	0017517248	1756534118
ALDH1A1	aldehyde dehydrogenase 1 family member A1	HGNC:402	9q21.13	4293361497	0006067427	221699546
APEX1	apurinic/apyrimidinic endodeoxyribonuclease 1	HGNC:587	14q11.2	5633394859	0034324999	146438947
APOF	apolipoprotein F	HGNC:615	12q13.3	7872610167	0027156628	1566124162
BIRC3	baculoviral IAP repeat containing 3	HGNC:591	11q22.2	1207276882	0019816999	1702962122
BMP5	bone morphogenetic protein 5	HGNC:1072	6p12.1	344462978	0048441552	131478195
BMP6	bone morphogenetic protein 6	HGNC:1073	6p24.3	1493907417	001702794	1768837901
BOC	BOC cell adhesion associated, oncogene regulated	HGNC:17173	3q13.2	1805727403	171E-04	3766349101
BPIFA2	BPI fold containing family A member 2	HGNC:16203	20q11.21	5881979322	0033444243	1475678626
BRD3	bromodomain containing 3	HGNC:1104	9q34.2	4536857795	0039829721	139979274
BTD	biotinidase	HGNC:1122	3p25.1	5033092367	0037407643	1427039655
C1QTNF8	C1q and TNF related 8	HGNC:31374	16p13.3	1019304522	0001345599	2871084451
C4BPA	complement component 4 binding protein alpha	HGNC:1325	1q32.2	8464404551	0026006752	1584913876
CA1	carbonic anhydrase 1	HGNC:1368	8q21.2	5385763258	0004501639	2346629318
CA6	carbonic anhydrase 6	HGNC:1380	1p36.23	3259887579	0008391643	2076153021
CASP6	caspase 6	HGNC:1507	4q25	616184837	0032220972	1491861366
CCR8	C-C motif chemokine receptor 8	HGNC:1609	3p22.1	6754418004	003041053	1516976012
CD5L	CD5 molecule like	HGNC:1690	1q23.1	2349722226	0011327494	194586615
CEACAM3	CEA cell adhesion molecule 3	HGNC:1815	19q13.2	1039273176	0022312472	1651452302
CFHR3	complement factor H related 3	HGNC:16980	1q31.3	5755701423	0033884621	1469997367
CHRNA1	cholinergic receptor nicotinic alpha 1 subunit	HGNC:1955	2q31.1	4422125293	000599403	2222281056
CNDP1	carnosine dipeptidase 1	HGNC:20675	18q22.3	1215468887	0019719137	1705112099
COL22A1	collagen type XXII alpha 1 chain	HGNC:22989	8q24.23-q24.3	4084785101	0042447522	1372147661
CPT1B	carnitine palmitoyltransferase 1B	HGNC:2329	22q13.33	2470553564	0010691393	1970965704
CST5	cystatin D	HGNC:2477	20p11.21	3380572874	0049395704	1306310822
CTSH	cathepsin H	HGNC:2535	15q25.1	2957035302	0009345794	2029383778
CXCL16	C-X-C motif chemokine ligand 16	HGNC:16642	17p13.2	3146782157	0008758624	2057564114
CXCR5	C-X-C motif chemokine receptor 5	HGNC:1060	11q23.3	650202224	003126682	1504916287
CYP24A1	cytochrome P450 family 24 subfamily A member 1	HGNC:2602	20q13.2	1369859033	0017761902	175051052
CYP2W1	cytochrome P450 family 2 subfamily W member 1	HGNC:20243	7p22.3	1454641344	367E-04	3435355882
DAND5	DAN domain BMP antagonist family member 5	HGNC:26780	19p13.13	2864992174	0009345794	2029383778
DCN	decorin	HGNC:2705	12q21.33	1354177663	0018128884	1741628933
DKK1	dickkopf WNT signaling pathway inhibitor 1	HGNC:2891	10q21.1	4450674334	0040294564	1394753541
DKK4	dickkopf WNT signaling pathway inhibitor 4	HGNC:2894	8p11.21	754963855	0027988452	1553021116
DMD	dystrophin	HGNC:2928	Xp21.2-p21.1	1785252211	0014874982	1827543561
ECE1	endothelin converting enzyme 1	HGNC:3146	1p36.12	3528266083	0007510887	2124308765
EFEMP1	EGF containing fibulin extracellular matrix protein 1	HGNC:3218	2p16.1	5082307833	0005015413	229969328
ELANE	elastase, neutrophil expressed	HGNC:3309	19p13.3	5971114156	0033101727	1480149344
ENPP1	ectonucleotide pyrophosphatase/phosphodiesterase 1	HGNC:3356	6q23.2	8858806179	0025199393	1598609916
EPHB3	EPH receptor B3	HGNC:3394	3q27.1	3220551933	0008587366	2066140024
ESAM	endothelial cell adhesion molecule	HGNC:17474	11q24.2	1106400126	0021284924	1671927888
F2RL1	F2R like trypsin receptor 1	HGNC:3538	5q13.3	805179697	0026520526	1576417858
FBLN5	fibulin 5	HGNC:3602	14q32.12	3438009916	0007682145	2114517493
FGF22	fibroblast growth factor 22	HGNC:3679	19p13.3	4300228722	004122425	1384847235
FKBP14	FKBP prolyl isomerase 14	HGNC:18625	7p14.3	7769526968	0027425747	1561841528
GALK1	galactokinase 1	HGNC:4118	17q25.1	4850411796	0038288398	1416932799
GGT6	gamma-glutamyltransferase 6	HGNC:26891	17p13.2	7377477169	0002397612	2620221065
GLP2R	glucagon like peptide 2 receptor	HGNC:4325	17p13.1	2429996363	0011156236	1952482298
GLRA3	glycine receptor alpha 3	HGNC:4328	4q34.1	9310451583	0024563292	1609713428
GLRA4	glycine receptor alpha 4 (pseudogene)	HGNC:31715	Xq22.2	1751911967	0015144101	1819756492
GPC4	glypican 4	HGNC:4452	Xq26.2	3843716209	0006727993	2172114447
GPR3	G protein-coupled receptor 3	HGNC:4484	1p36.11	6190308222	003212311	1493182415
GPR37L1	G protein-coupled receptor 37 like 1	HGNC:14923	1q32.1	9707398108	0001345599	2871084451
GRIA3	glutamate ionotropic receptor AMPA type subunit 3	HGNC:4573	Xq25	2547439459	0010642462	1972957884
GUSB	glucuronidase beta	HGNC:4696	7q11.21	4510090635	0039927582	1398726986
HDGF	heparin binding growth factor	HGNC:4856	1q23.1	3574473746	0046998092	1327919776
HPSE2	heparanase 2 (inactive)	HGNC:18374	10q24.2	6256444536	0031976317	1495171553
HRC	histidine rich calcium binding protein	HGNC:5178	19q13.33	3695698605	0045799286	1339141296
HTR1B	5-hydroxytryptamine receptor 1B	HGNC:5287	6q14.1	9179475443	0024685619	1607555974
ICMT	isoprenylcysteine carboxyl methyltransferase	HGNC:5350	1p36.31	6968725345	0029529774	152973987
IL11RA	interleukin 11 receptor subunit alpha	HGNC:5967	9p13.3	6512600288	0031242355	1505256243
IL20RB	interleukin 20 receptor subunit beta	HGNC:6004	3q22.3	1010307037	002302197	1637857517
IL3	interleukin 3	HGNC:6011	5q31.1	4100967465	0042325195	1373401037
IL32	interleukin 32	HGNC:16830	16p13.3	4423800642	0040490287	1392649142
ITGAX	integrin subunit alpha X	HGNC:6152	16p11.2	1515357121	0016881147	177259805
KCNK15	potassium two pore domain channel subfamily K member 15	HGNC:13814	20q13.12	4668315893	0039169154	1407055809
KLHL1	kelch like family member 1	HGNC:6352	13q21.33	1607401518	0016024857	1795205841
KLHL18	kelch like family member 18	HGNC:29120	3p21.31	1816697646	0014654793	1834020318
KLK10	kallikrein related peptidase 10	HGNC:6358	19q13.41	1451623322	0017419386	1758967147
KNG1	kininogen 1	HGNC:6383	3q27.3	4990022861	0037603366	1424773273
LPL	lipoprotein lipase	HGNC:6677	8p21.3	1259423359	0019009639	1721026122
MAP3K1	mitogen-activated protein kinase kinase kinase 1	HGNC:6848	5q11.2	4349268863	00409062	1388210867
MAP3K19	mitogen-activated protein kinase kinase kinase 19	HGNC:26249	2q21.3	3816534721	0006776924	2168967372
MAP3K3	mitogen-activated protein kinase kinase kinase 3	HGNC:6855	17q23.3	5953280236	0033150658	1479507845
MAP3K5	mitogen-activated protein kinase kinase kinase 5	HGNC:6857	6q23.3	4172728774	0041909282	1377689778
MAP4K2	mitogen-activated protein kinase kinase kinase kinase 2	HGNC:6864	11q13.1	3539674012	0047389539	132431752
MAP4K4	mitogen-activated protein kinase kinase kinase kinase 4	HGNC:6866	2q11.2	6485720461	0031315751	1504237171
MASP1	mannan binding lectin serine peptidase 1	HGNC:6901	3q27.3	7502034706	0028012918	1552641654
MDM2	MDM2 proto-oncogene	HGNC:6973	12q15	8665116798	0001761511	2754114644
MET	MET proto-oncogene, receptor tyrosine kinase	HGNC:7029	7q31	38852002	0044184567	1354729395
MMP14	matrix metallopeptidase 14	HGNC:7160	14q11.2	580741427	0033639967	1473144442
MMP25	matrix metallopeptidase 25	HGNC:14246	16p13.3	5715449891	0004036796	2393963196
MSTN	myostatin	HGNC:4223	2q32.2	4214727213	0006214219	2206613424
MYH7	myosin heavy chain 7	HGNC:7577	14q11.2	447386694	004009884	1396868187
NCSTN	nicastrin	HGNC:17091	1q23.2	6020569691	0032832608	1483694625
NEGR1	neuronal growth regulator 1	HGNC:17302	1p31.1	2130160481	0012844351	1891287837
NGF	nerve growth factor	HGNC:7808	1p13.2	6136258972	0032269903	1491202345
OC90	otoconin 90	HGNC:8100	8q24.22	142292473	0017492783	1757141099
OVGP1	oviductal glycoprotein 1	HGNC:8524	1p13.2	5118716246	0036918334	1432757901

KNOWN MYCOBCATERIAL REGULATION

Gene	Gene Name	HGNC ID	Location	FC	*p* value	-Log10(*p* value)
ABCD4	ATP binding cassette subfamily D member 4	HGNC:68	14q24.3	513230236	0004990948	2301816973
ACAD8	acyl-CoA dehydrogenase family member 8	HGNC:87	11q25	167689087	0015731272	1803236168
ACSL1	acyl-CoA synthetase long chain family member 1	HGNC:3569	4q35.1	405707005	0006434408	2191491392
ADRA1A	adrenoceptor alpha 1A	HGNC:277	8p21.2	141888416	0017517248	1756534118
ALDH18A1	aldehyde dehydrogenase 18 family member A1	HGNC:9722	10q24.1	633628792	0003278368	2484342342
ARHGEF12	Rho guanine nucleotide exchange factor 12	HGNC:14193	11q23.3	414655794	0042031609	1376423981
ARL8B	ADP ribosylation factor like GTPase 8B	HGNC:25564	3p26.1	119760651	979E-04	3009387149
ATF3	activating transcription factor 3	HGNC:785	1q32.3	454270837	0039756324	1400593775
ATG7	autophagy related 7	HGNC:16935	3p25.3	559212761	0034545188	1461612444
BANK1	B cell scaffold protein with ankyrin repeats 1	HGNC:18233	4q24	631696299	0031756129	1498172448
BATF2	basic leucine zipper ATF-like transcription factor 2	HGNC:25163	11q13.1	353448052	0047414004	1324093368
BIRC3	baculoviral IAP repeat containing 3	HGNC:591	11q22.2	120727688	0019816999	1702962122
C1QBP	complement C1q binding protein	HGNC:1243	17p13.2	960369054	002387826	1621997323
CASP6	caspase 6	HGNC:1507	4q25	616184837	0032220972	1491861366
CCR8	C-C motif chemokine receptor 8	HGNC:1609	3p22.1	6754418	003041053	1516976012
CD5L	CD5 molecule like	HGNC:1690	1q23.1	234972223	0011327494	194586615
CDH10	cadherin 10	HGNC:1749	5p14.2-p14.1	570622552	0034031414	1468120011
CDH24	cadherin 24	HGNC:14265	14q11.2	518846354	0036526888	1437387333
CFHR3	complement factor H related 3	HGNC:16980	1q31.3	575570142	0033884621	1469997367
CHRNA1	cholinergic receptor nicotinic alpha 1 subunit	HGNC:1955	2q31.1	442212529	000599403	2222281056
CLDN1	claudin 1	HGNC:2032	3q28	134572607	0018128884	1741628933
CLEC4A	C-type lectin domain family 4 member A	HGNC:13257	12p13.31	225016477	0012183784	1914217798
CNDP1	carnosine dipeptidase 1	HGNC:20675	18q22.3	121546889	0019719137	1705112099
CORO2A	coronin 2A	HGNC:2255	9q22.33	544074663	0034863238	1457632276
CPLX2	complexin 2	HGNC:2310	5q35.2	477743245	0038704311	1412240661
CPT1B	carnitine palmitoyltransferase 1B	HGNC:2329	22q13.33	247055356	0010691393	1970965704
CREM	cAMP responsive element modulator	HGNC:2352	10p11.21	625052694	0032049714	1494175845
CTSH	cathepsin H	HGNC:2535	15q25.1	29570353	0009345794	2029383778
CXCL16	C-X-C motif chemokine ligand 16	HGNC:16642	17p13.2	314678216	0008758624	2057564114
DHCR24	24-dehydrocholesterol reductase	HGNC:2859	1p32.3	87405589	0001688115	277259805
ELANE	elastase, neutrophil expressed	HGNC:3309	19p13.3	597111416	0033101727	1480149344
ELMO2	engulfment and cell motility 2	HGNC:17233	20q13.12	886709776	0001639184	2785372338
ENPP1	ectonucleotide pyrophosphatase/phosphodiesterase 1	HGNC:3356	6q23.2	885880618	0025199393	1598609916
EPSTI1	epithelial stromal interaction 1	HGNC:16465	13q14.11	537336424	0035352547	1451579294
FAM180B	family with sequence similarity 180 member B	HGNC:34451	11p11.2	863146508	0001785976	274812428
FBLN5	fibulin 5	HGNC:3602	14q32.12	343800992	0007682145	2114517493
FTH1	ferritin heavy chain 1	HGNC:3976	11q12.3	187156278	0014116553	1850271327
FZD1	frizzled class receptor 1	HGNC:4038	7q21.13	519360156	0036257768	1440598937
GAL3ST3	galactose-3-O-sulfotransferase 3	HGNC:24144	11q13.1	12177049	0019719137	1705112099
GALK1	galactokinase 1	HGNC:4118	17q25.1	48504118	0038288398	1416932799
GATA4	GATA binding protein 4	HGNC:4173	8p23.1	576521701	0033762294	1471568054
GRIA3	glutamate ionotropic receptor AMPA type subunit 3	HGNC:4573	Xq25	254743946	0010642462	1972957884
GUSB	glucuronidase beta	HGNC:4696	7q11.21	451009064	0039927582	1398726986
HAUS4	HAUS augmin like complex subunit 4	HGNC:20163	14q11.2	165096491	0015804668	1801214623
IL11RA	interleukin 11 receptor subunit alpha	HGNC:5967	9p13.3	651260029	0031242355	1505256243
IL20RB	interleukin 20 receptor subunit beta	HGNC:6004	3q22.3	101030704	002302197	1637857517
IL3	interleukin 3	HGNC:6011	5q31.1	410096746	0042325195	1373401037
IL32	interleukin 32	HGNC:16830	16p13.3	442380064	0040490287	1392649142
INPP4A	inositol polyphosphate-4-phosphatase type I A	HGNC:6074	2q11.2	373912886	0045432304	1342635237
IRF8	interferon regulatory factor 8	HGNC:5358	16q24.1	929152144	0024612223	160884916
ITGAX	integrin subunit alpha X	HGNC:6152	16p11.2	151535712	0016881147	177259805
KTN1	kinectin 1	HGNC:6467	14q22.1	460116467	0039633997	1401932126
MAP3K3	mitogen-activated protein kinase kinase kinase 3	HGNC:6855	17q23.3	595328024	0033150658	1479507845
MASP1	mannan binding lectin serine peptidase 1	HGNC:6901	3q27.3	750203471	0028012918	1552641654
MRPL3	mitochondrial ribosomal protein L3	HGNC:10379	3q22.1	380631482	0006874786	2162740821
MS4A14	membrane spanning 4-domains A14	HGNC:30706	11q12.2	701037865	0029382982	1531904133
MSTN	myostatin	HGNC:4223	2q32.2	421472721	0006214219	2206613424
MYO5B	myosin VB	HGNC:7603	18q	387019348	0044257963	1354008574
NEBL	nebulette	HGNC:16932	10p12.31	393495755	0043866517	1357866851
NINJ1	ninjurin 1	HGNC:7824	9q22.31	905582783	0024881343	1604126188
NXPE4	neurexophilin and PC-esterase domain family member 4	HGNC:23117	11q23.2	627233688	0031780594	149783799
OSGIN1	oxidative stress induced growth inhibitor 1	HGNC:30093	16q23.3	409250022	0042447522	1372147661
P2RX2	purinergic receptor P2X 2	HGNC:15459	12q24.33	848798708	0025933356	1586141275
PCDH12	protocadherin 12	HGNC:8657	5q31.3	971391622	0023682537	1625571783
PDGFRA	platelet derived growth factor receptor alpha	HGNC:8803	4q12	589746344	0003327299	2477908232
PLG	plasminogen	HGNC:9071	6q26	348166661	0007608749	2118686752
PLIN2	perilipin 2	HGNC:248	9p22.1	263456872	0010177619	199235381
POLR2G	RNA polymerase II subunit G	HGNC:9194	11q12.3	122307889	0019719137	1705112099
PPP2R5A	protein phosphatase 2 regulatory subunit B'alpha	HGNC:9309	1q32.3	28497339	0009492587	2022615415
PTGES2	prostaglandin E synthase 2	HGNC:17822	9q34.11	815855363	0002030631	2692369048
QPRT	quinolinate phosphoribosyltransferase	HGNC:9755	16p11.2	194873753	0013749572	1861710825
RAB33A	RAB33A, member RAS oncogene family	HGNC:9773	Xq26.1	257420507	0010324412	198613469
RAB3C	RAB3C, member RAS oncogene family	HGNC:30269	5q11.2	617717487	003212311	1493182415
RGS3	regulator of G protein signaling 3	HGNC:9999	9q32	60251923	0032783677	1484342342
RNF175	ring finger protein 175	HGNC:27735	4q31.3	441426745	000599403	2222281056
SAMD11	sterile alpha motif domain containing 11	HGNC:28706	1p36.33	755983174	0027988452	1553021116
SARM1	sterile alpha and TIR motif containing 1	HGNC:17074	17q11.2	117618437	0020086118	1697103983
SCCPDH	saccharopine dehydrogenase (putative)	HGNC:24275	1q44	78602998	0027205559	1565342353
SCNN1D	sodium channel epithelial 1 subunit delta	HGNC:10601	1p36.33	108817848	0021676371	1664013419
SCPEP1	serine carboxypeptidase 1	HGNC:29507	17q22	573559675	0033933552	146937068
SDC1	syndecan 1	HGNC:10658	2p24.1	87286175	0025395117	1595249787
SDC4	syndecan 4	HGNC:10661	20q13.12	365346797	0045799286	1339141296
SERPINA1	serpin family A member 1	HGNC:8941	14q32.13	598602238	00329794	1481757248
SERPINB2	serpin family B member 2	HGNC:8584	18q21.33-q22.1	397081951	0006679062	2175284494
SFXN4	sideroflexin 4	HGNC:16088	10q26.11	183297619	0014483535	1839125434
SHISAL1	shisa like 1	HGNC:29335	22q13.31	554672056	0034667515	146007729
SIN3B	SIN3 transcription regulator family member B	HGNC:19354	19p13.11	115675107	0001052014	2977978685
SLC25A34	solute carrier family 25 member 34	HGNC:27653	1p36.21	512096578	0036893869	1433045799
SLC39A7	solute carrier family 39 member 7	HGNC:4927	6p21.32	725522093	0028624554	1543261279
SLC6A6	solute carrier family 6 member 6	HGNC:11052	3p25.1	111434826	0020673289	1684590432
SLFN11	schlafen family member 11	HGNC:26633	17q12	11038535	0021431717	1668943034
SNTB1	syntrophin beta 1	HGNC:11168	8q24.12	876425474	0025199393	1598609916

XENOBIOTIC RESPONSE GO TERMS GROUP

Gene	Gene Name	HGNC ID	Location	FC	*p* value	-Log10(*p* value)
BANK1	B cell scaffold protein with ankyrin repeats 1	HGNC:18233	4q24	6316962986	00317561	1498172448
BCL6B	BCL6B transcription repressor	HGNC:1002	17p13.1	7720144365	00020796	2682028215
CCS	copper chaperone for superoxide dismutase	HGNC:1613	11q13.2	439374414	00406615	1390816121
CDH10	cadherin 10	HGNC:1749	5p14.2-p14.1	570622552	00340314	1468120011
CYP2W1	cytochrome P450 family 2 subfamily W member 1	HGNC:20243	7p22.3	1454641344	367E-04	3435355882
GLRA3	glycine receptor alpha 3	HGNC:4328	4q34.1	9310451583	00245633	1609713428
GLRA4	glycine receptor alpha 4 (pseudogene)	HGNC:31715	Xq22.2	1751911967	00151441	1819756492
HTR1B	5-hydroxytryptamine receptor 1B	HGNC:5287	6q14.1	9179475443	00246856	1607555974
IL20RB	interleukin 20 receptor subunit beta	HGNC:6004	3q22.3	1010307037	0023022	1637857517
MAP4K4	mitogen-activated protein kinase kinase kinase kinase 4	HGNC:6866	2q11.2	6485720461	00313158	1504237171
NGF	nerve growth factor	HGNC:7808	1p13.2	6136258972	00322699	1491202345
SLFN11	schlafen family member 11	HGNC:26633	17q12	11038535	00214317	1668943034
TAOK3	TAO kinase 3	HGNC:18133	12q24.23	3563437089	0007462	2127147301
TLR8	toll like receptor 8	HGNC:15632	Xp22.2	8504140759	00258355	1587783222
TNIP2	TNFAIP3 interacting protein 2	HGNC:19118	4p16.3	3003762738	00090522	2043245417
UBASH3A	ubiquitin associated and SH3 domain containing A	HGNC:12462	21q22.3	5359684177	00354259	1450678579

P2RX2	purinergic receptor P2X 2	HGNC:15459	12q24.33	8487987082	0025933356	1586141275	SOAT1	sterol O-acyltransferase 1	HGNC:11177	1q25.2	425841984	0041542301	1381509455
PADI1	peptidyl arginine deiminase 1	HGNC:18367	1p36.13	3436403389	0007755541	2110387878	SOX18	SRY-box transcription factor 18	HGNC:11194	20q13.33	562596953	0004110192	2386137859
PDE5A	phosphodiesterase 5A	HGNC:8784	4q26	3527026681	0047438469	1323869332	SPIB	Spi-B transcription factor	HGNC:11242	19q13.33	386362765	0044257963	1354008574
PDGFRA	platelet derived growth factor receptor alpha	HGNC:8803	4q12	5897463445	0003327299	2477908232	SPOCK1	SPARC (osteonectin), cwcv and kazal like domains proteoglycan 1	HGNC:11251	5q31.2	744074953	0002275285	2642964192
PENK	proenkephalin	HGNC:8831	8q12.1	3419115151	0007755541	2110387878	TAOK3	TAO kinase 3	HGNC:18133	12q24.23	356343709	0007461956	2127147301
PKD2	polycystin 2, transient receptor potential cation channel	HGNC:9009	4q22.1	1357483085	0018104418	1742215421	TENT4A	terminal nucleotidyltransferase 4A	HGNC:16705	5p15.31	440637558	000599403	2222281056
PKD2L2	polycystin 2 like 2, transient receptor potential cation channel	HGNC:9012	5q31.2	4475857141	0005578118	2253512294	TFAM	transcription factor A, mitochondrial	HGNC:11741	10q21.1	50781829	0037065127	1431034508
PLG	plasminogen	HGNC:9071	6q26	3481666613	0007608749	2118686752	TRAF3	TNF receptor associated factor 3	HGNC:12033	14q32.32	390680596	0043866517	1357866851
PLIN2	perilipin 2	HGNC:248	9p22.1	2634568721	0010177619	199235381	TXNIP	thioredoxin interacting protein	HGNC:16952	1q21.1	931905837	0024343103	161362406
PLTP	phospholipid transfer protein	HGNC:9093	20q13.12	2709516949	0009517052	2021497539	VAMP5	vesicle associated membrane protein 5	HGNC:12646	2p11.2	25088208	489E-05	4310417145
PNLIPRP2	pancreatic lipase related protein 2 (gene/pseudogene)	HGNC:9157	10q25.3	5493089391	000428145	2368409092	XDH	xanthine dehydrogenase	HGNC:12805	2p23.1	907933333	0024856877	1604553433
PSMB10	proteasome 20S subunit beta 10	HGNC:9538	16q22.1	7892964359	0027034301	1568084863							
PSPN	persephin	HGNC:9579	19p13.3	176616751	0014948378	182540593							
PTPRG	protein tyrosine phosphatase receptor type G	HGNC:9671	3p14.2	1094074747	0021480648	1667952625							
PXDN	peroxidasin	HGNC:14966	2p25.3	3744663055	0045358908	1343337411							
SCNN1D	sodium channel epithelial 1 subunit delta	HGNC:10601	1p36.33	1088178479	0021676371	1664013419							
SCPEP1	serine carboxypeptidase 1	HGNC:29507	17q22	5735596754	0033933552	146937068							
SDC1	syndecan 1	HGNC:10658	2p24.1	8728617496	0025395117	1595249787							
SDC4	syndecan 4	HGNC:10661	20q13.12	3653467966	0045799286	1339141296							
SENP6	SUMO specific peptidase 6	HGNC:20944	6q14.1	4565836889	0039658463	1401664126							
SERPINA1	serpin family A member 1	HGNC:8941	14q32.13	5986022379	00329794	1481757248							
SERPINA9	serpin family A member 9	HGNC:15995	14q32.13	7081126986	0029113862	1535900179							
SERPINB2	serpin family B member 2	HGNC:8584	18q21.33-q22.1	3970819507	0006679062	2175284494							
SIGLEC12	sialic acid binding Ig like lectin 12	HGNC:15482	19q13.41	1226338869	0019303225	1714370137							
SLC12A2	solute carrier family 12 member 2	HGNC:10911	5q23.3	7096660237	0002422078	2615811946							
SLC6A6	solute carrier family 6 member 6	HGNC:11052	3p25.1	1114348255	0020673289	1684590432							
SLCO5A1	solute carrier organic anion transporter family member 5A1	HGNC:19046	8q13.3	3457703716	0047952243	1319191069							
SLIT1	slit guidance ligand 1	HGNC:11085	10q24.1	6341224149	0003204971	2494175845							
SOAT1	sterol O-acyltransferase 1	HGNC:11177	1q25.2	4258419844	0041542301	1381509455							
SPINK1	serine peptidase inhibitor Kazal type 1	HGNC:11244	5q32	307120083	0009003278	2045599322							
SPOCK1	SPARC (osteonectin), cwcv and kazal like domains proteoglycan 1	HGNC:11251	5q31.2	7440749533	0002275285	2642964192							
SPOCK2	SPARC (osteonectin), cwcv and kazal like domains proteoglycan 2	HGNC:13564	10q22.1	3348241475	0049664824	1303951103							
SULT1A2	sulfotransferase family 1A member 2	HGNC:11454	16p11.2	5781016932	0033664432	1472828707							
TAOK3	TAO kinase 3	HGNC:18133	12q24.23	3563437089	0007461956	2127147301							
TG	thyroglobulin	HGNC:11764	8q24.22	1807807633	0014654793	1834020318							
TLR8	toll like receptor 8	HGNC:15632	Xp22.2	8504140759	0025835494	1587783222							
UBA2	ubiquitin like modifier activating enzyme 2	HGNC:30661	19q13.11	118101393	0020037187	1698163239							
VWF	von Willebrand factor	HGNC:12726	12p13.31	5575955782	0004183589	237845103							
WNK4	WNK lysine deficient protein kinase 4	HGNC:14544	17q21.2	1702964192	0015584479	1807307708							
WNT16	Wnt family member 16	HGNC:16267	7q31.31	6845841489	0030043548	1522248774							
XDH	xanthine dehydrogenase	HGNC:12805	2p23.1	907933333	0024856877	1604553433							

TABLE 4

GO TERMS GROUPS (REVIGO)	GO TERMS	FONCTION	HGNC	GENES
Cellular Import	GO:0051222	positive regulation of protein transport	HGNC=22201	TCAF1
Cellular Import	GO:0006836	neurotransmitter transport	HGNC=20151	SLC17A8
Cellular Import	GO:0006829	zinc II ion transport	HGNC=4927	SLC39A7
Cellular Import	GO:1990126	retrograde transport, endosome to plasma membrane	HGNC=20073 HGNC=18708	SNX27 , GRIP1
Cellular Import	GO:0006855	drug transmembrane transport	HGNC=11061	SLC7A3
Cellular Import	GO:0006869	lipid transport	HGNC=36	ABCA6
Cellular Import	GO:0006890	retrograde vesicle-mediated transport, Golgi to ER	HGNC=18622 HGNC=29205	COG7 , ERGIC1
Cellular Import	GO:0071705	nitrogen compound transport	HGNC=11052	SLC6A6
Cellular Import	GO:1990778	protein localization to cell periphery	HGNC=4452	GPC4
Cellular Import	GO:0016197	endosomal transport	HGNC=25104	AP5B1
Cellular Import	GO:0017157	regulation of exocytosis	HGNC=30269	RAB3C
Cellular Import	GO:0099504	synaptic vesicle cycle	HGNC=20151	SLC17A8
Cellular Import	GO:0099500	vesicle fusion to plasma membrane	HGNC=30269 HGNC=2310	RAB3C , CPLX2
Cellular Import	GO:0098657	import into cell	HGNC=11061 HGNC=10911 HGNC=14544	SLC7A3 , SLC12A2 , WNK4
Cellular Import	GO:0008298	intracellular mRNA localization	HGNC=17351	PRPF18
Cellular Import	GO:0072657	protein localization to membrane	HGNC=17359 HGNC=4452	NUP54 , GPC4
Cellular Import	GO:0006606	protein import into nucleus	HGNC=15913 HGNC=9851	NXT1 , RANBP6
Cellular Import	GO:0042886	amide transport	HGNC=10919 HGNC=9093	SLC14A2 , PLTP
Cellular Import	GO:0097120	receptor localization to synapse	HGNC=4452	GPC4
Cellular Import	GO:0015802	basic amino acid transport	HGNC=23090	SLC15A4
Cellular Import	GO:0015809	arginine transport	HGNC=11061	SLC7A3
Cellular Import	GO:0015849	organic acid transport	HGNC=11052	SLC6A6
Cellular Import	GO:0051051	negative regulation of transport	HGNC=14544	WNK4
Cellular Import	GO:0006623	protein targeting to vacuole	HGNC=1908	VPS13A
Cellular Import	GO:0042908	xenobiotic transport	HGNC=11052	SLC6A6
Cellular Import	GO:0034067	protein localization to Golgi apparatus	HGNC=1908	VPS13A
Cellular Import	GO:0009306	protein secretion	HGNC=30269	RAB3C
Cellular Import	GO:1990542	mitochondrial transmembrane transport	HGNC=29786 HGNC=16088	SLC25A37 , SFXN4
Cellular Import	GO:0061512	protein localization to cilium	HGNC=21057 HGNC=19009 HGNC=25705	RSPH9 , CLUAP1 , KCTD17
Cellular Import	GO:1902476	chloride transmembrane transport	HGNC=10911 HGNC=4328 HGNC=31715	SLC12A2 , GLRA3 , GLRA4
Cellular Import	GO:0015698	inorganic anion transport	HGNC=22201 HGNC=3356	TCAF1 , ENPP1
Cellular Import	GO:0048193	Golgi vesicle transport	HGNC=19347	CUX2
Cellular Import	GO:0006909	phagocytosis	HGNC=7029 HGNC=9802 HGNC=3309	MET , RAC2 , ELANE
Cellular Import	GO:0043266	regulation of potassium ion transport	HGNC=14544	WNK4
Cellular Import	GO:0051640	organelle localization	HGNC=30269	RAB3C
Membrane Invagination	GO:0032273	positive regulation of protein polymerization	HGNC=24164	TPPP
Membrane Invagination	GO:0051260	protein homooligomerization	HGNC=12968	RNF112
Membrane Invagination	GO:0051276	chromosome organization	HGNC=20944	SENP6
Membrane Invagination	GO:0010639	negative regulation of organelle organization	HGNC=18165	STYXL1
Membrane Invagination	GO:0010638	positive regulation of organelle organization	HGNC=25705	KCTD17
Membrane Invagination	GO:0043062	extracellular structure organization	HGNC=9093	PLTP
Membrane Invagination	GO:0097435	supramolecular fiber organization	HGNC=3602	FBLN5
Membrane Invagination	GO:0030198	extracellular matrix organization	HGNC=14246 HGNC=3602 HGNC=7160 HGNC=11345	MMP25 , FBLN5 , MMP14 , RECK
Membrane Invagination	GO:0000422	mitophagy	HGNC=25072	WDR45B
Membrane Invagination	GO:0099560	synaptic membrane adhesion	HGNC=4452	GPC4
Membrane Invagination	GO:0044089	positive regulation of cellular component biogenesis	HGNC=3420 HGNC=25705 HGNC=21295	EPS8 , KCTD17 , EPS8L1
Membrane Invagination	GO:0044087	regulation of cellular component biogenesis	HGNC=4452	GPC4
Membrane Invagination	GO:0065004	protein-DNA complex assembly	HGNC=20944	SENP6
Membrane Invagination	GO:0030865	cortical cytoskeleton organization	HGNC=9802	RAC2
Membrane Invagination	GO:0016233	telomere capping	HGNC=7652	NBN
Membrane Invagination	GO:1905349	ciliary transition zone assembly	HGNC=21057 HGNC=19009 HGNC=25705	RSPH9 , CLUAP1 , KCTD17
Membrane Invagination	GO:0010324	membrane invagination	HGNC=9782 HGNC=9802	RAB4B , RAC2
Membrane Invagination	GO:0032508	DNA duplex unwinding	HGNC=7652	NBN
Membrane Invagination	GO:0000045	autophagosome assembly	HGNC=25072	WDR45B
Membrane Invagination	GO:0016575	histone deacetylation	HGNC=23784 HGNC=19354	MTA3 , SIN3B
Membrane Invagination	GO:0001558	regulation of cell growth	HGNC=30093	OSGIN1
Membrane Invagination	GO:0007029	endoplasmic reticulum organization	HGNC=12968	RNF112
Membrane Invagination	GO:0007030	Golgi organization	HGNC=18622	COG7
Membrane Invagination	GO:0035735	intraciliary transport involved in cilium assembly	HGNC=21057 HGNC=19009 HGNC=25705	RSPH9 , CLUAP1 , KCTD17
Membrane Invagination	GO:0006998	nuclear envelope organization	HGNC=11214	SPAG4
Membrane Invagination	GO:0006997	nucleus organization	HGNC=17359	NUP54
Membrane Invagination	GO:0035082	axoneme assembly	HGNC=21057 HGNC=19009 HGNC=25705	RSPH9 , CLUAP1 , KCTD17
Membrane Invagination	GO:0097712	vesicle targeting, trans-Golgi to periciliary membrane compartment	HGNC=21057 HGNC=19009 HGNC=25705	RSPH9 , CLUAP1 , KCTD17
Membrane Invagination	GO:0030031	cell projection assembly	HGNC=9802	RAC2
Membrane Invagination	GO:0045216	cell-cell junction organization	HGNC=1749 HGNC=14265	CDH10 , CDH24
Membrane Invagination	GO:0090148	membrane fission	HGNC=25589	SEPTIN11
Membrane Invagination	GO:0008360	regulation of cell shape	HGNC=9802	RAC2
Cell Fate Commitment	GO:0007610	behavior	HGNC=7808	NGF
Cell Fate Commitment	GO:0051301	cell division	HGNC=26624	KDF1
Cell Fate Commitment	GO:0042692	muscle cell differentiation	HGNC=19098	RBPMS2
Cell Fate Commitment	GO:0001708	cell fate specification	HGNC=6132 HGNC=11598 HGNC=11597	ISL1 , TBX20 , TBX2
Cell Fate Commitment	GO:0048747	muscle fiber development	HGNC=16932	NEBL
Cell Fate Commitment	GO:0006939	smooth muscle contraction	HGNC=277	ADRA1A
Cell Fate Commitment	GO:0003009	skeletal muscle contraction	HGNC=11353	STAC
Cell Fate Commitment	GO:0001501	skeletal system development	HGNC=7160	MMP14
Cell Fate Commitment	GO:0007283	spermatogenesis	HGNC=25929	MAEL
Cell Fate Commitment	GO:0045165	cell fate commitment	HGNC=4173 HGNC=16267	GATA4 , WNT16
Cell Fate Commitment	GO:0043588	skin development	HGNC=3052	DSP
Cell Fate Commitment	GO:0030282	bone mineralization	HGNC=3356	ENPP1
Cell Fate Commitment	GO:0014033	neural crest cell differentiation	HGNC=2254	CORO1C
Cell Fate Commitment	GO:0045446	endothelial cell differentiation	HGNC=243 HGNC=22989	ADD1 , COL22A1
Cell Fate Commitment	GO:0007606	sensory perception of chemical stimulus	HGNC=15249	OR5A2
Cell Fate Commitment	GO:0007140	male meiosis	HGNC=25929	MAEL
Cell Fate Commitment	GO:0045664	regulation of neuron differentiation	HGNC=7808	NGF
Cell Fate Commitment	GO:0051960	regulation of nervous system development	HGNC=4452	GPC4
Cell Fate Commitment	GO:0031016	pancreas development	HGNC=7029	MET
Cell Fate Commitment	GO:0007422	peripheral nervous system development	HGNC=7808	NGF
Cell Fate Commitment	GO:0048732	gland development	HGNC=7029	MET
Response to Xenobiotic Stimulus	GO:0001816	cytokine production	HGNC:15632	**TLR8**
Response to Xenobiotic Stimulus	GO:0050727	regulation of inflammatory response	HGNC:1002	BCL6B
Response to Xenobiotic Stimulus	GO:0002224	toll-like receptor signaling pathway	HGNC:19118 HGNC:15632	TNIP2 , **TLR8**
Response to Xenobiotic Stimulus	GO:0051607	defense response to virus	HGNC:15632 HGNC:26633	**TLR8** , SLFN11
Response to Xenobiotic Stimulus	GO:0006954	inflammatory response	HGNC=1002	BCL6B
Response to Xenobiotic Stimulus	GO:0031098	stress-activated protein kinase signaling cascade	HGNC=18133 HGNC=6866	TAOK3 , MAP4K4
Response to Xenobiotic Stimulus	GO:0042113	B cell activation	HGNC=18233	BANK1
Response to Xenobiotic Stimulus	GO:0050852	T cell receptor signaling pathway	HGNC=12462	UBASH3A
Response to Xenobiotic Stimulus	GO:0002683	negative regulation of immune system process	HGNC=12462	UBASH3A
Response to Xenobiotic Stimulus	GO:0009410	response to xenobiotic stimulus	HGNC=20243	CYP2W1
Response to Xenobiotic Stimulus	GO:0043200	response to amino acid	HGNC=4328 HGNC=31715	GLRA3 , GLRA4
Response to Xenobiotic Stimulus	GO:0000302	response to reactive oxygen species	HGNC=1613	CCS
Response to Xenobiotic Stimulus	GO:0042493	response to drug	HGNC=5287 HGNC=20243	HTR1B , CYP2W1
Response to Xenobiotic Stimulus	GO:0019221	cytokine-mediated signaling pathway	HGNC=6004	IL20RB
Response to Xenobiotic Stimulus	GO:0009636	response to toxic substance	HGNC=1613	CCS
Response to Xenobiotic Stimulus	GO:0071363	cellular response to growth factor stimulus	HGNC=7808 HGNC=174	NGF , CDH10
Response to Xenobiotic Stimulus	GO:0071407	cellular response to organic cyclic compound	HGNC=5287	HTR1B
Response to Xenobiotic Stimulus	GO:0010035	response to inorganic substance	HGNC=1613	CCS
Response to Xenobiotic Stimulus	GO:0071417	cellular response to organonitrogen compound	HGNC=5287	HTR1B
RNA Processing	GO:0090503	RNA phosphodiester bond hydrolysis, exonucleolytic	HGNC=17035	EXOSC8
RNA Processing	GO:0006367	transcription initiation from RNA polymerase II promoter	HGNC=9194	POLR2G
RNA Processing	GO:0016071	mRNA metabolic process	HGNC=16060	TRUB1
RNA Processing	GO:0000398	mRNA splicing, via spliceosome	HGNC=9900 HGNC=17351	RBM3 , PRPF18
RNA Processing	GO:0034472	snRNA 3'-end processing	HGNC=17035	EXOSC8
RNA Processing	GO:0000466	maturation of 5.8S rRNA from tricistronic rRNA transcript (SSU-rRNA, 5.8S rRNA, LSU-rRNA)	HGNC=17035	EXOSC8
RNA Processing	GO:0002181	cytoplasmic translation	HGNC=21746	CPEB3
RNA Processing	GO:0032543	mitochondrial translation	HGNC=1243	C1QBP
RNA Processing	GO:0006575	cellular modified amino acid metabolic process	HGNC=2329	CPT1B
RNA Processing	GO:0006413	translational initiation	HGNC=9194	POLR2G
RNA Processing	GO:0006414	translational elongation	HGNC=14027 HGNC=1243 HGNC=7234 HGNC=9900 HGNC=21746 HGNC=9194	MRPL39 , C1QBP , MRRF , RBM3 , CPEB3 , POLR2G
RNA Processing	GO:0006399	tRNA metabolic process	HGNC=14027 HGNC=16219 HGNC=17035	MRPL39 , DTD1 , EXOSC8
RNA Processing	GO:0072521	purine-containing compound metabolic process	HGNC=12805	XDH
RNA Processing	GO:0031123	RNA 3'-end processing	HGNC=16705	TENT4A
RNA Processing	GO:0000737	DNA catabolic process, endonucleolytic	HGNC=2957	DNASE1L1
RNA Processing	GO:0034660	ncRNA metabolic process	HGNC=25929	MAEL
Intracellular Signalling	GO:0035249	synaptic transmission, glutamatergic	HGNC=20151	SLC17A8
Intracellular Signalling	GO:0048016	inositol phosphate-mediated signaling	HGNC=3538 HGNC=277	F2RL1 , ADRA1
Intracellular Signalling	GO:0016055	Wnt signaling pathway	HGNC=16267	WNT16
Intracellular Signalling	GO:0007265	Ras protein signal transduction	HGNC=21165 HGNC=9768	RAP2C , RAB28
Intracellular Signalling	GO:0050804	modulation of synaptic transmission	HGNC=7808	NGF
Intracellular Signalling	GO:0043123	positive regulation of I-kappaB kinase/NF-kappaB signaling	HGNC=19118	TNIP2

Intracellular Signalling	GO:0007249	I-kappaB kinase/NF-kappaB signaling	HGNC=19118 HGNC=15632 HGNC=12033	TNIP2 , **TLR8** , TRAF3
Intracellular Signalling	GO:0007200	phospholipase C-activating G-protein coupled receptor signaling pathway	HGNC=3538 HGNC=277	F2RL1 , ADRA1
Intracellular Signalling	GO:0007193	adenylate cyclase-inhibiting G-protein coupled receptor signaling pathway	HGNC=14923 HGNC=5287	GPR37L1 , HTR1B
Intracellular Signalling	GO:0007218	neuropeptide signaling pathway	HGNC=8831 HGNC=4328 HGNC=31715	PENK , GLRA3 , GLRA4
Intracellular Signalling	GO:0051896	regulation of protein kinase B signaling	HGNC=18233	BANK1
Intracellular Signalling	GO:0007169	transmembrane receptor protein tyrosine kinase signaling pathway	HGNC=7808	NGF
Intracellular Signalling	GO:0007187	G-protein coupled receptor signaling pathway, coupled to cyclic nucleotide second messenger	HGNC=15296	OR5T2
Intracellular Signalling	GO:0043491	protein kinase B signaling	HGNC=18233	BANK1
Intracellular Signalling	GO:1902532	negative regulation of intracellular signal transduction	HGNC=18233	BANK1
Catabolism process	GO:0031331	positive regulation of cellular catabolic process	HGNC=9194	POLR2G
Catabolism process	GO:0046395	carboxylic acid catabolic process	HGNC=9755 HGNC=4696	QPRT , GUSB
Catabolism process	GO:0044270	cellular nitrogen compound catabolic process	HGNC=9755 HGNC=12805 HGNC=25941	QPRT , XDH , TET2
Catabolism process	GO:0030433	ER-associated ubiquitin-dependent protein catabolic process	HGNC=27735 HGNC=14236	RNF175 , DERL3
Catabolism process	GO:0043632	modification-dependent macromolecule catabolic process	HGNC=17035 HGNC=17351	EXOSC8 , PRPF18
Catabolism process	GO:0046348	amino sugar catabolic process	HGNC=8524	OVGP1
Catabolism process	GO:1901565	organonitrogen compound catabolic process	HGNC=9755 HGNC=12805 HGNC=8524 HGNC=25941	QPRT1 , XDH , OVGP1 , TET2
Catabolism process	GO:0019439	aromatic compound catabolic process	HGNC=12805 HGNC=25941	XDH , TET2
Catabolism process	GO:0006022	aminoglycan metabolic process	HGNC=8524	OVGP1
Catabolism process	GO:0030166	proteoglycan biosynthetic process	HGNC=19721	CANT1
Catabolism process	GO:0044282	small molecule catabolic process	HGNC=14522 HGNC=4118	MIOX , GALK1
Catabolism process	GO:1901361	organic cyclic compound catabolic process	HGNC=9755 HGNC=12805 HGNC=25941	QPRT , XDH , TET2
Catabolism process	GO:0031146	SCF-dependent proteasomal ubiquitin-dependent protein catabolic process	HGNC=14150	FBXL16
Cell Movement	GO:0007018	microtubule-based movement	HGNC=14566 HGNC=21202	KIF13A , KIF6
Cell Movement	GO:0030336	negative regulation of cell migration	HGNC=21165	RAP2C
Cell Movement	GO:0008045	motor neuron axon guidance	HGNC=9802	RAC2
Cell Movement	GO:0072676	lymphocyte migration	HGNC=16642	CXCL16
Cell Movement	GO:0007098	centrosome cycle	HGNC=20163 HGNC=30532	HAUS4 , HAUS8
Cell Movement	GO:0001578	microtubule bundle formation	HGNC=24164	TPPP
Cell Movement	GO:0007052	mitotic spindle organization	HGNC=24502	WDR62
Cell Movement	GO:0007015	actin filament organization	HGNC=9802 HGNC=2255 HGNC=16932 HGNC=2254	RAC2 , CORO2A , NEBL , CORO1C
Cell Movement	GO:0060294	cilium movement involved in cell motility	HGNC=21057	RSPH9
Cell Movement	GO:0000132	establishment of mitotic spindle orientation	HGNC=978	BBCIP
Cell Movement	GO:0060326	cell chemotaxis	HGNC=1060 HGNC=1609	CXCR5 , CCR8
Cell Movement	GO:0031122	cytoplasmic microtubule organization	HGNC=18600	TUBGCP5
Cell Movement	GO:0030042	actin filament depolymerization	HGNC=243	ADD1
Metabolic Process	GO:0070989	oxidative demethylation	HGNC=25941	TET2
Metabolic Process	GO:1901568	fatty acid derivative metabolic process	HGNC=26222	FAR1
Metabolic Process	GO:0009112	nucleobase metabolic process	HGNC=12805	XDH
Metabolic Process	GO:0008610	lipid biosynthetic process	HGNC=20715	MID1IP1
Metabolic Process	GO:0009143	nucleoside triphosphate catabolic process	HGNC=3356	ENPP1
Metabolic Process	GO:0046890	regulation of lipid biosynthetic process	HGNC=20715	MID1IP1
Metabolic Process	GO:0006208	pyrimidine nucleobase catabolic process	HGNC=3013	DPYS
Metabolic Process	GO:0046034	ATP metabolic process	HGNC=3356	ENPP1
Metabolic Process	GO:0006066	alcohol metabolic process	HGNC=14522	MIOX
Metabolic Process	GO:0009247	glycolipid biosynthetic process	HGNC=24275	SCCPDH
Metabolic Process	GO:0006631	fatty acid metabolic process	HGNC=2329	CPT1B
Metabolic Process	GO:0045761	regulation of adenylate cyclase activity	HGNC=13841 HGNC=240 HGNC=277 HGNC=237	ADGRG6 , ADCY9 , ADRA1A , ADCY6
Protein Regulation	GO:0018205	peptidyl-lysine modification	HGNC=20944	SENP6
Protein Regulation	GO:0045859	regulation of protein kinase activity	HGNC=9309	PPP2R5A
Protein Regulation	GO:0007254	JNK cascade	HGNC=12033	TRAF3
Protein Regulation	GO:0001522	pseudouridine synthesis	HGNC=16060	TRUB1
Protein Regulation	GO:0070475	rRNA base methylation	HGNC=16385	NSUM5
Protein Regulation	GO:0016567	protein ubiquitination	HGNC=30688 HGNC=591	CAND1 , BIRC3
Protein Regulation	GO:0006470	protein dephosphorylation	HGNC=9671 HGNC=9309	PTPRG , PPP2R5A
Protein Regulation	GO:0000186	activation of MAPKK activity	HGNC=6857	MAP3K5
Protein Regulation	GO:0070534	protein K63-linked ubiquitination	HGNC=12033	TRAF3
Protein Regulation	GO:0016485	protein processing	HGNC=591 HGNC=17091	BIRC3
Protein Regulation	GO:0006497	protein lipidation	HGNC=25072	WDR45B
Cellular Homeostasis	GO:0050803	regulation of synapse structure or activity	HGNC=20151	SLC17A8
Cellular Homeostasis	GO:0055067	monovalent inorganic cation homeostasis	HGNC=10911	SLC12A2
Cellular Homeostasis	GO:0051208	sequestering of calcium ion	HGNC=9009	PKD2
Cellular Homeostasis	GO:0051235	maintenance of location	HGNC=3976 HGNC=11214 HGNC=1908	FTH1 , SPAG4 , VPS13A
Cellular Homeostasis	GO:0019725	cellular homeostasis	HGNC=10911	SLC12A2
Cellular Homeostasis	GO:0006882	cellular zinc ion homeostasis	HGNC=4927	SLC39A7
Cellular Homeostasis	GO:0050801	ion homeostasis	HGNC=14544	WNK4
Cellular Homeostasis	GO:0051453	regulation of intracellular pH	HGNC=16438	SLC4A11
Cellular Homeostasis	GO:0042391	regulation of membrane potential	HGNC=13814 HGNC=1955 HGNC=4328 HGNC=31715	KCNK15 , CHRNA1 , GLRA3 , GLRA4
Cellular Homeostasis	GO:0008361	regulation of cell size	HGNC=10911	SLC12A2
Other	GO:0044262	cellular carbohydrate metabolic process	HGNC=4696 HGNC=14522	GUSB , MIOX
Other	GO:0009108	coenzyme biosynthetic process	HGNC=9755	QPRT
Other	GO:0017144	drug metabolic process	HGNC=9755 HGNC=8524 HGNC=20243	QPRT , OVGP1 , CYP2W1
Other	GO:0007163	establishment or maintenance of cell polarity	HGNC=9802	RAC2
Other	GO:0006801	superoxide metabolic process	HGNC=3062 HGNC=7890 HGNC=1613	DUOX1 , NOX3 , CCS
Other	GO:0001525	angiogenesis	HGNC=22989	COL22A1
Other	GO:0045777	positive regulation of blood pressure	HGNC=277	ADRA1A
Other	GO:0008284	positive regulation of cell proliferation	HGNC=5967	Il11RA
Other	GO:0006457	protein folding	HGNC=28115	TBCEL
Detection of Stimulus	GO:0006298	mismatch repair	HGNC=7527	MUTYH
Detection of Stimulus	GO:0006284	base-excision repair	HGNC=7527 HGNC=587	MUTYH , APEX1
Detection of Stimulus	GO:0042060	wound healing	HGNC=3052	DSP
Detection of Stimulus	GO:0009612	response to mechanical stimulus	HGNC=9009 HGNC=18053 HGNC=9012 HGNC=9015	PKD2 , PKD1L1 , PKD2L2 , PKDREJ
Detection of Stimulus	GO:0009314	response to radiation	HGNC=6990	MECP2
Detection of Stimulus	GO:0009411	response to UV	HGNC=2718	DDB2
Detection of Stimulus	GO:0032675	regulation of interleukin-6 production	HGNC=15632	**TLR8**
Regulation of Cell Division	GO:0000086	G2/M transition of mitotic cell cycle	HGNC=7652	NBN
Regulation of Cell Division	GO:0006281	DNA repair	HGNC=2718	DDB2
Regulation of Cell Division	GO:0044773	mitotic DNA damage checkpoint	HGNC=7652	NBN
Regulation of Cell Division	GO:0010564	regulation of cell cycle process	HGNC=20944	SENP6
Regulation of Cell Division	GO:0000724	double-strand break repair via homologous recombination	HGNC=7652	NBN
Regulation of Cell Division	GO:0007134	meiotic telophase I	HGNC=29814 HGNC=27729	MUS81 , CTXN3
Regulation of Cell Division	GO:0051302	regulation of cell division	HGNC=26624	KDF1
Regulation of Cell Division	GO:0061640	cytoskeleton-dependent cytokinesis	HGNC=25589	SEPTIN11
Enzyme Activity	GO:0007202	activation of phospholipase C activity	HGNC=3538 HGNC=277	F2RL1 , ADRA1A
Enzyme Activity	GO:0043547	positive regulation of GTPase activity	HGNC=25418 HGNC=17791 HGNC=20310 HGNC=24715	RUNDC1 , TBC1D8 , GRTP1 , TBC1D8B
Enzyme Activity	GO:0010951	negative regulation of endopeptidase activity	HGNC=15995 HGNC=6383 HGNC=8584 HGNC=8941 HGNC=11345	SERPINA9 , KNG1 , SERPINB2 , RECK
Enzyme Activity	GO:0043433	negative regulation of sequence-specific DNA binding transcription factor activity	HGNC=12033	TRAF3
Apoptotic Process	GO:0051402	neuron apoptotic process	HGNC=7808	NGF
Apoptotic Process	GO:0043066	negative regulation of apoptotic process	HGNC=7808	NGF
Apoptotic Process	GO:0097193	intrinsic apoptotic signaling pathway	HGNC=18165	STYXL1
Apoptotic Process	GO:0042771	intrinsic apoptotic signaling pathway in response to DNA damage by p53 class mediator	HGNC=25722	AEN

TABLE 5

TLR8 Forward 5'- CAGAAACATGGAAAACATG TTCCTTCA GTCGTCAATGC-3'	Sigma-Aldrich	This paper
TLR8-2 Reverse 5'-CACATGCCAGACACCAG TGCTGTCATAACC ATGGTGGTGATAAAGAACG-3'	Sigma-Aldrich	This paper
TLR8-2 Forward 5'CGTTCTTT ATCACCACCATGGTTATGACAGCACTGGTGTCTGGCATGTG- 3'	Sigma-Aldrich	This paper
TLR2-TM Reverse 5'-CCTAGGACTTTATCGCAGCTC TCAGATTTACCCAAAATCC-3'	Sigma-Aldrich	This paper
PCR1 CRISPR screen: Fwd 5'-AGGGCCTATTTCCCATGATTCCTT-3'	Sigma-Aldrich	This paper
Rev 5'-TCAAAAAAGCACCGACTCG-3'	Sigma-Aldrich	This paper
Illumina amplification PCR Fwd 5'-AATGATACGGCGACCACCGAGATCTACACTCTCTTGTGGAAAGGACGAGGTACCG-3'	Sigma-Aldrich	This paper
Illumina amplification PCR Rev 5'-CAAGCAGAAGACGGCATACGAGAT [TCACTGT]GTGACTGGAGTTCAGACG TGTGCTCTTCCGATCTATTTTAACTTGCTATTTC TAGCTCTAAAAC-3'	Sigma-Aldrich	This paper
CAAUUAAUAUAGAUCGUUU<br>CUGGGAUG UUUGGUUAUA<br>CUAUCAACUUGGGUAUUAA<br>GUCUUGACUGAAAAUGAUU	Accell SMARTpool siRNA for Human TLR8	E-004715-00-0010
GCAAUAACUACGUUUUCUA<br>UUGUGACCGCAAUGGUAU<br>UUCUCAUCUCACAAAAUUG<br>UCUUUAUGUCACUAGUUAU	Accell SMARTpool siRNA for Human TLR2	E-005120-00-0010
GCAGCAUAUAUAAUUCAUG<br>UCACUAUGCUCGAUCUUUC<br>UUGGAUGUAGGAUUUAAC<br>CUGUUAGCCAUGAAGUUGC	Accell SMARTpool siRNA for Human TLR3	E-007745-00-0010
CUAGCUUUCUUAAAUCUUA<br>CUCUCUACCUUAAUAUUGA<br>UUCUGGACUAUCAAGUUUA<br>CCUAUAAGCUAAUAUCAUA	Accell SMARTpool siRNA for Human TLR4	E-008088-00-0010
CUAUCGUGCAUCUAUGAAU<br>CUGUGAUGCUGUGUGGUUU<br>CUAUGAUGCUUUUAUUGUG<br>GUAUCAGCGUCUAAUAUCA	Accell SMARTpool siRNA for Human TLR7	E-004714-00-0010
GCCACAACUUCAGCUUCGU<br>CUUGGAUCUGUCACGGAAC<br>CCUUCGUGGUCUUCGACAA<br>CCUGCAAAUACUAGAUGUA	Accell SMARTpool siRNA for Human TLR9	E-004066-00-0010
CCUUCGUCCUGCAUCACUU<br>CGUUCAGGUCGAAAGCUUC<br>GGCUUAUCCAGAAUCAGAU<br>CCAGCAGUCCUAUGAGUUU	Accell SMARTpool siRNA for Human NOD1	E-004398-00-0010
GCCACAUGCAAGAAGUAUA<br>CGAGCAAUUGCAGAAGUUA<br>GCUUUAGGAUGUACAGUUA<br>CUGUUAACCUUUGAUGGCU	Accell SMARTpool siRNA for Human NOD2	E-003464-00-0010
GUAUAAUCAUGGAUCGCUU<br>GGAUUAGCGACAAAUUUAA<br>CUGACAUACAGAUUUUCUA<br>UCUUGAUGCGUCAGUGAUA	Accell SMARTpool siRNA for Human RIG-1, DDX58	E-012511-00-0010
UGGUUUACAUGUCGACUAA<br>UGGUUUACAUGUUUUCUGA<br>UGGUUUACAUGUUUUCCUA<br>UGGUUUACAUGUUGUGUGA.	Accell SMARTpool siRNA for Human non-targeting control	D-001910-10-20
